# The Identification of *MATE* Antisense Transcripts in Soybean Using Strand-Specific RNA-Seq Datasets

**DOI:** 10.3390/genes13020228

**Published:** 2022-01-26

**Authors:** Yee-Shan Ku, Xiao Lin, Kejing Fan, Sau-Shan Cheng, Ting-Fung Chan, Gyuhwa Chung, Hon-Ming Lam

**Affiliations:** 1 Centre for Soybean Research of the State Key Laboratory of Agrobiotechnology and School of Life Sciences, The Chinese University of Hong Kong, Hong Kong, China; ysamyku@cuhk.edu.hk (Y.-S.K.); alanlamsiu@gmail.com (X.L.); kejing68164614@gmail.com (K.F.); chengsaushan@yahoo.com (S.-S.C.); tf.chan@cuhk.edu.hk (T.-F.C.); 2 Department of Biotechnology, Chonnam National University, Yeosu 59626, Korea; chung@chonnam.ac.kr

**Keywords:** MATE (multidrug and toxic compound extrusion) transporter, natural antisense transcript (NAT), sense transcript, soybean, strand-specific RNA-seq

## Abstract

Natural antisense transcripts (NATs) have been generally reported as negative regulators of their sense counterparts. Multidrug and toxic compound extrusion (MATE) proteins mediate the transport of various substrates. Although *MATE*s have been identified genome-wide in various plant species, their transcript regulators remain unclear. Here, using the publicly available strand-specific RNA-seq datasets of *Glycine soja* (wild soybean) which have the data from various tissues including developing pods, developing seeds, embryos, cotyledons and hypocotyls, roots, apical buds, stems, and flowers, we identified 35 antisense transcripts of *MATE*s from 28 gene loci after transcriptome assembly. Spearman correlation coefficients suggested the positive expression correlations of eight *MATE* antisense and sense transcript pairs. By aligning the identified transcripts with the reference genome of *Glycine max* (cultivated soybean), the *MATE* antisense and sense transcript pairs were identified. Using soybean C08 (*Glycine max*), in developing pods and seeds, the positive correlations between *MATE* antisense and sense transcript pairs were shown by RT-qPCR. These findings suggest that soybean antisense transcripts are not necessarily negative transcription regulators of their sense counterparts. This study enhances the existing knowledge on the transcription regulation of MATE transporters by uncovering the previously unknown *MATE* antisense transcripts and their potential synergetic effects on sense transcripts.

## 1. Introduction

Multidrug and toxic compound extrusion (MATE) transporters typically consist of 12 transmembrane domains (TMDs) [1] and play important roles in cellular transport, metabolism, and physiology [2,3,4]. MATEs are ancient proteins that can be found in all three domains of life [3,5]. Previous research suggested that prokaryotic MATE transporters use H^+^ or Na^+^ in exchange for their substrates such as ions and secondary metabolites while most eukaryotic MATE transporters use H^+^ in exchange for their target substrates [3,5]. In plants, MATE transporters have been reported to be involved in the transportation of various substrates, including ion chelators, phytohormones, alkaloids and flavonoids [4,6,7]. In terms of biological processes, MATE proteins have been reported to regulate various processes such as detoxification, stress tolerance, growth and development, and senescence of plants [8,9]. The diverse substrate specificities of MATE proteins to transport various substrates and regulate diverse biological processes could be the possible reason rendering the big families of *MATE*s in plant genomes. In a genome-wide survey based on the presence of the conserved MATE domain, 117 *MATE* genes were identified in the soybean genome [10]. Similarly, 49 *MATE* genes were identified in *Zea mays* [11], 67 in *Solanum lycopersicum* [12], 71 in *Populus trichocarpa* [13], 53 in *Oryza sativa* [14], 56 in *Arabidopsis thaliana* [15], 40 in *Medicago truncatula* [16], and 65 in *Vitis* *vinifera* [17]. Although MATE proteins are structurally similar, as characterized by the typical 12 TMDs [1], they have different substrate specificities [4,6,7], probably due to different substrate recognitions by different amino acid residues [6]. Some substrates transported by MATE proteins are specific to particular groups of plants. For example, anthocyanins are especially rich in the skin of berries and colored seed coats while isoflavones are especially rich the seeds of legumes [18]. VvMATE1 and VvMATE2 were reported to be putative proanthocyanidin transporters in seed berries [19] while GmMATE1 and GmMATE2 were reported to mediate isoflavone transport and storage in soybean seeds [7]. The rich diversity of metabolites in particular plant species hints at the need for a large number of transporters and thus the big MATE families in plants compared to other eukaryotes. Since *MATE* genes are differentially expressed in different tissue types and different developmental stages [7,20], it is plausible that there are additional regulatory roles that these genes play besides transcriptional regulation.

Natural antisense transcripts (NATs) were believed to negatively regulate their corresponding sense transcripts [21]. For example, in the model plant *Arabidopsis*, the antisense transcripts covering the entire *FLOWERING LOCUS C* (*FLC* locus) were suggested to silence *FLC* transcripts [22]; the antisense transcript of *DOG1* (*Delay of Germination 1*) was reported to suppress the expression of the sense *DOG1* transcript [23]; *CDF5* (*CYCLING DOF FACTOR 5*) transcript and its antisense pair transcript *FLORE* (*CDF5 LONG NONCODING RNA*) were reported to inhibit each other’s expression [24]. However, accumulated evidence suggested that NATs are not necessarily the negative regulators of their sense counterparts [25]. For example, also in the model plant *Arabidopsis*, in a global analysis of antisense and sense transcript pairs that result in elevated small RNA levels, in terms of expression, the transcript pairs could have negative correlations, no correlations, or positive correlations [26]. In another study, *MAS*, the antisense transcript of *MAF4* (*MADS AFFECTING FLOWRING4*), was reported to be the positive regulator of the transcription of its sense counterpart [27].

The previous soybean NAT database was built using many fewer tissue types [28] or predicted only by genome annotation [29]. Based on the annotation information, transcript orientation, and the degree of overlapping, in the Plant Natural Antisense Transcripts DataBase (PlantNATsDB), it was predicted that there are 436 *cis*-NATs and 77,903 *trans*-NATs in soybean [29]. In another genome-wide identification of NATs in soybean, in which sequences of small RNAs were aligned to the predicted cDNA sequences from the soybean gene sequence database, 994 *cis*-NATs and 25,222 *trans*-NATs were predicted [28]. However, such predictions relied heavily on the accuracy of transcript annotation [29] and had possibly left out NATs that do not lead to small RNA generation [28].

In this study, we performed a more robust genome-wide analysis to identify NATs that may regulate *MATE* genes in soybean.

## 2. Materials and Methods

### 2.1. Transcriptome Assembly and MATE Antisense Transcripts Identification

The antisense transcript identification was performed with the transcriptome datasets of the wild soybean accessions (*Glycine soja*) since *Glycine soja* has more comprehensive strand-specific RNA-seq data from different tissues compared to cultivated soybean (*Glycine max*) [30,31]. The strand-specific RNA-seq datasets used for transcriptome assembly and MATE antisense transcript identification are summarized in Table S1. Publicly available RNA-seq datasets of *Glycine soja* (Table S1) were collected. After that, adapter trimming and quality trimming were performed with the raw transcriptome reads using Trimmomatic (0.36) [32]. The clean read data were then aligned to the reference genome of *Glycine soja* (W05) [31] using HISAT2 (2.1.0) [33], followed by de novo transcriptome assembly using StringTie (1.3.4d) [34]. *De novo* transcriptome assembly was performed for each sample. After that, the data were merged using the merge function of StringTie [34]. The merged transcriptome data were integrated with the reference annotation of the reference genome of *Glycine soja* (W05) [31] using the transcript annotation script (NIAP_annotate_v1.1.pl) in the NIAP package (https://github.com/alanlamsiu/NIAP, last accessed on 9 April 2021). This merged transcriptome annotation was then used for *MATE* antisense transcript identification and expression analysis. Antisense transcripts of *MATE* loci were identified by intersecting the merged transcriptome annotation and predicted *MATE* loci for W05 [31] at opposite strands using BEDTools (v2.27.1) [35]. The sequences for these sense and antisense transcripts of *MATE*s from soybean accession W05 were then extracted. Considering that most research on soybean is done with cultivated soybean (*Glycine max*), the corresponding *MATE* antisense and sense transcripts in cultivated soybean were identified with the use of the reference genome of cultivated soybean (Gmax_275_Wm82.a2.v1) [36]. Reciprocal BLAST was performed with all protein sequences of the accession Williams 82 (Wm82) (Gmax_275_Wm82.a2.v1 [36]) and W05 [31] to identify corresponding genes between Wm82 and W05. The transcript sequences of W05 was aligned with the genome sequence of Wm82 (Gmax_275_Wm82.a2.v1 [36]) to identify the corresponding *MATE* antisense and sense transcripts, which were used later in designing the primers for the RT-qPCR validation experiment. Sequences and coordinates of the W05 and Wm82 antisense transcripts can be found in Supplementary Materials S1 and S2. Genomic loci in W05 corresponding to genes in Wm82 which were annotated as *MATE* [10] were selected for *MATE* antisense transcript identification.

### 2.2. Plant Sample Preparation

Soybean plants (cultivated soybean accession C08 (*Glycine max*) [37]), were grown on an experimental field in the Chinese University of Hong Kong, watered twice a day, under normal conditions. The developing pods were harvested at 40 days after flowering (DAF). For each biological replicate, the pods were harvested from three individual plants. Two biological replicates were harvested and used for RNA extraction, followed by expression analysis. The pod shells and the seeds with seed coat removed were frozen in liquid nitrogen, then stored at −80 °C before RNA extraction.

### 2.3. RNA Extraction from Plant Samples

Total RNA was extracted from the frozen pods and seeds without seed coat using Fruit-mate (Cat# 9192, TaKaRa, Shiga, Japan) supplemented with RNasin (recombinant, Cat# N2515, Promega, Madison, WI, USA), and TRIzol Reagent (Cat# 15596018, ThermoFisher Scientific, Waltham, MA, USA) according to the manufacturers’ instructions. The total RNA was treated with DNase I (Cat# 18068015, ThermoFisher Scientific, Waltham, MA, USA) according to the manufacturer’s protocol and then used for expression analysis.

### 2.4. Expression Analysis

The expression levels of the antisense and sense transcripts were validated using One Step PrimeScript RT-PCR Kit (Perfect Real Time) (Cat# RR064B, TaKaRa, Shiga, Japan) according to the manufacturer’s protocol with the following modifications. The reverse primer was added to the DNaseI-treated RNA for reverse transcription. After that, the forward primer was added to the reaction mix for qPCR. The relative expressions of the antisense and sense transcripts were calculated using the 2^−ΔΔCT^ method with *VPS* as the normalizing gene [38]. Primers used in the experiments are listed in Table S2. Two biological replicates, with each replicate having the total RNA extracted from seeds (seed coats removed) or pods from three individual plants, were used for the expression analysis. Three RT-qPCR reactions were done for each template and primer pair combination. One RT-qPCR was regarded as one technical replicate.

## 3. Results

### 3.1. Genome-Wide Identification of MATE Antisense Transcripts

**Figure 1:**
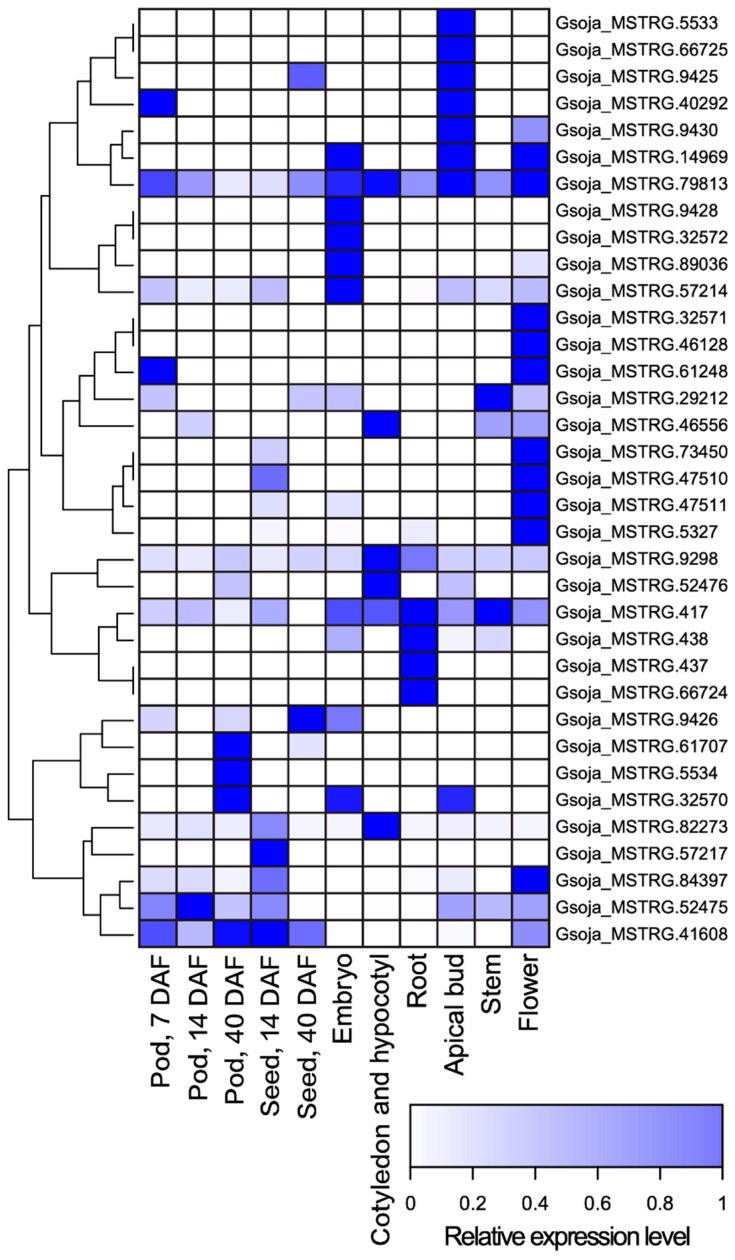
A heatmap of the predicted relative expression levels of *MATE* antisense transcripts identified from strand-specific RNA-seq datasets of pod (7 DAF), pod (14 DAF), pod (40 DAF), seed (14 DAF), seed (40 DAF), embryo, cotyledon and hypocotyl, root, apical bud, stem, and flower of soybean accession W05 (Table S1) [30,31].

Using the publicly available, strand-specific RNA-seq datasets of a wide variety of tissue types including developing pods, developing seeds, embryos, cotyledons and hypocotyls, roots, apical buds, stems, and flowers, we identified 35 antisense transcripts of *MATE*s from 28 gene loci in the wild soybean accession W05 (Supplementary Material S1). The predicted relative expression levels of these antisense transcripts are illustrated in Figure 1. Subsequently, we identified the antisense *MATE* transcripts in the Wm82 by aligning the antisense transcripts identified from strand-specific RNA-seq datasets of W05 to the reference genome of Wm82 (Gmax_275_Wm82.a2.v1 [36]) (Supplementary Material S2). 

### 3.2. Predicted Expression Correlations between MATE Antisense and Their Respective Sense Transcripts

**Figure 2:**
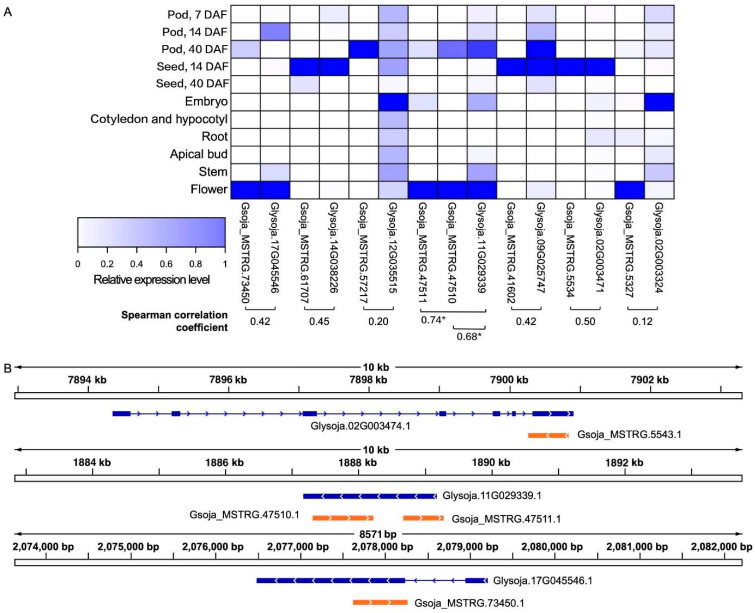
Antisense *MATE* transcripts and their sense counterparts in soybean accession W05 with positively correlated expression levels identified based on the publicly available RNA-seq datasets. Left panel. (**A**) A heatmap of the expression levels of the antisense-sense transcript pairs (joined by a square bracket) that are predicted to be correlated, with the corresponding Spearman correlation coefficients. *Gsoja_MSTRG.47510 and Gsoja_MSTRG.47511 are both antisense transcripts corresponding to the sense transcript Glysoja.11G029339, with the Spearman correlation coefficients being 0.68 and 0.74 respectively; (**B**) the genomic locations of three of the antisense-sense transcript pairs having high Spearman correlation coefficients are showcased.

Being NATs, the antisense *MATE* transcripts could potentially regulate the levels of the respective sense transcripts due to the sequence complementarity. As reflected by the Spearman correlation coefficients, there are several antisense *MATE* transcripts that exhibited positive expression correlations with their respective sense transcripts in the dataset (Figure 2).

### 3.3. Experimental Validations and Expression Analyses of the Positively Correlated Antisense-Sense MATE Transcript Pairs

**Figure 3:**
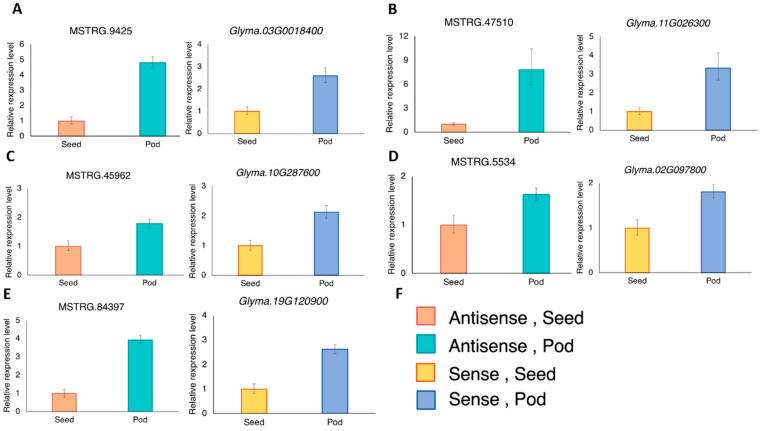
The expression levels of the antisense and their corresponding sense transcripts in the developing seed and pod were analyzed using RT-qPCR. The antisense and sense pairs are (**A**) MSTRG.9425 and *Glyma03G0018400*; (**B**) MSTRG.47510 and *Glyma.11G026300*; (**C**) MSTRG.45962 and *Glyma.10G287600*; (**D**) MSTRG.5534 and *Glyma.02G097800*; (**E**) MSTRG.84397 and *Glyma.19G120900* (*GmMATE4* [20]); (**F**) The color key of the bars shown in panel A–E. Three RT-qPCR reactions were done for each template and primer pair combination. One RT-qPCR was regarded as one technical replicate. Each bar represents the mean of three technical replicates ± standard deviation. The expression was normalized to the reference gene, *VPS* [38] using the 2^−ΔΔCT^ method. Similar expression trends were obtained from another biological replicate using a separate set of seed and pod from three other plants. Results of the biological replicates are shown in Supplementary Figure S1.

MATE-type proteins are known to be transporters of secondary metabolites [18]. During pod development, nutrients are actively accumulated inside the developing seeds. In this study, developing pods of the soybean accession C08 at 40 DAF, the stage of active seed-filling [39], were used for experimental validation of the transcripts and comparisons of the expression levels between seed and pod (Figure 3). In line with the expression correlations between the antisense and the respective sense transcripts of soybean accession W05 (Figure 2), the antisense and sense *MATE* transcripts from soybean accession C08 showed similar expression trends between the developing seed and pod (Figure 3).

## 4. Discussion

MATE transporters have diverse physiological roles in growth, development, and stress responses [4,6]. The expression of *MATE* genes also exhibits differential patterns to cope with their functions [7,13,20]. *MATE* genes display tissue specific expression patterns. For example, the soybean genes *GmMATE1*, *GmMATE2*, and *GmMATE4* were shown to have differential expression levels in different tissues such as seeds, seed coats, and pods [7,20]. In *Hordeum vulgare*, *HvAACT1* is expressed in the whole root but has a higher expression level in the root tip [40]. In *Nicotiana tabacum*, *Nt-JAT1* is expressed in leaf, stem, and root [41] while *NtJAT2* is expressed in leaf but not stem or root [42]. In terms of the response to stimuli, both *Nt-JAT1* and *Nt-JAT2* were shown to be induced by methyl jasmonic acid treatment [41,42]. In *Populus poplar*, *PtrMATE1* and *PtrMATE2* were shown to be induced by aluminum stress [13]. In *Solanum lycopersicu*, *Sl-ALMT9* was also demonstrated to be induced by aluminum stress [43]. The diverse expression patterns and responses to stimuli of *MATE* genes imply the possible existence of transcript regulators.

In this study, we identified novel antisense transcripts of *MATE*s and investigated the expression correlation between NATs and their sense transcripts to reveal the possible negative regulatory roles or the synergetic effects of the NATs on their sense transcripts. 

We successfully employed a whole-genome approach to identify high confident NATs of the *MATE* transcript (Figure 1). These antisense transcripts were not listed in existing databases of soybean antisense transcripts [28,29]. We were able to validate the expressions of the predicted antisense transcripts using RT-qPCR (Figure 3). Among the experimentally validated transcripts (Figure 3), *GmMATE4* (Glyma.19G120900, Figure 3E) was previously reported to be localized in the overlapping QTLs regulating the content of antioxidants, phenolics, and flavonoids in soybean seeds [20]. The antisense transcript of *GmMATE4* was not reported in the previous study on the expression patterns in developing soybean seeds and pods [20]. The identification of novel *MATE* antisense transcripts enriches the knowledge of the expression of *MATE* genes in soybean.

Surprisingly, we found that the relative expression levels of many of the *MATE* NATs are positively correlated to the corresponding sense transcripts (Figure 2). Whether NATs promote or reduce the target transcript stability has been a subject of debate [25,44]. Nevertheless, there has been accumulating evidence to support the synergy brought forth by *cis*-NATs on the sense mRNA [25]. In the global identification of *Arabidopsis* long non-coding RNAs (lncRNAs), based on the Pearson correlation coefficients, the strong tendency of NATs to positively regulate the expression of their sense overlapping genes was suggested [27]. *MAS*, the NAT overlapping the sense transcript *of MADS AFFECTING FLOWERING4* (*MAF4*), was found to be promoting *MAF4* expression at the transcriptional level [27]. It was reported that *MAS* recruits WDR5a, a core component of COMPASS-like complexes, to enhance histone 3 lysine 4 trimethylation (H3K4me3), which brings forth transcriptional activation of *MAF4* [27].

In this study, we predicted positive correlation between antisense transcripts of *MATE*s and their corresponding sense transcripts based on the Spearman correlation coefficients (Figure 2), and validated the positive correlation of the antisense and sense pairs in developing seeds and pods by RT-qPCR (Figure 3). These results are in line with the possible synergetic effects of the antisense transcripts on the expression of their sense counterparts. 

## 5. Conclusions

In this study, using strand-specific RNA-seq datasets from the tissues of soybean accession W05 and the reference grade genome of W05 [31], we identified a set of *MATE* antisense transcripts. Based on Spearman correlation coefficients, several antisense transcripts were predicted to have positive correlations with their sense counterparts. By aligning the antisense transcripts identified in the dataset to the accession Williams 82 (Wm82) (Gmax_275_Wm82.a2.v1 [36]), *MATE* antisense transcripts in Wm82 were identified. The expressions of the antisense transcripts and their sense counterparts were validated in developing soybean seeds and pods. Results showed positively correlated differential expression patterns of the antisense transcripts and their corresponding sense transcripts in both seed and pod. This is consistent with a possible synergistic effect on sense transcript expressions by the antisense counterparts. This study provides new information on the genome-wide identification of *MATE* genes, demonstrates the enhanced capacity to detect novel antisense transcripts using strand-specific RNA-seq datasets, and enriches our understanding on the possible role of antisense transcripts in transcription regulation.

## Data Availability

All data generated in this study are available within this manuscript and companion Supplementary Materials.

## Supplementary Materials

The following are available online at https://www.mdpi.com/article/10.3390/genes13020228/s1, Figure S1: The expression levels of the antisense and their corresponding sense transcripts in the developing seed and pod of the second biological replicate were analyzed using RT-qPCR. Supplementary Material S1: Sequences, chromosomal positions, and strand information of *MATE* antisense transcripts identified in *Glycine soja* in this study. Supplementary Material S2: Sequences, chromosomal position, and strand information of *Glycine max MATE* antisense transcripts predicted from the *Glycine soja MATE* antisense transcripts identified in this study. Table S1: List of strand-specific RNA-seq datasets of *Glycine soja* used for transcriptome assembly and *MATE* antisense transcript identification. Table S2: List of primers used in this study.

## References

[1] Shoji T., Inai K., Yazaki Y., Sato Y., Takase H., Shitan N., Yazaki K., Goto Y., Toyooka K., Matsuoka K. (2008). Multidrug and toxic compound extrusion-type transporters implicated in vacuolar sequestration of nicotine in tobacco roots. Plant Physiol..

[2] Meyer S., De Angeli A., Fernie A.R., Martinoia E. (2009). Intra- and extra-cellular excretion of carboxylates. Trends Plant Sci..

[3] Omote H., Hiasa M., Matsumoto T., Otsuka M., Moriyama Y. (2006). The MATE proteins as fundamental transporters of metabolic and xenobiotic organic cations. Trends Pharmacol. Sci..

[4] Takanashi K., Shitan N., Yazaki K. (2014). The multidrug and toxic compound extrusion (MATE) family in plants. Plant Biotechnol..

[5] Lu M. (2016). Structures of multidrug and toxic compound extrusion transporters and their mechanistic implications. Channels.

[6] Kusakizako T., Miyauchi H., Ishitani R., Nureki O. (2020). Structural biology of the multidrug and toxic compound extrusion superfamily transporters. Biochim. Biophys. Acta Biomembr..

[7] Ng M.-S., Ku Y.-S., Yung W.-S., Cheng S.-S., Man C.-K., Yang L., Song S., Chung G., Lam H.-M. (2021). MATE-type proteins are responsible for isoflavone transportation and accumulation in soybean seeds. Int. J. Mol. Sci..

[8] Upadhyay N., Kar D., Deepak Mahajan B., Nanda S., Rahiman R., Panchakshari N., Bhagavatula L., Datta S. (2019). The multitasking abilities of MATE transporters in plants. J. Exp. Bot..

[9] Tang R.J., Luan M., Wang C., Lhamo D., Yang Y., Zhao F.-G., Lan W.-Z., Fu A.-G., Luan S. (2020). Plant membrane transport research in the post-genomic era. Plant Commun..

[10] Liu J., Li Y., Wang W., Gai J., Li Y. (2016). Genome-wide analysis of MATE transporters and expression patterns of a subgroup of *MATE* genes in response to aluminum toxicity in soybean. BMC Genom..

[11] Zhu H., Wu J., Jiang Y., Jin J., Zhou W., Wang Y., Han G., Zhao Y., Cheng B. (2016). Genomewide analysis of *MATE*-type gene family in maize reveals microsynteny and their expression patterns under aluminum treatment. J. Genet..

[12] dos Santos A.L., Chaves-Silva S., Yang L., Maia L.G.S., Chalfun-Júnior A., Sinharoy S., Zhao J., Benedito V.A. (2017). Global analysis of the MATE gene family of metabolite transporters in tomato. BMC Plant Biol..

[13] Li N., Meng H., Xing H., Liang L., Zhao X., Luo K. (2017). Genome-wide analysis of MATE transporters and molecular characterization of aluminum resistance in *Populus*. J. Exp. Bot..

[14] Tiwari M., Sharma D., Singh M., Tripathi R.D., Trivedi P.K. (2014). Expression of OsMATE1 and OsMATE2 alters development, stress responses and pathogen susceptibility in *Arabidopsis*. Sci. Rep..

[15] Li L., He Z., Pandey G.K., Tsuchiya T., Luan S. (2002). Functional cloning and characterization of a plant efflux carrier for multidrug and heavy metal detoxification. J. Biol. Chem..

[16] Zhao J., Dixon R.A. (2009). MATE transporters facilitate vacuolar uptake of epicatechin 3′-O-glucoside for proanthocyanidin biosynthesis in Medicago truncatula and Arabidopsis. Plant Cell.

[17] Gomez C., Terrier N., Torregrosa L., Vialet S., Fournier-Level A., Verries C., Souquet J.-M., Mazauric J.-P., Klein M., Cheynier V. (2009). Grapevine MATE-type proteins act as vacuolar H^+^-dependent acylated anthocyanin transporters. Plant Physiol..

[18] Ku Y.-S., Ng M.-S., Cheng S.-S., Lo A.W.-Y., Xiao Z., Shin T.-S., Chung G., Lam H.-M. (2020). Understanding the composition, biosynthesis, accumulation and transport of flavonoids in crops for the promotion of crops as healthy sources of flavonoids for human consumption. Nutrients.

[19] Pérez-Díaz R., Ryngajllo M., Pérez-Díaz J., Peña-Cortés H., Casaretto J.A., González-Villanueva E., Ruiz-Lara S. (2014). *VvMATE1* and *VvMATE2* encode putative proanthocyanidin transporters expressed during berry development in *Vitis vinifera* L.. Plant Cell Rep..

[20] Li M.W., Muñoz N.B., Wong C.F., Wong F.L., Wong K.S., Wong J.W.H., Qi X., Li K.P., Ng M.S., Lam H.M. (2016). QTLs regulating the contents of antioxidants, phenolics, and flavonoids in soybean seeds share a common genomic region. Front. Plant Sci..

[21] Vanhée-Brossollet C., Vaquero C. (1998). Do natural antisense transcripts make sense in eukaryotes?. Gene.

[22] Swiezewski S., Liu F., Magusin A., Dean C. (2009). Cold-induced silencing by long antisense transcripts of an *Arabidopsis* Polycomb target. Nature.

[23] Fedak H., Palusinska M., Krzyczmonik K., Brzezniak L., Yatusevich R., Pietras Z., Kaczanowski S., Swiezewski S. (2016). Control of seed dormancy in *Arabidopsis* by a *cis*-acting noncoding antisense transcript. Proc. Natl. Acad. Sci. USA.

[24] Henriques R., Wang H., Liu J., Boix M., Huang L.-F., Chua N.H. (2017). The antiphasic regulatory module comprising *CDF5* and its antisense RNA *FLORE* links the circadian clock to photoperiodic flowering. New Phytol..

[25] Reis R.S., Poirier Y. (2021). Making sense of the natural antisense transcript puzzle. Trends Plant Sci..

[26] Tiwari B., Habermann K., Arif M.A., Weil H.L., Garcia-Molina A., Kleine T., Mühlhaus T., Frank W. (2020). Identification of small RNAs during high light acclimation in *Arabidopsis thaliana*. BMC Plant Biol..

[27] Zhao X., Li J., Lian B., Gu H., Li Y., Qi Y. (2018). Global identification of Arabidopsis lncRNAs reveals the regulation of MAF4 by a natural antisense RNA. Nat. Commun..

[28] Zheng H., Qiyan J., Zhiyong N., Hui Z. (2013). Prediction and identification of natural antisense transcripts and their small RNAs in soybean (*Glycine max*). BMC Genom..

[29] Chen D., Yuan C., Zhang J., Zhang Z., Bai L., Meng Y., Chen L.-L., Chen M. (2012). PlantNATsDB: A comprehensive database of plant natural antisense transcripts. Nucleic Acids Res..

[30] Lin X., Lin W., Ku Y.-S., Wong F., Li M.-W., Lam H.-M., Ngai S.-M., Chan T.-F. (2020). Analysis of soybean long non-coding RNAs reveals a subset of small peptide-coding transcripts. Plant Physiol..

[31] Xie M., Chung C.Y.-L., Li M.-W., Wong F.-L., Wang X., Liu A., Wang Z., Leung A.K.-Y., Wong T.-H., Tong S.-W. (2019). A reference-grade wild soybean genome. Nat. Commun..

[32] Bolger A.M., Lohse M., Usadel B. (2014). Trimmomatic: A flexible trimmer for Illumina sequence data. Bioinformatics.

[33] Kim D., Paggi J.M., Park C., Bennett C., Salzberg S.L. (2019). Graph-based genome alignment and genotyping with HISAT2 and HISAT-genotype. Nat. Biotechnol..

[34] Pertea M., Pertea G.M., Antonescu C.M., Chang T.C., Mendell J.T., Salzberg S.L. (2015). StringTie enables improved reconstruction of a transcriptome from RNA-seq reads. Nat. Biotechnol..

[35] Quinlan A.R., Hall I.M. (2010). BEDTools: A flexible suite of utilities for comparing genomic features. Bioinformatics.

[36] Schmutz J., Cannon S.B., Schlueter J., Ma J., Mitros T., Nelson W., Hyten D.L., Song Q., Thelen J.J., Cheng J. (2010). Genome sequence of the palaeopolyploid soybean. Nature.

[37] Lam H.-M., Xu X., Liu X., Chen W., Yang G., Wong F.-L., Li M.-W., He W., Qin N., Wang B. (2010). Resequencing of 31 wild and cultivated soybean genomes identifies patterns of genetic diversity and selection. Nat. Genet..

[38] Yim A.K.-Y., Wong J.W.-H., Ku Y.-S., Qin H., Chan T.-F., Lam H.-M. (2015). Using RNA-seq data to evaluate reference genes suitable for gene expression studies in soybean. PLoS ONE.

[39] Agrawal G.K., Hajduch M., Graham K., Thelen J.J. (2008). In-depth investigation of the soybean seed-filling proteome and comparison with a parallel study of rapeseed. Plant Physiol..

[40] Fujii M., Yokosho K., Yamaji N., Saisho D., Yamane M., Takahashi H., Sato K., Nakazono M., Ma J.F. (2012). Acquisition of aluminium tolerance by modification of a single gene in barley. Nat. Commun..

[41] Morita M., Shitan N., Sawada K., Van Mongtagu M.C.E., Inzéc D., Rischer H., Goossens A., Oksman-caldentey K., Moriyama Y., Yazaki K. (2009). Vacuolar transport of nicotine is mediated by a multidrug and toxic compound extrusion (MATE) transporter in *Nicotiana tabacum*. Proc. Natl. Acad. Sci. USA.

[42] Shitan N., Minami S., Morita M., Hayashida M., Ito S., Takanashi K., Omote H., Moriyama Y., Sugiyama A., Goossens A. (2014). Involvement of the leaf-specific multidrug and toxic compound extrusion (MATE) transporter Nt-JAT2 in vacuolar sequestration of nicotine in *Nicotiana tabacum*. PLoS ONE.

[43] Ye J., Wang X., Hu T., Zhang F., Wang B., Li C., Yang T., Li H., Lu Y., Giovannoni J.J. (2017). An InDel in the promoter of AI-ACTIVATED MALATE TRANSPORTER9 selected during tomato domestication determines fruit malate contents and aluminum tolerance. Plant Cell.

[44] Brophy J.A., Voigt C.A. (2016). Antisense transcription as a tool to tune gene expression. Mol. Syst. Biol..

