# Supplementary material for: The Identification of MATE Antisense Transcripts in Soybean Using Strand-Specific RNA-Seq Datasets
Source: Genes (Basel). 2022 Jan 26;13(2):228. doi: 10.3390/genes13020228 (PMC8871956; doi:10.3390/genes13020228)
Supplement: Supplementary file 1 [file genes-13-00228-s001.zip › Supplementary Figure S1.pdf]

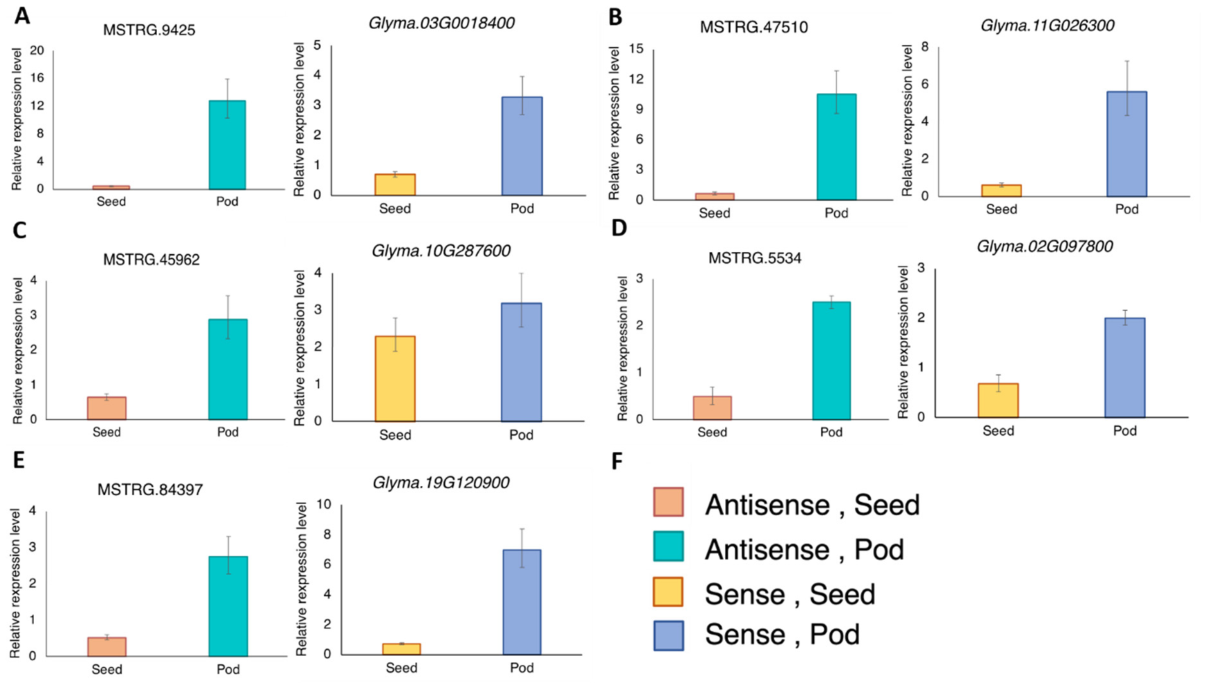

Figure S1. The expression levels of the antisense and their corresponding sense transcripts in the developing seed and pod of the second biological replicate were analyzed using RT-qPCR. The antisense and sense pairs are (A) MSTRG.9425 and *Glyma03G0018400*, (B) MSTRG.47510 and *Glyma.11G026300*, (C) MSTRG.45962 and *Glyma.10G287600*, (D) MSTRG.5534 and *Glyma.02G097800*, (E) MSTRG.84397 and *Glyma.19G120900* (*GmMATE4* [1]). (F) The color key of the bars shown in panel A-E. Three RT-qPCR reactions were done for each template and primer pair combination. One RT-qPCR was regarded as one technical replicate. Each bar represents the mean of three technical replicates $\pm$ standard deviation. The expression was normalized to the reference gene, *VPS*, [2] using the $2^{-\Delta\Delta Ct}$ method.

References

1. Li, M.W.; Muñoz, N.B.; Wong, C.F.; Wong, F.L.; Wong, K. Sen; Wong, J.W.H.; Qi, X.; Li, K.P.; Ng, M.S.; Lam, H.M. QTLs regulating the contents of antioxidants, phenolics, and flavonoids in soybean seeds share a common genomic region. *Front. Plant Sci.* **2016**, *7*, 854.

2. Yim, A.K.-Y.; Wong, J.W.-H.; Ku, Y.-S.; Qin, H.; Chan, T.-F.; Lam, H.-M. Using RNA-seq data to evaluate reference genes suitable for gene expression studies in soybean. *PLoS One* **2015**, *10*, e0136343.
