# Supplementary material for: The Identification of MATE Antisense Transcripts in Soybean Using Strand-Specific RNA-Seq Datasets
Source: Genes (Basel). 2022 Jan 26;13(2):228. doi: 10.3390/genes13020228 (PMC8871956; doi:10.3390/genes13020228)
Supplement: Supplementary file 1 [file genes-13-00228-s001.zip › Supplementary Material 1.pdf]

**Supplementary Material 1:** Sequences, chromosomal positions, and strand information of *MATE* antisense transcripts identified in *Glycine soja* in this study.

>**Gsoja_MSTRG.66727.1** GeneID=Gsoja_MSTRG.66727;Strand=+;Pos=Chr15:8377942-8378384,

ATAAATGGAACCCAACTGAGGTGAGATAGACCTTCTAATATGAGAAATGAAATGAATGTT

TACCATTAGGCCATAGGCGAAGAGCTGAATGCCACTGTTGCCGAGGTTGGCAGCGGCGAG

TTCGAGGTTTCCGAGGTGGCCGGCGAAGGCGCGAGTAACGTTGGACATGAGGTTGTTGAA

GACGTAAACGAGGATGGCGGGAGCGGCGAGGGGGAAGAGGAGGTTGAGTTCGATCCATGT

GGCGGAGAGGATGCGCTTGGACCAGGGTAGGGTGGGGTCAGAGAGGACCTCTTCGAGACG

GGAATCAGCAGAGTGATGATGATGATGCGTTGAGTTTATCAAGGGTTGGCGTAGTAGGTT

CTGGTTCTGGGATTCCATCATGTGTGTGTGTGGGGTTGTCGTGTGTGCTGGAACTATAGA

ATGGGGTACTAAATACTGTTGGG

>**Gsoja_MSTRG.32572.1** GeneID=Gsoja_MSTRG.32572;Strand=-;Pos=Chr08:4000730-4001052,

CATGTTTTTATGAAATTCATTCTGAAATAGGTAGTCATTTGAGTTGTTATTTTTGTTCTT

CCACTGGTTACAATGCACAAAAGTTCTATAAATGTATGTCTTTGAAATTCATGGCCCTTC

CAATCTTTATTCCATTACTTGTTCCCAATTTGGGGCTGTATACAACAACATTAGTCCTTC

GGTGGCAGGTTTTGGGATCAGAATGATAAAGCCAATAACCATAAATTGATTAGGGAACTA

GTTCCTGTTTTTGATGCGGTAATACGCTGTCTCTAGTAGTTGGCGGGATGGTTATGAGTT

CAACTCTCCTTTGAGCTTTCCTT

>**Gsoja_MSTRG.39211.1** GeneID=Gsoja_MSTRG.39211;Strand=-;Pos=Chr09:18277715-18278130,

TGACTGAAAAAAGTAATTTATTTTTCTCATTTGTATACATGCACTCAGAGTTTACATACT

ACTAACAGTACGAAGAATAATCTTCTAAATACGACAAAATATTACAATAAGCAGAGGATA

CATCATTAAACAGAGACCATACAGTAAACATAGGCCAAAAAAAAAGAAAGATACTTTGAA

TATAACACTTGCAAGTGTAAAATGAACCCGTCCAATATAATATATAATAAGACTATCACC

ATAGTTGTATAGTGCATAAATCATGTTTTGAATAAAGACAACTGGTCCTAAATGGTGAAA

AGTGATACAAAACATTTTCTGCTCAACAATGAAATTTCTTTTGTTAGATGTCATCTCATC

ATCAAGACATTGAGCGGCCTCTGAGAAAACGCCATGGTCCCGTACCTGTTCCCATC

>**Gsoja_MSTRG.52475.22** GeneID=Gsoja_MSTRG.52475;Strand=-;Pos=Chr11:45344733-45344898,45345073-45345246,45345421-45345558,45345862-45345960,

GTGGGTAAGATGGAGAGAGTGAGGTTGAGGGAGGGATGGTGGATGGATAGGGCGATGATG

GGTAGGGTGGTTGAGATGTTGGGGAAGGAGTGTTTGGAGGACTATAAATTGGAGAAGGTG

TCTTTGGTGGTGTTGAAACATAGGGAGGTTGGTTATGAGGGGAAGGAGCATTTGGAGGAC

TATAAGCAGGAGATGGTGTTTTTGGAGGAGTAGGAACATTTGGTGGAGTGTAAATAGGTG

AAGGTGTTTTGGGAGGAGTGGGAACATTAGGAGGACTATAAATTGGAGAAGGTGTCTTTG

GTGGTGTAGGAACATAGGGAGGTTGGTTATGAGGGGAAGGAGCATTTGGAGGACTATAAG

CAGGAGATGGTGTTTTTGGAGGAGTAGGAACATTTGGTGGAGTGTAAATAGGTGAAGGTG

TTTTGGGAGGAGTGGGAACATTAGGAGGATTATAAGAAGGAGAAGGTGTTTTTGGAGGTG

TAGGAACATAGGGTGGATTGTTACTAGGGGAAGGAGCTTTTGGGGGAGTGTACACAGGAG

AGGGTATTTTTGGAGGCGTTGGAAAATTAGGAGGAGT

>**Gsoja_MSTRG.15167.1** GeneID=Gsoja_MSTRG.15167;Strand=-;Pos=Chr04:8803608-8804149,8828564-8828668,

CCGTTACTATTATAGAATTATGTATAGAACTTATTTCCTGTCTCAACTAATTTCCATCCA

CGCAACACTTAAAGAAAAAAAAAATGATCCACTCACTTTAATAACAAAGAAACCTTGTCC

AGCTAGTTGTTAGCCAGCAATAAAAGATAAGAAAACAAGGAATCAAAGAAATTAATGATG

ACAGGGTAAATCACGAGCTTCATCACTTTTCTCTCATCTTATCCGTCTCCACAATCCTGC

AATAAAAATTGAAAAATATGATTAATTGAACTTTTTCTGTGCTAAAATATGATTAAAACA

TCTAATTAATTAGTCTTACTAAAGAATATGTAGTAACACCATAGGTTGGAATTACAACTG

TATTGTCGGTGCGTGGGGATCTCCAAATTCAACTCATGGTAATATAACATAGCTATCCTA

AAGAATAACCGGGCCTTTAATTTTGAATACAACAACAACTTGGTGAATATTGAAAGAAAT

ATATATATATGCAGCATCCAATACAACGGCAAAACTCATAGAATCCAGATTGGTGAATAA

CAAACACGATCCATTGGCTAAATCAAATGTTAATTGGAGGCTTCCAGATTGCCATTGGAG

TTAGATTTTGACCACTTACTTACACGAAAGCAAGCTTTCTCCTCCTG

>Gsoja_MSTRG.32571.1 GeneID=Gsoja_MSTRG.32571;Strand=-;Pos=Chr08:3999993-4000225,

GAAAGTGTACCTTGGATCCCATCTAGAAAGCTAGAAAGGGCCAAAACTGGCATCATGAAT

GAAACATATCTGATCACTTCTTTGTCATTACTGTAAACATGTCCCCAGACATTCCGCAGT

AGAATCATGGCTGAAACTAAAATGACCCCTTCAATTAAGGCCATTGATAACACCACCCGA

ACTGCTAGACTTGCGGCTTGTGGATTTCCACTCCCCAGTTCATTGGAGACCCG

>**Gsoja_MSTRG.52475.8** GeneID=Gsoja_MSTRG.52475;Strand=-;Pos=Chr11:45344091-45344898,45345073-45345162,45345262-45346448,

TACAACTCACATTGGTAACCATAGATACACAGATTATACGACATAAAATTAATTATGAGA

CGCGATCTCATTTGTCCAAGTAAAAATATTAGAAAAAATATATAAAAGTGTTCACAATTA

CTATATACTATAACTCAAGTTCCAAAGAAAGTTTGATTTTTCTAATCAATAGTGGTACTT

AGAGCAGCACCGACCAGAACTATAACTCAAGTTCCAAAAAAAAAGTTTGATTTTTCTAACC

AAGAGTGGTGCTTAGAGCAGCACCGACCAGAATGGTTAGTGAATACACTCCAAACCGCGA

CGGCGTTATAGACGACGCACCGTTTAGTCTGGCTGGCGTCGTTTCGTTCGACGACGACGG

CGTTTGAGATGATCCCGACGACGACGACGGCGAGGGGGTTGTTGCCGCCGCAGACGGTAA

TGGCGGCGATGTTGCCGGGGTGAGGGAGGAAGAGGGTGGGTAAGTGGGTGGTGCAGTTGA

AGCAGAAGGTGGGTAAGATGGAGAGAGTGAGGTTGAGGGAGGGATGGTGGATGGATAGGG

CGATGATGGGTAGGGTGGTTGAGATGTTGGGGAAGGAGTGTTTGGAGGTGTTGGATTAAT

GTAAGGTGGTTGAGATGTTGGGGAATGAGAGTTTGGAGGTGCTGCAATGGAAGGAGGTTG

GGAAGTGGGTGAAGGAGTGGTGTTTGGAGGTGTGGGAATGTAAGGTGGTTGAGATGTTGG

AGATGGGGTTATTGGAACAAAGGGTGGTTGATGGGCAGGGGAAGGCGTTTTTGGAGGGGT

TGGAGTGTAGGGAGGTTGAGAAATAGGAGAAGGAGTGTTTGAGGGAGTTGGGACATAAGG

TTGGTTGCCAGGGGGAGGTGTTTTTGGAGTAGTAGTGGGAACATTTGGAGGACTATAAAT

TGGAGAAGGTGTCTTTGGTGGTGTTGAAACATAGGGAGGTTGGTTATGAGGGGAAGGAGC

ATTTGGAGGACTATAAGCAGGAGATGGTGTTTTTGGAGGAGTAGGAACATTTGGTGGAGT

GTAAATAGGTGAAGGTGTTTTGGGAGGAGTGGGAACATTAGGAGGATTATAAGAAGGAGA

AGGTGTTTTTGGAGGTGTAGGAACATAGGGAGGTTGGTTATGAGGGGAAGGAGCATTTGG

AGGACTATAAGCAGGAGATGGTGTTTTTGGAGGAGTAGGAACATTTGGTTGGTTATGAGG

GGAAGGAGCATTTGGAGGACTATAAGCAGGAGATGGTGTTTTTGGAGGAGTAGGAACATT

TGGTGGAGTGTAAATAGGTGAAGGTGTTTTGGGAGGAGTGGGAACATTAGGAGGATTATA

AGAAGGAGAAGGTGTTTTTGGAGGTGTAGGAACATAGGGTGGATTGTTACTAGGGGAAGG

AGCTTTTGGGGGAGTGTACACAGGAGAGGGTATTTTTGGAGGCGTTGGAAAATTAGGAGG

AGTATAAACTGGGGAAGGAGTTTGTGGAGGAGTTGGAATATATGGAGGATGATTAGCAGG

TGAAGGTGCTTGAGTGGGAACATTGGGAGGAGTGTATACAGGGGAAGGAGTTTTTGGAGG

TGTTGGAACATTAGGTGGATAATGAATAGGTGAAGGTGGTTTTGGATGAACATAGGGAGG

GCTGGAAATAGGTGAAGGTGTTTTAGGAGGAGGTATTGGAACATAGGGTGGATGATAAGG

AGTTTCTGGAGGAGTTGGAACAGTTGGAGTTTGAGGAGGTGCTGGAACATAAGGTGCTCT

CGGAGGAGTAATTGGAACATAAGGAGTGTTTGGAGGGGTTGGAACATAAGGTGGATTTGG

ATGATAAGGTGCTTTTGGAGGAGTAATTGGAACGTAAGGAGTGTTTGGAGGTGTCGGAGA

ATAATAAGGAGGTGGCTCTCTCACAGCCAACACCACAATAATCAATTTCTGACCCTTCTC

ACAATTATCATCTTTCCCACTAATGAAGTAAAATGGACCCGATCTATCAAACTGAAACTC

CGAGTCACCATCCTCAAACTTCTTGATCGGGTTCTTCTTGTTACATTTCTCATAATCATC

ATTGTTCACCACCAACACAGTATCTGAGTCCTTCTTGTACTTAAA

>**Gsoja_MSTRG.52475.5** GeneID=Gsoja_MSTRG.52475;Strand=-;Pos=Chr11:45344091-45344832,45345007-45345162,45345337-45346476,

AACAACAAAAGAATTTGATGATAATTATTACAACTCACATTGGTAACCATAGATACACAG

ATTATACGACATAAAATTAATTATGAGACGCGATCTCATTTGTCCAAGTAAAAATATTAG

AAAAAATATATAAAAGTGTTCACAATTACTATATACTATAACTCAAGTTCCAAAGAAAGT

TTGATTTTTCTAATCAATAGTGGTACTTAGAGCAGCACCGACCAGAACTATAACTCAAGT

TCCAAAAAAAAGTTTGATTTTTCTAACCAAGAGTGGTGCTTAGAGCAGCACCGACCAGAA

TGGTTAGTGAATACACTCCAAACCGCGACGGCGTTATAGACGACGCACCGTTTAGTCTGG

CTGGCGTCGTTTCGTTCGACGACGACGGCGTTTGAGATGATCCCGACGACGACGACGGCG

AGGGGGTTGTTGCCGCCGCAGACGGTAATGGCGGCGATGTTGCCGGGGTGAGGGAGGAAG

AGGGTGGGTAAGTGGGTGGTGCAGTTGAAGCAGAAGGTGGGTAAGATGGAGAGAGTGAGG

TTGAGGGAGGGATGGTGGATGGATAGGGCGATGATGGGTAGGGTGGTTGAGATGTTGGGG

AAGGAGTGTTTGGAGGTGTTGGATTAATGTAAGGTGGTTGAGATGTTGGGGAATGAGAGT

TTGGAGGTGCTGCAATGGAAGGAGGTTGGGAAGTGGGTGAAGGAGTGGTGTTTGGAGGTG

TGGGAATGTAAGGTGGTTGAGATGTTGGAGATGGGGTTATTGGAACAAAGGGTGGTTGAT

GGGCAGGGGAAGGCGTTTTTGGAGGGGTTGGAGTGTAGGGAGGTTGAGAAATAGGAGAAG

GAGTGTTTGAGGGAGTTGGGACATAAGGTTGGTTGCCAGGGGGAGGTGTTTTTGGAGTAG

TAGTGGGAACATTTGGAGGACTATAAATTGGAGAAGGTGTCTTTGGTGGTGTTGAAACAT

AGGGAGGTTGGTTATGAGGGGAAGGAGCATTTGGAGGACTATAAGCAGGAGATGGTGTTT

TTGGAGGAGTAGGAACATTTGGTGGAGTGTAAATAGGTGAAGGTGTTTTGGGAGGAGTGG

GAACATTAGGAGGATTATAAGAAGGAGAAGGTGTTTTTGGAGGTGTAGGAACATAGGGAG

GTTGGTTATGAGGGGAAGGAGCATTTGGAGGACTATAAGCAGGAGATGGTGTTTTTGGAG

GAGTAGGAACATTTGGTGGAGTGTAAATAGGTGAAGGTGTTTTGGGAGGAGTGGGAACAT

TAGGAGGATTATAAGAAGGAGAAGGTGTTTTTGGAGGTGTAGGAACATAGGGTGGATTGT

TACTAGGGGAAGGAGCTTTTGGGGGAGTGTACACAGGAGAGGGTATTTTTGGAGGCGTTG

GAAAATTAGGAGGAGTATAAACTGGGGAAGGAGTTTGTGGAGGAGTTGGAATATATGGAG

GATGATTAGCAGGTGAAGGTGCTTGAGTGGGAACATTGGGAGGAGTGTATACAGGGGAAG

GAGTTTTTGGAGGTGTTGGAACATTAGGTGGATAATGAATAGGTGAAGGTGGTTTTGGAT

GAACATAGGGAGGGCTGGAAATAGGTGAAGGTGTTTTAGGAGGAGGTATTGGAACATAGG

GTGGATGATAAGGAGTTTCTGGAGGAGTTGGAACAGTTGGAGTTTGAGGAGGTGCTGGAA

CATAAGGTGCTCTCGGAGGAGTAATTGGAACATAAGGAGTGTTTGGAGGGGTTGGAACAT

AAGGTGGATTTGGATGATAAGGTGCTTTTGGAGGAGTAATTGGAACGTAAGGAGTGTTTG

GAGGTGTCGGAGAATAATAAGGAGGTGGCTCTCTCACAGCCAACACCACAATAATCAATT

TCTGACCCTTCTCACAATTATCATCTTTCCCACTAATGAAGTAAAATGGACCCGATCTAT

CAAACTGAAACTCCGAGTCACCATCCTCAAACTTCTTGATCGGGTTCTTCTTGTTACATT

TCTCATAATCATCATTGTTCACCACCAACACAGTATCTGAGTCCTTCTTGTACTTAAA

>**Gsoja_MSTRG.79813.1** GeneID=Gsoja_MSTRG.79813;Strand=+;Pos=Chr18:23010953-23011524,23012138-23012817,

AGATAATTAACCAGAGCATGACAGCAGAGGCAAGAGAGAGACGAACAAAGCCCCAAAGAT

TGTGAAAGGCTTTGAAGGTAAAGCCAGACCAGGCCTCCCCGCAGGCCCCACCCATTATAT

AAACGAGCTGCGCAAGGTCGATGAACCACCACGACGCGTTCAGCACCACGGCAGCGCCGA

CGAGCCCCCACCGGAGCTTCAGCATGAGCAGCCAGCTGAACACGGTGTGCAGCACCAGCG

CCGCCGCCGCAATCCACGCCATCACCATGATCCTGCTCTGCGCCTGCAGGAACTTCTGCG

CCGGGTAGTTCACCGCGTACGCGAACAGCTGCGGGATCATCCACACCGCGAAATCCCCCG

CCGCCGCCGAAATCGCCTCCGTCTGCCCTATCGCCCGCAGCAGCGGCGCCGCGAAGATGT

AGAGGAGCGTCAGCAGGATCGCGGTGGCGTTCAGGATCACCCACGAGCGCTGCATGTACA

CGCCCAGCATGTGCACTTGCCCCGCTCCGTACGCTTGTCCACATAGCGTTTCCAACGCGC

TTCCCATTCCAAACTGCGCGCAAAGTCCACAATGATGCCAAGGCAGAAGCCGGCGATGAC

GGAGTTTTCAATGGAGATGGCGGCGAGGGCGAGGGTGCTGACGTGGCCGGAGAAGACTTG

GGTGACGGCGCCGAGGGAGTACTGGCAGACGGAGGTGAAGATGGCGGGGCCGGCGAGGTA

CCAGAGCTTCTTCGACTCGGCGAAGAACTCTCTGGCGAAGTCGCCGGCGCCGGCGATCGG

AGCGATGTCATCGGATTTCGCCGTGAAGACGACGGCGGGGTCCGGTGTGTGGTGTCGCGG

CGTGAGAAGTGGGTGAGTTTGGTCCTCATGGTCCTCCATCGTGAATTCGTGATATGAATC

AGAGATACGAAAAAAATTGAAAATTCTAGAACAACTTTATAAAGAAAAAGTTCCGGCCTTC

AGAGCAGGTTTTTACTGTTACCCAATGTAATGCCACACTCCGTCATCGGTTCGGAAAAGG

AGATAAAAGTAAAAAGGATAGGGAGAAAAATCAATCATAATGTCACGGAAAGTAGATAAA

AAAACATAGTGACAAAATAAAATTATTTTAAAGTTTAGGGTTATAACTTTTTTTCCAGAG

TTTAGGGTTATAGTTGAAACGCCATAGTTGCTATAAAATATTTTATTCGCAATTGTCCGG

TTCTCATGTAAGAAAAAGTATTGTTCATGTCATTTAAAAGATAGTTTTAGCC

>**Gsoja_MSTRG.52475.15** GeneID=Gsoja_MSTRG.52475;Strand=-;Pos=Chr11:45344112-45344898,45345073-45345162,45345337-45346411,

ACGACATAAAATTAATTATGAGACGCGATCTCATTTGTCCAAGTAAAAATATTAGAAAAA

ATATATAAAAGTGTTCACAATTACTATATACTATAACTCAAGTTCCAAAGAAAGTTTGAT

TTTTCTAATCAATAGTGGTACTTAGAGCAGCACCGACCAGAACTATAACTCAAGTTCCAA

AAAAAAGTTTGATTTTTCTAACCAAGAGTGGTGCTTAGAGCAGCACCGACCAGAATGGTT

AGTGAATACACTCCAAACCGCGACGGCGTTATAGACGACGCACCGTTTAGTCTGGCTGGC

GTCGTTTCGTTCGACGACGACGGCGTTTGAGATGATCCCGACGACGACGACGGCGAGGGG

GTTGTTGCCGCCGCAGACGGTAATGGCGGCGATGTTGCCGGGGTGAGGGAGGAAGAGGGT

GGGTAAGTGGGTGGTGCAGTTGAAGCAGAAGGTGGGTAAGATGGAGAGAGTGAGGTTGAG

GGAGGGATGGTGGATGGATAGGGCGATGATGGGTAGGGTGGTTGAGATGTTGGGGAAGGA

GTGTTTGGAGGTGTTGGATTAATGTAAGGTGGTTGAGATGTTGGGGAATGAGAGTTTGGA

GGTGCTGCAATGGAAGGAGGTTGGGAAGTGGGTGAAGGAGTGGTGTTTGGAGGTGTGGGA

ATGTAAGGTGGTTGAGATGTTGGAGATGGGGTTATTGGAACAAAGGGTGGTTGATGGGCA

GGGGAAGGCGTTTTTGGAGGGGTTGGAGTGTAGGGAGGTTGAGAAATAGGAGAAGGAGTG

TTTGAGGGAGTTGGGACATAAGGTTGGTTGCCAGGGGGAGGTGTTTTTGGAGTAGTAGTG

GGAACATTTGGAGGACTATAAATTGGAGAAGGTGTCTTTGGTGGTGTTGAAACATAGGGA

GGTTGGTTATGAGGGGAAGGAGCATTTGGAGGACTATAAGCAGGAGATGGTGTTTTTGGA

GGAGTAGGAACATTTGGTGGAGTGTAAATAGGTGAAGGTGTTTTGGGAGGAGTGGGAACA

TTAGGAGGATTATAAGAAGGAGAAGGTGTTTTTGGAGGTGTAGGAACATAGGGAGGTTGG

TTATGAGGGGAAGGAGCATTTGGAGGACTATAAGCAGGAGATGGTGTTTTTGGAGGAGTA

GGAACATTTGGTGGAGTGTAAATAGGTGAAGGTGTTTTGGGAGGAGTGGGAACATTAGGA

GGATTATAAGAAGGAGAAGGTGTTTTTGGAGGTGTAGGAACATAGGGTGGATTGTTACTA

GGGGAAGGAGCTTTTGGGGGAGTGTACACAGGAGAGGGTATTTTTGGAGGCGTTGGAAAA

TTAGGAGGAGTATAAACTGGGGAAGGAGTTTGTGGAGGAGTTGGAATATATGGAGGATGA

TTAGCAGGTGAAGGTGCTTGAGTGGGAACATTGGGAGGAGTGTATACAGGGGAAGGAGTT

TTTGGAGGTGTTGGAACATTAGGTGGATAATGAATAGGTGAAGGTGGTTTTGGATGAACA

TAGGGAGGGCTGGAAATAGGTGAAGGTGTTTTAGGAGGAGGTATTGGAACATAGGGTGGA

TGATAAGGAGTTTCTGGAGGAGTTGGAACAGTTGGAGTTTGAGGAGGTGCTGGAACATAA

GGTGCTCTCGGAGGAGTAATTGGAACATAAGGAGTGTTTGGAGGGGTTGGAACATAAGGT

GGATTTGGATGATAAGGTGCTTTTGGAGGAGTAATTGGAACGTAAGGAGTGTTTGGAGGT

GTCGGAGAATAATAAGGAGGTGGCTCTCTCACAGCCAACACCACAATAATCAATTTCTGA

CCCTTCTCACAATTATCATCTTTCCCACTAATGAAGTAAAATGGACCCGATCTATCAAAC

TGAAACTCCGAGTCACCATCCTCAAACTTCTTGATCGGGTTCTTCTTGTTACATTTCTCA

TAATCATCATTGTTCACCACCAACACAGTATC

>**Gsoja_MSTRG.40295.1** GeneID=Gsoja_MSTRG.40295;Strand=+;Pos=Chr09:38832874-38833374,

TGAAATCGTAACGCGACCACGACTGATATTTAAAACCTTGGTTCCCACCCATCCATCAGT

TTAAACTTATGAAGGAAAAGAGAACTAACATACCAATAAACTGAAACCTGTGACAGAGGC

AAAAGAAGTGGCCATGGAAGCACCAGAGAGAGACAACTCGCCAAGATGGCCAACAAACAT

GACAGATATGATACTGAGGCAAAAATTCAAAAGGGTCACTGATATCAAAGGCCCTGCAAG

CCATAACTGCTTCTTCACTTCTTCTATAACTTCCCTTCTCTCAATACCCCTCCCTGATGA

TGATGAGTGTGAGTGCTTAATCAATGGAGACTGAAGAGATGCACTTTGGTTTTGGTCTCC

TCTCTCCATTTCTCTTCTTTGTTGCTCTTTAAGTTCTTAACACATGGAAGACTCAGACAG

AGAAAAATGTTGCATATTGCATGGTGTTGTTAAAAAGATTATAAAAACATCTGAAAAACT

CTAGATAAAGGCGGAACTGGA

>**Gsoja_MSTRG.52475.9** GeneID=Gsoja_MSTRG.52475;Strand=-;Pos=Chr11:45344091-45345072,45345421-45346429,

CATAGATACACAGATTATACGACATAAAATTAATTATGAGACGCGATCTCATTTGTCCAA

GTAAAAATATTAGAAAAAATATATAAAAGTGTTCACAATTACTATATACTATAACTCAAG

TTCCAAAGAAAGTTTGATTTTTCTAATCAATAGTGGTACTTAGAGCAGCACCGACCAGAA

CTATAACTCAAGTTCCAAAAAAAAAGTTTGATTTTTCTAACCAAGAGTGGTGCTTAGAGCA

GCACCGACCAGAATGGTTAGTGAATACACTCCAAACCGCGACGGCGTTATAGACGACGCA

CCGTTTAGTCTGGCTGGCGTCGTTTCGTTCGACGACGACGGCGTTTGAGATGATCCCGAC

GACGACGACGGCGAGGGGGTTGTTGCCGCCGCAGACGGTAATGGCGGCGATGTTGCCGGG

GTGAGGGAGGAAGAGGGTGGGTAAGTGGGTGGTGCAGTTGAAGCAGAAGGTGGGTAAGAT

GGAGAGAGTGAGGTTGAGGGAGGGATGGTGGATGGATAGGGCGATGATGGGTAGGGTGGT

TGAGATGTTGGGGAAGGAGTGTTTGGAGGTGTTGGATTAATGTAAGGTGGTTGAGATGTT

GGGGAATGAGAGTTTGGAGGTGCTGCAATGGAAGGAGGTTGGGAAGTGGGTGAAGGAGTG

GTGTTTGGAGGTGTGGGAATGTAAGGTGGTTGAGATGTTGGAGATGGGGTTATTGGAACA

AAGGGTGGTTGATGGGCAGGGGAAGGCGTTTTTGGAGGGGTTGGAGTGTAGGGAGGTTGA

GAAATAGGAGAAGGAGTGTTTGAGGGAGTTGGGACATAAGGTTGGTTGCCAGGGGGAGGT

GTTTTTGGAGTAGTAGTGGGAACATTTGGAGGACTATAAATTGGAGAAGGTGTCTTTGGT

GGTGTTGAAACATAGGGAGGTTGGTTATGAGGGGAAGGAGCATTTGGAGGACTATAAGCA

GGAGATGGTGTTTTTGGAGGAGTAGGAACATTTGGTGGAGTGTAAATAGGTGAAGGTGTT

TTGGGAGGAGTGGGAACATTAGGAGGATTATAAGAAGGAGAAGGTGTTTTTGGAGGTGTA

GGAACATAGGGAGGTTGGTTATGAGGGGAAGGAGCATTTGGAGGACTATAAGCAGGAGAT

GGTGTTTTTGGAGGAGTAGGAACATTTGGTGGAGTGTAAATAGGTGAAGGTGTTTTGGGA

GGAGTGGGAACATTAGGAGGATTATAAGAAGGAGAAGGTGTTTTTGGAGGTGTAGGAACA

TAGGGTGGATTGTTACTAGGGGAAGGAGCTTTTGGGGGAGTGTACACAGGAGAGGGTATT

TTTGGAGGCGTTGGAAAATTAGGAGGAGTATAAACTGGGGAAGGAGTTTGTGGAGGAGTT

GGAATATATGGAGGATGATTAGCAGGTGAAGGTGCTTGAGTGGGAACATTGGGAGGAGTG

TATACAGGGGAAGGAGTTTTTGGAGGTGTTGGAACATTAGGTGGATAATGAATAGGTGAA

GGTGGTTTTGGATGAACATAGGGAGGGCTGGAAATAGGTGAAGGTGTTTTAGGAGGAGGT

ATTGGAACATAGGGTGGATGATAAGGAGTTTCTGGAGGAGTTGGAACAGTTGGAGTTTGA

GGAGGTGCTGGAACATAAGGTGCTCTCGGAGGAGTAATTGGAACATAAGGAGTGTTTGGA

GGGGTTGGAACATAAGGTGGATTTGGATGATAAGGTGCTTTTGGAGGAGTAATTGGAACG

TAAGGAGTGTTTGGAGGTGTCGGAGAATAATAAGGAGGTGGCTCTCTCACAGCCAACACC

ACAATAATCAATTTCTGACCCTTCTCACAATTATCATCTTTCCCACTAATGAAGTAAAAT

GGACCCGATCTATCAAACTGAAACTCCGAGTCACCATCCTCAAACTTCTTGATCGGGTTC

TTCTTGTTACATTTCTCATAATCATCATTGTTCACCACCAACACAGTATCTGAGTCCTTC

TTGTACTTAAA

>**Gsoja_MSTRG.40292.1** GeneID=Gsoja_MSTRG.40292;Strand=+;Pos=Chr09:38829349-38829867,

AGAGGTTAAGATAGAATATGAAATAAGAAAATATTCCATTAGTGCATCAAATGACTTATT

ACAACAAGATAAAATAATTGTACTACTCTATTCTATCCGAAACTTAGTTTACAGTCACAA

AATTACCCCTAATATAACCAACCATAGACTCACCAGTGGGGCAATGAGAAAAATTGAAAG

GTTTTCAATAACTAGATCAAAATCACATTTAGTATCTTCTACACAAGAACATAATCTTTT

CCCTCCAAAAAACATCAAGACGTCCCCTACCAACACATACATAAAAATAAAATAATAAAA
CCACTTTATTCACTTCCAATGGCCAACTGCATCATCTATCATCTTTCGCGCATATATAAT
TGCCCCTAGACTTTTCTGCCAAAAGAAGGATACCAAAACATGTATTGTTTTCATCCAAAT
AGAATTTAACTCAGAATGTGCAACTGCAACGATATTGCATCTTCATGAAACTAAGCTCTC
AAGTGTTACTGAATTATAGACTCTATCTGTTGCTTTCTT

>**Gsoja_MSTRG.65597.1** GeneID=Gsoja_MSTRG.65597;Strand=-;Pos=Chr15:2792958-2793198,
GCCCAGCAACAGCCAGACCATCCGCAAGAAGAGACACTGCCAACCAAACCTGCAGACATA
CTTGAAATGCAGCCATAGATGTTGGCCCCTGCCGTGCAGCTAGTGATGCAGCCAGTGTCA
CACAGAATGTTACAGCGATGACTCTCATTAATAATAGAAAACCTGGATGACAACAACAAT
ATAGGGAAGGACAAAGATGGTGTCAAGTCTCAATACTAAAGTGTTAGTAAACCTAAACAG
A

>**Gsoja_MSTRG.15162.1** GeneID=Gsoja_MSTRG.15162;Strand=-;Pos=Chr04:8777225-8778120,
GAAAGTTTGAAGAATTCCCAAAGGCCAACAAAAGCTTCAACAGAAAAACCAGTCCACGAG
CGAGGGCAACCACCAAAGAGTGTATACCCGAGCATTCCCAAAACAGAGAGCCACCACGAG
AAACCAATGGAAAGAGCAGTGCCAACAATCCCAATTCTCATCCTATAAACAAAAACCCAA
CTCACTAACACGTGAACCGCAAGAGCCACCCCTGAAACCCAAGCAATGATGCCGGTCTTC
AACTGGCACTGCAGGAACCGCTGCAAAGTGAACTGGAACGGGAAGCTCAGGTGCAATGGG
ATCAACCAAACCGCCACCAGCCCCGCCTGCTCCGCCACCGCGACGGGCTGCCCTATTAGC
TTCAACACCGGCGTCGCAAAAATGAACACCGGCAACAACAGAATGGAAGACAGGAACAGA
ACAACCCACGAGCGTTGCAAATAAACGCCCAATATGCGCTGTTGTCCGGCGCCGTACGCC
TGCCCGCATAGTGTCTCAAGCGCACTCGCCATACCCAACTAAAAAAGGAATAAGTTCGTT
AACAAAAATTAACTAACTAACGACTAACAACAATAATGCGGTGAATGAAAAGTGGGAAAT
AGTGAATGAAAAGTGGTAAGTGGAAAGTGTTACCAAGAAACCGAAGGTGATGGAGATGAG
GACGGTGCAGGCGATGGAAATGGCGGCGAGGTCGAGATCGCCGAGGTGGCCGGCTAAGGA
CTGGGTGACGACGGTGATGGAGAACATGGCGAGGCGGGTGAAGATGGAGGGGGCAGCGAT
GTGCCATAGCTTCTTGGATTCGGAACATGATCGTTGGAAGAGAGAGTGATTGTTGGATCC
GTGTGGTTGTGGTGGCAGTGTTGACTTTATCAGAGGATGCTCATGCTCTTCTTCAT

>**Gsoja_MSTRG.52475.28** GeneID=Gsoja_MSTRG.52475;Strand=-;Pos=Chr11:45344746-45344898,45345247-45345354,45345529-45345654,45345862-45346016,

TGCCGGGGTGAGGGAGGAAGAGGGTGGGTAAGTGGGTGGTGCAGTTGAAGCAGAAGGTGG

GTAAGATGGAGAGAGTGAGGTTGAGGGAGGGATGGTGGATGGATAGGGCGATGATGGGTA

GGGTGGTTGAGATGTTGGGGAAGGAGTGTTTGGAGGTTGAGAAATAGGAGAAGGAGTGTT

TGAGGGAGTTGGGACATAAGGTTGGTTGCCAGGGGGAGGTGTTTTTGGAGTAGTAGTGGG

AACATTTGGAGGACTATAAATTGGAGAAGGTGTCTTTGGTGGTGTAGGAACATAGGGAGG

TTGGTTATGAGGGGAAGGAGCATTTGGAGGACTATAAGCAGGAGATGGTGTTTTTGGAGG

AGTAGGAACATTTGGTGGAGTGTAAATAGGTGAAGGTGTTTTGGGAGGAGTGGGAACATT

AGGAGGATTATAAGAAGGAGAAGGTGTTTTTGGAGGTGTAGGAACATAGGGTGGATTGTT

ACTAGGGGAAGGAGCTTTTGGGGGAGTGTACACAGGAGAGGGTATTTTTGGAGGCGTTGG

AA

>**Gsoja_MSTRG.52475.29** GeneID=Gsoja_MSTRG.52475;Strand=-;Pos=Chr11:45344746-45344832,45345007-45345102,45345355-45345366,45345463-45345654,45345862-45346016,

TGCCGGGGTGAGGGAGGAAGAGGGTGGGTAAGTGGGTGGTGCAGTTGAAGCAGAAGGTGG

GTAAGATGGAGAGAGTGAGGTTGAGGGAGGGATGGTGGATGGATAGGGCGATGATGGGTA

GGGTGGTTGAGATGTTGGGGAAGGAGTGTTTGGAGGTTGAGAAATAGGAGAAGGAGTGTT

TGAGGGAGTTGGGACATAAGGTTGGTTGCCAGGGGGAGGTGTTTTTGGAGTAGTAGTGGG

AACATTTGGAGGACTATAAATTGGAGAAGGTGTCTTTGGTGGTGTTGAAACATAGGGAGG

TTGGTTATGAGGGGAAGGAGCATTTGGAGGACTATAAGCAGGAGATGGTGTTTTTGGAGG

AGTAGGAACATTTGGTGGAGTGTAAATAGGTGAAGGTGTTTTGGGAGGAGTGGGAACATT

AGGAGGATTATAAGAAGGAGAAGGTGTTTTTGGAGGTGTAGGAACATAGGGTGGATTGTT

ACTAGGGGAAGGAGCTTTTGGGGGAGTGTACACAGGAGAGGGTATTTTTGGAGGCGTTGG

AA

>**Gsoja_MSTRG.57214.2** GeneID=Gsoja_MSTRG.57214;Strand=-;Pos=Chr12:39967237-39967640,39967716-39968346,

GAATCAAGAGCCAACTCAGGATTAGGGAGAAGCCCCGCGAGCAAAACCAGAATCTGAAAG

TACCATGTCTCCAGGCACAGCATGACCGCGGACGCAGCGGAGAGCTTGAAGAACCCATAC

AAACCGCTAAACGCCTCCCACGTGAACCCCTGCCACGTGCGCCTGCACCTCTCGCTCTTC

ACTATGTACACGTACTGTCCAATCACCATTATCCACCACGACAGGCTCAGCACCAGCGAC

GCCCCCAACAGCCCCAACCCAATCTCGTACACCGCCACCCAACTCATCCCCAGGTGGACC

ACCAGCGTCGCCGCTGATATGTACGCGCTCGGTGCCACTATGCTCTGCGCTTGGAGAAAC

TTCTGGATCGGGAAGTTCGCCGCGTATGCGAATATCTGTGGGATCAACCCGTACACAAAG

AGTGCTGCCGCCGACGCGATTCTCGGTGATTCCCCGAGGAATATTAGCATCGGTTCGCTG

AATACGTATATCACGGTCAACACTACGCCGGCTAGTGAGAGAAGAATCGTTGACCGCTGC

ATGTAGACTCCTAACATCCCGTACTTTTGAGCTCCGAATGCTTGTCCGCATAGCGTCTCC

ACGGCACTCCCCATACCCAACTGCAAGTAATCATGAGGCCGTAGGCGAACATTTGGATGC

CGGTGTTGCCGAGGGAGGCGGCGGCGAGTTCGAGGTTGCCGAGGTGGCCGGAGAAGATTT

GGGTGGACATGGACATGAGGTAGTTGATGAGGTAGACGATGACGGCAGGGGCGGCGAGGA

AGAAGAGGAGCTTCAACTCGATCCACGTGGCGGGTCCCACACGGTTGAGGAAGGGTAGGG

TGTCGTCGGAGAGAATTCTCTCAAGTTGCCCATGGGAGGAGGACTCGTGCTTTGAGTTTG

ATCGAGTTCCCTCCGCTGTTAACAGTGGCTCTTTCTCCGCTGAACCCATTGATCTCGATC

CGAATTCACGGCAAAGAAGGCTTTCTCTCCTGTAACGTTTGGAGCAGCAGCAATCTCGCC

GCCACAAGTCCCAAC

>**Gsoja_MSTRG.52475.19** GeneID=Gsoja_MSTRG.52475;Strand=-;Pos=Chr11:45344518-45344706,45344755-45344898,45345073-45345162,45345511-45345792,45345841-45345929,

AGGGATGGTGGATGGATAGGGCGATGATGGGTAGGGTGGTTGAGATGTTGGGGAAGGAGT

GTTTGGAGGTGTTGGATTAATGTAAGGTGGTTGGGAAGTGGGTGAAGGAGTGGTGTTTGG

AGGTGTGGGAATGTAAGGTGGTTGAGATGTTGGAGATGGGGTTATTGGAACAAAGGGTGG

TTGATGGGCAGGGGAAGGCGTTTTTGGAGGGGTTGGAGTGTAGGGAGGTTGAGAAATAGG

AGAAGGAGTGTTTGAGGGAGTTGGGACATAAGGTTGGTTGCCAGGGGGAGGTGTTTTTGG

AGTAGTAGTGGGAACATTTGGAGGACTATAAATTGGAGAAGGTGTCTTTGGTGGTGTTGA

AACATAGGGAGGTTGGTTATGAGGGGAAGGAGCATTTGGAGGACTATAAGCAGGAGATGG

TGTTTTTGGAGGAGTAGGAACATTTGGTGGAGTGTAAATAGGTGAAGGTGTTTTGGGAGG

AGTGGGAACATTAGGAGGATTATAAGAAGGAGAAGGTGTTTTTGGAGGTGTAGGAACATA

GGGTGGATTGTTACTAGGGGAAGGAGCTTTTGGGGGAGTGTACACAGGAGAGGGTATTTT

TGGAGGAGTTGGAATATATGGAGGATGATTAGCAGGTGAAGGTGCTTGAGTGGGAACATT

GGGAGGAGTGTATACAGGGGAAGGAGTTTTTGGAGGTGTTGGAACATTAGGTGGATAATG

AATAGGTGAAGGTGGTTTTGGATGAACATAGGGAGGGCTGGAAATAGGTGAAGGTGTTTT

AGGAGGAGGTATTG

>**Gsoja_MSTRG.5529.1** GeneID=Gsoja_MSTRG.5529;Strand=+;Pos=Chr02:7817334-7817573,

ACACAAAACTCCTAGAAAGCCATCCACCATGAAAGAGAGACAAAGCACAGGAACTATCTT

TGCAACATAATGTACAACTTCCATCTCATTGCTAAATGCAAATCCCAACACATGACGGAA

GCAAAAGAGAACAGAGCTAAAGACAACTGCATCCGTGAATGCTAGAACAATTACAGCAAA

AACAGCCTCTCGCGCTGCCTGTGGTCTTCGTGCCCCTAATTCATTCGAAACCCGGGTACT

>Gsoja_MSTRG.28717.1 GeneID=Gsoja_MSTRG.28717;Strand=-;Pos=Chr07:9578514-9578773,

GGTTTGTAGGAACTTAGTAATGCACCGAAGAAGACCATTGGCCGAAAGGCTTGGGATCAA

GTATCTGGCATATAGTTGAGCATGTGCAGCAATTTCTTTGTCTTGATGGAGAGCAACTAG

AATAGGCCCTAAGAATACCCAAATAATGGAGAGGGGTATGGTAACAAGGGTGAGAACCAA

CATGGCACCTTGGGTGTGTACACCAAGCATGTGAAACTGCTTTGCTCCATATGCTTGACC

ACAAAATGTGTCTAGTGCGC

>**Gsoja_MSTRG.29212.1** GeneID=Gsoja_MSTRG.29212;Strand=-;Pos=Chr07:14875254-14877261,

AATTAATTCCCTTTAATAAATATAAGAATTATTACAAAAAGAAAAGAATATTACACCGTC

AATCAACAATCATCATAATTTTTTTTTTTTTTTTATCAAGAGTTGTTGTTGTCCACAGCG

GCAAGCAACGGTTGTTTGTGGCCGTCGGATCCTTGATCAAGGGCCGTGAGTAACTGGGCC

CGGCAGGCCTGGTACTCCCAATCGGTGGTCCCAATCATATACAACATTAGGCCCGCGCAA

CAAACCTGGGCCGACAACAGGCCCAGCCAGAGCCCACAGAACCCAACCTCGAGCCAGAAC

GCAAGCCCAACCGCCACCGGCATCCCCACCAGATAAAACGCTCCCAGGTTCACGTTCGCC

GCCACGTTCGGCCGCGCCGTCCCCCTCACCACCCCGCATCCCACCGTCTGCGGGCAGTTT

CCGAGCTCGCACAACCCCAGAATCGGCAGCGCCGCTGCCGTCAGCCGCAGAATCCCCTCG

TCCCCGGTGAACATCCTCCCCCACCTCCTCCGCATCGCCGTCGCAAAAACCACCGCGGAG

AAGCCCATCACTGCGGCGAAGAACACCGCTACCACCGCCGACATCCTGGCGCGTGGACCC

CTGTTCGCGCCAAGCTGGTTCCCCACGCGCGTGGAAACCGCAAATCCCAGAGACGAGGGG

AAGACATAGATCAACGACGTCGTCTGGATTAAAATCCCCATAGCCGCCACGCTGGCAGTG

GGGTCCACCAAAACCCCACACAACAAAATCATAATCTCATACCACCACCATTCCAAACAA

ACCGAGACGCAGCTCGGCGCCGCAAGCCTAAGCAGCGGCTCCCAGCCGCCGAAACACTCC

CGGCTCGGCGCGGTCCACGTGGCCAGGTGGACCCCACTTATCCACACGTAGAGGACCAGC

AGACTTAGAATGGAAAAACTGGAGGCGGCGGAGGCGGCGGCGACGCCGGGCAGGCCGCGC

TGGACGAGGAGCAAGTTGAATGGAACGTGGAGGAGAGTGCCGGCGAGGGACGCTAACGTT

ACCGGGTGGGTGATGTTCTGGGCGCGAAGGTAGACTCTGATTGGGTGGAGGAAGGAGTTG

GTGACGAGGTCGGGGAGAAGGAAAACTAAATAGGTTTGTGCCATTTGGGTGATGTGTGTG

TGTTGGTGGAGTAGGATAAAGACTTTAGACATGTTAAGCCACAAGAGCGAAATGGGGATT

GAAGAGAATAATAAGAAGATGACGCAGCGTTGGAGGGTGAGAGAGAGGAGTTTAGGACGC

TTAGCTCCGAAGGCTTGGGAACAAAGAGGTTCCATTCCTAAGGAGAGACCAGAGAGAACG

GAGTAACCGGTGATGTTGGCGAAGGCGATGGCTAAGGAACCTGCGGCCAGTTCGGTGTCT

CCTAGGTGGCCGAGGAACAGCATGGAGACCATGGAACGCGCGTAGAAAATGAGACCAGTG

AGTGCTGTCGGGAATGCCAACTCCCACAGGGATTTCGCTTCTCCTATCACATCCCACATT

ATTGTCGAACGTCTTGTTAACACACCACCTAATTCTTCGGTCTGCTTTTCCTTATCATCA

TGCCTCTGCTCTGATTTTGATTCGCACATGTTCTTGGTTCTTCTATTTTATGACGACGAC

TACTACTACTACTACTACACGTCTTCCTTCGTTGTTATGAAATGAAGACTCAAAAGAGGG

TATATATGTACGTGTTTATGAATGATTTTGAATAATATGATGAAGGAAGAGTGCGTGTTG

TGAAAACTGCGATACACATACACTAGACAGTGTGTTCTGAAACAAAGAGGAGGAGGAGGA

GGAGGAGGAGAGGGGACTCTGTTTTTTTTTAAAGGGTGGAAAGCGGTGGGAGCTAGGAGC

AGAGTGATAAATGAAGAAAGAAACTAATTACTGCGTGGGAGTGAAGAAGTGAGAAGGATG

GGCGAGTATAAATAGAATTGAGGAGGATTTGAATTCGAGCGTGTTTTTCCTGTTTAAAAT

ACTATGTGTAAGTGACCCTTCTTCCTTT

>**Gsoja_MSTRG.28712.1** GeneID=Gsoja_MSTRG.28712;Strand=-;Pos=Chr07:9566342-9566887,

CAGACCATGAGTGCTGAAGGAAAAGCAAGACTGAGAAATTGAGGAATGTTCTGCAAGGAT

TCCCTTGAGAAACCAGTCCAAGTGCTTTTGCAGGAAGAGGAGAACCTGATGTAAAGTGCT

AGTAGCACCGTGTTAAGCCAATTTGAAATGCAAAATGCAATGGCAGCTCCTTTGATGCCA

AGCCCAAATTTTAGAACCAAAAGCCAACAGAGAAGGGCATGTGCTAAGGTAGTGAATCCA

GAAGCTAGCATCATTGGAAGGACAATGTTCTGGGTTTGTAGGAACTTAGTAATGCACCGA

AGAAGAGCGTTGGCTGAAAGGCTTGGGATCAAGTATGTGGCATATAGTTGAGCTTGTGCT

GCAATTGTTTTGTCTTGATGCAGAACAACCAGAATAGGCCTCAAGTATGCCCAAATAAAA

GACATTGGTATAGTAGCAAGCATGATGATCACTATGGCTCTCTGCATGTGTATGCCAACC

ATATGATACTGCTGTGCTCCATATGATTGACCACAAAATGTGTCAAGTGCACTTGACATA

CCTAGC

>**Gsoja_MSTRG.65595.1** GeneID=Gsoja_MSTRG.65595;Strand=-;Pos=Chr15:2789754-2790594,

GTTCAGCCTTTCAAGTTAGAACTTGCATTTTTCAGAAAGATATGTCCTACAGAAAGGCTA

TAACATGATATATGCATATCATACCTGGCATCCTTAAAGAAAGTGCAAATTGGTATTCTC

CTCCAATCACCTAATGAATACATACTCTCTTTCTCAGCCATATTTGAGAAGCCAAATTCA

CTGTGTTGTTTGAGAAACCAAACGAGCAAAGGCAACTTATTGAGGCTAGTGATACATAAC

TGAAGAGATACTGAAGGAGCTCCAATATTTGGCATGCGTTAAACTTTTAACTTGAGCATT

AACAGTTAAAGACATCATAATAATTTCGGCCCCAAAGTAGAGAGACTGCATAATACTATC

TCGGTATTTATGTGGTTAAACACAGACCAACCAATCAAGTCAAATCATAATGGAATAACA

TCATGGAATAGGGATAGACTCCACAGTGATGAAAAAGTTGGTAAAGGAGATGCCCTTTAA

TATTCTTCCCCTCTTGCATAGAATAGTCCTTCTCACTATGTATGAGCAAAACCACTAACT

ATAATAATAAAGAATGAAAAAAAGTACTTGATTAACACACACAAAGAGGGTTTACCCCCA

TGAGAAAACACTGTAAAGTGAGTTCCCCTTAATTCAAACTTGGACATTTGGGATTTGAAA

GAGAGTCAAACCAACCCATCACAAGTAAGACAAATAAACTGCAATTCAGGCTTTGAAACT

CCACAGTCAGGCTTTAATACTAGCTTTGAAATAAAACACCCTTAACTTGGGGGCGAAAAA

CAACCATAGCATGATAATGGAACTCTGAGAACAGAACAAGAGACGTTTGAGTTGAGGTAG

G

>**Gsoja_MSTRG.52475.2** GeneID=Gsoja_MSTRG.52475;Strand=-;Pos=Chr11:45317721-45317734,45344717-45344844,45344941-45345072,45345247-45345336,45345511-45345652,

TGAGAAATAGGAGAAGGAGTGTTTGAGGGAGTTGGGACATAAGGTTGGTTGCCAGGGGGA

GGTGTTTTTGGAGTAGTAGTGGGAACATTTGGAGGACTATAAATTGGAGAAGGTGTCTTT

GGTGGTGTTGAAACATAGGGAGGTTGGTTATGAGGGGAAGGAGCATTTGGAGGACTATAA

GCAGGAGATGGTGTTTTTGGAGGAGTAGGAACATTTGGTGGAGTGTAAATAGGTGAAGGT

GTTTTGGGAGGAGTGGGAACATTAGGAGGATTATAAGAAGGAGAAGGTGTTTTTGGAGGT

GTAGGAACATAGGGAGGTTGGTTATGAGGGGAAGGAGCATTTGGAGGACTATAAGCAGGA

GATGGTGTTTTTGGAGGTGTAGGAACATAGGGTGGATTGTTACTAGGGGAAGGAGCTTTT

GGGGGAGTGTACACAGGAGAGGGTATTTTTGGAGGCGTTGGAAAATTAGGAGGAGTATAA

ACTGGGGAAGGAGTTCTTGGAGGTGT

>**Gsoja_MSTRG.52478.1** GeneID=Gsoja_MSTRG.52478;Strand=-;Pos=Chr11:45322732-45323210,

CGAGAAAATAATCTATCATCAATCATATGGTTCTCCATTGTCAATTACACGTAGTTTCCT

AGTTTTTTTATAGTATTCTTTTCACGGTGGGATACATTACTCCCCATCTATTCTTGATGT

GTTCCCGTGAGACTGTTACATTATATGCCAAGATTTACTCTCTGCAACTCACCAACACTC

CCAAATAAAATATAAAAAAAAGTGTGAGCACTTTCTCAAAATGAATATAAGCAAGAAACG

CTTCACCAAAATCATGTTCAGTGTTCACCCTATCTGATAAACTTCTGCATAATAGAATGC

AAACCTTTTTCTTTTTTTTTTACCATAAGTCCAATTCCATTCAGTTAAACAACAAGAGGC

CGTTCTTTATCATTGTCCCTAGAGAGATCCAATTTAGTCCATTTGGCACGCCTTATGAAA

AGGAAGAGAGTCCCACTTTGACAAAGTAACCCACAAATCAGACCAATCCATAAACCCTA

>Gsoja_MSTRG.5532.1 GeneID=Gsoja_MSTRG.5532;Strand=+;Pos=Chr02:7841861-7842099,

GGAGAGAATAATTAACCTGAAAGAACTCCTTGTAAGCTGTCAGTAAAAATAGAGAGACAG

AGCAGAGGGATCATAACAGCCACATAATGAACAACCATACGGTCATCACTATAGGCATAA

CCCAAGATATGTCTGCAGCCGAATAGTGTTGCACTTACAATGAAACCCTCTGTGACTGCA

AGGAACATCGTTGCAGAAACAGCCACATGAACTGCTTGTGGATTCCCAGCTCCTAATTC

>Gsoja_MSTRG.52477.1 GeneID=Gsoja_MSTRG.52477;Strand=-;Pos=Chr11:45320962-45321906,

TAGAATTTAGAACCTTACCATATTGCAATCAACGAAGTGGTTATCTGTGAGTCGGGCATT

AGTCCAGCTAAGAAAACCAAAACTTCAAAAGCCCAATACTCCAAACTTGCATAACATAAA

CATCAGGTAATAAGATGATATATGAATCTGGACGAACCATGGTTTTTACCCTTGTATTGA

GGTGTTTTTCACTTTAAACAAATCGTGTCTCTGATTTTTCTTAATTTCTATTTTGCGCTC

TATAATTTATTGGTGCTCCTAGGTTTTTGCAAATTTGGGAGAATTTATTTTCACTGTCTA

TTACTCTATTATTGAGTTTGTTATTGTGTTGGCTTGTTTTTCCAATCAAGATGAACAATC

AAAAGGGCTAGCTAAGTGAAAGGTTATTAGTAATTCATACCATACCATTGCAGCAGAGGG

CAGAGCTAGTCTCATGTTTGTAAATACGTAACGAAATGAATGCGTTGAAAATCCTTTCCA

TGTCTGCTTGAACTTCTTAGCATACATTACATACAAGGCCAATAATAGCAATGATATCCA

TAGTGAAATAGAAGCAGCAACTGGTGCACCTGTAAAACTCAGACCTGACCACTGAACCAA

GCCATATGCAACACCAATATGAACCAGCATTGGAAGAGCAGAGAGTACAACCAATGGAAT

TACTACAGATTGTGTCTGGAGAAACCTCAAGATGTTTTGCAAGAAGCTATAAGCAAACAC

TCCCGGGATAAGAAACTTCATATAAAGTGCAGCTGTTCTTGCAATGTCGGGAGATTGATG

AAGCAACACTAGGATGGGTTCTGTATAGAACCAAATAATGGATATAATGATTGAAAAAAT

AAGAGATATGATGCAAGAGGCTTGTAGGTAAATTCCCAGCATTTGGTATTCCTTTGCACC

AAATCCTTGTCCGCAGAGTGTTTCCAGTGCCCCACTCAAACCAAC

>**Gsoja_MSTRG.61707.50** GeneID=Gsoja_MSTRG.61707;Strand=+;Pos=Chr14:2477086-2477438,2477511-2477911,

GAAAATGATTGGACAAGAACACCTAAATTTGTTTCATTAATTATTATTTGCAACATCAAA
TGTTTGTTTCACATATATTAGACAAGTTTTTTTTTGGAATAAAGCAACTTCAAAGCACC
CATCATTTTTATTATACTATTTCTTCCTTTGCCTTAATCATGAGCTTAATAAAGTTTTCA
TCCTGAAGTTGTAATTGGTGCTCTTGGTGGCCCTGAAATTGTAATCGGTGCCCTTGGCGA
CCCTGCCTTTGGTGGCCCCGAAGTTGTAATCGGTGCCCTTGGTGGCCCTGCCTTTGGTGG
TCCCGAAGTTGTAATTGGTGCTCTTGGTTGCCCTGCCTTTGGTGGTCCTGAAGTTGTAAT
CGGTACCCTTGGGGGCCCTTCCTTTGGTGGTCCCGAAGTTGAAATTGGTGCTCTTGGTAG
CCCTGCCTTTGGTGGTCCTGAAGTTGTAACTGGTGGCTCTGGTTCTGAAGCTGTAACTAG
TGCCCTTGGTGACCCTGCCTTTGGTGGCCCCAAAGTTGTAATAAGTACCCTTGGGGGCCC
TTCCTTTGGTGGCCCCGAAGTTGAAATTGGTGCTCTTGGTGGCCCTGCCTTTGGTGGTCC
TGAAGGTGTAACTGGTGGCTCTGGTTCTGAAGCTGTAACTGTGAGAACAAATACAACTAG
TAGATGAAAAATTGCAACAAACACTTTCTTCATTTTGTAAAACGAATTTCTATCTCAATA
AACTTTTTAGGATACACTTGTATTTTGATGGGAA

>**Gsoja_MSTRG.14969.1** GeneID=Gsoja_MSTRG.14969;Strand=-;Pos=Chr04:7659745-7660042,

AGACGGAGACGCAGTTAGGAACAGCTAGTGCGAGAAGGGAAGACCATCCCCTGAGGCAGT
CGGTGCTAGGGGGAACCCACGAGTCCTTGTAGACACGAGAGAAGTAAACAAAGGAAGAAA
GGAAGATGAAGAGGTTTAGGTTGGTCCACACCATGGCTATTGCAACGCCAGACACTCCCA
TTTTAAAGTGGACAACTAATAGGAAGTTGAGAGGGACGTGGAGGAGGACGGAGATTGCGG
AGCAGTAGGTGAGGGGTAACGTGATGCTTTGTGTTCGGAGGTAGATTCTCAGAGGGTG
>Gsoja_MSTRG.26694.1 GeneID=Gsoja_MSTRG.26694;Strand=+;Pos=Chr06:46819304-46819564,
GAGCCAACTCAGGGTGAGGAAGCAATCCTGCAAGCAGAACCAAAATTTGGAAGTACCACG
TTTCTAAGCACAGCATCACCGCTGAAGCTGCAGACAACTTAAAAAACTCTGCTAACCCCG
AAAATGCTTGAAAACTGAACCCTCTCCACGTGTGCTTACACCTCTCACTCTTCACAATGT
ACACAAACTGCGCAATCACAATGATCCACCACGAAACACTCAACACCAAAGATGCACCCA
ACAGACCAAGCCCCACCTTAT

>**Gsoja_MSTRG.417.2** GeneID=Gsoja_MSTRG.417;Strand=-;Pos=Chr01:2612174-2612427,2612458-2612933,2696306-2696317,

AGAAGAAGAAGAAAGAAGAAAACACATAAAACCCGAAGAAGAAGAAGAAGAAAAAAGAAA
CCGCTACTCAACATGACCAGAGGATGAAATAAATCAAATAGATCCCGTCCGTCCGTGTGC

GGAAAAGAAAGATCAAGGGCGGAAATGCCACCACGTGGGACGAATCAAAGACAATTGTTC

TCATGAAAGATCTTAAGCCACCAAGGCCATGGCTACGAAGGTTCCAGCGAGTGCAGAGGT

AGCAATGAAGGCCTTGCTGACGGAGAATGCAGCGGAGGGCGGTGGAGGCTCGGCGCCACC

GGAGGGTCCGGGTGAGGGTCCGGGTGGAGTGTTAGGAGAGGTGGGGCTTGGTGTCACTGT

AGGAGAAGGTGAAGGAGGAGAGGCTGCAGGAGAGGAAGCAGGTGTAGGTGCAGCCGTTGG

TGGTGCTGGTGCTGGTGTTGGTGTTGCTGCTGGTGGTGGAGTTGCTGCTGGTGGTGGGGT

TGCTGGTGTGGTGGGTGCTGGTGCAGGTGCTGATCTGGGTGGTGGTGGTGATGGGGTTGT

AGTGGGTGGTTGTGACGGTGCAGCACCTGGGGCTTGGGCTAAGCATGAGCTAGCCAAGAG

CCCCAAGATCAGAAACAGTTGCACAGCACCAGAACCCATATCTAGAGAGAAAGTAGTAGT

AGTAGTGCTTGGGTGTGTAAGAGAGAGATAAGAGAGTTTGAGCAGTGGAAAGATGATAAG

AGAACAGAATATATAAAAAGAG

>**Gsoja_MSTRG.57914.1** GeneID=Gsoja_MSTRG.57914;Strand=+;Pos=Chr12:44102584-44103337,

AGAAATAGAAGAAGGAGATGGGTCCATACGATATGTATGATATTATATTGTAACAAGACG

ACAGATAAAGAAAAATGAAAAATGTCAATCAAGTATATATAGTGTGTAAAAGTACATGCA

TGTCTCATTACTTGCTATGAAATGATTGTTGACAGGATATATACAAATACCTAAATGACC

TATGAATGCTGTGTCTATGAGAGAAGCAATGGGATCAGCAGCAATAGCCAGTGCCGAGGG

GAATGCAATCCCTAGTATCTCCCGAGAAAGAGCATCCAACTTGGAAACATTCCTACATTT

ATTCACATTAGCAGAATCATATCACTCAAGAGCTTCATGTAAATTCAATAATGTAACCAC

ATATATGACAAGATAAGATCTTTTATAATTATTCTAGAAAGCATAATGTTCTCCTTCAAG

AAAAATAGCATAATGTATATATGCGCTGAATATATACCTCGCATCTCTGAAGAAAACAAA

GAGAGGCATCTTCCACTTGTTGTTGGGCTCGTTGGAACATCCATTCTCGTCCATTGGATT

GTGAATCTCACACAAGAGATCTTCACTGAAGTGCTTGAAACAATTGATCAAAAGAAGAAA

TTAAGATCGTTCACGATAATTAAGGTCAATCCTCAACTATGTACCTGCCAAGTTAATCAT

AATTTGAGTTTGAGATCGTCCAATATTAATGTTTATATAATGTTCACATTAATCAAAACT

AAGAAGGAATGTATATATATATGATGGTCACGCC

>**Gsoja_MSTRG.52475.13** GeneID=Gsoja_MSTRG.52475;Strand=-;Pos=Chr11:45344107-45344832,45345007-45345162,45345511-45346449,

TTACAACTCACATTGGTAACCATAGATACACAGATTATACGACATAAAATTAATTATGAG

ACGCGATCTCATTTGTCCAAGTAAAAATATTAGAAAAAATATATAAAAGTGTTCACAATT

ACTATATACTATAACTCAAGTTCCAAAGAAAGTTTGATTTTTCTAATCAATAGTGGTACT

TAGAGCAGCACCGACCAGAACTATAACTCAAGTTCCAAAAAAAAGTTTGATTTTTCTAAC

CAAGAGTGGTGCTTAGAGCAGCACCGACCAGAATGGTTAGTGAATACACTCCAAACCGCG

ACGGCGTTATAGACGACGCACCGTTTAGTCTGGCTGGCGTCGTTTCGTTCGACGACGACG

GCGTTTGAGATGATCCCGACGACGACGACGGCGAGGGGGTTGTTGCCGCCGCAGACGGTA

ATGGCGGCGATGTTGCCGGGGTGAGGGAGGAAGAGGGTGGGTAAGTGGGTGGTGCAGTTG

AAGCAGAAGGTGGGTAAGATGGAGAGAGTGAGGTTGAGGGAGGGATGGTGGATGGATAGG

GCGATGATGGGTAGGGTGGTTGAGATGTTGGGGAAGGAGTGTTTGGAGGTGTTGGATTAA

TGTAAGGTGGTTGAGATGTTGGGGAATGAGAGTTTGGAGGTGCTGCAATGGAAGGAGGTT

GGGAAGTGGGTGAAGGAGTGGTGTTTGGAGGTGTGGGAATGTAAGGTGGTTGAGATGTTG

GAGATGGGGTTATTGGAACAAAGGGTGGTTGATGGGCAGGGGAAGGCGTTTTTGGAGGGG

TTGGAGTGTAGGGAGGTTGAGAAATAGGAGAAGGAGTGTTTGAGGGAGTTGGGACATAAG

GTTGGTTGCCAGGGGGAGGTGTTTTTGGAGTAGTAGTGGGAACATTTGGAGGACTATAAA

TTGGAGAAGGTGTCTTTGGTGGTGTTGAAACATAGGGAGGTTGGTTATGAGGGGAAGGAG

CATTTGGAGGACTATAAGCAGGAGATGGTGTTTTTGGAGGAGTAGGAACATTTGGTGGAG

TGTAAATAGGTGAAGGTGTTTTGGGAGGAGTGGGAACATTAGGAGGATTATAAGAAGGAG

AAGGTGTTTTTGGAGGTGTAGGAACATAGGGTGGATTGTTACTAGGGGAAGGAGCTTTTG

GGGGAGTGTACACAGGAGAGGGTATTTTTGGAGGCGTTGGAAAATTAGGAGGAGTATAAA

CTGGGGAAGGAGTTTGTGGAGGAGTTGGAATATATGGAGGATGATTAGCAGGTGAAGGTG

CTTGAGTGGGAACATTGGGAGGAGTGTATACAGGGGAAGGAGTTTTTGGAGGTGTTGGAA

CATTAGGTGGATAATGAATAGGTGAAGGTGGTTTTGGATGAACATAGGGAGGGCTGGAAA

TAGGTGAAGGTGTTTTAGGAGGAGGTATTGGAACATAGGGTGGATGATAAGGAGTTTCTG

GAGGAGTTGGAACAGTTGGAGTTTGAGGAGGTGCTGGAACATAAGGTGCTCTCGGAGGAG

TAATTGGAACATAAGGAGTGTTTGGAGGGGTTGGAACATAAGGTGGATTTGGATGATAAG

GTGCTTTTGGAGGAGTAATTGGAACGTAAGGAGTGTTTGGAGGTGTCGGAGAATAATAAG

GAGGTGGCTCTCTCACAGCCAACACCACAATAATCAATTTCTGACCCTTCTCACAATTAT

CATCTTTCCCACTAATGAAGTAAAATGGACCCGATCTATCAAACTGAAACTCCGAGTCAC

CATCCTCAAACTTCTTGATCGGGTTCTTCTTGTTACATTTCTCATAATCATCATTGTTCA

CCACCAACACAGTATCTGAGT

>**Gsoja_MSTRG.52475.6** GeneID=Gsoja_MSTRG.52475;Strand=-;Pos=Chr11:45344091-45344616,45344833-45344844,45344941-45345072,45345247-45345336,45345511-45345654,45345841-45345843,45345895-45346448,

TACAACTCACATTGGTAACCATAGATACACAGATTATACGACATAAAATTAATTATGAGA

CGCGATCTCATTTGTCCAAGTAAAAATATTAGAAAAAATATATAAAAGTGTTCACAATTA

CTATATACTATAACTCAAGTTCCAAAGAAAGTTTGATTTTTCTAATCAATAGTGGTACTT

AGAGCAGCACCGACCAGAACTATAACTCAAGTTCCAAAAAAAAGTTTGATTTTTCTAACC

AAGAGTGGTGCTTAGAGCAGCACCGACCAGAATGGTTAGTGAATACACTCCAAACCGCGA

CGGCGTTATAGACGACGCACCGTTTAGTCTGGCTGGCGTCGTTTCGTTCGACGACGACGG

CGTTTGAGATGATCCCGACGACGACGACGGCGAGGGGGTTGTTGCCGCCGCAGACGGTAA

TGGCGGCGATGTTGCCGGGGTGAGGGAGGAAGAGGGTGGGTAAGTGGGTGGTGCAGTTGA

AGCAGAAGGTGGGTAAGATGGAGAGAGTGAGGTTGAGGGAGGGATGGTGGATGGATAGGG

CGATGATGGGTAGGGTGGTTGAGAAATAGGAGAAGGAGTGTTTGAGGGAGTTGGGACATA

AGGTTGGTTGCCAGGGGGAGGTGTTTTTGGAGTAGTAGTGGGAACATTTGGAGGACTATA

AATTGGAGAAGGTGTCTTTGGTGGTGTTGAAACATAGGGAGGTTGGTTATGAGGGGAAGG

AGCATTTGGAGGACTATAAGCAGGAGATGGTGTTTTTGGAGGAGTAGGAACATTTGGTGG

AGTGTAAATAGGTGAAGGTGTTTTGGGAGGAGTGGGAACATTAGGAGGATTATAAGAAGG

AGAAGGTGTTTTTGGAGGTGTAGGAACATAGGGAGGTTGGTTATGAGGGGAAGGAGCATT

TGGAGGACTATAAGCAGGAGATGGTGTTTTTGGAGGTGTTGGAACATTAGGTGGATAATG

AATAGGTGAAGGTGGTTTTGGATGAACATAGGGAGGGCTGGAAATAGGTGAAGGTGTTTT

AGGAGGAGGTATTGGAACATAGGGTGGATGATAAGGAGTTTCTGGAGGAGTTGGAACAGT

TGGAGTTTGAGGAGGTGCTGGAACATAAGGTGCTCTCGGAGGAGTAATTGGAACATAAGG

AGTGTTTGGAGGGGTTGGAACATAAGGTGGATTTGGATGATAAGGTGCTTTTGGAGGAGT

AATTGGAACGTAAGGAGTGTTTGGAGGTGTCGGAGAATAATAAGGAGGTGGCTCTCTCAC

AGCCAACACCACAATAATCAATTTCTGACCCTTCTCACAATTATCATCTTTCCCACTAAT

GAAGTAAAATGGACCCGATCTATCAAACTGAAACTCCGAGTCACCATCCTCAAACTTCTT

GATCGGGTTCTTCTTGTTACATTTCTCATAATCATCATTGTTCACCACCAACACAGTATC

TGAGTCCTTCTTGTACTTAAA

>**Gsoja_MSTRG.437.1** GeneID=Gsoja_MSTRG.437;Strand=+;Pos=Chr01:2762297-2762533,

CAAAAATGGTGTGAGGTCCTTAACTAGATCTTGCACTTCTGTGTCGTTTGAAAACAGGGA

AGGGTACTCATTCCGCGAGATCATCAAAACAATAGCGAGTAAAACTCCGATCAAAACCGA

AGTAATCACGGCAACCACTAGGGAAAATAATGCTGTTCTTGGGTGGCGTGCCCCTAGTTC

ATTCGATATTCTCACACTGAAATTTCATCCAAACTTTACATAAAAAAACAATAGTCT

>Gsoja_MSTRG.9430.1 GeneID=Gsoja_MSTRG.9430;Strand=+;Pos=Chr03:492299-492519,

CAGTGAATACAAGAGCATAGGCAGCGAGTTCTCTTGAACCAATATGACCAATAAAAGCTT

GGCTTATAACATTGATGCCAAAGGTCGTAAACCTTGTGAATATGGCTGGTGCTGCCACTA

TCCACATCTCTTTGCTCTCTTCCCACACCCTCTTCACCAATGATAAATTATCTTCTTCTG

ACACTTCCTCTTTGCTCAGCAACTTCTTCTCTAGATTCCCC

>**Gsoja_MSTRG.39207.1** GeneID=Gsoja_MSTRG.39207;Strand=-;Pos=Chr09:18274102-18274358,

ATTCATTTCCACATTCTCAGTCTCATTGTTCTCTTTGGATGCAACTTTCTCTATGTCTTG

AAGGGAATGATCATCGAGCATTACTTGTTCTTTGGACTTAGCCTTATCACTTAACTTCTT

TTCTGTATTGATTTTTTCAATGGTATTTTCCTCAGCTACGAAGGAAGTTGTGATGCTAAC

CAAAGGGAAGATGGTAATCCTTGAAGCTTGGTTGAACAAAGCAATGGACACTCCTGCAGC

AGCAAGCTCCACGGGCC

>**Gsoja_MSTRG.52475.33** GeneID=Gsoja_MSTRG.52475;Strand=-;Pos=Chr11:45344784-45344988,45345088-45345178,

GTAGGAACATAGGGAGGTTGGTTATGAGGGGAAGGAGCATTTGGAGGACTATAAGCAGGA

GATGGTGTTTTTGGAGGAGTAGGAACATTTGGTTGGTTATGAGGGGAAGGAGCATTTGGA

GGACTATAAGCAGGAGATGGTGTTTTTGGAGGAGTAGGAACATTTGGTGGAGTGTAAATA

GGTGAAGGTGTTTTGGGAGGAGTGGGAACATTAGGAGGATTATAAGAAGGAGAAGGTGTT

TTTGGAGGTGTAGGAACATAGGGTGGATTGTTACTAGGGGAAGGAGCTTTTGGGGG

>Gsoja_MSTRG.77249.1 GeneID=Gsoja_MSTRG.77249;Strand=+;Pos=Chr17:40642095-40642385,

ACCATGACTCCAAAGGCGAGGCCAGCAATAACAGAATTTTCAACAGAAACTGCTGCAAGA

TCAAGGTCACCAACAAGGCCAGCAAATGTTTGAGTGAGTGCACCAAGTGAGTACTGACAT

ATAGAGGTAACGATAGCAGGGCCAGCTATTTTCCATAGCTTCTTCGACTCAAACCCAAAT

CTCTTAACAACAGTGTTCGGTGTCTTCTCCTCCTCAACATTAGAACAAGAGTTGTCAATG

TTTTCTAGTAAGGGAGAATCCATTGGCTTGGTTAGTAGGTTACTTGGCACT

>**Gsoja_MSTRG.65598.1** GeneID=Gsoja_MSTRG.65598;Strand=-;Pos=Chr15:2794482-2794793,

GATTCTATATTTCATTTCATTTCACTATATATGAGACTATGAGAGTAGAGTTAGAGATTT

TTCTGTACATACTTATGATGTAGAGGTAAATTTTTCAACCAATACCCCAAGCACAATCAA

GCAGGGAAAAGTGCCTATTCTTTCTGAAGAATTTTTTAAGCAACTTAAAAACCTAGGGAA

AGCTTCTCTTCACTTAAATGTAATCAGTAAACACCACTGAAAACTAGGCCTAATATGAAG

CTATAACTACGGTGTCTGCCATGTGCAGTCATGAGCTCCTTAGGAACTCCCAAGGTCCTG

ATCCCGTTCCAA

>**Gsoja_MSTRG.52475.7** GeneID=Gsoja_MSTRG.52475;Strand=-;Pos=Chr11:45344091-45344832,45345007-45345162,45345262-45346448,

TACAACTCACATTGGTAACCATAGATACACAGATTATACGACATAAAATTAATTATGAGA

CGCGATCTCATTTGTCCAAGTAAAAATATTAGAAAAAATATATAAAAGTGTTCACAATTA

CTATATACTATAACTCAAGTTCCAAAGAAAGTTTGATTTTTCTAATCAATAGTGGTACTT

AGAGCAGCACCGACCAGAACTATAACTCAAGTTCCAAAAAAAAAGTTTGATTTTTCTAACC

AAGAGTGGTGCTTAGAGCAGCACCGACCAGAATGGTTAGTGAATACACTCCAAACCGCGA

CGGCGTTATAGACGACGCACCGTTTAGTCTGGCTGGCGTCGTTTCGTTCGACGACGACGG

CGTTTGAGATGATCCCGACGACGACGACGGCGAGGGGGTTGTTGCCGCCGCAGACGGTAA

TGGCGGCGATGTTGCCGGGGTGAGGGAGGAAGAGGGTGGGTAAGTGGGTGGTGCAGTTGA

AGCAGAAGGTGGGTAAGATGGAGAGAGTGAGGTTGAGGGAGGGATGGTGGATGGATAGGG

CGATGATGGGTAGGGTGGTTGAGATGTTGGGGAAGGAGTGTTTGGAGGTGTTGGATTAAT

GTAAGGTGGTTGAGATGTTGGGGAATGAGAGTTTGGAGGTGCTGCAATGGAAGGAGGTTG

GGAAGTGGGTGAAGGAGTGGTGTTTGGAGGTGTGGGAATGTAAGGTGGTTGAGATGTTGG

AGATGGGGTTATTGGAACAAAGGGTGGTTGATGGGCAGGGGAAGGCGTTTTTGGAGGGGT

TGGAGTGTAGGGAGGTTGAGAAATAGGAGAAGGAGTGTTTGAGGGAGTTGGGACATAAGG

TTGGTTGCCAGGGGGAGGTGTTTTTGGAGTAGTAGTGGGAACATTTGGAGGACTATAAAT

TGGAGAAGGTGTCTTTGGTGGTGTTGAAACATAGGGAGGTTGGTTATGAGGGGAAGGAGC

ATTTGGAGGACTATAAGCAGGAGATGGTGTTTTTGGAGGAGTAGGAACATTTGGTGGAGT

GTAAATAGGTGAAGGTGTTTTGGGAGGAGTGGGAACATTAGGAGGATTATAAGAAGGAGA

AGGTGTTTTTGGAGGTGTAGGAACATAGGGAGGTTGGTTATGAGGGGAAGGAGCATTTGG

AGGACTATAAGCAGGAGATGGTGTTTTTGGAGGAGTAGGAACATTTGGTTGGTTATGAGG

GGAAGGAGCATTTGGAGGACTATAAGCAGGAGATGGTGTTTTTGGAGGAGTAGGAACATT

TGGTGGAGTGTAAATAGGTGAAGGTGTTTTGGGAGGAGTGGGAACATTAGGAGGATTATA

AGAAGGAGAAGGTGTTTTTGGAGGTGTAGGAACATAGGGTGGATTGTTACTAGGGGAAGG

AGCTTTTGGGGGAGTGTACACAGGAGAGGGTATTTTTGGAGGCGTTGGAAAATTAGGAGG
AGTATAAACTGGGGAAGGAGTTTGTGGAGGAGTTGGAATATATGGAGGATGATTAGCAGG
TGAAGGTGCTTGAGTGGGAACATTGGGAGGAGTGTATACAGGGGAAGGAGTTTTTGGAGG
TGTTGGAACATTAGGTGGATAATGAATAGGTGAAGGTGGTTTTGGATGAACATAGGGAGG
GCTGGAAATAGGTGAAGGTGTTTTAGGAGGAGGTATTGGAACATAGGGTGGATGATAAGG
AGTTTCTGGAGGAGTTGGAACAGTTGGAGTTTGAGGAGGTGCTGGAACATAAGGTGCTCT
CGGAGGAGTAATTGGAACATAAGGAGTGTTTGGAGGGGTTGGAACATAAGGTGGATTTGG
ATGATAAGGTGCTTTTGGAGGAGTAATTGGAACGTAAGGAGTGTTTGGAGGTGTCGGAGA
ATAATAAGGAGGTGGCTCTCTCACAGCCAACACCACAATAATCAATTTCTGACCCTTCTC
ACAATTATCATCTTTCCCACTAATGAAGTAAAATGGACCCGATCTATCAAACTGAAACTC
CGAGTCACCATCCTCAAACTTCTTGATCGGGTTCTTCTTGTTACATTTCTCATAATCATC
ATTGTTCACCACCAACACAGTATCTGAGTCCTTCTTGTACTTAAA

>**Gsoja_MSTRG.57217.1** GeneID=Gsoja_MSTRG.57217;Strand=-;Pos=Chr12:39971164-39971424,
ACTTCAAATTGCTGCTTAGACAACTAGAATTAATCCTTGGCAGTTTTAATTTTTAATTCC
CTACCTCATAATGGAACAACGAAGTGTTAAACTACCTTGTTGAATCTCTAGAATTAGTCC
TTATAAATTGGTTTCAATAAAAAATAATAATAAAGAGAATATTATGTCTCTCTTGCTCTC
CTACATAAACAAGTTATTCTTCAATTTAGTAGTGGTTCCTTCTTGTCCTCCCATTTGGTC
AGCCTCTTGGCTGCTTCCTCT

>**Gsoja_MSTRG.89036.1** GeneID=Gsoja_MSTRG.89036;Strand=-;Pos=Chr20:42497574-42499349,
TGATAATAAAGAATCTTCCTTGATTTCAGCTTTCGGTGGCTTCTCTGCACCAACCTCCTT
GCTGTCATCAACAACAACACTGGTTAGTTTCTTTGCCCTTAGAGCCTCAGCATCCCAATC
CGTTCGACTCAGAACCACCAACATGGTCACTGCACAAGACCCCTGAGCCGCAAGTAACCC
AAGCCACAACCCTTGAAAATCAAACCCTGCGAAAAAACCAAGCCAAACCGCCACAGGCAT
TCCCACAAGATAGAAACAACCTAAGTTAATGTTGGCACCAACTTTAGGCCTTGCTGTGCC
TCTCAGCACTCCACACCCCGTTGTTTGAGGACAGTTTCCGAGTTCGCAGAGTCCTATAAC
CGGTAGCACAAATGAGGTCAAAGTTATTATCTCCTTGTCTTGTGTGAACATGTTGGCCCA
AATGTTCCTAACCAGAATGGTGAAAACCAAAGCGAAAACACCTAACATGAAGCTGCAAGA
GAGGCCAACTATGGAAGAAAATTTTGCCTTCGAGGGCTTTTGCGCGCCTAATTTGTTGCC
AACTCTTGTGGACACGCTGAAGCTTATTGACGATGGGAAAATGTAGAGCAAGGAAGTTGT

TTGAATCAAAATCCCCATGGAGGCCACGGTTGCTCTAGGATTCACCAACAACCCGCATAA

CAAAATCATGATCTCATACCACCACCATTCTAGGCACACGGAAATGCAGCTTGGGATAGC

CAAATTGAGGAGCGATTTCCATTGTGTAAAACACTCGAAAGAAAAGCCTCCCCATGTTTT

CTTATGGGTGCCAGAGAAGACTATGTAGAGAATCAAAGAAGCTACGAGGTTGAGGTTAGT

CCAAACCCCGCTGAGAGCCACGCCTTTGATTCCCCAATTGAGGTGGGAAACGAGAAGGTA

GTTGATAGGAATATGGAGGAGAATTGAGAAAGTGGCACAAAGAGTGAGAGGCAGAGTAAT

GGATTGGCTTCGAAGGTAAATTCTTAAAGGGTGTAAAAAAGATTGTGCTAAGAGGTCAGG

GATGGAATAAAGAAGATATGATTGGGCTTGTGTGGCGATAGCCTCGTCTTGGCCACATAA

TAGAAGGATATGCTTCATGTAGAGCCAAAGGAGAGAGATTGGGATACAAGTGAAGAGGAG

CAAGAGAATGGTTCTTTGTAAGCAGAGGCCGAGGAGGGAGAACTTTTTGGCACCGTAGGC

TTGTCCGCAAATGGATTCCATTCCGACGGCGAGGCCGGAGAGGATAGAGTAGCCGCTGAT

GTTGGCGAAGCCCACGGCGAGTGAGCCGCCGGCTAAGGCCAGCTCGCCGAGGCGGCCCAG

GAAGAGCATGGAGATCATGGAACGGCAGTAGAGCAAGAGACCGGTGAGGATCATGGGGAA

GGCTATCTTGCATATTGAAATGAGTTCTTTCAAGACATGATGATGGGTTTGCATTGTTCT

TGGGTCGTTTTTTTCTTCATGGAGTGTTGTTAGTGTTACTGTTATGTCCTTTTGGATCAA

GGGGTTGGTGATCATCATGTCTGCCTCTGGGGGTTTCATTGAAACTACATACTCCGGGTT

TGAGTTGCATTTGCAGGGGAGGTGGGAAGTTATATGACACATATTTTCTGCTTTTTGTGT

GTGTGTGTGTCAAGAAAAGAGTGGTTTAGAGAACACGTGAAAGTTGAACCGAGAAAGAAA

GAGAGAGATAGAGAGGATTATAGGGTGAACGGGAGGGGGTTGAGCACAGGCTTGTCTTGG

GGCTTTTATAGGAGATTTTGGGTGGGAATATGGGCG

>**Gsoja_MSTRG.52475.18** GeneID=Gsoja_MSTRG.52475;Strand=-;Pos=Chr11:45344491-45344646,45344989-45345006,45345181-45345336,45345511-45345654,45345841-45345843,45345895-45345986,

AGTGGGTGGTGCAGTTGAAGCAGAAGGTGGGTAAGATGGAGAGAGTGAGGTTGAGGGAGG

GATGGTGGATGGATAGGGCGATGATGGGTAGGGTGGTTGAGAAATAGGAGAAGGAGTGTT

TGAGGGAGTTGGGACATAAGGTTGGTTGCCAGGGGGAGGTGTTTTTGGAGTAGTAGTGGG

AACATTTGGAGGACTATAAATTGGAGAAGGTGTCTTTGGTGGTGTTGAAACATAGGGAGG

TTGGTTATGAGGGGAAGGAGCATTTGGAGGACTATAAGCAGGAGATGGTGTTTTTGGAGG

AGTAGGAACATTTGGTGGAGTGTAAATAGGTGAAGGTGTTTTGGGAGGAGTGGGAACATT

AGGAGGACTATAAATTGGAGAAGGTGTCTTTGGTGGTGTAGGAACATAGGGAGGAGTGTA

TACAGGGGAAGGAGTTTTTGGAGGTGTTGGAACATTAGGTGGATAATGAATAGGTGAAGG

TGGTTTTGGATGAACATAGGGAGGGCTGGAAATAGGTGAAGGTGTTTTAGGAGGAGGTAT

TGGAACATAGGGTGGATGATAAGGAGTTT

>**Gsoja_MSTRG.52475.23** GeneID=Gsoja_MSTRG.52475;Strand=-;Pos=Chr11:45344733-45344898,45345247-45345354,45345529-45345672,45345862-45345960,

GTGGGTAAGATGGAGAGAGTGAGGTTGAGGGAGGGATGGTGGATGGATAGGGCGATGATG

GGTAGGGTGGTTGAGATGTTGGGGAAGGAGTGTTTGGAGGGGTTGGAGTGTAGGGAGGTT

GAGAAATAGGAGAAGGAGTGTTTGAGGGAGTTGGGACATAAGGTTGGTTGCCAGGGGGAG

GTGTTTTTGGAGTAGTAGTGGGAACATTTGGAGGACTATAAATTGGAGAAGGTGTCTTTG

GTGGTGTAGGAACATAGGGAGGTTGGTTATGAGGGGAAGGAGCATTTGGAGGACTATAAG

CAGGAGATGGTGTTTTTGGAGGAGTAGGAACATTTGGTGGAGTGTAAATAGGTGAAGGTG

TTTTGGGAGGAGTGGGAACATTAGGAGGATTATAAGAAGGAGAAGGTGTTTTTGGAGGTG

TAGGAACATAGGGTGGATTGTTACTAGGGGAAGGAGCTTTTGGGGGAGTGTACACAGGAG

AGGGTATTTTTGGAGGCGTTGGAAAATTAGGAGGAGT

>**Gsoja_MSTRG.46556.1** GeneID=Gsoja_MSTRG.46556;Strand=+;Pos=Chr10:50641045-50641577,50726117-50726126,

TACGGTAATAGGGGAGCCAGTGTTGAGAACGCGCCAGCCTCTAACGACACCTTCGGTGGC

ATCCATAGCAATGGTTCGGACAACGCCTTCACCCAAATGCTGCGCCACCTCCAACACAAG

CCTCGACGAGTGATCCAGAACCTCCAGCGCGGTCATGATCGGAGGCAAACCCTCGTCGAA

TCTGACATCGACGACGGCACCAATGACCTGGCAGACCTGCCCGATCGCGCCCTTCCCGGT

GAATTCATCGGTGATCTTCCCGCCGCCGCCGAGCTCCTTCTTCCCCGGAGGAGGAGGAGA

GGGAGGAGCGGTAGCAGCAGCCGCGGCGGTGGCGTACTCGGCGACGCGGTTCAGCAAGTA

TCCGTGCGGTGAGGCACGGTTGGATGACGTGAGCCTCGATGTCGATGCGGAAATTGAGGG

CTTCGATTGAGATCTACGAAGGGAGGATCGAATCAGAGAAGATACGAGCCTGCGTGAAGC

CATCTTCAGAGAGAGAGAGAGAGAGAGAGAGAAAGAAAGAAGAGGGCTAGGGTTTTCATGGA

TGA

>**Gsoja_MSTRG.52476.1** GeneID=Gsoja_MSTRG.52476;Strand=-;Pos=Chr11:45319666-45320071,

CAAAAGATAGAGGAATTTACCATAACTGCCACGCCAGTGACACTGAACCATGAATTTGCA

AGAGTAGCACCAGCAAGCTGAAGCTCACCAAGGTGACCAACAAGCATGACAGAAACCAAA

ATGATCAAGTGATAGAACAAGTTGGTAAGAATCATTGGCAGTGAAAACAAGAGTTGGTGC

TTGGCCTCTTCCATGTCCAACACTTTGTTCCACCATCCCTCAGCTTGGGGGGATCTTGTG

TTTTGATCTCTTCCATTATTATCAGATGCTCTCAATAGGGGTACTCCTGTTCCTTCTGAA

CTAGAACTTGCCACCATCTTCTCTATCTCCTCTTTTATCTGCTCCACGTTTTCCATATAT

ATGTGTAAGGAAGCACTTTGAATTTTTGGTGATATTAATATAAGCC

>**Gsoja_MSTRG.84379.1** GeneID=Gsoja_MSTRG.84379;Strand=-;Pos=Chr19:37666702-37666711,37713437-37715223,

TATTATTGGCTACATTATGTGGTAGAGTTATGTGTATACCTCTATTGCGTTTGCAGTAGC

ATGTCCAAGATCATTATGACAGTGCATAGAAATAATAACATTTTCTGCTCCAGGGACATT

GGCTTTTATATCAGCAATCAATTGTCTAATCTCGAATGGCATTGTTATGCCCACAGTGTC

ACCTATGCCCAAAGTTGTTGCCCCAGCTTTGATAACTTCTTCCAAAATTTGATAAAGAAA

CTCTCTATCCGATCTGGTCTCATAATGGTTGAATAATGTAATAAGAAGAACTCAAAATTC

ATGACTATATATATCAACATTTGACACCAACAAAAATGTATATAATATTCACTTCATACA

AAATTACACTATTTACACTTATTTATTTTAATACTACTTAATTTTTACTTTTAAATATAG

GGTTAGTTTGCTTATTCTTATTTTTTTCAAGTAAGCATTTTTTTAAATAAAATAAAAATT

TTATTTTTTTTCTAATATGTTTGTTTAAACTATTTACTTGAAATAAGTAATTCTATCTAT

TTATTAAAAAAAATTAAAAATCACCTATTTTAAAATTATTTTTTAAGTTTAAACAAAATC

ACCCATAAATCATAAATACTTCATTTGTCTCTAATTATAAGACTCTTTTAATTAATTCAC

ACCCCTTAAATAATTAATTTAATTAATCACATTAAATTTATCAATTATTTATATTATCAT

TTTAAAATTATTCTTTTATTATCACTCTATCCATTTAATTATTTCTCATTAATATTTAGA

GAGAAAATAATTAAATATATAAAAGAAAAAATAATTAATACATCTATAAATCAAAAAAAA

TATCTTATTAAAAAAATAAACAAATTTTTAAAAATAGTCTTATAATCAGGAAAAAAAGTA

TAAGCTAAAAGCTATTTTAAATAATGTAGGAAAAAATGCATTATGAATCTTATTCTAAGT

GATAACTAATGCTACAAAACAAAACAAACAACTTAGTGTATGTTTGCGAATGCGATCCAA

TCGTGTTGAACGGAAAAAATAGACTTTCTGAGGCAAAATTTCCAAAAGCAACACCCATGT

TGCTTTCCCGTTGCATTGAAGCCAACGGAAATACAAACACAGCCTTAACCTCTGGAAAGA

TGAGGCAGAAACGTGGGTGTGCAACGTAGAATGAGCACAAACTAATAATTTGTCCGATAT

GATTGAGTTAACGCCTACCCTTGTCGTGTTTTTTCTTTATGATCACAGTAGTCTCGTCCA

AGCACTTTGGCCAAACGATAAAAAGAATATGGACAAGCGCAGTGGCATCGTGATTCGTTT

TTTTTTTCAGTAAGAAGGTGAGCGTGCATTACTAGGTGTTGGCTTAACTGGTGTACGCAG

GTGGTAGCATTGTGTTGGTGCAGTGTTTGGTGAACTTTGTGAACTACTGTGAGTGAGCAA

GGAAAATGCCAAACGAGAGGGTGTGTGTACCCGCGAGTTCAGGGACCTAAGAAGAGGGGC

AAAGGGTGAGTTTTATCAGTGTGTTTGCTTTGGGTTGGTGATTTCAAAATTATATATAGT

GAGTTTAATTACCACAAAAGCCACAATTTTTGTTGCCTTTAATTTAAAAATAATGTTGAG

CTTAAGTTTCCGCCAATCCAAACACAGTGAAAATTAAGAACAATGTTACATCTTACTTTA

GGGTGGATTGAAATAACTTGAATTCAATACTCTGCATCTGTAGAGAGGAGAGGTGAGGAG

AGAGAATTTAAAATTGAAATAAAAGAGAGATGGAAAAAAAGGAAAATATGCATCTTA

>**Gsoja_MSTRG.5327.1** GeneID=Gsoja_MSTRG.5327;Strand=+;Pos=Chr02:6508568-6509264,

ATCATAAAGCAACCACTCACAAGCCTCCAACCCCATTGATCCTACCCACCAAACAAAATC

CCAATGTCATAAATCATAATTGGTGTCATTTCATTTCTCTAGTGTGGTGTCTTTTTTCTT

AGCAAATGTCATCTTTGTTGTTCCCATTTCCATTCACCAACAACCCTTTTCTTTCTTCAT

CTCTCTCATTTTCCTTATTCCTAAGCCCATTGCAACTACCCATTTCAATCCTTGTGAGCT

TCTCAGCCTTCAAAGCCTCAGCTTCCCAATCTGTCCTCACCAACACAACATAGAGGATTG

ACACAGCACATGCAACCTGGGCCGACAGAAGCCCAAACCAAAGCCCACTGAACCCAACCT

TGAACCAAAATGCCAGGCCCACTGCCACCGGGGTGCCCACAAAGTAGAAAGAGCCCAGGT

TTATGTGAGCCCCAATACCAGGCCGGGCCATGCCACGAAGGATCCCACAGCCCGTGGTTT

GTGGGCAGTTTCCAAGCTCACACAAGCCCATAATTGGCATAACTGATGCAACCAACGCTT

TGACGGGCTCATCATTGGTGAAAAGCCCAGCCCATCTTTGACCCAGAATCACAGTCCATG

TCACATTGATGAAGCCTATCACAAAAGCGCATCCTAAGGCAACCACTGCTGCTAGCTTTG

CCTTGTATGGTTTTCCAGCTCCAAGCTCATTCCCTAC

>**Gsoja_MSTRG.77238.1** GeneID=Gsoja_MSTRG.77238;Strand=+;Pos=Chr17:40586745-40586952,40635551-40635867,40636051-40636135,

CAGCAATACTGTTTGTGGATAAGTATGCAAGAACCAATGCCAAGTACCCCTCAGCAACAC

ATGTTAGCCTTTAGAAGAGCGGGGGTCACATGAAAAGGAAAATGTTAGATGTTGTCTATT

TGGATGACTTGGATATGGACCATTTGAAATACAAGAAATGAGTGTGTCTTCACAAATAAG

TAGGTTACATGACAAATTTGGATTTAAGGTGGTGTGATTTATAGATAAGATTGAGATTCT

TTTGTTTAAATACTACTTAGTTAAACACTTGTAATTAGTGCTCAACCAAATGGTGCTCAA

TCATGTGAAATATCTATGTAGAAATCATAACAAGCTGGCCTATAATCTAAAATTAAATTT

CCAAGTCCTCACTCAAATACATGTCGAAAAATTTAAAGTTCTTCATTTATTGGTGCCACT

CAATAGAGTCTCTGTCTAATTCTCTACTATGATAAAAAAAATCATTGATCATACGAAATT

GCTCCTCCCCACTTTCTCACGCGGCTTTCTGCTTCTTCAGCCTGTTTTTTTCCAGTTTCG

TATTGAAGTGACGATTATAAGGATTGTAGTCTGCAAAACTATGCCTGCAATCATCCCTGA

CCAAATACCC

>**Gsoja_MSTRG.28715.1** GeneID=Gsoja_MSTRG.28715;Strand=-;Pos=Chr07:9570506-9571093,

AACATTTAATATTAGAGAAAATAGCAAAAATGTATTTAGATTAACATAATATTTTTTTT

ATTAATATCATACGAAATTCTATTAAATTGTGAACCATTCATTCTCCCACTGCTAATTTA

GTCTTTGGTTTGATTTTAACAGCCTAATTATGAATCTACGAGGGCACTTTCCTTTTATAT

ACAAAATAGTTGTACCAAAAAAAATGTTCACTAAAAACTGCTTTTCTAAATAATCAGAAT

CACTAAGCATAATTACTGTTCAACTGTCGAAGATTTTCATTTTGGTCTCTTGGCAGTGCA

TTTGCATCAGTTGGCACTCCGTTGCTTCTTATTCTTTTTGCTGCCTTATTTGCCTGAACA

GTATATATAAAGATATTGCGTTATTGATGGTATGATCCAAAGTTAGAATTTTTTTTATAG

ACAAATATTAATTATTTATTTATTAGTTTTTATAAGCAGACAAATGAAGATCGAATGGGT

GACCTTTCCCTCATTCCTTTCTTTCTCAACCACCGACCAATCTTACATTTCCTCCAAAAT

TGAAAATTGATAACACCAGTGATTTGAACGAAGACTTACTTCTTTCTC

>**Gsoja_MSTRG.61707.49** GeneID=Gsoja_MSTRG.61707;Strand=+;Pos=Chr14:2477086-2477438,2477466-2477912,

GAAAATGATTGGACAAGAACACCTAAATTTGTTTCATTAATTATTATTTGCAACATCAAA

TGTTTGTTTCACATATATTAGACAAGTTTTTTTTTTGGAATAAAGCAACTTCAAAGCACC

CATCATTTTTATTATACTATTTCTTCCTTTGCCTTAATCATGAGCTTAATAAAGTTTTCA

TCCTGAAGTTGTAATTGGTGCTCTTGGTGGCCCTGAAATTGTAATCGGTGCCCTTGGCGA

CCCTGCCTTTGGTGGCCCCGAAGTTGTAATCGGTGCCCTTGGTGGCCCTGCCTTTGGTGG

TCCCGAAGTTGTAATTGGTGCTCTTGGTTGCCCTGCCTTTGGTGGTCCTGAAGCTGTAAC

TGGTGCCCTTGGTGGCCCTGCCTTTGGTGGCCCTGAAGTTGTAATCGGTACCCTTGGGGG

CCCTTCCTTTGGTGGTCCCGAAGTTGAAATTGGTGCTCTTGGTAGCCCTGCCTTTGGTGG

TCCTGAAGTTGTAACTGGTGGCTCTGGTTCTGAAGCTGTAACTAGTGCCCTTGGTGACCC

TGCCTTTGGTGGCCCCAAAGTTGTAATAAGTACCCTTGGGGGCCCTTCCTTTGGTGGCCC

CGAAGTTGAAATTGGTGCTCTTGGTGGCCCTGCCTTTGGTGGTCCTGAAGGTGTAACTGG

TGGCTCTGGTTCTGAAGCTGTAACTGTGAGAACAAATACAACTAGTAGATGAAAAATTGC

AACAAACACTTTCTTCATTTTGTAAAACGAATTTCTATCTCAATAAACTTTTTAGGATAC

ACTTGTATTTTGATGGGAAT

>**Gsoja_MSTRG.61707.2** GeneID=Gsoja_MSTRG.61707;Strand=+;Pos=Chr14:2414297-2414306,2477172-2477438,2477763-2477912,

ACATTTTTTTTTTTTTTTTTGGAATAAAGCAACTTCAAAGCACCCATCATTTTTATTATA

CTATTTCTTCCTTTGCCTTAATCATGAGCTTAATAAAGTTTTCATCCTGAAGTTGTAATT

GGTGCTCTTGGTGGCCCTGAAATTGTAATCGGTGCCCTTGGCGACCCTGCCTTTGGTGGC

CCCGAAGTTGTAATCGGTGCCCTTGGTGGCCCTGCCTTTGGTGGTCCCGAAGTTGTAATT

GGTGCTCTTGGTTGCCCTGCCTTTGGTGGTCCTGAAGGTGTAACTGGTGGCTCTGGTTCT

GAAGCTGTAACTGTGAGAACAAATACAACTAGTAGATGAAAAATTGCAACAAACACTTTC

TTCATTTTGTAAAACGAATTTCTATCTCAATAAACTTTTTAGGATACACTTGTATTTTGA

TGGGAAT

>**Gsoja_MSTRG.5534.1** GeneID=Gsoja_MSTRG.5534;Strand=+;Pos=Chr02:7845482-7845723,

CAAAACACTAAACCCAGAAACATTGGTGAGAGAGGTCGCAATAGCAACACTTGAAAGAGA

GAGCTGGTTAAGATGGCCAACCATTACTAAGGACACCACTGGCAAAAGGTATTGTAACAC

ACTTGAAACCGCCATTGGAGCTGCTATAGTACCCACCTTCTTCAGCTCTTCCCTCATTTT

CATCCATCCCCACTTTCCTGTCGAACTTTCTTCCATCACTGCACCCTTTTATCATTTTTG

TA

>**Gsoja_MSTRG.52475.12** GeneID=Gsoja_MSTRG.52475;Strand=-;Pos=Chr11:45344092-45344898,45345073-45345162,45345262-45345336,45345511-45346404,

AAAATTAATTATGAGACGCGATCTCATTTGTCCAAGTAAAAATATTAGAAAAAATATATA

AAAGTGTTCACAATTACTATATACTATAACTCAAGTTCCAAAGAAAGTTTGATTTTTCTA

ATCAATAGTGGTACTTAGAGCAGCACCGACCAGAACTATAACTCAAGTTCCAAAAAAAAG

TTTGATTTTTCTAACCAAGAGTGGTGCTTAGAGCAGCACCGACCAGAATGGTTAGTGAAT

ACACTCCAAACCGCGACGGCGTTATAGACGACGCACCGTTTAGTCTGGCTGGCGTCGTTT

CGTTCGACGACGACGGCGTTTGAGATGATCCCGACGACGACGACGGCGAGGGGGTTGTTG

CCGCCGCAGACGGTAATGGCGGCGATGTTGCCGGGGTGAGGGAGGAAGAGGGTGGGTAAG

TGGGTGGTGCAGTTGAAGCAGAAGGTGGGTAAGATGGAGAGAGTGAGGTTGAGGGAGGGA

TGGTGGATGGATAGGGCGATGATGGGTAGGGTGGTTGAGATGTTGGGGAAGGAGTGTTTG

GAGGTGTTGGATTAATGTAAGGTGGTTGAGATGTTGGGGAATGAGAGTTTGGAGGTGCTG

CAATGGAAGGAGGTTGGGAAGTGGGTGAAGGAGTGGTGTTTGGAGGTGTGGGAATGTAAG

GTGGTTGAGATGTTGGAGATGGGGTTATTGGAACAAAGGGTGGTTGATGGGCAGGGGAAG

GCGTTTTTGGAGGGGTTGGAGTGTAGGGAGGTTGAGAAATAGGAGAAGGAGTGTTTGAGG

GAGTTGGGACATAAGGTTGGTTGCCAGGGGGAGGTGTTTTTGGAGTAGTAGTGGGAACAT

TTGGAGGACTATAAATTGGAGAAGGTGTCTTTGGTGGTGTTGAAACATAGGGAGGTTGGT

TATGAGGGGAAGGAGCATTTGGAGGACTATAAGCAGGAGATGGTGTTTTTGGAGGAGTAG

GAACATTTGGTTGGTTATGAGGGGAAGGAGCATTTGGAGGACTATAAGCAGGAGATGGTG

TTTTTGGAGGAGTAGGAACATTTGGTGGAGTGTAAATAGGTGAAGGTGTTTTGGGAGGAG

TGGGAACATTAGGAGGATTATAAGAAGGAGAAGGTGTTTTTGGAGGTGTAGGAACATAGG

GTGGATTGTTACTAGGGGAAGGAGCTTTTGGGGGAGTGTACACAGGAGAGGGTATTTTTG

GAGGCGTTGGAAAATTAGGAGGAGTATAAACTGGGGAAGGAGTTTGTGGAGGAGTTGGAA

TATATGGAGGATGATTAGCAGGTGAAGGTGCTTGAGTGGGAACATTGGGAGGAGTGTATA

CAGGGGAAGGAGTTTTTGGAGGTGTTGGAACATTAGGTGGATAATGAATAGGTGAAGGTG

GTTTTGGATGAACATAGGGAGGGCTGGAAATAGGTGAAGGTGTTTTAGGAGGAGGTATTG

GAACATAGGGTGGATGATAAGGAGTTTCTGGAGGAGTTGGAACAGTTGGAGTTTGAGGAG

GTGCTGGAACATAAGGTGCTCTCGGAGGAGTAATTGGAACATAAGGAGTGTTTGGAGGGG

TTGGAACATAAGGTGGATTTGGATGATAAGGTGCTTTTGGAGGAGTAATTGGAACGTAAG

GAGTGTTTGGAGGTGTCGGAGAATAATAAGGAGGTGGCTCTCTCACAGCCAACACCACAA

TAATCAATTTCTGACCCTTCTCACAATTATCATCTTTCCCACTAATGAAGTAAAATGGAC

CCGATCTATCAAACTGAAACTCCGAGTCACCATCCTCAAACTTCTTGATCGGGTTCTTCT

TGTTACATTTCTCATAATCATCATTGTTCACCACCAACACAGTATCTGAGTCCTTCTTGT

ACTTAA

>**Gsoja_MSTRG.77238.2** GeneID=Gsoja_MSTRG.77238;Strand=+;Pos=Chr17:40635557-40635865,40636048-40636151,

TGATTTATAGATAAGATTGAGATTCTTTTGTTTAAATACTACTTAGTTAAACACTTGTAA

TTAGTGCTCAACCAAATGGTGCTCAATCATGTGAAATATCTATGTAGAAATCATAACAAG

CTGGCCTATAATCTAAAATTAAATTTCCAAGTCCTCACTCAAATACATGTCGAAAAATTT

AAAGTTCTTCATTTATTGGTGCCACTCAATAGAGTCTCTGTCTAATTCTCTACTATGATA

AAAAAAATCATTGATCATACGAAATTGCTCCTCCCCACTTTCTCACGCGGCTTTCTGCTT

CTTCAGCCTCTCTTTTTTCCAGTTTCGTATTGAAGTGACGATTATAAGGATTGTAGTCTG

CAAAACTATGCCTGCAATCATCCCTGACCAAATACCCTGAATTACACAGGCAT

>**Gsoja_MSTRG.47510.1** GeneID=Gsoja_MSTRG.47510;Strand=+;Pos=Chr11:1887315-1888221,

TTGTGTTTTTCTTTTCTTTGTATGAATGCATGCGTCTATGCTCTGCCTCTTTAAAATTAG

CACAACCTAGAAAGAATCAATTTGATGCAAAAAATATAAAAGAATGGAGGTGACGTCGTT

GTGATGATCAATCAAACTAATAATGAATCAGAGCAATATTCCTTGGTAGCGCAGGAACTG

AAGAAGCAATTCTGATCTTGTTCCTCAGACGATGATGTGAGTTCTTTGGCTCTTTGAACT

TGGCCCTCCCAATTGGTGCGAGCCAACACAATTAACATGGTGAACATACAAGAGGCTTGG

GCGGCCAATAAACCAAGCCACAAGCCTTTGAAATCAAAGCCGGCAAAGAAACTCAACCTC

ACCGCAACGGGCATTCCCACTAGATAGAAGCAACCCAAATTTATATTCGCTCCCAACTTG

GGCCTCGCGGTTCCCCTCAAAACGCCACAAACCGTTGTTTGCGGACAGTTTCCCAATTCG

CAGAGCCCAATGATGGGCAACACCATCGACGTCAAAGCAATGATCTCCGCGTCACGTGTG

AACATCGAAGCCCACGCTTGCCTCACCGAAACGGCGAAAAATAATGCCGAGAAACCCAAA

CCGTAACTGATACATAACCCCACCAGTGCTGCGACTTTTGCCTTTTTGGGATTCTCCGCA

CCCAGTTCATTTCCGACACGTGTGGAAACCGCAAAGCTCAGGGAAGATGGGAAAATGTAT

ATCAGAGCAGTGGTTTGTATCAGAACCCCCATTGATGCAACCGTTGCTTGGGGATTAATC

AATAACCCGCAAAGTAAAATCATGATTTCGTACCACCACCACTCGAGGCATACGGAAATG

CAACTTGGAATTGCCAAATTCAAAAGCGATTTCCAACCGCTCAAGATGCCCTTCAACGAA

ACACCTG

>**Gsoja_MSTRG.47511.1** GeneID=Gsoja_MSTRG.47511;Strand=+;Pos=Chr11:1888673-1889280,

GCCCATGGCGAGGCCCGAGAGAATAGAATAGCCCGTGATGTTGGCGAATCCAATGGCGAG

AGAGCCACCAGCTAAGGCAAGCTCACCCACACGACCGAGGAAGAGCATGGAGATTACGGA

GCGAGAATAGAGTAATAACCCGGTTAGCACCATGGGTAATGCTATGTTGGCTATGCATTT

GACTTCGTTGAGAGCGAGGGAAAAATGGGTTTTAGTTTGATGGTTATTTTGGCCTTGTTC

GAATAATGTTGTTGGGGGTTTGGGGATCAAAAGGGGGAGCATGTGAGCTGGCTCCTCATG

AATTTTGGTTGTGGGCATGTTTGGATGGCCCTCATTGCTATCACACAAAGTGGAGGGAGA

TGACGATAACTGGCACATGGCTTGCACGGAGACAGAGATATTTAGCTAGGGAGTACTAAG

TGTTTGTGTGTAAGTCACAGAGAAAATCTTGGTTGGTTTGGGGATTTGCATTTTGCAAGA

GTGTGTGAGGAGAGGTATTTAAAAGAGATTCTTGGACGTATCTGAATTTATTTATTATGG

AGTATCCGGGACGGCCACTAATGGAAATGGAAAGATAGGGTTGACAGAGTATTGGCGTTT

CCGAGTAG

>**Gsoja_MSTRG.41608.1** GeneID=Gsoja_MSTRG.41608;Strand=-;Pos=Chr09:45513654-45515073,45515411-45515983,45516125-45516465,

CACCAACCCCACATTCAATTCTCCTTTTCCAACAAAATTCCCTCTCCTAATCCTCCACCG

TCCAATTCCCTGCATATTCCCAAGATGATGAGATGTTGCCAAGTGGATGCTCACCTTTAA

CTAGGTTATTTGTAGCCCTTGAGGCAGAATCGTGATGTTCAAGCAGTTTGTATTTTTTTT

TTTTTATCTGTATATAGTTAAAACCCTGGCTAGTATCAGTTAGCTCTCGATACTAATATG

GTGCATTCCATAGATGAGGAGAGAGCCATTGTCTCATCCTTCTACCGAAAATACTGCATC

CTTCTTTCACACCTGCTTTAACGGAATCAATGCAGTATCAGTTCCGTACACTCTTGCATC

TGGGGGCTGGTTAAGCTTGGCTCTTTTGTTTGCTATTGCTGCCGCGACATTTTACACAGG

CGTGTTGATTAAAAGATGAATGGATAAGGATTCGAATATCAGAACCTACCCTGATATGGG

CGAACTTGCGTTTGGAAAGACGGGAAGGCTAATAATATCAGGGTTAATATACACGGAGCT

CTTCTTGGTTTCGGTAGGGTTCTTGATTCTCGAAGGTGATAACTTGAGTAACTTATTTCC

CACTGTGGAGATTCATACAGCTGGCTTAGCAATTGGTGGGAAGAAACTGTTTGTGATATT

GGTTGCCCTTGTCTTGGACAACTTGAGGATACTTTCTTATGTATCTGCAAGTAGGGTATT

TGCTTCTGCTATCATTATTCTGTCAATATCATGGACTGCAACATTTGATGGAGTTGGGTT

TCATCAAAAGGGAACTCTTGTTAATTGGAAGGGAAACCCCACAGCTGTTAGCTTATATGC

CTTTTGCTATTGTGCACATCCTGTGTTCCCCAGCTTGTACAATTCCATGAGAAACAAACA

CCAATTCTCTAATGTACTACTTGTTAGCTTTCTCTTAAGCACTGCTGGTTATGCATCCAT

GGCTATAATTTGTTGCCTAATGTTTGGTCCCAAGGTTGAATCACAGGTCACATTAAACCT

GAAAATAAATAAAGTAAGCCCGAAAATAGCAATATGTACTACCTTGGTCAATCCCATATC

CAAGTTTGCTTTGATGGTAACACCTATTACCAATGCTTTGAAAGACTTGCTTCCAAGGAC

TTACAGGAATAGAGCGACCAGAATCCTGATTAGTACTGTCTTAGTAATCAGAACTACTAC

TGTTGCCCTTGTTGTTCCTTTCTTTGGGTATCTCATGTCCCTTTCTTGAGCGTTTCTAAG

TGTTACAGCTTCTATTATGTTTCCATGCCTTCGTTACTTGAAGATTTCAGGCACTTAGAG

AAAATTTGGGTGTGAGATAGTAGCCATGGTGATAATAATAATAGCACCTATAGCAATGGG

AATAACCGGGACCTACACCTCTCTCATGGAAATAGTGCATCATTTGTAAGGAAAATGTAA

AAATTACACCCAATTTTTTTGGGTGCATGTATATGAAAGGAAGAAATTTTGGCGTGAGAT

AGTCAGCATGTTCGCGTGTTTGGACACACGATGCCAAGGTCATTTGACGTGAAAATCCAC

GTTTGGATAGAAGCTAGTATTAGCATGCACGTTTGGTTCCACGATTTAGATGTGATTTTC

AGAAGCTAACAGTTATATCAAGATAGGTTCTGATGTAGATGAGCTTGCATTTGGAAAGAC

AGGAAGGCTAATAATATCAGGTTAATATACACGGAACTAGCTCTACTTGGTTTTAATAGG

TTCCTTGATTCTGGAAGGTGATAACCCGAGTAACTTATTCCCCAATGTGGATATTCTTAG

TTTCTTGCACTTTGGCTTGACAATTGGTGGGAAGAAACTCTTTGTGATATTGGGTACCCT

TATCATCTTGCCCACTTCGGTTGAACAACTTGAGTTATGAGGTGATGAATGAGTTTTTAA

AAAGAGAAGGGATGAGGTGTGCAGGCTATGGCCAGAGTAGAGAGAGACAGGTATTGTGTG

TGTGATAGAGGTTAGAGAGAATTGTTGGTGAGTGTTAGGTGAGGTCACGGTAGTTGCGGT

GACAGAGAGAAATATTTGATTGGTATGGCATGTGCATGTAATGAGAGACAGAATGTGTGA

GTTTTGTTTAGTTATTTGTGTGTGAAATTGCAGTGCATGCGAGTAATTAGTGGCCGGGGA

GTGTGTGAGAAGAATGGGAAAAAGTTCGTTACACTGATCAATGTAAACCCAATTATGTCC

ATCTTATGATTCTGCAAAAAAAGAAGTTAATTTTATTTTATAAATGATCAATGATAAATA

AATATGATTGTATAAAATTATTATTGTGATCAATATTGATCACACATGATTAAC

>**Gsoja_MSTRG.40293.1** GeneID=Gsoja_MSTRG.40293;Strand=+;Pos=Chr09:38830162-38830941,

CCAATATGCAATACAAAAGCAAACACGATAGATGATGGAATCCCAACTATATAGTATGAC

CCCAGATTGACAAATGCACCAATTTTCTGCCAACCACATCCTCTAGCAGTGCCTGAATTA

TTGTTGACAAAGTGAAAAAAGAAAGTTATACATCATCTATTTCATGCTTATGTGATGTGC

AAGAAGGAAATGTGGGTTCTAAAGAAAATTGGCCACAAAATGAACTAATTCCGTTTAAGT

CTTAACTGTCAGTATGCCTCGCATATTTATGCAATATGCATTGGAGAAATTTAAAACTAA

AGAAAATCAAATCTTACAATTGCTTCACTGAAAGCTTCACATACACTCATTTGTACTATG

CACACATGTTCCTTTCCGGGTATTGGCCCATAGTAACCAGATGTCAATTGACTGTATACT

ATTTGCATTTTTGTATGTGAGATGTATTGGAAGTTCCAATATGCCTCTCAGCCTATACCA

GTGCTTTTGAGTCATAGCTCCTGTGCAGACAAGTAAAAGTAATGGGGGAAAAGAAAAACC

TGAAAGAACACATTGGAGTCCATCCAGAAAGTTGGATGCTGCCAGAATTGGGAACATAGT

TGCTACATATTCGACCACTTCTACTTCATTGCTATATGCATAGCCCCAGATATTGCGTAC

AAGTATCATCACTGTTCCCACTATGATGCCCTCAATAATTGCTAGTACTAAGACAACACG

CACTGCTAAACGTGCATTCCACGGACGACCAGCTCCAAGTTCATTTGAGACACGAATGCT

>Gsoja_MSTRG.61248.1 GeneID=Gsoja_MSTRG.61248;Strand=+;Pos=Chr14:252124-253126,

AGTTTTTGTATCCTTAAAACCGCGGAAAATGCCTTGAAGAGCCAAAGAAAGTACAACAGC

AGGAGCGCCAACAGCTCTTAAAGAGAGAAAGTGTCGTGCGGGAACATATGTTGGATTTTG

CTGATGAAAAGGAGAAGACAATTGATATACTTGGAAGAGGATACAAGAAATATAACGCAT

AAAGTAGAGGGTGAAAACATACTGTAGGAACACCAATTAAATGGAGAAAAGCTCCAGCTC

CCAGGTATAAAGCTAAAGCCTCAAAAAAGCCAAGGGCGAGTGCCAATAGCAGAGCAGTGG

AGACAGAGGAGAGTTGTTGTTTTGTTTTGGCATCTGCAGCGGAGGAAGACTTGGCAATGT

CCTCGGCAACAAAGGAAGTAGCAACACTTAGAAGGGGGATATTAAAAAGCTTAGAAATGA

TGTTAAAGATGGATATGGAAACGCCGGCAGAAGCCAATTCAACGGTGCCAAGTCGACCAA

TGTAGGCGGTTTCCATCAACTGTGCTAAGGGGTCAATTGCCTGACTGGCAAGGGCGGGCA

GAGTCAGCGATATAAGCTCTCGTTTGACATCCACAGAAGCAGAATCTGAATGATGTTGGT

CCTCAGAAGTGTAGGAAGAGGGTTTAGAGCCAAGGGCTATGAACGGCGAGGAGAGACTTG

GGCTATGGCGCAGGAAGTGGTGGTGGGGGTGGTGGCCATAACCGAAACTGATATGGTTGC

GCTTGCGCAGGAAGGGTATTCTGGTGAAGCCTCCATGATATTGAAGTTGGTATTGAGAGG

TGGTGAAATGTATAGCCTCCATGAGCATCAGCGCTGCACTGCAACAAAGTACAAAGTTGA

ATAATCCATCAACATGGAAATAAATATCAAATAGCAGACTCGACTCCACCTGCAGCCCAC

AGAATGTATCGTCGCCCTCTGCAATTGAAATTGTTAGCCGATGATTGCAACGTTTTCCTT

TTATAAATTTCAACAACGCCCATTTGCCAACAATTTTAGTTCC

>**Gsoja_MSTRG.40294.1** GeneID=Gsoja_MSTRG.40294;Strand=+;Pos=Chr09:38831463-38832003,

AAACCATAACAGCTGAAGGAATGGCAAGCCTCACAAATGAGGGAATGTTATGCAGTGCCT

CTTTGGAAAATCCTGTCCAAGATTTTGCACATGAAGGAGAAAACATGACATAGAGTGAGA

GTATAGTAACATTCAACCAATAAGATATAGAATTTGCTACAGCAGCTCCTCTGTTCCCAA

GTCCAGACTTGAAAACTAGAATCCAACATATAAGTACATGTAGTAATGTGGTAATTGCAG

AGCTGCACATCATTGGGAATACAATATTTTGGGTTTGTAGAAATCTGTTGAGGCACAGTA

GAAGGCCATAAGCAAAAAGGCTTGGAAGCATAAACCTAGCATAACTCCCGGCTTCTGCAG

CTATTTCAGGATCCTGGCCAAGGAAAGTTAGAATGGACCTTGTGTTTGCCCAAATAATTG

CAAGCGGAATGCTGACGATCATGAGAGTGAACATGGCTCTCTGCAAGTGTATGCCTAACA

TGTGATACTGCTTTGCTCCATATGACTGGCCACAAAACGTGTCCAAGGAACTTGCCATTC

C

>**Gsoja_MSTRG.57216.1** GeneID=Gsoja_MSTRG.57216;Strand=-;Pos=Chr12:39969695-39970055,

AGATAAGACGGGTTGAATGCCATTGAGGACAAGAGAAAGGGCAAGGAGTGGACAAAGATC

TGAAACTGCAGCAGCAACCTCTTCACCCCCTGTGAAGGCATAGCTAATGACATCTCTTAA

TGCAAGAACCACCAGTGCTGCAATTACTGATATTATGAAAGAAATCACTGTCACCACAAC

CACCGAAAACGATGCTGATTTTGGACTTCTTGCTCCTAGTTCGTTGCTCACCCTCACGCT

GCATTATATATTCTCACAGTATATAATATATAATAATACTTGCAAATTTCACGAAATAAA

TAAATAAAAAACAGGAATCCAATTACTACCTTGCAGCTGCATTGAATCCAACAGAGATCA

T

>**Gsoja_MSTRG.82273.2** GeneID=Gsoja_MSTRG.82273;Strand=-;Pos=Chr19:96440-96462,158281-158457,178085-178100,

TCTAATTATTAATAAGTCCCTCGAATTTTAGGGGGCATGGGGATTGTAATTCTTTCAACT

TCTAGAGGTATAATGACAGATCGAGAGGCTCGATTAGAAAGAATTGGCGGAGAAGTTTTA

TGTTATATATGGTAATCTTTCTAACACCCGAATTATATGAGAAACTTCGATCCATTTTTG

GTAAAAAAAAAAAAAAAAAAAAAAAAAAAAAAAAAA

>**Gsoja_MSTRG.39210.1** GeneID=Gsoja_MSTRG.39210;Strand=-;Pos=Chr09:18276833-18277083,

CTCCACACACCGGCAAACATGCGAAGACTCATGTAGATGGTTAGTGCAATCCATATCCCA

ACGAAACCTTTGCTCTTAGAGAGAAGAAACAATGAAGCAACACTTGCCAATGACACGGTG

ACCTGAACAAATTATGAGCATAGAAATTTGCTAAATAAATTTGATAGAGATGACGAATAC

TTTTTTTTAATAAAAGTTATTGTATGAGAGAACTTACCAAGGAGTATGCAGAATATGCAA

AATCAGATGCC

>**Gsoja_MSTRG.66726.1** GeneID=Gsoja_MSTRG.66726;Strand=+;Pos=Chr15:8376864-8377489,

AAACTGAGATAGAATCAAGAGAGAGTTGAGGGTTATCAAGCAAGCCAGTAATGAGCACGA

GCACCTGAAAATACCAAGTCTCCAAACACAGCATCACCGCGGACGCAGCCGATAGCTTAA

CAAAATCCCAGAGTCCAGAGAATGCCTCAACACTAAACCCACTCCACGTATCCTTAAACT

TTGAAGCACTAACAACATACAAAAACTGTGCCCCAACAATGATCCACCAAGACAAACTCA

GCATCAACGACGAACCCATGATCCCAAACCCTAACTTGTACACCACCACCCAGCTCAGCG

CCACGTGCAGCACCAGCGTGGCAGCGGAGATGTATGTGCTCGGCGCCACCACGCTCTGCG

CCTGCAGAAACTTCTGTATGGGGAAGTTCACTGCGTAAGCGAAGATCTGCGGGATCAGAC

CGTACACGAACATCGCGGCCACTGATGCCACCTCCGGTGGCTCCCCCAGCAAGAGAAGAA

TTGGTTTGCAGAAAATGTACACCACCGTTAAGGGAATCCCTGTTATGGTTAGCACGATAA

TTGCTCTCTGCATGTATATCCCTAGCATTTCATATTTGTTCGCTCCGTACGCTTGTCCAC

ATAAAGTCTCCACCGCACTTCCCATA

>**Gsoja_MSTRG.52475.35** GeneID=Gsoja_MSTRG.52475;Strand=-;Pos=Chr11:45344812-45345072,45345247-45345366,45345415-45345497,

GGAAGGAGCATTTGGAGGACTATAAGCAGGAGATGGTGTTTTTGGAGGAGTAGGAACATT

TGGTGGAGTGTAAATAGGTGAAGGTGTTTTTGGAGGTGTAGGAACATAGGGAGGTTGGTT

ATGAGGGGAAGGAGCATTTGGAGGACTATAAGCAGGAGATGGTGTTTTTGGAGGAGTAGG

AACATTTGGTGGAGTGTAAATAGGTGAAGGTGTTTTGGGAGGAGTGGGAACATTAGGAGG

ATTATAAGAAGGAGAAGGTGTTTTTGGAGGTGTAGGAACATAGGGAGGTTGGTTATGAGG

GGAAGGAGCATTTGGAGGACTATAAGCAGGAGATGGTGTTTTTGGAGGAGTAGGAACATT

TGGTGGAGTGTAAATAGGTGAAGGTGTTTTGGGAGGAGTGGGAACATTAGGAGGATTATA

AGAAGGAGAAGGTGTTTTTGGAGGTGTAGGAACATAGGGTGGAT

>**Gsoja_MSTRG.66724.1** GeneID=Gsoja_MSTRG.66724;Strand=+;Pos=Chr15:8371058-8371331,

CTTAAATTATTTGAAGCATATTATTACTTGCAAAATTTCCAAGCATACATAAGAAAAAAC

AAAACAATAATTCATCAACTTTTCTTTTCTTTTAATTTTCCAGTGAGAACTCGAAATCCG

AACCCACAACTATCTGTTAGGCCAATGGTCAGCAGAAGAACCATTTCAACTCTGAATCTT

AGGTTCCTTCTTGTACCCCCACTTATTTAATCGTTTCTTTGCTGTATTTACCTGCATGCA

TCATAATGTACGATATATGGATGTTGACTTAATT

>**Gsoja_MSTRG.9426.1** GeneID=Gsoja_MSTRG.9426;Strand=+;Pos=Chr03:461641-461920,467578-467757,

CTTGACTTGCAAGTGAATAATGTTACCAAGCACTATTCCAACTGGAATACCTATGAGGTA

ATAACACCCTATGTTCACATATGCTACAATGCTTTGCCACCCTGCTCCAACAGCCACCCC

TATTGAAATCATATAATTTCATGTCGTTCAGACTGTAAAAACAATAGGGTTCAAATCTTA

AACTATTAAAAGTTCTTTAAACCAAGACACCAAGATTTATAAAGCATACCTGAGAGTACA

GGTTGAATACTGTTTAGTAACAAAGAAACTGCTAACAAAGGTGACAAATCCCCCACAGCA

GTAGCCACATCTTCGTTTGAAGTAAAGAGATAAGCTACTTTTTCTCTTAAAATTAAAAAA

AGAACAAATAAGATAAACCCAATTGCAAATGATGTAAGCACTGTCACAACTATAGAGAAC

TTTGCAGCTTTGGAGTTTTCCCTTCCAAGTTCATTTGCCA

>**Gsoja_MSTRG.52475.34** GeneID=Gsoja_MSTRG.52475;Strand=-;Pos=Chr11:45344787-45345006,45345181-45345246,45345421-45345540,

GTGTCTTTGGTGGTGTTGAAACATAGGGAGGTTGGTTATGAGGGGAAGGAGCATTTGGAG

GACTATAAGCAGGAGATGGTGTTTTTGGAGGAGTAGGAACATTTGGTGGAGTGTAAATAG

GTGAAGGTGTTTTGGGAGGAGTGGGAACATTAGGAGGACTATAAATTGGAGAAGGTGTCT

TTGGTGGTGTAGGAACATAGGGAGGTTGGTTATGAGGGGAAGGAGCATTTGGAGGACTAT

AAGCAGGAGATGGTGTTTTTGGAGGAGTAGGAACATTTGGTGGAGTGTAAATAGGTGAAG

GTGTTTTGGGAGGAGTGGGAACATTAGGAGGATTATAAGAAGGAGAAGGTGTTTTTGGAG

GTGTAGGAACATAGGGTGGATTGTTACTAGGGGAAGGAGCTTTTGG

>**Gsoja_MSTRG.26930.1** GeneID=Gsoja_MSTRG.26930;Strand=+;Pos=Chr06:48017638-48017864,

AAAACACTAAACCCAGAAACATTGGTGAGAGAGGTTGCAATAGCAACAGTTGAAAGAGAG

AGCTGGTTAAGATGGCCAACCATTACCAAGGACACCACTTGCAAAAGATATTGCAACACA

CTTGCAACCACCATTGGAGCTGCTATAGTACCCACCTTCTTCAGCTCTTCCCTCATTGCT

CTCCTCCTTTTCATCCATCCCCACTTTCCTGTCTCTGAACCTTCTTC

>**Gsoja_MSTRG.79812.1** GeneID=Gsoja_MSTRG.79812;Strand=+;Pos=Chr18:23000838-23001216,

TAAAGAGAAAATGACTCACCATGACCCCCTTGTCAAGCTTGTACCCTAAAATAAGACCCA

GAGGAATACCGAAAAAGTAGTAACAAGCAATATTCACGTAAGCAACCACAGCCTGCCACC

CTGCGCCAACAGCAACACCTGAGAGAACGGGTTGCACGTTGTTGATGACAATGCAAAGGG

CCAACATGGGCGTGAGCTCTACCACAATCTTTCTCACTTCTGAATCATTTGAAAACAAGA

AAGGGTACTGGTTCCGGAAGATTATCAGCACCATCGAGAGCATGACACCGATCAGAGTTG

AAGTAATCACAGCAACAAGAAGTGAAAACTTTGCTGTTCTTGGGTGACATGCTCCTAGTT

CATTTGACACCCTCACACT

>**Gsoja_MSTRG.52475.11** GeneID=Gsoja_MSTRG.52475;Strand=-;Pos=Chr11:45344092-45345162,45345262-45345336,45345511-45346404,

AAAATTAATTATGAGACGCGATCTCATTTGTCCAAGTAAAAATATTAGAAAAAATATATA

AAAGTGTTCACAATTACTATATACTATAACTCAAGTTCCAAAGAAAGTTTGATTTTTCTA

ATCAATAGTGGTACTTAGAGCAGCACCGACCAGAACTATAACTCAAGTTCCAAAAAAAAG

TTTGATTTTTCTAACCAAGAGTGGTGCTTAGAGCAGCACCGACCAGAATGGTTAGTGAAT

ACACTCCAAACCGCGACGGCGTTATAGACGACGCACCGTTTAGTCTGGCTGGCGTCGTTT

CGTTCGACGACGACGGCGTTTGAGATGATCCCGACGACGACGACGGCGAGGGGGTTGTTG

CCGCCGCAGACGGTAATGGCGGCGATGTTGCCGGGGTGAGGGAGGAAGAGGGTGGGTAAG

TGGGTGGTGCAGTTGAAGCAGAAGGTGGGTAAGATGGAGAGAGTGAGGTTGAGGGAGGGA

TGGTGGATGGATAGGGCGATGATGGGTAGGGTGGTTGAGATGTTGGGGAAGGAGTGTTTG

GAGGTGTTGGATTAATGTAAGGTGGTTGAGATGTTGGGGAATGAGAGTTTGGAGGTGCTG

CAATGGAAGGAGGTTGGGAAGTGGGTGAAGGAGTGGTGTTTGGAGGTGTGGGAATGTAAG

GTGGTTGAGATGTTGGAGATGGGGTTATTGGAACAAAGGGTGGTTGATGGGCAGGGGAAG

GCGTTTTTGGAGGGGTTGGAGTGTAGGGAGGTTGAGAAATAGGAGAAGGAGTGTTTGAGG

GAGTTGGGACATAAGGTTGGTTGCCAGGGGGAGGTGTTTTTGGAGTAGTAGTGGGAACAT

TTGGAGGACTATAAATTGGAGAAGGTGTCTTTGGTGGTGTTGAAACATAGGGAGGTTGGT

TATGAGGGGAAGGAGCATTTGGAGGACTATAAGCAGGAGATGGTGTTTTTGGAGGAGTAG

GAACATTTGGTTGGTTATGAGGGGAAGGAGCATTTGGAGGACTATAAGCAGGAGATGGTG

TTTTTGGAGGAGTAGGAACATTTGGTGGAGTGTAAATAGGTGAAGGTGTTTTGGGAGGAG

TGGGAACATTAGGAGGATTATAAGAAGGAGAAGGTGTTTTTGGAGGTGTAGGAACATAGG

GAGGTTGGTTATGAGGGGAAGGAGCATTTGGAGGACTATAAGCAGGAGATGGTGTTTTTG

GAGGAGTAGGAACATTTGGTGGAGTGTAAATAGGTGAAGGTGTTTTGGGAGGAGTGGGAA

CATTAGGAGGATTATAAGAAGGAGAAGGTGTTTTTGGAGGTGTAGGAACATAGGGTGGAT

TGTTACTAGGGGAAGGAGCTTTTGGGGGAGTGTACACAGGAGAGGGTATTTTTGGAGGCG

TTGGAAAATTAGGAGGAGTATAAACTGGGGAAGGAGTTTGTGGAGGAGTTGGAATATATG

GAGGATGATTAGCAGGTGAAGGTGCTTGAGTGGGAACATTGGGAGGAGTGTATACAGGGG

AAGGAGTTTTTGGAGGTGTTGGAACATTAGGTGGATAATGAATAGGTGAAGGTGGTTTTG

GATGAACATAGGGAGGGCTGGAAATAGGTGAAGGTGTTTTAGGAGGAGGTATTGGAACAT

AGGGTGGATGATAAGGAGTTTCTGGAGGAGTTGGAACAGTTGGAGTTTGAGGAGGTGCTG

GAACATAAGGTGCTCTCGGAGGAGTAATTGGAACATAAGGAGTGTTTGGAGGGGTTGGAA

CATAAGGTGGATTTGGATGATAAGGTGCTTTTGGAGGAGTAATTGGAACGTAAGGAGTGT

TTGGAGGTGTCGGAGAATAATAAGGAGGTGGCTCTCTCACAGCCAACACCACAATAATCA

ATTTCTGACCCTTCTCACAATTATCATCTTTCCCACTAATGAAGTAAAATGGACCCGATC

TATCAAACTGAAACTCCGAGTCACCATCCTCAAACTTCTTGATCGGGTTCTTCTTGTTAC

ATTTCTCATAATCATCATTGTTCACCACCAACACAGTATCTGAGTCCTTCTTGTACTTAA

>**Gsoja_MSTRG.5533.1** GeneID=Gsoja_MSTRG.5533;Strand=+;Pos=Chr02:7842648-7843173,

GGGACAGCAAAGCGAAAGAACTCTCCAACACCAACCAAAGCATTCTTGGAGAAAGAGATG

CGTGTTTTCTCACAAGCAGAGGAATACCTCACAAAGGACAAAAGCATTATCACATTAAAC

CAAACGCAAAGGCTGAAGGAGATTGCGGCACCAACATGTCCCAACCCCAGTTTAAAAACA

AGGGTCCAACATGTTACTACGTGGAAGCACAAAGCTATAGCGGAGGTTAGAATCATGGGA

GAAATCAAACTCTGGGTCTGGAAAAATCGTGTTAGAGGTTTGAGAATTGCGGAACCAAAT

AGAGCAGGTATTAGCCAAATGGCATATTTACGAACTTCAAGGGATATTGTTGGGTCTTGG

CCTAATAGAGTTAATATTTTGTCATTGAAAATCCATAGAATTGTGATCGGGAAACAAACC

AAAGAAAGGGATATTATGGCAGTGTAAGTATATAGTCCAAATTTTTCATATTGCCCTGCC

CCGAAAGCTTGGCCACATAAAGTTTCCAATCCACCAGCCATCCCTG

>**Gsoja_MSTRG.52475.16** GeneID=Gsoja_MSTRG.52475;Strand=-;Pos=Chr11:45344112-45344844,45344941-45345102,45345355-45345366,45345463-45346411,

ACGACATAAAATTAATTATGAGACGCGATCTCATTTGTCCAAGTAAAAATATTAGAAAAA

ATATATAAAAGTGTTCACAATTACTATATACTATAACTCAAGTTCCAAAGAAAGTTTGAT

TTTTCTAATCAATAGTGGTACTTAGAGCAGCACCGACCAGAACTATAACTCAAGTTCCAA

AAAAAAGTTTGATTTTTCTAACCAAGAGTGGTGCTTAGAGCAGCACCGACCAGAATGGTT

AGTGAATACACTCCAAACCGCGACGGCGTTATAGACGACGCACCGTTTAGTCTGGCTGGC

GTCGTTTCGTTCGACGACGACGGCGTTTGAGATGATCCCGACGACGACGACGGCGAGGGG

GTTGTTGCCGCCGCAGACGGTAATGGCGGCGATGTTGCCGGGGTGAGGGAGGAAGAGGGT

GGGTAAGTGGGTGGTGCAGTTGAAGCAGAAGGTGGGTAAGATGGAGAGAGTGAGGTTGAG

GGAGGGATGGTGGATGGATAGGGCGATGATGGGTAGGGTGGTTGAGATGTTGGGGAAGGA

GTGTTTGGAGGTGTTGGATTAATGTAAGGTGGTTGAGATGTTGGGGAATGAGAGTTTGGA

GGTGCTGCAATGGAAGGAGGTTGGGAAGTGGGTGAAGGAGTGGTGTTTGGAGGTGTGGGA

ATGTAAGGTGGTTGAGATGTTGGAGATGGGGTTATTGGAACAAAGGGTGGTTGATGGGCA

GGGGAAGGCGTTTTTGGAGGGGTTGGAGTGTAGGGAGGTTGAGAAATAGGAGAAGGAGTG

TTTGAGGGAGTTGGGACATAAGGTTGGTTGCCAGGGGGAGGTGTTTTTGGAGTAGTAGTG

GGAACATTTGGAGGACTATAAATTGGAGAAGGTGTCTTTGGTGGTGTTGAAACATAGGGA

GGTTGGTTATGAGGGGAAGGAGCATTTGGAGGACTATAAGCAGGAGATGGTGTTTTTGGA

GGAGTAGGAACATTTGGTGGAGTGTAAATAGGTGAAGGTGTTTTGGGAGGAGTGGGAACA

TTAGGAGGATTATAAGAAGGAGAAGGTGTTTTTGGAGGTGTAGGAACATAGGGAGGTTGG

TTATGAGGGGAAGGAGCATTTGGAGGACTATAAGCAGGAGATGGTGTTTTTGGAGGTGTA

GGAACATAGGGTGGATTGTTACTAGGGGAAGGAGCTTTTGGGGGAGTGTACACAGGAGAG

GGTATTTTTGGAGGCGTTGGAAAATTAGGAGGAGTATAAACTGGGGAAGGAGTTTGTGGA

GGAGTTGGAATATATGGAGGATGATTAGCAGGTGAAGGTGCTTGAGTGGGAACATTGGGA

GGAGTGTATACAGGGGAAGGAGTTTTTGGAGGTGTTGGAACATTAGGTGGATAATGAATA

GGTGAAGGTGGTTTTGGATGAACATAGGGAGGGCTGGAAATAGGTGAAGGTGTTTTAGGA

GGAGGTATTGGAACATAGGGTGGATGATAAGGAGTTTCTGGAGGAGTTGGAACAGTTGGA

GTTTGAGGAGGTGCTGGAACATAAGGTGCTCTCGGAGGAGTAATTGGAACATAAGGAGTG

TTTGGAGGGGTTGGAACATAAGGTGGATTTGGATGATAAGGTGCTTTTGGAGGAGTAATT

GGAACGTAAGGAGTGTTTGGAGGTGTCGGAGAATAATAAGGAGGTGGCTCTCTCACAGCC

AACACCACAATAATCAATTTCTGACCCTTCTCACAATTATCATCTTTCCCACTAATGAAG

TAAAATGGACCCGATCTATCAAACTGAAACTCCGAGTCACCATCCTCAAACTTCTTGATC

GGGTTCTTCTTGTTACATTTCTCATAATCATCATTGTTCACCACCAACACAGTATC

>**Gsoja_MSTRG.52475.31** GeneID=Gsoja_MSTRG.52475;Strand=-;Pos=Chr11:45344746-45344832,45345007-45345018,45345067-45345162,45345337-45345354,45345529-45345654,45345862-45346016,

TGCCGGGGTGAGGGAGGAAGAGGGTGGGTAAGTGGGTGGTGCAGTTGAAGCAGAAGGTGG

GTAAGATGGAGAGAGTGAGGTTGAGGGAGGGATGGTGGATGGATAGGGCGATGATGGGTA

GGGTGGTTGAGATGTTGGGGAAGGAGTGTTTGGAGGTTGAGAAATAGGAGAAGGAGTGTT

TGAGGGAGTTGGGACATAAGGTTGGTTGCCAGGGGGAGGTGTTTTTGGAGTAGTAGTGGG

AACATTTGGAGGACTATAAATTGGAGAAGGTGTCTTTGGTGGTGTAGGAACATAGGGAGG

TTGGTTATGAGGGGAAGGAGCATTTGGAGGACTATAAGCAGGAGATGGTGTTTTTGGAGG

AGTAGGAACATTTGGTGGAGTGTAAATAGGTGAAGGTGTTTTTGGAGGTGTAGGAACATA

GGGTGGATTGTTACTAGGGGAAGGAGCTTTTGGGGGAGTGTACACAGGAGAGGGTATTTT

TGGAGGCGTTGGAA

>**Gsoja_MSTRG.82125.1** GeneID=Gsoja_MSTRG.82125;Strand=-;Pos=Chr18:56851923-56852396,

GAGAAAGGGATGCGTGTTTTCTCACAAGCAGAGGAATACCTCACAAAGGACAAAAGCAAC

ATCACATTAAACCAAACACAAAGGCTGAAGGAGATTGCAGCACCAACATGTCCCAACTCC

AATTTAAATACAAGGGTCCAACATGTTGCTCCGTGGAAGCACAAAGCTATGGCAGAGGTT

AGAATCATGGGAGAAATCAAACTCTGGGTCTGGAAAAATCGTGTTAGAGGTTTGAGAATT

GCGGAACCAAATAGAGCAGGTATTAGCCAAATGGCATATTTACGAGCTTCAAGGGAAATT

GTTGGGTCTTGGCCTAAGAGTGTTAATATTTTGTCCATGAAAGTCCATAGAATTGTGATT

GGGAAACAAATCAAAGAAAGGGAGATTACGGCAGTGTAAGTATATTGTCCAAATTTTTCA

TATTGCCCTGCCCCGAAAGCTTGGCCACATAAAGTTTCCAATCCACCAGCCATC

>**Gsoja_MSTRG.82126.1** GeneID=Gsoja_MSTRG.82126;Strand=-;Pos=Chr18:56853611-56854220,

AGACACTCCATTATTACAAACACTACTTGTTTGACATTCCTGGTGTGTTTCTCTATTTTT

CTTGTAACATTATATTATCTATTACATATTTATGTCTTTGCTTTCATTTATTTCTTTGTC

TCTCTCGATGTTGATGTAGGTGTCTCATAACATTTCTTTGCGAAAACCATACTTTCGTGA

CAGATTGTATATCTCTGTACCAACTAGCTTTAAGCACTCGTCATGTGATTTGATTCATTT

TCGTCTGGTGGTTTAGCATCAAATATTCTCTCCCTTGCCATCATTGCCTACAAAAATTGC

ACAAATCCGACACCTAATTAACACCAAACATACAATATTTCTACATTGCAACAAAGAAAT

CATATTGGGTTATTAAGGATAGTGTCAAATTTATAAATCGTGAACCTGTTTCTTCCAGTT

GGTAAGAGCAGTAACAAGGGAAAGGAGAATCGATTGGACAATGGAGCCAGTGACTATTCC

AATCCAGAGGCCCTTTGCTCTTAAATGTGCAACAAAGCCAAGTACGATACCTACAGGAAT

TCCTACCAGATAAAATGCTCCCAGATTGACATAGGCTCCTAGATGTTGCCACCCACTTCC

TCTAGCAACC

>**Gsoja_MSTRG.5528.1** GeneID=Gsoja_MSTRG.5528;Strand=+;Pos=Chr02:7816755-7816954,

CCTGTTTTTCCCAGTTTGTAAATGCTGTCAGCAAAGCCAGTATTATTGTTTGTAGTGTAG

ATCCGGTTAATATTCCAATCCAAAGGCCTTTACCATTGAAGTTAAGACCAAAACCAAACA

AGAGAGACACAGGGATTCCCACGGCATAATATGCTACAAGATTGGTAATTGCCCCTATCT

TCTGCCATCCACTGCCTCTG

>**Gsoja_MSTRG.52475.1** GeneID=Gsoja_MSTRG.52475;Strand=-;Pos=Chr11:45317721-45317734,45344717-45344832,45345007-45345072,45345247-45345336,45345511-45345654,45345862-45346016,

TGCCGGGGTGAGGGAGGAAGAGGGTGGGTAAGTGGGTGGTGCAGTTGAAGCAGAAGGTGG

GTAAGATGGAGAGAGTGAGGTTGAGGGAGGGATGGTGGATGGATAGGGCGATGATGGGTA

GGGTGGTTGAGATGTTGGGGAAGGAGTGTTTGGAGGTTGAGAAATAGGAGAAGGAGTGTT

TGAGGGAGTTGGGACATAAGGTTGGTTGCCAGGGGGAGGTGTTTTTGGAGTAGTAGTGGG

AACATTTGGAGGACTATAAATTGGAGAAGGTGTCTTTGGTGGTGTTGAAACATAGGGAGG

TTGGTTATGAGGGGAAGGAGCATTTGGAGGACTATAAGCAGGAGATGGTGTTTTTGGAGG

AGTAGGAACATTTGGTGGAGTGTAAATAGGTGAAGGTGTTTTGGGAGGAGTGGGAACATT

AGGAGGATTATAAGAAGGAGAAGGTGTTTTTGGAGGTGTAGGAACATAGGGTGGATTGTT
ACTAGGGGAAGGAGCTTTTGGGGGAGTGTACACAGGAGAGGGTATTTTTGGAGGCGTTGG
AAAATTAGGAGGAGTATAAACTGGGGAAGGAGTTCTTGGAGGTGT

>**Gsoja_MSTRG.32263.1** GeneID=Gsoja_MSTRG.32263;Strand=+;Pos=Chr08:2663248-2664830,
CAGGATCAGCACGGGGTGTTGTTGCTTTTGATCAAACAAACGATCAAGACGAGGGAGTCT
CAGTAACAACACCCGTCAACGGCTGTTTCTGTCCGTCCATCACTCCATCTTCGACCAACG
TCAGCAACTGGGCCCGATGGGCTTCAAACTCCCAATCGGTGGTCCCAATCACATACAACA
TAAGACCCGCGCAACACACCTGGGCCGAGAGCAGGCCCAGCCATAGCCCACAGAATCCAA
CGTCAAACCAGAACCCAAGTCCAACTGCCACGGGCATCCCGACCAAATAGAACGCGCCCA
GGTTCACATTCGCCGCCGTGTTCGGCCGCGCCGTCCCTCTAACGACGCCGCACCCCACCG
TCTGCGGACAGTTCCCGAGTTCGCAAATTCCGAGGATCGGCAGCGCCATCGACGTGATCC
TTAATATGTCCTCGTCGGCGGTGAACATCGTCCCCCACCGCCGCCGCATCCCGACGGCGA
AAAACATCGCCGAGAATCCCATAATCGCTGCGAGAAAAACAGAAACCACCGCGGATAGTT
TGGCCCGAGAGGGACGATTGGCTCCCAGCGCGTTCCCGACGCGAGTCGAAACCGCGAAAC
CAAGCGAGGAGGGAAAAACATAGATGAGTGAAGTGATTTGAATGAGTATCCCCATGGAAG
CAACGGTAGCGGTGGGGTCCACGAGAAGCCCGCAAAGAATGATCATGATCTCATACCACC
ACCACTCCAAGCAAACGGAGACGCAGCTAGGCGCAGCGAGCCGAAGGAGCGGCTTCCAGC
CGCTGAGACATTCTCGGCTCGGCGCGGAGCAGTGCAAGCCGGAAAAGAAGACGGCGGCAC
CGAGGAAGAGGAGGATGGAGAGGTTGGAAGCGGCGGAGGCAGCGGCGACACCGGCGAGGC
CGAGGCGGAGGCGCGTGACGAGGAGGTAGTTGAAGGGGAGGTGGAGGAGGGTTCCGGCGA
GGGAAGCTAAGGTGACAGGGTGGGTGACGCCCTGGGCGCGAAGGTAAATGCGGATTGGGT
GGAGGAAGGAGTGGGTGAGGAGGTCGGGGAGGGAGAAGAGGAGGTAGGTGTGGGCCATTA
GGGTGATGTTGGGGTCCTGGTGCAAGAGATCAACGAGGATGTTGGACATGTTGAGCCAGA
GGAGTGATATTGGGATGGAGCAGAGTAACAGGAACATGACGCAGCGGTGGAGGGTCAACG
ACAACACATTCACGCGTTTTGCGCCGAATGCTTGGGAGCATAGCGGTTCCATCCCGAGTG
CTAGACCCGACAGCACCGAATAGCCTGTGATGTTCGCGAACGCCATTCCTAGGGACCCTG
CTGCCAGTTCTAGCTCTCCTAGGTGTCCCAGGAACAGCATCGATACCATGGAACGCGCGT
AGAATATCAGCGCCGTTAAGGCTATCGGAAACGCCAGCTCCCCCACCGCTTTCACTTCTC
TCCTTATGCTCCAACAGTGCTGCCCCTCTTTTTCTACTGCTCCCTCCATCAACGTGTAAT
CTACTACTTCCTCTGTTTTTGATCCCATCTTAATGGATCCTCTCAAACTCAAACTTGCAC

TCTCACTCTCACTCTCCTAAACA

>**Gsoja_MSTRG.52475.21** GeneID=Gsoja_MSTRG.52475;Strand=-;Pos=Chr11:45344733-45344832,45345007-45345162,45345337-45345558,45345862-45345960,

GTGGGTAAGATGGAGAGAGTGAGGTTGAGGGAGGGATGGTGGATGGATAGGGCGATGATG

GGTAGGGTGGTTGAGATGTTGGGGAAGGAGTGTTTGGAGGACTATAAATTGGAGAAGGTG

TCTTTGGTGGTGTTGAAACATAGGGAGGTTGGTTATGAGGGGAAGGAGCATTTGGAGGAC

TATAAGCAGGAGATGGTGTTTTTGGAGGAGTAGGAACATTTGGTGGAGTGTAAATAGGTG

AAGGTGTTTTGGGAGGAGTGGGAACATTAGGAGGATTATAAGAAGGAGAAGGTGTTTTTG

GAGGTGTAGGAACATAGGGAGGTTGGTTATGAGGGGAAGGAGCATTTGGAGGACTATAAG

CAGGAGATGGTGTTTTTGGAGGAGTAGGAACATTTGGTGGAGTGTAAATAGGTGAAGGTG

TTTTGGGAGGAGTGGGAACATTAGGAGGATTATAAGAAGGAGAAGGTGTTTTTGGAGGTG

TAGGAACATAGGGTGGATTGTTACTAGGGGAAGGAGCTTTTGGGGGAGTGTACACAGGAG

AGGGTATTTTTGGAGGCGTTGGAAAATTAGGAGGAGT

>**Gsoja_MSTRG.82124.1** GeneID=Gsoja_MSTRG.82124;Strand=-;Pos=Chr18:56849921-56850147,

CAGAAACATTGGTGAGAGAGATTGCAATAGCAACACTTGAAAGAGAGAGCTGGTTAAGAT

GGCCAACCATTACCAATGACACCACTTGCAAAAGATATTGCAACACACTTGCAACCACCA

TTGGAGCTGCTATAGTACCCACCTTCTTCAGCTCTTCCCTCATTGCTCTCCTCCTTTTCA

TCCATCCCCACTTTCCTGTCTCTGAACCTTCTTCCATCACTGCCTCT

>**Gsoja_MSTRG.73450.1** GeneID=Gsoja_MSTRG.73450;Strand=+;Pos=Chr17:2077621-2078263,

GTACAAAAGAAGGAAGAAAAGGTTGCTAAAATTGGCAACAAAGGATGAGATAGCGATTCC

CGGCACGCCAAGGTTAAGTTTGAAGGTTAGAAAAGTGAGGGTTGGAAGGTGTAGAAGAAT

GGATAGTAAAGTGCACCACAACAGTGGCCAAGTTGTGCCTTTACTACGTAGGAAGATTCG

AAGAGGGTGGAGGAAGCTATTGGCAATGAGGTCTGGAATTGCGAAGCGGCAATAGAGGGT

TGCTACTCGGGTTATGTCTGGATTCTGGCGAAGGCATAGCATGAGAGATTCGAGGTTGAG

CCAAAGAAGAGAAATGGGGAGAGAAAATAGCAGTAACATGAGAATTGTTCTCTGCAAAGT

TAGAGAGAGTAAGGACAAGTTTCGAGAACCGAAGGCTTGGGTGCAGAGGGGTTCCATGCC

CATGGCTAGACCTGAGAGGACAGAGTAACCGGTTATGTTGGTCAAGCCTATGGCCAAAGA

ACCTCCGGCTAATTCCAGGCTTCCCAATCTTCCCATGCACACAACTAAGGTCATGTTTTT

GAGGTAACCCACAAGACTCATGGCTGCTATTGGGAACCCTATGTCTGTCATTCTCCTTAC

TTCTTCCAATACCTGCATTCATGGTAAATAAAATACAGACGTC

>**Gsoja_MSTRG.438.1** GeneID=Gsoja_MSTRG.438;Strand=+;Pos=Chr01:2766060-2766637,

GGTGTGTTTTGCAAGCACTCACCAGAGCATAACAGCAGAAGCAAGAGAGAGGCGAAAGAA

CCCCCAAAGACTCCGAAACGCTTCCCACGAGAACCCGTTCCACGCCGGCCAGCACCACCC

TCCGAAAACATAAACCAACTGGGCCACCACCACGAACCACCACGATCCGTTGAGCACGAC

GGCGGCGCCCACGAGCCCCCACTCCAGCTTCACCATGAGCAGCCAGCTCAGCACCGGGTG

CAGCACCATGGCCATCCCGGCTATCGCCGCGATCACCATCACCTTGCTCTGCGCCTGCAG

GAACTTCGCCACCGGAAAATTCAGCGCGTAAGCGAACAGCTGCGGAATCATCCAAATGGC

GAAGGTCCCCGCAGCCTCCGAAATTTCGGTGTCTTGCCCTATCAACTTCAGAACTTGTCC

CGCGAAGATGTACAAAGGGCACAGTACACAGGCCGTGCTGAGGAGTAACACCCATGATCG

TTGCATGTACACTCCCAGCATGTCCAGCTTCCCTGCCCCCACTGCTTGTCCGCACAGCGT

TTCCAACGCGCTTCCCATCCCTAGCTGCTCGGAATCAC

>**Gsoja_MSTRG.9298.1** GeneID=Gsoja_MSTRG.9298;Strand=-;Pos=Chr02:50466587-50466615,50537508-50538020,

ATCTTGATGTGCATTGGAGTAGATTAGTGTAGAGAAGAGGGGCAGGAGTGGAAATTGCAA

AGAGAACCGGCTGCAGTGTGAGGAAGAGAGAGATGGGGGCAAGAAGTAAATTAAGAATGA

TGGCAGTGATGCATGAGGGGATGAAGGAAATTAGAAGGAGCGTGACCTACATGCCTCGAC

CAGGGGGATGAGGTGGTGGAGCAGGTGATGGAGGCGATGAGGCGTGTGCCGGAGGAGGTG

CTGGATTCGGGTGGTTTGAGGACTCCGGTGGGTGGGGGTGTGAGGGACCTCGATCTCTAC

GCTTCGCGAGAACACCGAGGTCGTTCCTGGCGTCGTCGAGAGCCTCGAGGTTATGTCCAA

GTAATATGCCTTCGAGCAGTTATTAGAGTACTCCCCCCTTTTTGTAATAAATAAAACTTC

ATTTTCTATTGCTATTTTTTCACCTGGGTCGAATCCTCACCCTGTAATAATTCGGCTTAA

TTTTCATGTAATTATGTTTGCTCAACCAAAGAAAAAAAAAAAAAAAAAAAAAAAAAAAAA

AA

>**Gsoja_MSTRG.52475.32** GeneID=Gsoja_MSTRG.52475;Strand=-;Pos=Chr11:45344777-45344898,45345421-45345542,

AGGTGTCTTTGGTGGTGTTGAAACATAGGGAGGTTGGTTATGAGGGGAAGGAGCATTTGG

AGGACTATAAGCAGGAGATGGTGTTTTTGGAGGAGTAGGAACATTTGGTGGAGTGTAAAT

AGGTGAAGGTGTTTTGGGAGGAGTGGGAACATTAGGAGGATTATAAGAAGGAGAAGGTGT

TTTTGGAGGTGTAGGAACATAGGGTGGATTGTTACTAGGGGAAGGAGCTTTTGGGGGAGT

GTAC

>**Gsoja_MSTRG.52475.27** GeneID=Gsoja_MSTRG.52475;Strand=-;Pos=Chr11:45344745-45344832,45345007-45345072,45345247-45345276,45345355-45345366,45345463-45345591,45345635-45345792,45345841-45345929,

AGGGATGGTGGATGGATAGGGCGATGATGGGTAGGGTGGTTGAGATGTTGGGGAAGGAGT

GTTTGGAGGTGTTGGATTAATGTAAGGTGGTTGGGAAGTGGGTGAAGGAGTGGTGTTTGG

AGGTGTGGGAATGTAAGGTGGTTGAGATGTTGGAGATGGGGTTATTGGAACAAAGGGTGG

TTGATGGGCAGGGGAAGGCGTTTTTGGAGGGGTTGGAGTGTAGGGAGGTTGAGAAATAGG

AGAAGGAGTGTTTTTGGAGTAGTAGTGGGAACATTTGGAGGACTATAAATTGGAGAAGGT

GTCTTTGGTGGTGTTGAAACATAGGGAGGTTGGTTATGAGGGGAAGGAGCATTTGGAGGA

CTATAAGCAGGAGATGGTGTTTTTGGAGGAGTAGGAACATTTGGTGGAGTGTAAATAGGT

GAAGGTGTTTTGGGAGGAGTGGGAACATTAGGAGGATTATAAGAAGGAGAAGGTGTTTTT

GGAGGTGTAGGAACATAGGGTGGATTGTTACTAGGGGAAGGAGCTTTTGGGGGAGTGTAC

ACAGGAGAGGGTATTTTTGGAGGCGTTGGAAA

>**Gsoja_MSTRG.417.1** GeneID=Gsoja_MSTRG.417;Strand=-;Pos=Chr01:2599678-2599687,2612473-2612895,2696306-2696317,

AGAAGAAGAAGAAAAAAAGAAACCGCTACTCAACATGACCAGAGGATGAAATAAATCAAA

TAGATCCCGTCCGTCCGTGTGCGGAAAAGAAAGATCAAGGGCGGAAATGCCACCACGTGG

GACGAATCAAAGACAATTGTTCTCATGAAAGATCTTAAGCCACCAAGGCCATGGCTACGA

AGGTTCCAGCGAGTGCAGAGGTAGCAATGAAGGCCTTGCTGACGGAGAATGCAGCGGAGG

GCGGTGGAGGCTCGGCGCCACCGGAGGGTCCGGGTGAGGGTCCGGGTGGAGTGTTAGGAG

AGGTGGGGCTTGGTGTCACTGTAGGAGAAGGTGAAGGAGGAGAGGCTGCAGGAGAGGAAG

CAGGTGTAGGTGCAGCCGTTGGTGGTGCTGGTGCTGGTGTTGGTGTTGCTGCTGGTGGTG

GAGTTGCTGCTGGTGTTTGTTGTTG

>**Gsoja_MSTRG.52475.24** GeneID=Gsoja_MSTRG.52475;Strand=-;Pos=Chr11:45344744-45344832,45345007-45345072,45345247-45345354,45345529-45346458,

TGATAATTATTACAACTCACATTGGTAACCATAGATACACAGATTATACGACATAAAATT

AATTATGAGACGCGATCTCATTTGTCCAAGTAAAAATATTAGAAAAAATATATAAAAGTG

TTCACAATTACTATATACTATAACTCAAGTTCCAAAGAAAGTTTGATTTTTCTAATCAAT

AGTGGTACTTAGAGCAGCACCGACCAGAACTATAACTCAAGTTCCAAAAAAAAGTTTGAT

TTTTCTAACCAAGAGTGGTGCTTAGAGCAGCACCGACCAGAATGGTTAGTGAATACACTC

CAAACCGCGACGGCGTTATAGACGACGCACCGTTTAGTCTGGCTGGCGTCGTTTCGTTCG

ACGACGACGGCGTTTGAGATGATCCCGACGACGACGACGGCGAGGGGGTTGTTGCCGCCG

CAGACGGTAATGGCGGCGATGTTGCCGGGGTGAGGGAGGAAGAGGGTGGGTAAGTGGGTG

GTGCAGTTGAAGCAGAAGGTGGGTAAGATGGAGAGAGTGAGGTTGAGGGAGGGATGGTGG

ATGGATAGGGCGATGATGGGTAGGGTGGTTGAGATGTTGGGGAAGGAGTGTTTGGAGGTG

TTGGATTAATGTAAGGTGGTTGAGATGTTGGGGAATGAGAGTTTGGAGGTGCTGCAATGG

AAGGAGGTTGGGAAGTGGGTGAAGGAGTGGTGTTTGGAGGTGTGGGAATGTAAGGTGGTT

GAGATGTTGGAGATGGGGTTATTGGAACAAAGGGTGGTTGATGGGCAGGGGAAGGCGTTT

TTGGAGGGGTTGGAGTGTAGGGAGGTTGAGAAATAGGAGAAGGAGTGTTTGAGGGAGTTG

GGACATAAGGTTGGTTGCCAGGGGGAGGTGTTTTTGGAGTAGTAGTGGGAACATTTGGAG

GACTATAAATTGGAGAAGGTGTCTTTGGTGGTGTAGGAACATAGGGAGGTTGGTTATGAG

GGGAAGGAGCATTTGGAGGACTATAAGCAGGAGATGGTGTTTTTGGAGGAGTAGGAACAT

TTGGTGGAGTGTAAATAGGTGAAGGTGTTTTGGGAGGAGTGGGAACATTAGGAGGATTAT

AAGAAGGAGAAGGTGTTTTTGGAGGTGTAGGAACATAGGGTGGATTGTTACTAGGGGAAG

GAGCTTTTGGGGGAGTGTACACAGGAGAGGGTATTTTTGGAGGCGTTGGAAAA

>**Gsoja_MSTRG.61707.53** GeneID=Gsoja_MSTRG.61707;Strand=+;Pos=Chr14:2477112-2477356,2477681-2477912,

ATTTGTTTCATTAATTATTATTTGCAACATCAAATGTTTGTTTCACATATATTAGACAAG

TTTTTTTTTTGGAATAAAGCAACTTCAAAGCACCCATCATTTTTATTATACTATTTCTTC

CTTTGCCTTAATCATGAGCTTAATAAAGTTTTCATCCTGAAGTTGTAATTGGTGCTCTTG

GTGGCCCTGAAATTGTAATCGGTGCCCTTGGCGACCCTGCCTTTGGTGGCCCCGAAGTTG

TAATCAGTACCCTTGGGGGCCCTTCCTTTGGTGGCCCCGAAGTTGAAATTGGTGCTCTTG

GTGGCCCTGCCTTTGGTGGTCCTGAAGGTGTAACTGGTGGCTCTGGTTCTGAAGCTGTAA

CTGTGAGAACAAATACAACTAGTAGATGAAAAATTGCAACAAACACTTTCTTCATTTTGT

AAAACGAATTTCTATCTCAATAAACTTTTTAGGATACACTTGTATTTTGATGGGAAT

>**Gsoja_MSTRG.5530.1** GeneID=Gsoja_MSTRG.5530;Strand=+;Pos=Chr02:7820058-7820304,

GTGACATCAGCAAAGGAAGTAGCCAATGCAACCCCTGCAAGGGAGAGTTCACCAAGATGA

CCAGCCATCATCAATGACACCACTTGCAGCAAAAACTGTGACACTGAGACCACCACCATT

GGTGCTGCCATCAAACTCACATTCTTCAACTCTTGGACAAAGCCACTACTACTCTTTGAG

TTTGTTAAGGTCACCCTCTTGTTTTCTTTTGGCAACAATGTCTCCTCCATTTCTTTCTTT

CTTTCTT

>**Gsoja_MSTRG.61707.51** GeneID=Gsoja_MSTRG.61707;Strand=+;Pos=Chr14:2477094-2477299,2477345-2477438,2477763-2477912,

TTGGACAAGAACACCTAAATTTGTTTCATTAATTATTATTTGCAACATCAAATGTTTGTT

TCACATATATTAGACAAGTTTTTTTTTGGAATAAAGCAACTTCAAAGCACCCATCATTT

TTATTATACTATTTCTTCCTTTGCCTTAATCATGAGCTTAATAAAGTTTTCATCCTGAAG

TTGTAATTGGTGCTCTTGGTGGCCCTGAAGTTGTAATCGGTGCCCTTGGTGGCCCTGCCT

TTGGTGGTCCCGAAGTTGTAATTGGTGCTCTTGGTTGCCCTGCCTTTGGTGGTCCTGAAG

GTGTAACTGGTGGCTCTGGTTCTGAAGCTGTAACTGTGAGAACAAATACAACTAGTAGAT

GAAAAATTGCAACAAACACTTTCTTCATTTTGTAAAACGAATTTCTATCTCAATAAACTT

TTTAGGATACACTTGTATTTTGATGGGAAT

>**Gsoja_MSTRG.28718.1** GeneID=Gsoja_MSTRG.28718;Strand=-;Pos=Chr07:9580303-9580542,

TTGAAATGCTGTTTGGATTGAATCTATAAAGGTAGAGCTAGCAACAAGTGGCATCATGGA

AGTCACATATTTGACCACTTCATGTACATTGGTAAAAACACGGCCCCAAACTCTCCATAA

TATCATAAGGGAAGCAAATTCTAAAATCCCCACTGCAGAGGCCAAGAACATGGTGACTTT

AACTGCTAAATATGCAGCTTTTGGACTACCAGCCCCTAATTCATTTGAGATCCTTGTACT

>Gsoja_MSTRG.9427.1 GeneID=Gsoja_MSTRG.9427;Strand=+;Pos=Chr03:462346-463548,

TGTTAATACTGTAAATAATAAAATTTAATCTCAATATGCCAAGAGAGAAGAGCATACTTT

AAGTCTGGCCAATAAAATCAACAACATAAAATAGAAACTCAACATAGATATGCTATAGTG

CAAAGGATTACCATATGGATAGAGCATCAATTTGAACCTCAGCGTTTTTCATATTACCAG

TCAAAAGAATCAATATTGTGTTGTACCAGAGCTCAAGACTGTAAAATATACAACAACTTC

CAAGTTAGGATGCATCTAACTTTAGTAATCCATTCCTAATTTCCCATACAAATTAAGGAA

ATCCAAATTTATTTCACAATCATGGCATTTCTAAGGACCTTAACCCCTTTAGTTGATTGA

GAACAAAGCTCACCATAACATGGCACCAGCTGAAAGGGAAAGCTTGACAACAGGCCAAAG

ATCTTTGAATGCTAAAGAAGAGAAACCTTTCCATGTTTCAGGGCACCAACCGCATGTAAT
AAATATTAGTTGACCAACATTGGGAATCCAATATGCCAAAATTGTTGAAATCATTGCACC
AGGAATCCCATACTTGAATTGTATTGTGAATAGCCAAGAGAGGGACACATGAATGATTAT
TGATAATGTTGCCAAGAATGCAATTATGACATTCTTGCTTTGAGATTGAAGGAATGTCTG
ACAGTTGAATGACACAATATAAGCAAATAAGATTGGAATTGACCAAAGAGAAACGTTTCT
TGCCACTCGTGCTATGCTCTCATCTTGGCCTAAGAGAGTCAAAATTGGGCTTGTGAAGAT
GAACAACGGAAGAAGGCAGAGTGCAGTTAAGAATAAAACTATCGATGATCTTTGAAGATA
CACTCCCATCATGTCATATTCTTTTGCACCGTATGCTTGTCCGCAAAGTGTTGACAACGC
ACTTGACATTCCTAACTACAATCGCCAGTTCACACCCACAAATAATTTGTGTTATTCCTT
GCCTATACTTCTCCACTGAATAAATAGTATTGTTGGCCAATAGCCATATGAAGTTGAATT
ATATATACATATAACATGATGAAAGCTGCACTGATCAACAACAATGTAATTTTGTAATAT
GTTTTTGGCTCATCTTTCTTTTAGAATTTGCAAAGTTCGGATCTTGTTCCAGCAAAAATT
TTATCCATCTTCAACCCTAAATAAATTATAATGTATCATTTTATGATCCGTAGCTAAGGT
TCT

>**Gsoja_MSTRG.46128.1** GeneID=Gsoja_MSTRG.46128;Strand=+;Pos=Chr10:48333319-48334810,
CTTCCCTATACAGCTAAAGACAAATTCCTGATAGATTTCTCTCTCTCCCTTTGTCTTCTT
TGATTTTTCCCCTTTTGGTTTTTGTTCCTGTCCAAAAAAACCGAGATTACTAAAAGTTAA
ACCCACGATTGTTTATCTTCTTCTGAATCAGCTAATGATAATAAAGAATCTTCCTTGATT
TCAGCTTTGGGTGGCTTCTCTGCACCAACCTCCTTGCTGTCATCAACAGCAACAACAACA
CTGGTTAGTTTCTTTGCCCTTAGAGCCTCAACATCCCAATCCGTTTGACTCATAACCACC
AACATGGTCACTGCACAAGACCCCTGAGCCGCAAGTAACCCAAGCCACAACCCTTGAAAA
TCAAGCCCTGCGAAGAAACCAAGCCAAACCGCCACAGGCATTCCAACAAGGTAGAAACAA
CCTAAGTTAATGTTAGCACCAACTTTAGGCCTTGCTGTGCCTCTTAGCACTCCGCACCCC
GTTGTTTGAGGACAGTTTCCGAGTTCGCAGAGTCCTATAATCGGTAACACCAATGAGGTC
AAGGTTATTATTTCCTTGTCTTGTGTGAACATGCTGGCCCAAATGTTCCTAACCAGAATG
GTGAAAACAAAAGCTAAAAAACCTAACATGAAGCTGCAAGAGAGGCCAACTATGGCAGAA
AGTTTAGCCTTTGAGGGCTTTTGAGCACCTAATTTGTTACCAACTCTTGTGGACACGCTG
AAGCTTATTGACGATGGGAGAATGTAGAGCAGAGAAGTGGTTTGGATCAAAATCCCCATG
GAAGCCACGGTTGCTCTAGGATTCACCAACAACCCGCATAACAAAATCATGATCTCATAC
CACCACCATTCTAGGCACACGGAAATGCAGCTTGGGATAGCCAAGTCGAGGAGCGATTTC

CATTGCGTGAAACACTCAAAAGAAAAACCTCCCCATGTTTTCTTATGCGTACCAGAGAAG

ACTATGTAAAGAATCAAAGAAGCTATGAGGTTGAAGTTAGTCCAAACCCCGCTGAGAGCC

ACGCCTTTGATTCCCCAATTGAGGTGGGAAACAAGAAGGTAGTTAATGGGGATGTGAAGG

AGAATTGAGAAAGTGGCGCAGAGAGTGAGAGGCAGAGTAATGGATTGGCTTCGAAGGTAA

ATTCTTAAAGGGTGTAAAAAAGATTGTGCTAAGAGGTCAGGGATGGAATAAAGAAGATAT

GATTGGGCTTGTGTGGCTATGGCCTCGTCCTGGCCACATAAAAGAAGGATATGCTTCATG

TAGAGCCAAAGGAGAGAGATTGGAATGGAAGTGAAGAGGAGCAAAAGAATGGTTCTTTGT

AAGCAGAGGCCGAGGAGGGTGAACTTTTTGGCACCGTAGGCTTGTCCGCAAAAGGGTTCC

ATTCCGACAGCGAGGCCGGAGAGGATCGAGTAACCGGTGATGTTGGCGAAGCCGACGGCG

AGTGAGCCGCCGGCTAAGGCCAGCTCGCCGAGGCGGCCGAGGAAGAGCATGG

>**Gsoja_MSTRG.52475.3** GeneID=Gsoja_MSTRG.52475;Strand=-;Pos=Chr11:45317721-45317734,45344717-45344832,45345007-45345102,45345355-45345366,45345463-45345558,45345862-45345986,

AGTGGGTGGTGCAGTTGAAGCAGAAGGTGGGTAAGATGGAGAGAGTGAGGTTGAGGGAGG

GATGGTGGATGGATAGGGCGATGATGGGTAGGGTGGTTGAGATGTTGGGGAAGGAGTGTT

TGGAGGACTATAAATTGGAGAAGGTGTCTTTGGTGGTGTTGAAACATAGGGAGGTTGGTT

ATGAGGGGAAGGAGCATTTGGAGGACTATAAGCAGGAGATGGTGTTTTTGGAGGAGTAGG

AACATTTGGTGGAGTGTAAATAGGTGAAGGTGTTTTGGGAGGAGTGGGAACATTAGGAGG

ATTATAAGAAGGAGAAGGTGTTTTTGGAGGTGTAGGAACATAGGGTGGATTGTTACTAGG

GGAAGGAGCTTTTGGGGGAGTGTACACAGGAGAGGGTATTTTTGGAGGCGTTGGAAAATT

AGGAGGAGTATAAACTGGGGAAGGAGTTCTTGGAGGTGT

>**Gsoja_MSTRG.52475.4** GeneID=Gsoja_MSTRG.52475;Strand=-;Pos=Chr11:45344082-45345006,45345355-45346448,

TACAACTCACATTGGTAACCATAGATACACAGATTATACGACATAAAATTAATTATGAGA

CGCGATCTCATTTGTCCAAGTAAAAATATTAGAAAAAATATATAAAAGTGTTCACAATTA

CTATATACTATAACTCAAGTTCCAAAGAAAGTTTGATTTTTCTAATCAATAGTGGTACTT

AGAGCAGCACCGACCAGAACTATAACTCAAGTTCCAAAAAAAAAGTTTGATTTTTCTAACC

AAGAGTGGTGCTTAGAGCAGCACCGACCAGAATGGTTAGTGAATACACTCCAAACCGCGA

CGGCGTTATAGACGACGCACCGTTTAGTCTGGCTGGCGTCGTTTCGTTCGACGACGACGG

CGTTTGAGATGATCCCGACGACGACGACGGCGAGGGGGTTGTTGCCGCCGCAGACGGTAA

TGGCGGCGATGTTGCCGGGGTGAGGGAGGAAGAGGGTGGGTAAGTGGGTGGTGCAGTTGA

AGCAGAAGGTGGGTAAGATGGAGAGAGTGAGGTTGAGGGAGGGATGGTGGATGGATAGGG

CGATGATGGGTAGGGTGGTTGAGATGTTGGGGAAGGAGTGTTTGGAGGTGTTGGATTAAT

GTAAGGTGGTTGAGATGTTGGGGAATGAGAGTTTGGAGGTGCTGCAATGGAAGGAGGTTG

GGAAGTGGGTGAAGGAGTGGTGTTTGGAGGTGTGGGAATGTAAGGTGGTTGAGATGTTGG

AGATGGGGTTATTGGAACAAAGGGTGGTTGATGGGCAGGGGAAGGCGTTTTTGGAGGGGT

TGGAGTGTAGGGAGGTTGAGAAATAGGAGAAGGAGTGTTTGAGGGAGTTGGGACATAAGG

TTGGTTGCCAGGGGGAGGTGTTTTTGGAGTAGTAGTGGGAACATTTGGAGGACTATAAAT

TGGAGAAGGTGTCTTTGGTGGTGTTGAAACATAGGGAGGTTGGTTATGAGGGGAAGGAGC

ATTTGGAGGACTATAAGCAGGAGATGGTGTTTTTGGAGGAGTAGGAACATTTGGTGGAGT

GTAAATAGGTGAAGGTGTTTTGGGAGGAGTGGGAACATTAGGAGGATTATAAGAAGGAGA

AGGTGTTTTTGGAGGTGTAGGAACATAGGGAGGTTGGTTATGAGGGGAAGGAGCATTTGG

AGGACTATAAGCAGGAGATGGTGTTTTTGGAGGAGTAGGAACATTTGGTGGAGTGTAAAT

AGGTGAAGGTGTTTTGGGAGGAGTGGGAACATTAGGAGGATTATAAGAAGGAGAAGGTGT

TTTTGGAGGTGTAGGAACATAGGGTGGATTGTTACTAGGGGAAGGAGCTTTTGGGGGAGT

GTACACAGGAGAGGGTATTTTTGGAGGCGTTGGAAAATTAGGAGGAGTATAAACTGGGGA

AGGAGTTTGTGGAGGAGTTGGAATATATGGAGGATGATTAGCAGGTGAAGGTGCTTGAGT

GGGAACATTGGGAGGAGTGTATACAGGGGAAGGAGTTTTTGGAGGTGTTGGAACATTAGG

TGGATAATGAATAGGTGAAGGTGGTTTTGGATGAACATAGGGAGGGCTGGAAATAGGTGA

AGGTGTTTTAGGAGGAGGTATTGGAACATAGGGTGGATGATAAGGAGTTTCTGGAGGAGT

TGGAACAGTTGGAGTTTGAGGAGGTGCTGGAACATAAGGTGCTCTCGGAGGAGTAATTGG

AACATAAGGAGTGTTTGGAGGGGTTGGAACATAAGGTGGATTTGGATGATAAGGTGCTTT

TGGAGGAGTAATTGGAACGTAAGGAGTGTTTGGAGGTGTCGGAGAATAATAAGGAGGTGG

CTCTCTCACAGCCAACACCACAATAATCAATTTCTGACCCTTCTCACAATTATCATCTTT

CCCACTAATGAAGTAAAATGGACCCGATCTATCAAACTGAAACTCCGAGTCACCATCCTC

AAACTTCTTGATCGGGTTCTTCTTGTTACATTTCTCATAATCATCATTGTTCACCACCAA

CACAGTATCTGAGTCCTTCTTGTACTTAAAAACTATCAA

>**Gsoja_MSTRG.66725.1** GeneID=Gsoja_MSTRG.66725;Strand=+;Pos=Chr15:8373481-8373749,

CAGATAACACCGGTTGGATTCCATTAAGAATGAGAGTGACGGCCAAGTAGGGACAGAGGT

CTGAGACCGCATTAGCGACGGTTTCACCATCTGTGAATGCATAGCTTACAACCCGACGCA

GTGCAAGGACCACCACTGCTTCTATCACCGCAATTATGAAAGATATCATATTCACTACAA

TCACTGAGAATGCTGCAGACTTTGGATGCTCAGCTCCCAACTCGTTACTCACCCTCACGC

TGCACTAATTATCAGTTTACAACTATCAC

>**Gsoja_MSTRG.39209.1** GeneID=Gsoja_MSTRG.39209;Strand=-;Pos=Chr09:18275716-18275973,

AAGAAGGGAAGATGTGAGCCACACTTGCAAGCAGGTTTGGAATGCAGCCATAGGAATTGG

ACCAAACCTTGCTGCCAATGAGGCTGCCAAGGTCTGGCAGAATGTAACAGCTATTACTCT

TGCCAACAACAGACCTCCTATTGAAAATGAAAATAAATTTGAACATGAGTAAATTCTAGC

TTGATAAGTGATGAACATATATTTAATTTTTCTTTTTTGGTAAAGACAACAAATATTATG

TATCAGAGACAATTCTGC

>**Gsoja_MSTRG.31243.1** GeneID=Gsoja_MSTRG.31243;Strand=-;Pos=Chr07:44700847-44701086,

TGACAATCTTGCCCTTTCACCTTGCCCCGCTCCAAGTTCATTGCCTACTCTGGTTGACAC

CGATGCACTTAATGCCGTTGGCAAAGTGTACATAAGAGACGTGGTTTGTATCACTATGCC

AGCAGTGGCTAGAGAAACACGAGGGTTATGAAGGTAACCTGCTGAAATTGTCATCAACTC

GTACCACCACCATTCCAAGCAAACAGCTAGACAACTTTGTATCGAAAATTTCATTAGCAC

>**Gsoja_MSTRG.52475.10** GeneID=Gsoja_MSTRG.52475;Strand=-;Pos=Chr11:45344091-45344898,45345073-45345192,45345463-45346448,

TACAACTCACATTGGTAACCATAGATACACAGATTATACGACATAAAATTAATTATGAGA

CGCGATCTCATTTGTCCAAGTAAAAATATTAGAAAAAATATATAAAAGTGTTCACAATTA

CTATATACTATAACTCAAGTTCCAAAGAAAGTTTGATTTTTCTAATCAATAGTGGTACTT

AGAGCAGCACCGACCAGAACTATAACTCAAGTTCCAAAAAAAAGTTTGATTTTTCTAACC

AAGAGTGGTGCTTAGAGCAGCACCGACCAGAATGGTTAGTGAATACACTCCAAACCGCGA

CGGCGTTATAGACGACGCACCGTTTAGTCTGGCTGGCGTCGTTTCGTTCGACGACGACGG

CGTTTGAGATGATCCCGACGACGACGACGGCGAGGGGGTTGTTGCCGCCGCAGACGGTAA

TGGCGGCGATGTTGCCGGGGTGAGGGAGGAAGAGGGTGGGTAAGTGGGTGGTGCAGTTGA

AGCAGAAGGTGGGTAAGATGGAGAGAGTGAGGTTGAGGGAGGGATGGTGGATGGATAGGG

CGATGATGGGTAGGGTGGTTGAGATGTTGGGGAAGGAGTGTTTGGAGGTGTTGGATTAAT

GTAAGGTGGTTGAGATGTTGGGGAATGAGAGTTTGGAGGTGCTGCAATGGAAGGAGGTTG

GGAAGTGGGTGAAGGAGTGGTGTTTGGAGGTGTGGGAATGTAAGGTGGTTGAGATGTTGG

AGATGGGGTTATTGGAACAAAGGGTGGTTGATGGGCAGGGGAAGGCGTTTTTGGAGGGGT

TGGAGTGTAGGGAGGTTGAGAAATAGGAGAAGGAGTGTTTGAGGGAGTTGGGACATAAGG

TTGGTTGCCAGGGGGAGGTGTTTTTGGAGTAGTAGTGGGAACATTTGGAGGACTATAAAT

TGGAGAAGGTGTCTTTGGTGGTGTTGAAACATAGGGAGGTTGGTTATGAGGGGAAGGAGC

ATTTGGAGGACTATAAGCAGGAGATGGTGTCTTTGGTGGTGTAGGAACATAGGGAGGTTG

GTTATGAGGGGAAGGAGCATTTGGAGGACTATAAGCAGGAGATGGTGTTTTTGGAGGAGT

AGGAACATTTGGTGGAGTGTAAATAGGTGAAGGTGTTTTGGGAGGAGTGGGAACATTAGG

AGGATTATAAGAAGGAGAAGGTGTTTTTGGAGGTGTAGGAACATAGGGTGGATTGTTACT

AGGGGAAGGAGCTTTTGGGGGAGTGTACACAGGAGAGGGTATTTTTGGAGGCGTTGGAAA

ATTAGGAGGAGTATAAACTGGGGAAGGAGTTTGTGGAGGAGTTGGAATATATGGAGGATG

ATTAGCAGGTGAAGGTGCTTGAGTGGGAACATTGGGAGGAGTGTATACAGGGGAAGGAGT

TTTTGGAGGTGTTGGAACATTAGGTGGATAATGAATAGGTGAAGGTGGTTTTGGATGAAC

ATAGGGAGGGCTGGAAATAGGTGAAGGTGTTTTAGGAGGAGGTATTGGAACATAGGGTGG

ATGATAAGGAGTTTCTGGAGGAGTTGGAACAGTTGGAGTTTGAGGAGGTGCTGGAACATA

AGGTGCTCTCGGAGGAGTAATTGGAACATAAGGAGTGTTTGGAGGGGTTGGAACATAAGG

TGGATTTGGATGATAAGGTGCTTTTGGAGGAGTAATTGGAACGTAAGGAGTGTTTGGAGG

TGTCGGAGAATAATAAGGAGGTGGCTCTCTCACAGCCAACACCACAATAATCAATTTCTG

ACCCTTCTCACAATTATCATCTTTCCCACTAATGAAGTAAAATGGACCCGATCTATCAAA

CTGAAACTCCGAGTCACCATCCTCAAACTTCTTGATCGGGTTCTTCTTGTTACATTTCTC

ATAATCATCATTGTTCACCACCAACACAGTATCTGAGTCCTTCTTGTACTTAAA

>**Gsoja_MSTRG.26928.1** GeneID=Gsoja_MSTRG.26928;Strand=+;Pos=Chr06:48014148-48014350,

GTTTCTTCCAGTTGGTAAGAGCAGTAACAAGGGAAAGGAGAATCGATTGGACAATGGAGC

CAGTGACTATTCCAATCCAGAGGCCCTTTGCTCTTAAATGTGCAACAAAGCCAAGTACGA

TACCTACAGGAATTCCTACCAGATAAAATGCTCCCAGATTGACATAGGCTCCTAGATGTT

GCCACCCACTTCCTCTAGCAACC

>**Gsoja_MSTRG.28714.1** GeneID=Gsoja_MSTRG.28714;Strand=-;Pos=Chr07:9568723-9570163,

AACAAAATGTTATGAGTAGAGGGGATAGTGTGCCTTAGTTCGAGGCAAGGCTCCTCCTAA

AAGAGAAATGGAATTCTGGAAATAAGCTTTTGCATGGTTTATTCATTATGCATGCAGCCT

TTATATACTGCAGCTTTCTTTACACGTATAACCAACCTAATGAACAGGATTAACAAACTC

TATCAACCTGCAACTGAATTAACTAACTACACGTAACTAATCTGCACGTGGGTTATTATA
CTTGGCTATAGCGTTTTCTTCTTACTAACATTTCTTAGCCCATCAAAAACACCTTGTCCT
CAAGGTGTTGGGTGCAGAAAAAAAAGAACATGAGTAAAACAGAAGCTCAGCCTGTGCCAG
CCATGGTTGTGGTTGAGGCGGTGGCTTCGGTGGATGTGGTGGCGGGTGTGGTGGCGAACG
TTGTCGCCGATGACAGACAGAGCAAATCCAAGTCTTGACGACGAAAAAAATCCAAACCAA
GCGCAGATGAATCTGATGCTTGATGGCATCATCGCCGAGCAAATTGGTGTCATTGCCATA
AGGGATTTCGCGGCAATGGTCGTGGCGGTAAAGACAGTGGGCAGTGCGGAGTGATAGGGG
CAACGAACGACTAAGCTGGAAGTGGTGGTTGGTCGTATGAAAGTGGGGAAGCGGCAGCGG
AGCCATGGGTGCGACTGTTTTGAGTGTGGGATGGTGGGAAACAGCCAAGGGAGGCGGAAT
TGACGTGGGTGCTGCTGTAGGTGGTGGTTGTGCGGAGGAGGACGAAGCTGATGTGGGTGC
TGCTGTAGGTGGTGGTTGTGCGGAAGAGGACGGAGCTGATGTGGGTGTTGTTGTTGTTGG
CGTTATAGGATCCATGGAAGGTGGCATGGTTTGCAGAGTGGTGGTACTAAACAAGCTGAA
CATACGGTGTTGGGTACTCGCAAAATCAATAGCTAGCGGCGGCAGTGGTGGCTGCACCGA
AGAAGGAAGCCATGGCATCCGGGTATCGCACAAGGCTAGTAAGCGGATCCGAGTACTCTC
AAAATCAATCCCAAGGGATTGAGCTGGATGTATATAAGGAGAAGGTGGGAACTGAGACGG
AAGGGATGACTCCTACAGAGGGTGGTGTGTTATGGCATAGCCGGAGTAGGGGTAGAGGCA
GCCATGGAAGACAGTCATGGATGACAGGTCGGACCAATTGTTATGAGTAGAGGGGATAAT
GTGCCTTAGTTCGAGACAAGGCTCCTCCTAAAAGAGAAATGGAATTCTGGAAATAAGCTT
TTGCATGGTTTATTCATTATGCATTCAGCCTTTATATACTGCAGCTTTCTTTACACGTAT
AACCAACCTAACTAACAGGATTAACAAACTCTAACAACCTGCAACTGAATTAACTAACTA
CACGTAACTAATCTGCACGTGGGTTATTATACTTGGCTATAGCGTTTTCTTCTTCCTAAC
A

>**Gsoja_MSTRG.39208.1** GeneID=Gsoja_MSTRG.39208;Strand=-;Pos=Chr09:18274868-18275081,
CTTTTAATTTATGCATTTTCATTCTTTCACACATTCTCTAAAGGCTTCATTTCAAAAGAA
AATATGGATCATTTGAACCTACGAATAACATATAAAGGAGTTGTAGTGTCCTTGAATCCT
CGAAAGATTCCTTGCATGGCCAAGGACAAAAGCACTGCTGGAGCACCTAATGATCTCAAT
CTCAAGTATTTAATAGCAGGGTTTAGCATAGGAG

>**Gsoja_MSTRG.77248.1** GeneID=Gsoja_MSTRG.77248;Strand=+;Pos=Chr17:40638590-40638935,
CATAACATAACAGCTGAAGCCAAAGAAAGCTTTACAAAACCAAACAAGTCCGAAAATGCT

AGCCATGTAAATCCACTCCATGCTCCATCTGACTTAGTGATGAAAATGTACAACAATTGT

GCAATCACAATCACCCACCATGATGTATTGAGAGTAACTGCTGCTCCAATTAGGCCCCAT

CCAAGCTTAAATATAACAAGCCAACTAAAAAATGTGTGCAGCACCAAAACAACTACTGAT

ATCCATAACATCACCAGCACTTTCCTCTGTGCCTGTAGGAACTTTACTATTGGAAAGTTT

ATAGCGTATGCAAACAACTGAGGTATCATCCACACAGCAAACTTTC

>**Gsoja_MSTRG.15163.1** GeneID=Gsoja_MSTRG.15163;Strand=-;Pos=Chr04:8781426-8781677,

GACATAAAAACATGAGAAAAGGCATCCTAATCTTTTCCCCTCATCATCATCACTCTATAC

ATAGATTGTCCCTCTGTTCAGCGTCAACTTCACTATTATCAGTGATTGAAGACGACTCAT

CCAATTTCACATACTTCACAAGCTTATGAATTCATCATATCTTTTTCCACATTATCTATA

AGTTCCTCTTTCAAACCCATTCCGAAACACACCAGTATAGTAGACGCGGAGTTTTATTTG

AGCAACTGCGTC

>**Gsoja_MSTRG.52475.20** GeneID=Gsoja_MSTRG.52475;Strand=-;Pos=Chr11:45344685-45344988,45345262-45345642,45345781-45345909,

GCGATGATGGGTAGGGTGGTTGAGATGTTGGGGAAGGAGTGTTTGGAGGTGTTGGATTAA

TGTAAGGTGGTTGAGATGTTGGGGAATGAGAGTTTGGAGGTGCTGCAATGGAAGGAGGTT

GGGAAGTGGGAGAAGGAGTGTTTGAGGGAGTTGGGACATAAGGTTGGTTGCCAGGGGGAG

GTGTTTTTGGAGTAGTAGTGGGAACATTTGGAGGACTATAAATTGGAGAAGGTGTCTTTG

GTGGTGTTGAAACATAGGGAGGTTGGTTATGAGGGGAAGGAGCATTTGGAGGACTATAAG

CAGGAGATGGTGTTTTTGGAGGAGTAGGAACATTTGGTGGAGTGTAAATAGGTGAAGGTG

TTTTGGGAGGAGTGGGAACATTAGGAGGATTATAAGAAGGAGAAGGTGTTTTTGGAGGTG

TAGGAACATAGGGAGGTTGGTTATGAGGGGAAGGAGCATTTGGAGGACTATAAGCAGGAG

ATGGTGTTTTTGGAGGAGTAGGAACATTTGGTTGGTTATGAGGGGAAGGAGCATTTGGAG

GACTATAAGCAGGAGATGGTGTTTTTGGAGGAGTAGGAACATTTGGTGGAGTGTAAATAG

GTGAAGGTGTTTTGGGAGGAGTGGGAACATTAGGAGGATTATAAGAAGGAGAAGGTGTTT

TTGGAGGTGTAGGAACATAGGGTGGATTGTTACTAGGGGAAGGAGCTTTTGGGGGAGTGT

ACACAGGAGAGGGTATTTTTGGAGGCGTTGGAAAATTAGGAGGAGTATAAACTGGGGAAG

GAGTTTGTGGAGGAGTTGGAATATATGGAGGATG

>**Gsoja_MSTRG.65596.1** GeneID=Gsoja_MSTRG.65596;Strand=-;Pos=Chr15:2791444-2791644,

GTGTGGTAATAACTCTTTGGTTTCAGCATCCTTAGGTGGACCTGTTTCCAAGCATCTACC

CTCCTCTATATGAGGGTTTTCTCCACTAAGGGTATCTTCCTCAGCCACAAAAGAGGTTGT

GACACTGACAAGCGGGAATATTGCGATCCTTGATACTTGATTGAAGAGAGCTATGGAAAC

TCCTACAGCAGCAAGCTCTAC

>**Gsoja_MSTRG.61707.54** GeneID=Gsoja_MSTRG.61707;Strand=+;Pos=Chr14:2477194-2477299,2477345-2477548,2477711-2477905,

CTTCAAAGCACCCATCATTTTTATTATACTATTTCTTCCTTTGCCTTAATCATGAGCTTA

ATAAAGTTTTCATCCTGAAGTTGTAATTGGTGCTCTTGGTGGCCCTGAAGTTGTAATCGG

TGCCCTTGGTGGCCCTGCCTTTGGTGGTCCCGAAGTTGTAATTGGTGCTCTTGGTTGCCC

TGCCTTTGGTGGTCCTGAAGGTGTAACTGGTGGCTTTGGTTCTGAAGCTGTAACTGGTGC

CCTTGGTGGCCCTGCCTTTGGTGGCCCTGAAGTTGTAATCGGTACCCTTGGGGGCCCTTC

CTTTGGTGGTCCCGAAGTTGAAATTGGTGCTCTTGGTGGCCCTGCCTTTGGTGGTCCTGA

AGGTGTAACTGGTGGCTCTGGTTCTGAAGCTGTAACTGTGAGAACAAATACAACTAGTAG

ATGAAAAATTGCAACAAACACTTTCTTCATTTTGTAAAACGAATTTCTATCTCAATAAAC

TTTTTAGGATACACTTGTATTTTGA

>**Gsoja_MSTRG.52475.30** GeneID=Gsoja_MSTRG.52475;Strand=-;Pos=Chr11:45344746-45344832,45345007-45345018,45345067-45345162,45345511-45345654,45345862-45346016,

TGCCGGGGTGAGGGAGGAAGAGGGTGGGTAAGTGGGTGGTGCAGTTGAAGCAGAAGGTGG

GTAAGATGGAGAGAGTGAGGTTGAGGGAGGGATGGTGGATGGATAGGGCGATGATGGGTA

GGGTGGTTGAGATGTTGGGGAAGGAGTGTTTGGAGGTTGAGAAATAGGAGAAGGAGTGTT

TGAGGGAGTTGGGACATAAGGTTGGTTGCCAGGGGGAGGTGTTTTTGGAGTAGTAGTGGG

AACATTTGGAGGACTATAAATTGGAGAAGGTGTCTTTGGTGGTGTTGAAACATAGGGAGG

TTGGTTATGAGGGGAAGGAGCATTTGGAGGACTATAAGCAGGAGATGGTGTTTTTGGAGG

AGTAGGAACATTTGGTGGAGTGTAAATAGGTGAAGGTGTTTTTGGAGGTGTAGGAACATA

GGGTGGATTGTTACTAGGGGAAGGAGCTTTTGGGGGAGTGTACACAGGAGAGGGTATTTT

TGGAGGCGTTGGAA

>**Gsoja_MSTRG.52475.17** GeneID=Gsoja_MSTRG.52475;Strand=-;Pos=Chr11:45344330-45344538,45344811-45344844,45344941-45345072,45345421-45346061,

CGGCGAGGGGGTTGTTGCCGCCGCAGACGGTAATGGCGGCGATGTTGCCGGGGTGAGGGA

GGAAGAGGGTGGGTAAGTGGGTGGTGCAGTTGAAGCAGAAGGTGGGTAAGATGGAGAGAG

TGAGGTTGAGGGAGGGATGGTGGATGGATAGGGCGATGATGGGTAGGGTGGTTGAGATGT

TGGGGAAGGAGTGTTTGGAGGTGTTGGATTAATGTAAGGTGGTTGAGATGTTGGGGAATG

AGAGTTTGGAGGTGCTGCAATGGAAGGAGGTTGGGAAGTGGGTGAAGGAGTGGTGTTTGG

AGGTGTGGGAATGTAAGGTGGTTGAGATGTTGGAGATGGGGTTATTGGAACAAAGGGTGG

TTGATGGGCAGGGGAAGGCGTTTTTGGAGGGGTTGGAGTGTAGGGAGGTTGAGAAATAGG

AGAAGGAGTGTTTGAGGGAGTTGGGACATAAGGTTGGTTGCCAGGGGGAGGTGTTTTTGG

AGTAGTAGTGGGAACATTTGGAGGACTATAAATTGGAGAAGGTGTCTTTGGTGGTGTTGA

AACATAGGGAGGTTGGTTATGAGGGGAAGGAGCATTTGGAGGACTATAAGCAGGAGATGG

TGTTTTTGGAGGAGTAGGAACATTTGGTGGAGTGTAAATAGGTGAAGGTGTTTTGGGAGG

AGTGGGAACATTAGGAGGATTATAAGAAGGAGAAGGTGTTTTTGGAGGTGTAGGAACATA

GGGAGGTTGGTTATGAGGGGAAGGAGCATTTGGAGGACTATAAGCAGGAGATGGTGTTTT

TGGAGGTGTAGGAACATAGGGTGGATTGTGTTTTAGGAGGAGGTATTGGAACATAGGGTG

GATGATAAGGAGTTTCTGGAGGAGTTGGAACAGTTGGAGTTTGAGGAGGTGCTGGAACAT

AAGGTGCTCTCGGAGGAGTAATTGGAACATAAGGAGTGTTTGGAGGGGTTGGAACATAAG

GTGGATTTGGATGATAAGGTGCTTTTGGAGGAGTAATTGGAACGTAAGGAGTGTTT

>**Gsoja_MSTRG.9429.1** GeneID=Gsoja_MSTRG.9429;Strand=+;Pos=Chr03:485756-486027,

GGAACATTTAATACTTTAGCAACTACTTATATAATTTTATTAATTTAAGAAATATTTGAG

AGAGAATATACTGATAGGATGGTACTATAGTATAATAACTATCAAGCAATCCCCTGAATG

TATAACGTACAATAGCCAAACAAACAATGATGATATTGATAAATAGGAGTCTGAAACTAA

TTATCTGATGTACTTGTTTCATGATCAAGTTCCACCTTATACCACTTACTAATACGATTA

CGGGCTATAGTTACCTGCACATTGACATAACG

>**Gsoja_MSTRG.28713.1** GeneID=Gsoja_MSTRG.28713;Strand=-;Pos=Chr07:9568099-9568405,

GTTATGATGCATAAGGTTCTTAGAAACTATGAGGAAAAGATAATTTTAGTGCAACTTTTG

GTATCACTTTACTCAATACCTGATAATGCTGTTTGAATTGAATCTACAAAGACAGAGCTT

GCAAGAATTGGTGTCATGGATGCCACATAACGGATTACTTCAGGTATATTGGTAAAAATA

TGCCCCCAAATGTTTCTTGTGACCATGAGAAGAATAAATCCTAAAGCCCCAACCACGAAG

GACATTAACAAGGTGACTTTAACCGCTAAATATGCAGCTTTTGGACAGCCAGCCCCTAGT

TCATTTG

>**Gsoja_MSTRG.52475.14** GeneID=Gsoja_MSTRG.52475;Strand=-;Pos=Chr11:45344112-45344832,45345007-45345162,45345337-45345975,45346127-45346411,

ACGACATAAAATTAATTATGAGACGCGATCTCATTTGTCCAAGTAAAAATATTAGAAAAA

ATATATAAAAGTGTTCACAATTACTATATACTATAACTCAAGTTCCAAAGAAAGTTTGAT

TTTTCTAATCAATAGTGGTACTTAGAGCAGCACCGACCAGAACTATAACTCAAGTTCCAA

AAAAAAGTTTGATTTTTCTAACCAAGAGTGGTGCTTAGAGCAGCACCGACCAGAATGGTT

AGTGAATACACTCCAAACCGCGACGGCGTTATAGACGACGCACCGCAGTTGAAGCAGAAG

GTGGGTAAGATGGAGAGAGTGAGGTTGAGGGAGGGATGGTGGATGGATAGGGCGATGATG

GGTAGGGTGGTTGAGATGTTGGGGAAGGAGTGTTTGGAGGTGTTGGATTAATGTAAGGTG

GTTGAGATGTTGGGGAATGAGAGTTTGGAGGTGCTGCAATGGAAGGAGGTTGGGAAGTGG

GTGAAGGAGTGGTGTTTGGAGGTGTGGGAATGTAAGGTGGTTGAGATGTTGGAGATGGGG

TTATTGGAACAAAGGGTGGTTGATGGGCAGGGGAAGGCGTTTTTGGAGGGGTTGGAGTGT

AGGGAGGTTGAGAAATAGGAGAAGGAGTGTTTGAGGGAGTTGGGACATAAGGTTGGTTGC

CAGGGGGAGGTGTTTTTGGAGTAGTAGTGGGAACATTTGGAGGACTATAAATTGGAGAAG

GTGTCTTTGGTGGTGTTGAAACATAGGGAGGTTGGTTATGAGGGGAAGGAGCATTTGGAG

GACTATAAGCAGGAGATGGTGTTTTTGGAGGAGTAGGAACATTTGGTGGAGTGTAAATAG

GTGAAGGTGTTTTGGGAGGAGTGGGAACATTAGGAGGATTATAAGAAGGAGAAGGTGTTT

TTGGAGGTGTAGGAACATAGGGAGGTTGGTTATGAGGGGAAGGAGCATTTGGAGGACTAT

AAGCAGGAGATGGTGTTTTTGGAGGAGTAGGAACATTTGGTGGAGTGTAAATAGGTGAAG

GTGTTTTGGGAGGAGTGGGAACATTAGGAGGATTATAAGAAGGAGAAGGTGTTTTTGGAG

GTGTAGGAACATAGGGTGGATTGTTACTAGGGGAAGGAGCTTTTGGGGGAGTGTACACAG

GAGAGGGTATTTTTGGAGGCGTTGGAAAATTAGGAGGAGTATAAACTGGGGAAGGAGTTT

GTGGAGGAGTTGGAATATATGGAGGATGATTAGCAGGTGAAGGTGCTTGAGTGGGAACAT

TGGGAGGAGTGTATACAGGGGAAGGAGTTTTTGGAGGTGTTGGAACATTAGGTGGATAAT

GAATAGGTGAAGGTGGTTTTGGATGAACATAGGGAGGGCTGGAAATAGGTGAAGGTGTTT

TAGGAGGAGGTATTGGAACATAGGGTGGATGATAAGGAGTTTCTGGAGGAGTTGGAACAG

TTGGAGTTTGAGGAGGTGCTGGAACATAAGGTGCTCTCGGAGGAGTAATTGGAACATAAG

GAGTGTTTGGAGGGGTTGGAACATAAGGTGGATTTGGATGATAAGGTGCTTTTGGAGGAG

TAATTGGAACGTAAGGAGTGTTTGGAGGTGTCGGAGAATAATAAGGAGGTGGCTCTCTCA

CAGCCAACACCACAATAATCAATTTCTGACCCTTCTCACAATTATCATCTTTCCCACTAA

TGAAGTAAAATGGACCCGATCTATCAAACTGAAACTCCGAGTCACCATCCTCAAACTTCT

TGATCGGGTTCTTCTTGTTACATTTCTCATAATCATCATTGTTCACCACCAACACAGTAT

C

>**Gsoja_MSTRG.5531.1** GeneID=Gsoja_MSTRG.5531;Strand=+;Pos=Chr02:7840680-7841468,

CCAAATTAGAATAATATAATAAAACAGTCATGCATGGACACTCCATTATTACAAACACTC

CATTATTTTTCTTGTAACATTTATATTATCTGTTACTTATTTCTTTGCTTTCATTTATTT

CTTTGGCTCTCTAGATGTTGACGTAGGTGTCTCGTAACATTTCTTTGCGAAACCATAGTT

TGGTGACTTATTTCCTACGGAAAATCAAAATTATATATCTATGTCTATGTGAGACACAGC

TGACCAAACTAGCTTTAAGCACTCGTCATGTGATATGATCCATTTACGTCTGGTGGTTTA

GCATCAAATACTCTTTCCCTTGCCATCATTGCCTACAAAAATTGCACAAATCCGACACCT

AATTAACACCAAACATACAATATTTCTACATTGCAAAAAAGAAAGCATATTGGATAATTA

AGGATAGTGTCAAATTTATCAATTGTGAACCTGTTTTTTCCAGTTGGTAAGAGCAGTAAT

AAGGGAAAGGAGAATCGATTGGACAATGGAGCCAGTGACAATTCCAATCCAGAGGCCCTT

TGCTCTGAAATGTGCAACAAAGCCAAGTAGGATACCCACAGGAATTCCTACCAGATAAAA

CGCTCCCAGATTGACATAGGCTCCTAGATGTTGCCACCCACTTCCTCTAGCAACCCCTGA

TTATTTTATCTATTTACTTATTTATTTTCGTTGCAGGTTAGCAAAATAATAATGATAGTT

ACTGACAAAATATAACACACCAAATGCATTGGTAACACATTTTCTAACGTTATCTTTTTA

ACAAACCCG

>**Gsoja_MSTRG.61707.48** GeneID=Gsoja_MSTRG.61707;Strand=+;Pos=Chr14:2472615-2472625,2477172-2477438,2477763-2477906,

GACAATTTTTTTTTTTTTTTTGGAATAAAGCAACTTCAAAGCACCCATCATTTTTATTAT

ACTATTTCTTCCTTTGCCTTAATCATGAGCTTAATAAAGTTTTCATCCTGAAGTTGTAAT

TGGTGCTCTTGGTGGCCCTGAAATTGTAATCGGTGCCCTTGGCGACCCTGCCTTTGGTGG

CCCCGAAGTTGTAATCGGTGCCCTTGGTGGCCCTGCCTTTGGTGGTCCCGAAGTTGTAAT

TGGTGCTCTTGGTTGCCCTGCCTTTGGTGGTCCTGAAGGTGTAACTGGTGGCTCTGGTTC

TGAAGCTGTAACTGTGAGAACAAATACAACTAGTAGATGAAAAATTGCAACAAACACTTT

CTTCATTTTGTAAAACGAATTTCTATCTCAATAAACTTTTTAGGATACACTTGTATTTTG

AT

>**Gsoja_MSTRG.9425.1** GeneID=Gsoja_MSTRG.9425;Strand=+;Pos=Chr03:460834-461427,

TTCTTTGAATAAGACTAAAAATTATCCGGGAAGTATTATATCATTTATCCTAAAGTACTT

CTTAAACTAATTAAACTATATTAGTCTATAAAGTATTACAAATCATGCTACATAGTTTTA

CACCTTTGAATAAGACTAACAATTATCCGGGAAGTATTATACCATTTATCCTAAAATACT

AATTAAACTATATTAGTCTTTAAAGTATTACAAATCATGCTACATAGTTTCTCAAGTATT

GAAAAATTCTTCAAATTAGTCTCTCAATGCTAGTAAAATATATCAATCCAGAGACTAACT

TAAGGAACATTCAATACTTTAAGAACTACTTGTATAATTTATAATTTAATTAATTTAAGG

ACTATTTGGGAGACTACATTGATAGTTTATTCAAATACCTATCAAGCAATCTCTTGGCTA

TATAACACCGGCTAAACAAATAAGGACGATATAGATTAGAGTCTGAAACTAATTATCTGA

TGTAACTGTTTCACGGTCAAGGTCCACCTTAGACCACTTACTAATACGATTACGGGCTAT

AATTACCTGCACAATGACAATGATTTCATCAAGTTCTATATCAAATATCTAATA

>**Gsoja_MSTRG.52475.26** GeneID=Gsoja_MSTRG.52475;Strand=-;Pos=Chr11:45344745-45344832,45345007-45345072,45345247-45345336,45345511-45345591,45345635-45345792,45345841-45345929,

AGGGATGGTGGATGGATAGGGCGATGATGGGTAGGGTGGTTGAGATGTTGGGGAAGGAGT

GTTTGGAGGTGTTGGATTAATGTAAGGTGGTTGGGAAGTGGGTGAAGGAGTGGTGTTTGG

AGGTGTGGGAATGTAAGGTGGTTGAGATGTTGGAGATGGGGTTATTGGAACAAAGGGTGG

TTGATGGGCAGGGGAAGGCGTTTTTGGAGGGGTTGGAGTGTAGGGAGGTTGAGAAATAGG

AGAAGGAGTGTTTTTGGAGTAGTAGTGGGAACATTTGGAGGACTATAAATTGGAGAAGGT

GTCTTTGGTGGTGTTGAAACATAGGGAGGTTGGTTATGAGGGGAAGGAGCATTTGGAGGA

CTATAAGCAGGAGATGGTGTTTTTGGAGGAGTAGGAACATTTGGTGGAGTGTAAATAGGT

GAAGGTGTTTTGGGAGGAGTGGGAACATTAGGAGGATTATAAGAAGGAGAAGGTGTTTTT

GGAGGTGTAGGAACATAGGGTGGATTGTTACTAGGGGAAGGAGCTTTTGGGGGAGTGTAC

ACAGGAGAGGGTATTTTTGGAGGCGTTGGAAA

>**Gsoja_MSTRG.84397.1** GeneID=Gsoja_MSTRG.84397;Strand=-;Pos=Chr19:37788756-37789093,37791007-37792862,

ATGACGTGATATAAAGAGATAGAAAGAAATAAAAAAATCTGACCAAGCTTCGAATTACAA

ACGAATCCGATTTTACAGAAGTTATGACGTGATATAAAGAGATAGATAGAAAGTTATCAA

CTCTTGAAGACTCAAGCTTCGAATTACAAACGAATCCGATTTTTTACTCCAACAAAATCT

GACCAAAGCAGATTCCAGCACTCATTACGTGATTAATCAAATCAAACTAGTGGGACGGAA

TTAACTTCTGTATGGGTACGTGACACCTTAATACTGATTTTTGTTTTTATAGATTAATAT

TAGTTATTAATTTGTTTTGTTAGTAAAAGAGATTCAAATCAATCACCTCTCTCTCCTCTC

TTCACCACCATACCAAATCTTATAACCCCTCCTAACACACTTATAAACTCCTTGATATTA

TTCAGAATAAAAGCATATCTATGAGATGAGGGAAAAATACTTATTGATTAACTCCACACT

AACCACAGAATCTGATTCCAGCCACACTTTCTTCAGATTCCGAATCCAGGCTATGGTATG

TGTAAGAACTGGTAATTGGTGACAAAATTGTATTTATCATATTCATTCATTGCCTGAGTT

TAATATATGAACAACTTCTTCATAGTTAAAAACAGAGAGCGCTTGGCCTAGGAAAATTAT

TAAAATTAGAGAGTAAGAAACAAGATAACATACCAGAATACCATTGGCAAAGCGTATGAT

GACAGTGAACACAAGAGCATAAGCAGCGAGTTCCCTTGAACCAATATGACCAATAAATGC

TTGGCTTATAACACTGATGCCAAAGGTGGTGAACCTTGTGAATATGGCTGGTGCTGCCAC

TATCCACATCACCTTGCTCTCTTCCCACACCCTCTTCACCAATGATAATTTCTCTTCTTC

TTCTTCTTCTTCTGATTTTTTTTCTCTGCTCAACAGCTTCTTCTCTAGATTCCCCTCCAT

TTTTTGTGTCTTATGTCGTTGTTGGGGACTCTTAAGATCGATCAGAGACTTAACAGTTCA

CAAGGGAGTTTGGGGCTGATATTAAAACAGAAAATAAAAAAAAGAATGAGTATATATCTT

ATCGTTGCTCTTGCACGAGAGGAACAGCAACTAGACGCACACTATACACAAGAACTTCAA

TTTATATGCACCAGGAGGGGTACTAAGTGTAACATCCCATTTTTTTCATAAATAAATTTA

ATAATTTTTTTTAGTAAAAATAATAAAAATAAATAAATAGAGTAAATAATAGGTCGTAAA

TGCCCCTAACTATAAATAGCGCTAGGTTTTTCAGATTGACTCCTCAGCCTCCTCTGTCTC

TCATTTTCGTTTTTCCCCTTCTCCTCTCAAAACCCTTTCTTTTTCCCGTCGCCCACAAAA

CCTGTCTCAGAAAAATGATGATCTCGGACTCATTCACCGTTGGATTGTCGTGAAATTTGA

GCACCACGTTCGCAACCCAATTTCGAGAATTCTCACCGTTGGGAATTGAAAAATCATGTC

TGGGCTTAGAGGAAAACCCTTCGCATTGTAGCTTTTTATTTTTCCGCAGAAACCCAAAAC

TGTCTCGGTAAAACTACGATCCCGATTTCGTTAACCGTTGGATTTTCATGAAATTTGGAT

ATGTTGCTCGAAATTAAATTTCTCACACTCTCACCGTTGGGATTTGCGAGATAATATTCT

TGGTGGGAGAAAAAGGAACCGCATGAAGACAGTACAAGCGGAGGCTTCAATCTCTTCTCC

GTCTCTCTGACGTTCGAGAATTCTATCGGAGCAGTCGGAGGAAAAACTTGAGGAATCTCA

GGAAACCGCTATAGATGTCTCTATCGTTGTCGGAAGACACGTGAGTCCACTTAGAGACGA

CTAGGTGAATTGATTTAAGGAACCTTTTGCTGAAGGACGTCGAGACACAACGCTCTGATA

GGATGTGACATTGGGGTATAGGGTTTTATATTAATTGTATGAAGTCTTAAACGGTCTTGC

TTAAACCGAGATAACTTTATTAGAAATTTGGACAAGTTTGAATATGATGTAGAAGAAAGT

GAATGTGAGCCTTTTATCCCTTTGAAAAACTTGTATTTACACATGTAAAAAAAAAATACT

ATTAATTAATATTTGGATTTTTATTCCTTTATCAGTATATATGTGAGAGATAGAGGGTGT

CACAGTAAGAGAATCTTTTATCACATATATTATT

>**Gsoja_MSTRG.57214.1** GeneID=Gsoja_MSTRG.57214;Strand=-;Pos=Chr12:39966271-39966421,39967073-39968662,

ATTTTTTCAAACAAAGAATGAAAATAGAATGTTTCTATCATATTTTTTTCATTCTCTATT

TTCTCCGAACAAACAATGGCACAAACTAAAGCAGCTTATTGTACTTGAATGGTGATTATT

TCACACCTCATTTTGGGGTATATGCTGCTTAAATTAAATAAGAAATGCTGAAAAGCTAGA

TATAATGGGGAAAAAAAGTGTTAATTTTGATATATTTTGAATCTGGAGAAGGTTGTATAT

TTTTATATATGAATGGATTTGAAATTTCGATGAGAGATGCAAAGTAAGAAATAAAAGAAG

TTACCAGATAGAAAGGGAATCAAGAGCCAACTCAGGATTAGGGAGAAGCCCCGCGAGCAA

AACCAGAATCTGAAAGTACCATGTCTCCAGGCACAGCATGACCGCGGACGCAGCGGAGAG

CTTGAAGAACCCATACAAACCGCTAAACGCCTCCCACGTGAACCCCTGCCACGTGCGCCT

GCACCTCTCGCTCTTCACTATGTACACGTACTGTCCAATCACCATTATCCACCACGACAG

GCTCAGCACCAGCGACGCCCCCAACAGCCCCAACCCAATCTCGTACACCGCCACCCAACT

CATCCCCAGGTGGACCACCAGCGTCGCCGCTGATATGTACGCGCTCGGTGCCACTATGCT

CTGCGCTTGGAGAAACTTCTGGATCGGGAAGTTCGCCGCGTATGCGAATATCTGTGGGAT

CAACCCGTACACAAAGAGTGCTGCCGCCGACGCGATTCTCGGTGATTCCCCGAGGAATAT

TAGCATCGGTTCGCTGAATACGTATATCACGGTCAACACTACGCCGGCTAGTGAGAGAAG

AATCGTTGACCGCTGCATGTAGACTCCTAACATCCCGTACTTTTGAGCTCCGAATGCTTG

TCCGCATAGCGTCTCCACGGCACTCCCCATACCCAACTGCAAGTAATATATATAATTTTG

TGTTTTTCAATAATTACGCGCAATTATCAGCAGAGATTCACTTGAAAAGAAAATGGTGGT

ACCATGAGGCCGTAGGCGAACATTTGGATGCCGGTGTTGCCGAGGGAGGCGGCGGCGAGT

TCGAGGTTGCCGAGGTGGCCGGAGAAGATTTGGGTGGACATGGACATGAGGTAGTTGATG

AGGTAGACGATGACGGCAGGGGCGGCGAGGAAGAAGAGGAGCTTCAACTCGATCCACGTG

GCGGGTCCCACACGGTTGAGGAAGGGTAGGGTGTCGTCGGAGAGAATTCTCTCAAGTTGC

CCATGGGAGGAGGACTCGTGCTTTGAGTTTGATCGAGTTCCCTCCGCTGTTAACAGTGGC

TCTTTCTCCGCTGAACCCATTGATCTCGATCCGAATTCACGGCAAAGAAGGCTTTCTCTC

CTGTAACGTTTGGAGCAGCAGCAATCTCGCCGCCACAAGTCCCAACTCACCTACCACGAG

GAGCCGTAAGGCACACCATCTCTTATATATACACATATAACGAGAATATTATATTATATT

ATATTCAACAACCACTAGGAGGCGGAACTCACTGTAATTATAACTGATTTGAAACGTGTT

TGGGTGGGATAATCGTAGAAATGCTAGCTTTTTCGTTATGAAAATTACTAGAATATATGT

GTTGATTTTTTATCCAAGTCAATCTCATTTTTACAAGAAAAAAAAAAAAACAGAATGTTA

ATTTTTTTTTTCAGCCAAAAGAAAAGCTTTTAATAAATGAAGGTACAGGGTACCAATCCG

T

>**Gsoja_MSTRG.82273.1** GeneID=Gsoja_MSTRG.82273;Strand=-;Pos=Chr19:77385-77406,96440-96460,158277-158457,

TCCCTCGAATTTTAGGGGGCATGGGGATTGTAATTCTTTCAACTTCTAGAGGTATAATGA

CAGATCGAGAGGCTCGATTAGAAAGAATTGGCGGAGAAGTTTTATGTTATATATGGTAAT

CTTTCTAACACCCGAATTATATGAGAAACTTCGATCCATTTTTGGTAAAAAAAAAAAGAA

AAAAAAAAAAAAAAAAAAAAAAAAAAAAAAAAAAAAAAAAA

>**Gsoja_MSTRG.32570.1** GeneID=Gsoja_MSTRG.32570;Strand=-;Pos=Chr08:3998727-3999590,

ACAAGATCTAGTTAAGAGCATCACATTAGCATCATTTTGCAAGGGAATTTACCATACCAG

TGCATAACAGCTGAAGGAGCAGCGAGTTTAAGGAAATCAAGAAGGTTGTGCAGGGCCATC

TTGGAAAAACCTGTCCAAGAGTGTTTGCATGCAGAAGAGAACCTCACATACAGAGAAATC

AAGATTACATTTATCCAATACGATATGGAATTGGCCAAGGCTGCTCCTTTGATTCCAAGC

CCTGACTTGAAAACCAAAAGCCAACACAAAAGGACATGAAGCACAGCTGCAATTCCAGAA

GTTAGCACCATTGGGAACACAATCTTTTGGGTTTGCAAGAACTTGAGAATGCACTGAAGA

AGACCGTATGCAAATAAACTCGGGATCATGTACTGGGTGTATGAGCCGGCTTCCTTGGAT

ATGGCTTTGTCTTGGTGCATAGCCTTGAGAATTGGTTCTGTGTTGTACCAGATGATCGAA

AGGAACACACTCATCATTAAAACAACAAGCATGGATCTCTGCATGTGTATTCCAAGCATA

TGGTATTGTCCTGCTCCATTTGATTGACCACAAAATGTGTCCAGTGCAGTAGCTAGCCCC

AACAATAAGTTGAAGCCGGTGGCCGATGCGAAGGAAGAGGCCATGGAAGCGCCGGAAAGA

GGGAGGGTACCAAGATGGCCAATAAACATGACAGAGATGGCTTGAAGAATGTATTGTAAA

ATTCCAACTGAGAAGAGAGGCACTGCTAACCATAGCAGCCTCTTCACTTCTTCCACCACT

TCTTCTCTCCCAATTCTCATTTTTTCTTCAGATACATGCAAACATACAGACTACAGACTC

TGCTGCCACCTTGTTTTGTTTCGG

>**Gsoja_MSTRG.26929.1** GeneID=Gsoja_MSTRG.26929;Strand=+;Pos=Chr06:48015542-48016055,

CTGGGACAGCAAAGCGAAAGAAGTCTCCAACACCAACCAAAGCATTCTTGGAGAAAGGGA

TGCGTGTTTTCTCACAAGCAGAGGAATACCTCACAAAGGACAAAAGCAACATCACATTAA

ACCAAACACAAAGGCTGAAGGAGATTGCAGCACCAACATGTCCCAACTCCAATTTAAATA

CAAGGGTCCAACATGTTGCTCCGTGGAAGCACAAAGCTATGGCAGAGGTTAGAATCATGG

GAGAAATCAAACTCTGGGTCTGGAAAAATCGTGTTAGAGGTTTGAGAATTGCGGAACCAA

ATAGAGCAGGTATTAGCCAAATGGCATATTTACGAGCTTCAAGGGAAATTGTTGGGTCTT

GGCCTAAGAGTGTTAATATTTTGTCCATGAAAGTCCATAGAATTGTGATTGGGAAACAAA

TCAAAGAAAGGGAGATTACGGCAGTGTAAGTATATTGTCCAAATTTTTCATATTGCCCTG

CCCCGAAAGCTTGGCCACCTAAAGTTTCCAATCC

>**Gsoja_MSTRG.77247.1** GeneID=Gsoja_MSTRG.77247;Strand=+;Pos=Chr17:40637108-40637356,

GGACTAACCTGATAGGACTGGTTGAAGGCTGTTTAGAAGCACTGTGACAGCTAGCAAAGC

AGAAAGCCTAGTTGTTTCATTGGCAACTGGAACACTGGTGGTAAACAAGTAGGGAAAGTA

ATCCTTTGTTAATAGCACTCCGATCATGACAACAACACCTATGGACACTGATGTGATGGA

AACCACCCACACTGAAAACTTTGCAGCCTTGAAATCACCAGCTCCAAGTTCATTTGATAC

TCTCACACT

>**Gsoja_MSTRG.28719.1** GeneID=Gsoja_MSTRG.28719;Strand=-;Pos=Chr07:9581432-9581504,9581832-9582136,

GGGTGTTACACCTCACTTTTAAAAGAAACTAAATCAAAATTTATCAGATAACTGAATTCC

AGCGGGGATTACTGTTTTTGTAGTGTTATGCTAACGAATAACTATGGTTGTGCTCTAGGT

ACATTGATGTTGATCTCAGTTTCGGATTCATATAACCAACATTATTATTCCAAGATAAGT

CACTAGTTCTACACGTAAAACTAGAATTAAAATTCTTCAACTATCAAGCAGTATTGGCAT

TTTGGTCACACGGAAGGTCCCCAAGTTGGACTCCATCGCCTCCTACTCTTTTTGCTGCCT

TTTTTTACTTCTTTCTCCCAATTGGCGCGTAAGGTGACCAGAAGGAAACACACCACTTGC

ACAGTAAGTGCTATTAGG

>**Gsoja_MSTRG.9428.1** GeneID=Gsoja_MSTRG.9428;Strand=+;Pos=Chr03:464035-464552,

GTTGACAAAATTATGTATCATATTCATTGCCTATGAGTTTAACAGATGAACAGTTCTTAA

AAATAACAAAATGCTTTATAAGCACTCTCAAATTCTGGCCTAGGAAAAAAAGAAATTGTG

AGTAAGAACATAACATACCAGAATACCATTGGCAAAGCGTATGATGACAGTGAATACAAG

AGCATAAGCAGCGAGTTCCTTTGAACCAATGTGACCAATAAATGCTTGGCTTATAACACT

GAGGCCAAAGGTGGTGAACCTAGTAAATATGGCTGGTGCTGCCACTATCCACATCACCTT

GCTCTCTTCCCACACCCTCTTCACCAATGATAAATTCTCTTCTTCTTCTGATATTTTCTC

TCTGCTCAGCAACTTCTGCTTTAGATTCCCCTCCATTTTTCGAGCCTTTTTTTGGGGGCT

TCTTAAGATCAGAATAGAGGAGGGAGTTTGGGGCTCACTGTTTATAAAGAAAAATGGAAT

AAAAGAATGAGTATCTTTTCGCTAATCTTGCATGAACC

>**Gsoja_MSTRG.52475.25** GeneID=Gsoja_MSTRG.52475;Strand=-;Pos=Chr11:45344745-45344832,45345007-45345162,45345337-45345354,45345529-45345617,

GACATAAGGTTGGTTGCCAGGGGGAGGTGTTTTTGGAGTAGTAGTGGGAACATTTGGAGG

ACTATAAATTGGAGAAGGTGTCTTTGGTGGTGTAGGAACATAGGGAGGTTGGTTATGAGG

GGAAGGAGCATTTGGAGGACTATAAGCAGGAGATGGTGTTTTTGGAGGAGTAGGAACATT

TGGTGGAGTGTAAATAGGTGAAGGTGTTTTGGGAGGAGTGGGAACATTAGGAGGATTATA

AGAAGGAGAAGGTGTTTTTGGAGGTGTAGGAACATAGGGTGGATTGTTACTAGGGGAAGG

AGCTTTTGGGGGAGTGTACACAGGAGAGGGTATTTTTGGAGGCGTTGGAAA
