# Supplementary material for: The Identification of MATE Antisense Transcripts in Soybean Using Strand-Specific RNA-Seq Datasets
Source: Genes (Basel). 2022 Jan 26;13(2):228. doi: 10.3390/genes13020228 (PMC8871956; doi:10.3390/genes13020228)
Supplement: Supplementary file 1 [file genes-13-00228-s001.zip › Supplementary Material 2.pdf]

Supplementary Material 2: Sequences, chromosomal position, and strand information of *Glycine max MATE* antisense transcripts predicted from the *Glycine soja MATE* antisense transcripts identified in this study.

>**Gmax_MSTRG.58089.1** GeneID=Gmax_MSTRG.58089;Strand=-;Pos=Chr11:8552160-8552384,

CAGCATACTTGTGAAACCATTGTAGCCCACGCAGCCCCTACAATTCCATAACCTAAATAG

GTGCACAAAAGTACACAACCAGCGACATTTATAACTGTAGCAGCAGCTAAAGCTTTCAAG

GGTCCCAAGGAATCTTTCATACCAAGACTGCAGAATGTGAATACCAAAACAATAAATTGA

TTCAATAAGCCAAGGCTTACCATATTTTTCAAATCATACAAAGCA

>**Gmax_MSTRG.99027.1** GeneID=Gmax_MSTRG.99027;Strand=-;Pos=Chr19:37884598-37884798,

ACTAAATCAATGCATATTCAATACTTTAAGGACTACTCATATAGTTTGATTAGTCTAAGG

ACTACTTAAAAGAGAGTGATACTTTATCTAATGTACAACACTGGGCAAACAAATAATGAG

GATATAAATAGGAATCTGAAACTAATTATCTGATGTAGTTGTTTCATGATCAGATTCCAC

TTTAGACCACTTATTAATACG

>**Gmax_MSTRG.47704.1** GeneID=Gmax_MSTRG.47704;Strand=+;Pos=Chr09:40373194-40373705,

ATAGAATATGAAATAAGAAAATATTCCATTAGTGCATCAAATGACTTATTACAACAAGAT

AAAATAATTGTACTACTCTATTCTATCCGAAACTTAGTTTACAGTCACAAAATTACCCCT

AATATAACCAACCATAGACTCACCAGTGGGGCAATGAGAAAAATTGAAAGGTTTTCAATA

ACTAGATCAAAATCACATTTAGTATCTTCTACACAAGAACATAATCTTTTCCCTCCAAAA

AACATCAAGACGTCCCCTACCAACACATACATAAAAATAAAATAATAAAACCACTTTATT

CACTTCCAATGGCCAACTGCATCATCTATCATCTTTCGCGCATATATAATTGCCCCTAGA

CTTTTCTGCCAAAAGAAGGATACCAAAACATGTATTGTTTTCATCCAAATAGAATTTAAC

TCAGAATGTGCAACTGCAACGATATTGCATCTTCATGAAACTAAGCTCTCAAGTGTTACT

GAATTATAGACTCTATCTGTTGCTTTCTTTGC

>**Gmax_MSTRG.10872.1** GeneID=Gmax_MSTRG.10872;Strand=+;Pos=Chr03:504887-505173,

GCATCTTCCTTAAAAAAAAATATTGGTTGTAGAGTATTGCTTTTTTAAATAACAATGTCC

AATTAAATTCAAGGATCCGATCCTATCCAACATAATTTGCGTCCATACAAATAAACAATA

TTTGCCTGTATAACACTAGCAACAGAAAATATATTGCCATAAATAAATCCGTGGCTAGAT

TCATTAATTTTGAATTTAAAAAACTAAATCAAAGAAGGAACATGAAACTAATCATCTGAT

GTAATTGTTTCATGATCAGATTCCACCTTGGACCACTTATTAATACG

>**Gmax_MSTRG.6395.1** GeneID=Gmax_MSTRG.6395;Strand=+;Pos=Chr02:7919393-7920474,

CTCCACCCTAGACGTTTACTTCCAAAGCTTATTTGGTTGTAAGTTGTAACTCAAGACTAT

AAAACATGCATCTTCTTCCACCTCGATACTATTAAATTGCATTGAATCTGCAAAACCATA

GAGGCTCCCACCACTTTGCATTCCACAACATTATACATAGACATAATAACTTTTATCTGT

TTACTTCACCAAAATAGAAATAATAGCAAAACCAGAATCTGACAGAAAATTAGTCATTAC

TTCCTTTTTCTTCCACTCTTTTATTCTTCTACTATAATTGCAAAATTTAAAATTTCAGAC

TGAACCATTATGAGCTTTAATTGACTTCTCAACTATTCTCACCCTTGCTTTTGTTGCCTG

CAGTAACCACAAAATACAATCATTTAGAGCCACTTTAATTAACAGTCAATTGTTATCCAT

TGGATTCATCAAGCAAAGGCAACAAAAAGGGCTCAAAACTAGAAGGAAAGCAAAAAAACA

AATGACACGTGCAATTATTAACAATGGAGAAGTTAAAACTGTTACACTGTTAAAAAAGTA

AAAACCAACCTCTTTCTGCCAATCTGTTAACACTGTTACAACAGTCAGAATAATCACTTG

TAGAACAGATCCTGTAAGACTTCCCATCCATAGTCCCTTGGCATTGAAATGTAGGACAAA

ACCCAATAAGAATGCTAAAGGAACTCCCACAAGATAATAGGCTCCAAGGTTCACATAAGC

TCCTATTTGCTGAAATCCTCCACCTCTTGCAATCCCTGCACATATGAATATCCATTTTAT

TGCTAATATTTGTGCTCAATTAATGATGCTAGAATAACATAGACTATTTCAGCAAATAAA

AACTCACCAGAAAGAGCTCCTATTAGACTATCTGCAGTAAAAGACCCACAAAGAATGGGA

ACAATATCTGAAACATAATCTACAACTTCCTTGTCGTTGCTATAAGCATATCCTAATATA

TGCCTACAACAAACGAAGAAAATGCTGACAATAACCCCATCAACAATTCCAATAATCACA

ATAACACGAACAATACCTTGAGCTGCCTTTGGATTCCCTGCTCCTAATTCATTCGAAATG

CG

>**Gmax_MSTRG.54537.1** GeneID=Gmax_MSTRG.54537;Strand=-;Pos=Chr10:46174491-46174911,

TACCTGAATATGTCACTATTTTATGCTATAAATAATTTTTATTACCTAATCTAGTCCTGC

ATAGAGATATATTAGCTCATCTCTATGTCAGAGTAAAAGAAAATATCGATGTTTTTCATT

TTTCACTGAGATGCTCAATAACTAAAAAATGAAAATTAAATTGGAAAAATAACCAAGTTT

TTTTATCTTAATCAGACACCACGGCTAAACTATGTTCTGTACCTAATTGGAATAGAGCAA

CATTACGTGTAAAATGGACAGAGAAGGAACACATAAAAAAAAAAACAAAAAACAAAAGAC

TTTGAGAATTTGACTCGAGTGGAGGTGGAAGATATACAGGATTATGTAGAAGCCACTATC

TTATCAACTCCAATGTCTTGTCCTCCCCATATCCGCATGCGTTCAGTTGTTTGTTCCACC

T

>Gmax_MSTRG.91144.1 GeneID=Gmax_MSTRG.91144;Strand=+;Pos=Chr17:40285361-40285584,

CTGGTTGAAGGCTGTTTAGAAGCACTGTGACAGCTAGCAAAGCAGAAAGCCTAGTTGTTT

CATTGGCAACTGGAACACTGGTGGTAAACAAGTAGGGAAAGTAATCCTTTGTTAATAGCA

CTCCGATCATGACAACAACACCTATGGACACTGATGTGATGGAAACCACCCACACTGAAA

ACTTTGCAGCCTTGAAATCACCAGCTCCAAGTTCATTTGATACT

>**Gmax_MSTRG.54675.1** GeneID=Gmax_MSTRG.54675;Strand=+;Pos=Chr10:46755636-46757588,

GTGCAAGTTAATTAACCGATGGCAAATAAAAGAGAAACAACAACAAGACGATAATTACGT

GAAAAATTTTAACATTATTATTGTAGTTAAATTCCTTGATCCACCCATGTGATCTAAAGT

TATATTAAAAAAAAAAGTCATCCTCTGTCACTACCCTCTCCTTCCCTATACAGCTAAAGA

CAAATTCCTGATAGATTTCTCTCTCTCCCTTTGTCTTCTTTGATTTTTCCCCTTTTGGTT

TTTGTTCCTGTCCAAAAAAACCGAGATTACTAAAAGTTAAACCCACGATTGTTTATCTTC

TTCTGAATCAGCTAATGATAATAAAGAATCTTCCTTGATTTCAGCTTTGGGTGGCTTCTC

TGCACCAACCTCTTTGCTGTCATCAACAGCAACAACAACACTGGTTAGTTTCTTTGCCCT

TAGAGCCTCAACATCCCAATCCGTTTGACTCATAACCACCAACATGGTCACTGCACAAGA

CCCCTGAGCCGCAAGTAACCCAAGCCACAACCCTTGAAAATCAAGCCCTGCGAAGAAACC

AAGCCAAACCGCCACAGGCATTCCAACAAGGTAGAAACAACCTAAGTTAATGTTAGCACC

AACTTTAGGCCTTGCTGTGCCTCTTAGCACTCCGCACCCCGTTGTTTGAGGACAGTTTCC

GAGTTCGCAGAGTCCTATAATCGGTAACACCAATGAGGTCAAGGTTATTATTTCCTTGTC

TTGTGTGAACATGCTGGCCCAAATGTTCCTAACCAGAATGGTGAAAACAAAAGCTAAAAA

ACCTAACATGAAGCTGCAAGAGAGGCCAACTATGGCAGAAAGTTTAGCCTTTGAGGGCTT

TTGAGCACCTAATTTGTTACCAACTCTTGTGGACACGCTGAAGCTTATTGACGATGGGAG

AATGTAGAGCAGAGAAGTGGTTTGGATCAAAATCCCCATGGAAGCCACGGTTGCTCTAGG

ATTCACCAACAACCCGCATAACAAAATCATGATCTCATACCACCACCATTCTAGGCACAC

GGAAATGCAGCTTGGGATAGCCAAGTCGAGGAGCGATTTCCATTGCGTGAAACACTCAAA

AGAAAAACCTCCCCATGTTTTCTTATGCGTACCAGAGAAGACTATGTAAAGAATCAAAGA

AGCTATGAGGTTGAAGTTAGTCCAAACCCCGCTGAGAGCCACGCCTTTGATTCCCCAATT

GAGGTGGGAAACAAGAAGGTAGTTAATGGGGATGTGAAGGAGAATTGAGAAAGTGGCGCA

GAGAGTGAGAGGCAGAGTAATGGATTGGCTTCGAAGGTAAATTCTTAAAGGGTGTAAAAA

AGATTGTGCTAAGAGGTCAGGGATGGAATAAAGAAGATATGATTGGGCTTGTGTGGCTAT

GGCCTCGTCCTGGCCACATAAAAGAAGGATATGCTTCATGTAGAGCCAAAGGAGAGAGAT
TGGAATGGAAGTGAAGAGGAGCAAAAGAATGGTTCTTTGTAAGCAGAGGCCGAGGAGGGT
GAACTTTTTGGCACCGTAGGCTTGTCCGCAAAAGGGTTCCATTCCGACAGCGAGGCCGGA
GAGGATCGAGTAACCGGTGATGTTGGCGAAGCCGACGGCGAGTGAGCCGCCGGCTAAGGC
CAGCTCGCCGAGGCGGCCGAGGAAGAGCATGGAGATCATGGAACGGCAGTAGAGCAAGAG
ACCGGTGAGGATCATGGGGAGGGCTATCTTGGATATGGAAATGAGTTCTTTCAAGACATG
ATGATGGGGTGGTGTTACTGTTATGTCCTTTTGGATCAAGGGGTTAGTGATCTTCATGTC
GGATTCCTCTGCCTCTTGGGATTTCATTGAGTTGCATTTGCAGGGGAGCTGGGAACTTAT
ATGACACATAGTTTCGCGAAAAGAGTGGTTTAGAGAACACGTGAAAGTTGAACTGAGAAA
GAAAGAGAGAGAGAGAGGATTCTAGGGGAAAGG

>**Gmax_MSTRG.85655.1** GeneID=Gmax_MSTRG.85655;Strand=-;Pos=Chr16:34064665-34065179,
TGGCACCGATGACACCTTGGTAAACACCGATAGAAGAGAGTTCAATGTCTCCAATATGAC
CAGCATAGATGGAAGTTGAGGAGTCCATGAGAAGCTGAAACAGTGCAAGCAGAGCCATTG
GGAGTGCTACTCTCCATATCTTCACTGTCTCCGTTCGCAAAACAAACATAACATCCTTCA
AGCTTTCCACCGGCAAATAGTCGGGTTCTGAAGTATGCTTTTGGATCACTAATGGGGTTT
CCATTTGAGGAGTTCTTTTTCTTCCCTCAACACTCAGTTTCTTTCTAACTGAAGTTTGTT
ATAAACTTCCAATCATACAACAAGGAGTCATAAGGATGGCTTGGTTGTTGAATGCAGAAG
GGTGAGAGAGATTGCTAACAAAAACTAACAAATTAACAATTAACTTTTGTCAATCAAATA
AAAATAATAACTTTCAAATCATAAAAGCCAAAATCTTAAACAGATCATGTAAAGCTTTGA
CGCAAATGAGTAAGCACGAAGGCTTTGCCTTTGAT

>**Gmax_MSTRG.10867.1** GeneID=Gmax_MSTRG.10867;Strand=+;Pos=Chr03:490615-490977,
CAGAATACCATTGGCGAAACGTATGATGACAGTGAATACAAGAGCATAGGCAGCGAGTTC
TCTTGAACCAATATGACCAATAAAAGCTTGGCTTATAACATTGATGCCAAAGGTCGTAAA
CCTTGTGAATATGGCTGGTGCTGCCACTATCCACATCTCTTTGCTCTCTTCCCACACCCT
CTTCACCAATGATAAATTATCTTCTTCTGACACTTCCTCTTTGCTCAGCAACTTCTTCTC
TAGATTCCCCTCCATTTTTTGAGTCTTTCTTCTTGAGATCGATCACTGTACACAAGGGAG
TTTGGGGGCTGATATTATAAAAAGAGAAAAAGGAAGAAAAAAATAAACACAAGTGAATCT
AGC

>**Gmax_MSTRG.10876.1** GeneID=Gmax_MSTRG.10876;Strand=+;Pos=Chr03:545409-545697,

TACGAGCAAAAGTAATGTCTCTATTGAAAAATTCAAAAGTAAGAAAGAAGTCCACTACTT

TTGATTGAAGACTTTATGATCAATAAATAACCATTTCTTCACCAAAAAAATGACGTACAA

TTGCTATCTACTTTTTTGCTGTATCACAACATAGTACATAGATGATGACACTGAAACTAA

TTAGCTAACGTTTTTTCTTTCTACTTCATTTTCATGATCAGGACTGTCCACCTTCGACCA

CCTGCTAATACGCTTCTGAGCAATGGTAACCTGCACAGCATGATATGAT

>**Gmax_MSTRG.58093.1** GeneID=Gmax_MSTRG.58093;Strand=-;Pos=Chr11:8558800-8559215,

GTCCACAAATCCAAAGACCCGTTGCTGGTCCTGTAAACATCACAATTTCCTTTATCTGAC

TCCATATGCTTTGCTTCGCGAGTTCCTTTTTCTCACCCTGCCTCGATATTTCTTCGTCTT

TTTCTTCTTCTTCAAAAACATCACTGCTTTTGCTTTCGTCTTGGCTTTGAAATGCGCGAA

CGGTTACGGCGAAGAGAGTGCGATGAAATGTGGAGACTGAGGCTGCGCCGGAGAGACACG

AAGTGGGAGGAAGGGAAGGTGCAAAGAAACGAAGGGGAATGCGATGGTTTGAATGCGACG

AATTAGGGTGCTGGTGGCGGAGAGAGGAGTGAAGAGATAGTGACGAGAGTTTCAAAGCCA

TTTTTCGCTCTCTGCTTTCGTTTGGTAGAGGGAACGAAAAAGAACCCGAATATTTC

>**Gmax_MSTRG.62822.1** GeneID=Gmax_MSTRG.62822;Strand=-;Pos=Chr12:8614915-8615378,

CTGTTAATAGGATATAGTGTAGCTAGACCCTAGATATAGAATGGTTTCGTGTAAGTAGGG

ATGATTCATGAAAATGAAATTGTCTATATATAGTTTTTATATCAATGGTTTAATTCTTAT

TGGATAAAAAAACACGCGGAAGAGAGGCAAATCCAACCATGCATGGATGTGCATCACATT

TTTATTTGTAGTGATTGGAATGAATATGAAAGTTAAATGAAGATATTACCAGATAAGACA

GGTTGAATGCCGTTGAGAATAATAGAAAGAGCAAGTAGAGGACAAAGATCTGAGACAGCA

GCAGCCACCTCTTCACCGTCTGTGAAGCATAGCTAATGACATCTCTTATTGCCAGCACCA

CAAGTGCTACAATAACTGATATTATGAAAGAAATCAATGTCACCACCACAACCGAAAATG

ACGCTGATTTTGGATTTCTTGCCCCTAGTTCATTGCTCACTCTC

>**Gmax_MSTRG.10865.1** GeneID=Gmax_MSTRG.10865;Strand=+;Pos=Chr03:484058-484336,

TGCTAGTAAAATATATAAATCCAAGGTCTAAATACAGGAACATTTAATACTTTAGCAACT

ACTTATATAATTTTATTAATTTAAGAAATATTTGAGAGAGAATATACTGATAGGATGGTA

CTATAGTATAATAACTATCAAGCAATCCCCTGAATGTATAACGTACAATAGCCAAACAAA

CAATGATGATATTGATAAATAGGAGTCTGAAACTAATTATCTGATGTACTTGTTTCATGA

TCAAGTTCCACCTTATACCACTTACTAATACGATTACGG

>**Gmax_MSTRG.5537.4** GeneID=Gmax_MSTRG.5537;Strand=+;Pos=Chr02:3724328-3724482,3724528-3724695,

GGGTCCGGCTGTGCCTGGTGCTGGTGGAGAACTAGCGGAAGGGACTGGTGAGGTTGCAGG

AGAAGAAGCAGGTGTAGGTGCAGCTGTTGGTGTTGGCGTTGGTGTTGTTGCTGGTGGTGA

TGTTGCTGGTGGAGGAGTTGCTGCTGCTGGTGGTGGTGCTGATCTTGGTGGTGGTGGTGA

TGGGGTTGTAGTGGGTGGTTGTGTGGGTGCAGCACCTGGGGCTTGTGCTAAGCATGAGCT

AGCCAGAAGACCCAAGATCAGAAGAAGTTGTAAAGCACCAGAACCCATCTAGCTATAGAG

AAAATGAGAGACCAAAGAAAATG

>**Gmax_MSTRG.65373.1** GeneID=Gmax_MSTRG.65373;Strand=+;Pos=Chr12:39628871-39629081,

CCCATGTTCCCTGTAGCTAATTTTTCCACATCCCCAGATGTAAAAGTACTCTTCGCTACA

ACATTGTTTGATTCGTCGGTTTCCCCCTTGAATTTTTTTGGAGCCTCAACATTTTCTTTG

GTTATTGTTTCATTTTCTATGTTATCAGTTTCAGTTTCTTTGTTAATCAACCTTTGGATA

GTATCTTCCTCAGCCACAAAGGAAGTTGTAA

>**Gmax_MSTRG.5537.1** GeneID=Gmax_MSTRG.5537;Strand=+;Pos=Chr02:3702043-3702058,3723845-3725003,

TTTTTTTTTTTTTTTTTGTAAGTAAAAAGAATGACAAACTGTTTTATTCTTTCAATTATT

AGATGTACAATGAAATGATCAAGGTAATCTACATACACGTGTGAACACCAAAACAAATGA

ATCACCAAATAATCATCATCAACGGCCACCACCCCTCCCAAATCTCGAATGGACAAACAC

ATCAACAAAGTACAAAACTAGAACAAAATAAGTAAACACAAAACCCGAAGAAAATAAAAC

CGCTAACTCAACATATGACCAGAGGATGAAACAAATCAAATGGATCTCGTCCGTCCGTGT

GCGGGAAAGAAGGATCAAGGGCGGAAAAGCCTCCACGTGGACGAATCATCAAAGACAATG

CTTCTCTCGAAAGTTTTTAAGCCAAGACCATGGCTACGAAGATTCCAGAGAGTGCGGAGC

CAGCAATGAAGGCCTTGCTGGCGGAGAATGCGGCGGAGGGTGGCGGTGGTGGCTCGGCGC

CACCTGCGGGTCCGGGAGCGGGTCCGGCTGTGCCTGGTGCTGGTGGAGAACTAGCGGAAG

GGACTGGTGAGGTTGCAGGAGAAGAAGCAGGTGTAGGTGCAGCTGTTGGTGTTGGCGTTG

GTGTTGTTGCTGGTGGTGATGTTGCTGGTGGAGGAGTTGCTGCTGCTGGTGGTGGTGTTG

CTGGTGGTGGTGTTGCTGGCGGGGTGGGTGCTGGTGCAGGTGCTGATCTTGGTGGTGGTG

GTGATGGGGTTGTAGTGGGTGGTTGTGTGGGTGCAGCACCTGGGGCTTGTGCTAAGCATG

AGCTAGCCAGAAGACCCAAGATCAGAAGAAGTTGTAAAGCACCAGAACCCATCTAGCTAT
AGAGAAAATGAGAGACCAAAGAAAATGTTGTAGTGCTTGTGTGTGTAAGAGAAGAGAGAG
AGAGAGAGTTTGAGCAGTGGAAAGATGATAAGGGAACAGAATATATAAAAAGAGAGGTAG
AGTATGTAAGTGGGGGGCTTCAAAGGCGTTAAAGGACAGCGCTGATTAATGCATCAATAA
TGCTACAAGATTCCTCCAACTCCAATGTCTTAATTTAATTTGCTCCAATATTTTTTTTCA
GAGAGATTACTACTGTACTCCAATCAACTAGATCAAATATTAATTTCTTGTATAATTAAT
GATTATCATTGATCCAATAAAATAAAATTATGTAG

>**Gmax_MSTRG.47706.1** GeneID=Gmax_MSTRG.47706;Strand=+;Pos=Chr09:40375297-40375880,
CAAACCATAACAGCTGAAGGAATGGCAAGCCTCACAAATGAGGGAATGTTATGCAGTGCC
TCTTTGGAAAATCCTGTCCAAGATTTTGCACATGAAGGAGAAAACATGACATAGAGTGAG
AGTATAGTAACATTCAACCAATAAGATATAGAATTTGCTACAGCAGCTCCTCTGTTCCCA
AGTCCAGACTTGAAAACTAGAATCCAACATATAAGTACATGTAGTAATGTGGTAATTGCA
GAGCTGCACATCATTGGGAATACAATATTTTGGGTTTGTAGAAATCTGTTGAGGCACTGT
AGAAGGCCATAAGCAAAAAGGCTTGGAAGCATAAACCTAGCATAACTCCCGGCTTCTGCA
GCTATTTCAGGATCCTGGCCAAGGAAAGTTAGAATGGACCTTGTGTTTGCCCAAATAATT
GCAAGCGGAATGCTGACGATCATGAGAGTGAACATGGCTCTCTGCAAGTGTATGCCTAAC
ATGTGATACTGCTTTGCTCCATATGACTGGCCACAAAACGTGTCCAAGGAACTTGCCATT
CCTACCTATTTCAACCAAAAACCAAACAAAGAAAAGAAAAAAAA

>**Gmax_MSTRG.46374.1** GeneID=Gmax_MSTRG.46374;Strand=-;Pos=Chr09:18853354-18853738,
ATGCACTCAGAGTTTACATACTACTAACAGTACGAAGAATAATCTTCTAAATACGACAAA
ATATTACAATAAGCAGAGGATACATCATTAAACAGAGACCATACAGTAAACATAGGCCAA
AAAAAAAGAAAGATACTTTGAATATAACACTTGCAAGTGTAAAATGAACCCGTCCAATAT
AATATATAATAAGACTATCACCATAGTTGTATAGTGCATAAATCATGTTTTGAATAAAGA
CAACTGGTCCTAAATGGTGAAAAGTGATACAAAACATTTTCTGCTCAACAATGAAATTTC
TTTTGTTAGATGTCATCTCATCATCAAGACATTGAGCGGCCTCTGAGAAAACGCCATGGT
CCCGTACCTGTTCCCATCCTGCATT

>**Gmax_MSTRG.46907.1** GeneID=Gmax_MSTRG.46907;Strand=-;Pos=Chr09:33360850-33361136,
TGAACTAGAAACATTTTATTTTAATTAACAATAAAGATAGTGTCAAAAAATATATTAAAA

AGTGTATTGTTATTTTTTGAAAAGAAAAAACAAATTTTACTACCACAAAACTTGTCAGAC

CACTAGATGTGTGGCCAAACAAACCAAATTACCAAAAGAGATGCTGATTAAAAAGTGCAT

TGTTAACATTGTTTTTTTTTCCTTCTCATTCATGTAACATTTTCTGCCAAGTCTGAGCGG

AAATTGTTAACGTTCCATATTCGCATGCGATGAGCAGTTTGCTCCAC

>**Gmax_MSTRG.536.1** GeneID=Gmax_MSTRG.536;Strand=-;Pos=Chr01:2550530-2550539,2601674-2602317,2684302-2684316,

AGCAGAAGAAGAAGAAAAAAAGAAACCGCTACTCAACATGACCAGAGGATGAAATAAATC

AAATAGATCCCGTCCGTCCGTGTGCGGAAAAGAAAGATCAAGGGCGGAAATGCCACCACG

TGGGACGAATCAAAGACAATTGTTCTCATGAAAGATCTTAAGCCACCAAGGCCATGGCTA

CGAAGGTTCCAGCGAGTGCAGAGGTAGCAATGAAGGCCTTGCTGGCGGAGAATGCAGCGG

AGGGCGGTGGAGGCTCGGCGCTACCGGAGGGTCCGGGTGAGGGTCCTGGTGGAGTGTTAG

GAGAGGTGGGGCTTGGTGTCACTGTAGGAGAAGGTGAAGGAGGAGAGGCTGCAGGAGAGG

AAGCAGGTGTAGGTGCAGCCGTTGGTGGTGCTGGTGCTGGTGTTGGTGTTGCTGCTGGTG

GTGGAGTTGCTGCTGGTGGTGGAGTTGCTGCTGGTGGTGGGGTTGCTGGTGGTGGGGTTG

CTGGTGTGGTGGGTGCTGGTGCAGGTGCTGATCTGGGTGGTGGTGGTGATGGGGTTGTAG

TGGGTGGTTGTGACGGTGCAGCACCTGGTGCTTGGGCTAAGCATGAGCTAGCCAAGAGCC

CCAAGATCAGAAACAGTTGCACAGCACCAGAACCCATATCTAGAGAGAAAGTAGTAGTAT

TGGTGCTTG

>**Gmax_MSTRG.65157.5** GeneID=Gmax_MSTRG.65157;Strand=+;Pos=Chr12:38511645-38511775,38511902-38511961,38512040-38512167,38524216-38524226,

GGTTATTGGAACAAAAGGTGGTTGATGGGTAGGGGAAGGGGTTTTTGGAGGGTTTGGAGT

GTAGGGAGGTTGAGAAATAGGAGAGGGAGTGTTTGGAGGGGTTGGGACATAAGGTTGGTT

GCCAGGGGAAGGTGTTTTTGGAGGAGTGGGAACATTTGGAGGACTATAAGAAGGAGAAGG

TGTTTTTGGAGGTGTTGGAACATTAGGAGGAGTATAAACAGGGGAAGGAGTTTGTGGAGG

AGTTGGAATAAATGGAGGATGATTAGTAGGTGAAGGTGCTTTAGGAGGAACATAGGGAGG

ACTGTGAATAGGAGAAGGTGGAGGAAGGAG

>**Gmax_MSTRG.54535.1** GeneID=Gmax_MSTRG.54535;Strand=-;Pos=Chr10:46171777-46172454,

TGGGACTACATGGCTTTTTTTGCCTGTGTGGGGATGAAAGTATGATCTTACCATATAGAG

AGGGAATCAACAGCAACCAATGCATTATCAAGGTTGCCAGCAAGAACTATAACGCTCATC

ATATACCAAACTTCAAGGCAAAGCATTACAGCTGATGCAAGAGAGAGCCTAACAAAGGCC

CAAATATCCTTAAAAGCCAGCCATGACAATCCAGTCCATCCATCCTTACACCAGATCACA

ACATAAACAAGTTGAGCCACTGTGATCCCCCAGCTTGTGATATCAAACGCCAAAGCTGCA

CCAGCTAAGCCAAAATCTAGCACATAAATTAAGAGCCAGAGCATCCCAATGTGCAGAATT

AAAGCCACCAACCCAATCCATGCAATAATGTTAACCTTGCTCTGAGCTTGAAGGAACTTT

TGCGTTGGAAAATTGAAGGGAAGTGAAAGAAATTGTGGAATTACTAAAATAGAGAAACTC

CCTGCAAGATCAGCTATATCTTCCTGTTGACCAAGAAACTTCAAAATTGGACCAGCAAAA

ATGTATATTGGCAAGAGCAAAATACTGGTCACGGATAATATCACCCATGAGCGTTGCATA

TAAACACCAAGCATATTAACCTGCCCAGCTCCGAAAGCTTGTCCACAAAGCGTCTCAGTT

GCACTTCCCATCCCAAGC

>**Gmax_MSTRG.46906.1** GeneID=Gmax_MSTRG.46906;Strand=-;Pos=Chr09:33358686-33358945,

CTGACATGACCTGTGAAGCACTATTGAGAACCATGGTTACACCAAGAAGGTATGCTAAAT

CAGCAACAGCTTGTATCATATCCTCACTATTGGTAAAGATAATGGCGAAGTCTTCTTTAG

TCAAGAAAATAACATTCATGAAAAGAACACCAAGGAGGAGAGACTGGAACATGGTCACAC

AGAAAGTGTATTTGGCTGCTCTTGGCTGCGACATGCCAAGCGCATTGGAGATGCGAACAC

TTGGAGGGCATAGAACAAAT

>**Gmax_MSTRG.104828.1** GeneID=Gmax_MSTRG.104828;Strand=-;Pos=Chr20:39433098-39435386,

ATTTACGTGAAAAATTTGTAACATTATTATTGTAGTTAAATTCCTCGATCCACCCATGTG

ATCTAAAATTATATAAAAAAGAAAAAGTCATCCTCTGTCACTACCCTCTCCTTCCCTATA

CAGCTAAAGACAAAATTCCTGATAGATTTCTCTCTCTCCCTTTGTCTTGTTTGATTTTTC

CCTTTTGGTTTTTGTTCCTGACCAACAAAAACCGAGATTACCAAAAAGTTGAAACCCACG

ATTGTTTATCTTCATCTGAATCAGCTAATGATAATAAAGAATCTTCCTTGATTTCAGCTT

TCGGTGGCTTCTCTGCACCAACCTCCTTGCTGTCATCAACAACAACACTGGTTAGTTTCT

TTGCCCTTAGAGCCTCAGCATCCCAATCCGTTCGACTCAGAACCACCAACATGGTCACTG

CACAAGACCCCTGAGCCGCAAGTAACCCAAGCCACAACCCTTGAAAATCAAACCCTGCGA

AAAAACCAAGCCAAACCGCCACAGGCATTCCCACAAGATAGAAACAACCTAAGTTAATGT

TGGCACCAACTTTAGGCCTTGCTGTGCCTCTCAGCACTCCACACCCCGTTGTTTGAGGAC

AGTTTCCGAGTTCGCAGAGTCCTATAACCGGTAGCACAAATGAGGTCAAAGTTATTATCT

CCTTGTCTTGTGTGAACATGTTGGCCCAAATGTTCCTAACCAGAATGGTGAAAACCAAAG

CGAAAACACCTAACATGAAGCTGCAAGAGAGGCCAACTATGGAAGAAAATTTTGCCTTCG

AGGGCTTTTGCGCGCCTAATTTGTTGCCAACTCTTGTGGACACGCTGAAGCTTATTGACG

ATGGGAAAATGTAGAGCAAGGAAGTTGTTTGAATCAAAATCCCCATGGAGGCCACGGTTG

CTCTAGGATTCACCAACAACCCGCATAACAAAATCATGATCTCATACCACCACCATTCTA

GGCACACGGAAATGCAGCTTGGGATAGCCAAATTGAGGAGCGATTTCCATTGTGTAAAAC

ACTCGAAAGAAAAGCCTCCCCATGTTTTCTTATGGGTGCCAGAGAAGACTATGTAGAGAA

TCAAAGAAGCTACGAGGTTGAGGTTAGTCCAAACCCCGCTGAGAGCCACGCCTTTGATTC

CCCAATTGAGGTGGGAAACGAGAAGGTAGTTGATAGGAATATGGAGGAGAATTGAGAAAG

TGGCACAAAGAGTGAGAGGCAGAGTAATGGATTGGCTTCGAAGGTAAATTCTTAAAGGGT

GTAAAAAAGATTGTGCTAAGAGGTCAGGGATGGAATAAAGAAGATATGATTGGGCTTGTG

TGGCGATAGCCTCGTCTTGGCCACATAATAGAAGGATATGCTTCATGTAGAGCCAAAGGA

GAGAGATTGGGATACAAGTGAAGAGGAGCAAGAGAATGGTTCTTTGTAAGCAGAGGCCGA

GGAGGGAGAACTTTTTGGCACCGTAGGCTTGTCCGCAAATGGATTCCATTCCGACGGCGA

GGCCGGAGAGGATAGAGTAGCCGCTGATGTTGGCGAAGCCCACGGCGAGTGAGCCGCCGG

CTAAGGCCAGCTCGCCGAGGCGGCCCAGGAAGAGCATGGAGATCATGGAACGGCAGTAGA

GCAAGAGACCGGTGAGGATCATGGGGAAGGCTATCTTGCATATTGAAATGAGTTCTTTCA

AGACATGATGATGGGTTTGCATTGTTCTTGGGTCGTTTTTTTCTTCATGGAGTGTTGTTA

GTGTTACTGTTATGTCCTTTTGGATCAAGGGGTTGGTGATCATCATGTCTGCCTCTGGGG

GTTTCATTGAAACTACATACTCCGGGTTTGAGTTGCATTTGCAGGGGAGGTGGGAAGTTA

TATGACACATATTTTCTGCTTTTTGTGTGTGTGTGTGTCAAGAAAAGAGTGGTTTAGAGA

ACACGTGAAAGTTGAACCGAGAAAGAAAGAGAGAGATAGAGAGGATTATAGGGTGAACGG

GAGGGGGTTGAGCACAGGCTTGTCTTGGGGCTTTTATAGGAGATTTTGGGTGGGAATATG

GGCGTCAAATCTGTCCGAGAACATGTTTCGCTATGATGTATGATTTGTAGTTAATTATAT

TTGTTAAGTATATAGTCAGGTATTGAATTTTTATATCAGTTATTTGTTGAGAAAAGATGA

ATTTTTTTAAGAAGAGTCTATGTTTACTTAGGAATTAGTCTTCGACTTTTCACTAGAAGG

ACCAAAAGATAAGTGAACACCCAACAAAATTCAAAAACAAAAAAATTAGCACTTGACGCA

CTTTTGGAG

>**Gmax_MSTRG.10878.1** GeneID=Gmax_MSTRG.10878;Strand=+;Pos=Chr03:548081-548619,

CATAACATGGCACCAGCTGAAAGGGACATCTTGACAACAGGCCAGAGGTCTTTGAATGCC

AAAAATGTGAAACCTTTCCATGTATCAGAGCACCAACCACATGTGACAAATATGAGTTGA

CCAATATTAGGAATCCAGTATGCCAAGCCTGCTGAAATCATAGCACCAGGAATCCCAAAC
TTGAACTTCATTGTCAAAAGCCAGGAGAGAAACACGTGAATGACTATCGAAAAAGCCGCC
AAGAACGCAATGATGATGTTCTTGCTTTGAGATTGCAGGAACGTCTGGCAAGTGAACGAG
ACAATGAAAGCAAACATGACAGGGATTGACCAAAGAGCAATGTTTCCTGCCACTTGTGCT
ATGCTCTCATCTTGGCCCAAGAGCAACAAAATGGGGCTTGTGAAGATGAACACAGGAAGA
AGACAGACTGCAGTTAAGAACAAAACTATCCATGATCTTTGAAGATACACTCCCATCATG
CCATATTCTTTTGCCCCATATGCTTGTCCACAAAGTGTTGACAACGCACTCGCCATTCC

>**Gmax_MSTRG.95016.1** GeneID=Gmax_MSTRG.95016;Strand=-;Pos=Chr18:49026223-49030293,
CCACACAGTACACACCTAACTTGAATAGCAACAATGGACAAATCAGAGTACCTACTCTTA
CTTGCATTGTAGCTACCTTTAGCAATGAAAAAATACAAAATTGTGCAATTTCCTAATAAA
TACAAATTTCCAACAAAAAACCAAAGGGCCAAAAAGACTAGCCTCTAGGCTGTAGGCTGG
TCATTCAATAATTTTCTCCCTCTCTGTTCTATTGGTCATTCAATCTCTACTTGAATCAGC
AAGATTTTGACAATCGGTTAGCAGTAATTGGCTGCGGCATTTCAATAATTTACTGTAGTC
CTGCAGTGATACTGGATTCTCTTTGCATGGATTTAGTGCACTCATATTAGAGATCTTGAG
AATCATGTTCTGATCTAATCATTATGAAAATGGCTTTTTAACCTGAGGAGTGAATGTTGG
AACTTCTGAGTACTACTGAACACATGACCTTGAATGCATGTTGGTCAATCTTCTGCTCCA
TCTTTATACCAGAATGTATCCCAAGGCCCACTTTTGCTGCTCAACCTTAAGAAAACAAAA
ATATTAACTCAATAAAAATCTTATGAAGAAAATTTTGATCATGTTATTCATAGAGTATCC
TACCCCTCCCCAATAAAATACCAAGATAAAAGAGAACAAATAATATGTGACTTGATGTCA
AGTCACATTCATCCAACATGTCCTGTGTCATTTTGCCATTGTTCCGAGGTAGGATTTGAA
TGAGTAGTGCAATGGGTAATAACACACCAACATGAAGTTTCCCAACTATCACCTTCCAAA
TGAATTAAATACTTCACATCCAAAGTCCAGCTAGAACACGCAAAGCCATGAAGATAAACA
ATCCAGTCCAGACTCCAGGAAGCCCTACTGGAGCCACCACCAGAAGGAAAGTTGAAGAAA
CTAGTCCAACTAACACCACCATAACAAAAACCAAAAGAAAAAAAGGGCACGAGGAAATGA
GTTAAGAGGAAAGAATCATATGGCATGTTTAACTATCTTTCAACCATGGTAGGAAATTAC
CATCGAGTAAGCAGCATAACCAAAGTCTGATACCCCGTAATAAATCCCATCAATAACAAA
TGCCAAAGCATTCACTGGTTGAGATCCAGCAACAAACTGCACATGTAAATAAATTTCAGA
CATTCCAATTGACGTTACTGTGTATACATGCTTCCACAGAAAGAATGGAGAATTGCACTG
CATATAATGAACCCTTATCAGCCCTACTAAAAGGTTACCATGGCTTAAACACCCATCAAG
AACAAAAAATTCTGATACTGTCAAAAAATATTAGCTTTCAATGTATTAATCAACATTAGAG

GAAGAACAGACAGACAAAAAAAAAATATAAACAGACATCCAAATGAAATGCTTTCTTTTA

CTTTCTTGAGCGAACTCCTTTCCTGGTAAATGCACTATAATAGTTTCAAACAAAAGGATT

TCAATTTGGAGTTGGTACTCATGACTTGGCCACTCAGACCCAATTCTGCACAGGCTAATA

CTTTCAAACAAAAGGATTTCAGTTTGGAGTTGGTACTCATGACTTGGCCACTCAGACCCA

CTTCTGCACAGGCTAATACTTTCAAACAAAAGGATTTCAGTTTGGAGTTGGTACTCATGA

CTTGGCCACTCAGACCCACTTCTGCACAGGCTAATACTTTCAAACAAAAGGATTTCAGTT

TGGAGTTGGTACTCATAACTTGGCCACTCAGACCCACTTCTGCACAGGCTGAGTGTCTGA

CTCTTAAAGTCAATCCAAGTCAACTAGGGAGTGGCATATAAAGCCTACAAAATGATAATA

AAGGTAATTTTCTGCAAACAACTGTGATTTCTTACCCATATACCTGACCGAGCAACATCC

AGAACTTCAGAATCTGTGCTAAATAAACTAGAAAATGCTCCAAACCCAAAGAATAAGATC

ATTGACAAAGTGATTCCTGCTCCTAAACCAATCTGTTTAACATGTAAAAGAGAGTACCAG

AAAAGAATTCGGGGTCAGTATAAGTTCCAATCTCACTAACAGAAGGTATGAAGGAAGAAG

AAAGCAATTTATGGTGACAACCTGCATCACTCTAAATATGACAAGGCTTGCTTGCTCATA

ATTTCCCAGGGAGTAATTACAGGCAAGAAGTGCCTGTAAAAGAAATGTCATTTTAACACG

ATACAGTAAGTTTCAGCCAAATTAATTTGATAGGACACCGGTGAATAATACTGATATTCA

TATTGGTGAGGAGTCACATAGGCCCACTATATGGAAAGTTTATTCTAGGAGAAAGAAAAT

AGGATTAGGGAGTGCGAGTTAGTTACATGACGTGGCATAGTAGATTCTAACAAATTCTGT

TAGGGGATGGAGTATGTAAGGAGGTGAGGAGAGAGAAAAAAGGGAAGGGGGAATGGTGGT

TGTTTAGGGGAATATTTCTCTGGAGAAGGAGTCACAGACTCTGGTTGTCATTCTCAATTC

CTGTACTAGCTTTCTGCTATCTTTCATATTAAATAACACTGTTATATTGAATTTCTGTTT

TTCATAACACCGTGACCGTGAGTTCTAACATAATTTCAAAGACCTAATAATTTTGAAATA

GTTAGTTTGTTTCATATCTTCAAATAATAGATCATATCATCTGTACCATTTGAAACTGCA

TGATACAGAGTCAATTCTTCCAATCTTTGCCAAATGTTTCTTTTTGGGCCACATTGGAGG

ATATGGTACAATATTTGAATTCTTATTTCTTACTAATACCATTTCAGTATCTTGGGAAAT

GGAAAAGTATCAAGCTTATCATGTAAAATGGAATGCCCCCTGCTGGACGGTCCCACATCG

TCTGCCTCAATTCCTGGGGTGTAACTTATATATTTGTTGGTCAACTTCACTTAATGTCAA

TTGGTTTTAAGATAAGATTTAACATGATATCAAAGCCTATATCCCATCTTGGTCAATCGC

CTGTTCTGGTAGGCTTGTTTGTTCTGACAGGCATTTGTGAAGGGAGGGGTGTTGCAAGGT

CCCACATCGCCTGCTTCAATTCTTGGTGTAAGCATCATTGAGCAAAGAAACAGACAACCA

AACTTGCATGCAAATTTGATGACCTGCCATAGGTGGTATAGGGCCCTGCTGAGCTGCCAC

AGATGTTGACAGCATCACAGTTATAAACACAGCCAAAGTCCTAGCAGAAACAAGACCACC

CACAAAGTGCAATAGTTAAAAAGGAAAGATTAATCTGCATCGCACCTTAACTCACATAAT
GTATTCTGCCTAGTAATAGAAGCAATTAACCAAACAAAACATTTTCAAGTTAATGAAATT
CATATTTCATTCTCAAAATACAAAAACATTTTAGGAATAGGGGGAGGGGGGATGTTAGGG
ATGTCTTTAAAAACTTCAGCTATTGGGATAGCTGGTTCAAGACACAGTAAAATCCAATTT
GAATCAAAAAGTAAAGTCTCTCCCAGAGTTTCAAGCATAGGACAGAATCAAATGAAAAAT
AATAAATAAATAAATAAAACTTAAAGCACAAAATCAACAGAAACCAAAATGTTATGCAAT
CCACATATTCACACTTAGGTCATTAATTCTCAAGAAAAGGTATTGTTTGAGATACTCAGT
TGAGTTAAATTTTCTATCCACAATCATGCACAGATGAAGCTGAATTTGCTATCCACAATC
ATGAATAGATGAAGCTTGTCTAAGAAAATTTCAATGACATACTGCAGAAACTTGCAATCA
TCTTTCCAATCTACTCCAATCATTTCATCATCATTACCAATTTTCATTGAAGGGAAGGGA
AAGGCAAAGCAACAATTCAACTTAATTGTGCATACCAGATTTCAGATAGCTGAAAAATTT
TCTCCCATAAAATTCGGAAGGGATTAGCAGCACTTTGTCACTCAATTTCCATAGCAGAAT
GAAAGCTATTAAATATCTGGTGAACACAAAGACAAACTCTGTTGAAAGCAGATCACATGA
ATTAAGGGATGTGTGCAGAATGCACAGATAATACTAAGTTTAACACATCATTGACATACT
CAGAGATCAAAGTAGCAACTGTAGCACCACCAAGACCAAAAAGAAATATTA

>**Gmax_MSTRG.56420.1** GeneID=Gmax_MSTRG.56420;Strand=+;Pos=Chr11:1868428-1871028,
CCGTGATAAGGTCGAGAGTCAAGTGATTTAATTAGAACTGCCATAATCATGTCCATATAA
TTAATAAATGAAACAGAAACAGAAACAAAAACGCATTCCTTTGTGTTTTTCTTTTCTTTG
TATGAATGCATGCGTCTATGCTCTGCCTCTTTAAAATTAGCACAACCTAGAAAGAATCAA
TTTGATGCAAAAAATATAAAAGAATGGAGGTGACGTCGTTGTGATGATCAATCAAACTAA
TAATGAATCAGAGCAATATTCCTTGGTAGCGCAGGAACTGAAGAAGCAATTCTGATCTTG
TTCCTCAGACGATGATGTGAGTTCTTTGGCTCTTTGAACTTGGCCCTCCCAATTGGTGCG
AGCCAACACAATTAACATGGTGAACATACAAGAGGCTTGGGCGGCCAATAAACCAAGCCA
CAAGCCTTTGAAATCAAAGCCGGCAAAGAAACTCAACCTCACCGCAACGGGCATTCCCAC
TAGATAGAAGCAACCCAAATTTATATTCGCTCCCAACTTGGGCCTCGCGGTTCCCCTCAA
AACGCCACAAACCGTTGTTTGCGGACAGTTTCCCAATTCGCAGAGCCCAATGATGGGCAA
CACCATCGACGTCAAAGCAATGATCTCCGCGTCACGTGTGAACATCGAAGCCCACGCTTG
CCTCACCGAAACGGCGAAAAATAATGCCGAGAAACCCAAACCGTAACTGATACATAACCC
CACCAGTGCTGCGACTTTTGCCTTTTTGGGATTCTCCGCACCCAGTTCATTTCCGACACG
TGTGGAAACCGCAAAGCTCAGGGAAGATGGGAAAATGTATATCAGAGCAGTGGTTTGTAT

CAGAACCCCCATTGATGCAACCGTTGCTTGGGGATTAATCAATAACCCGCAAAGTAAAAT

CATGATTTCGTACCACCACCACTCGAGGCATACGGAAATGCAACTTGGAATTGCCAAATT

CAAAAGCGATTTCCAACCGCTCAAGATGCCCTTCAACGAAACACCTGGCCATGTTTTTTT

GTATACGCCGGAGACCCATATGTAGAGAATCAACGAAACAACGAGGTTAAAATTCGTCCA

AACAGCGCCTAAGGCAATGCCTTTGATTCCAAGCTTAAGCACAGAGACGAGAAAGTAGTT

GATGGGTACGTGAAGGAGAATAGACAGAGATGCGGTGTATGTGAGAGGTAGGGTTATAGA

TTGACTTCGTAAGTAGATTCTCAAAGGGTGTAGCAATGATTGTGCTACAAGATCGGGAAT

AGAAAATAGTATGAAAGATTGAGCTTCGGTGGCGATGTCTTCTTGTTGGCCACATAGGAT

CAATAATTTCTTCATGTTGAGCCACAAGAATGAAATCAAAACTGAAGTTAGAAGAAGCAA

GACGATTGTCCTCTGCATAGCGAGACCCAAAAGTTTGAACCTTTTGGCCCCAAAAGCCTG

GCCGCAAATGGGCTCCATGCCCATGGCGAGGCCCGAGAGAATAGAATAGCCCGTGATGTT

GGCGAATCCAATGGCGAGAGAGCCACCAGCTAAGGCAAGCTCACCCACACGACCGAGGAA

GAGCATGGAGATTACGGAGCGAGAATAGAGTAATAACCCGGTTAGCACCATGGGTAATGC

TATGTTGGCTATGCATTTGACTTCGTTGAGAGCGAGGGAAAAATGGGTTTTAGTTTGATG

GTTATTTTGGCCTTGTTCGAATAATGTTGTTGGGGGTTTGGGGATCAAAAGGGGGAGCAT

GTGAGCTGGCTCCTCATGAATTTTGGTTGTGGGCATGTTTGGATGGCCCTCATTGCTATC

ACACAAAGTGGAGGGAGATGACGATAACTGGCACATGGCTTGCACGGAGACAGAGATATT

TAGCTAGGGAGTACTAAGTGTTTGTGTGTAAGTCACAGAGAAAATCTTGGTTGGTTTGGG

GATTTGCATTTTGCAAGAGTGTGTGAGGAGAGGTATTTAAAAGAGATTCTTGGACGTATC

TGAATTTATTTATTATGGAGTATCCGGGACGGCCACTAATGGAAATGGAAAGATAGGGTT

GACAGAGTATTGGCGTTTCCGAGTAGTACCAACTCAAACGCCCCCTCCTCATAATTTTTA

TTTTATTTTAAAAGAAAGCTAATATTCAAAGCGTATTTTTACGACCGTCTAATATCGTTA

GTGCAGGGATTATTAGTGGACTTGGACGTAATGTATACTACTATAATCCATATAGAAGAG

CCTTGTAGGAAGGAATTACCAATTCAAACGAATAAAGGTCAAAAGTCTAGTAATTCCATT

CCATAGTATATTTAACTCCTCCGCTTAATTTGTTGTGTAACCTCACATTGCTTTATATTA

AATTCATGAACTGGCCAATAGCATGAAGAACGTGTTAGGATTTGATGTATCCGTACCAAG

AGGCAGAATTGAGTTTTGAAGGCTTTCACTTTATGAGGAGAGGACCCATTATGAAAGCGG

CACCTTGACTTACTCTTTCTAAGTTGAGAGTTTAATTTGGGTCCTTTATCTTTAAAACTA

GCTCGTGTGAAAAAAATGCCATGTCGGCATTCGTTGATATGTAATGGGATTTAAATTAAT

CCACTTTGTTTTAAACCCGTG

>**Gmax_MSTRG.65157.1** GeneID=Gmax_MSTRG.65157;Strand=+;Pos=Chr12:38510854-38511494,38512270-38512501,

TCGAGCAACAAGGAATGAATTGATGGTAATTACTACAACTCACATTGGTAACCATAGATA

CACAGATTATACTACAACATAAAATTAATTATGAGACACGATCTCATCTGTCCAAAATAA

CAATATTAGCAAAAAAAATGAAAATGTTCACAATTACTATATACTATAACTCAAGTTCCA

AAAAACAAAAGTTTGATTTTTCTAACCAAGAATGGTGCTTAGAGCAGCACCAGCCAGAAT

GGTCAGTGAATACAGCCCTAACCTCGACGGTGCTACAGACGACGCGCCGTTTGGTCTGGC

TGGCGTCGTTTCGTTCGACGCCCCCGACGACGACGACGACGACGGTGAGGGAGTTGTTGC

TGCCGGAGAAATGGTGGGAGTGGTGGCTGACGTCGACGGGGATGGTGGCGATGTTGCCGG

AGCCAGGTAGGAAGGTGGGTAAGTGGGTGCAGATGAAGGTGAAGGTGAAGGTGGGTAAGA

TGGAGAGAGTGAGGTTGAGGGAGGGATGGTGGATGGATAGGGTGGTTGAGATGTTGGGGA

AAGAGTGTTTGGAGGTGTCGCAATAGAAGGAGGTTGGGAAATGGGAGAAGGAGGTGTGGG

GATGTAAGGTGGTTGAGATGTTGGAGATGGGGTTTTTGGAGGGAGTGTTTGGAGGTGTTG

GAGGATAATAAGGAGGTGGCTCTCTCACAGCCAACACCACAATAATCAATTTCTGACCCT

TTTCACAATTACCATCTTTCCCACTAATGAAGTAAAATGGACCCGATCGATCAAACTGAA

ACTCCGATTCACTATCCTCAAACTTCTTGATCGGGTTCTTCTTGTTACATTTCTCATAAT

CATCCTTGTTCACCACCAACACAGTATCCGAGC

>**Gmax_MSTRG.70935.1** GeneID=Gmax_MSTRG.70935;Strand=-;Pos=Chr13:37706536-37706727,37707123-37707391,

AAATTTTCCTAGCATATTGCATCATTGTTAAAGTAGCTAGCACCAATCTTATTATAAAAC

TCAACAATATTGCTTATCATACATGATAATTCATTTCACTACATTCCTAAAGAAATTCTG

AATATAATTTACCATGACAGCCAAACCAGTGACAGAAAACCAAGAGTTTGCAAGAGTAGC

ACCAGCAAGCTGAAGATCACCAAGGTGGCCAGCGAAAATTACAGAAACCAACACGATTAA

GTAATAGAACAAGTTTGTAAGGAACATAGGTAGTGAAAAAAGTAATTGATGTTTGGCCTC

CTCCAAATCCAGGATTTTGCTGTTCCACCATCGGTACTGTCTTGTGTTTTCTTTATGATG

AGATACATCTAATAGAGGTGTGAAGTGTGTTACTGTGCCATCTAAACTCCTGCTTTTCAG

CATCTTCTATAATCTCTTTGCTCCTATTTTCTATGTTCAAT

>**Gmax_MSTRG.64519.1** GeneID=Gmax_MSTRG.64519;Strand=-;Pos=Chr12:35571070-35571542,

CTTTTTCCTTCTACTTCAAATTGCTGCTTAGACAACTAGAATTAATCCTTGGCAGTTTTA

ATTTTTAATTCCCTACCTCATAATGGAACAACGAAGTGTTAAACTACCTTGTTGAATCTC

TAGAATTAGTCCTTATAAATTGGTTTCAATAAAAAATAATAATAAAGAGAATATTATGTC

TCTCTTGCTCTCCTACATAAACAAGTTGTTCTTCAATTTAGTAGTGGTTCCTTCTTGTCC

TCCCATTTGGTCAGCCTCTTGGCTGCTTCCTCTACCTACAATGAAGAAAATTCTCATTAG

ACAGAAAAATTAACTTCCCCATTGCATGCATAGTTTTACGGAAATAAATTATTGCAGCTA

TGGTTTTTTACCTCCTTAGTCCAATCCGTTCTAAATGTGACCCACAGTAGAATAATTGTT

TGCATAACGGTGCCACCTAGCATTCCCAGCCATATTCCCTGCCACAAAAAAGC

>**Gmax_MSTRG.10873.1** GeneID=Gmax_MSTRG.10873;Strand=+;Pos=Chr03:508607-509036,

CAGAATTCCATTGGCAAAGCGTATGAGAACAGTGAACACAAGAGCATAAGCAGCAAGTTC

CCTTGAACCAATATGACCAATAAATGCTTGGCTTATAACATTGATACCAAAGGTGGAGAA

CCTTGTAAAAATGCCTGGTGCTGCCACTACCCACATCACCTTGCTCTCATTCCACACCCT

CTTCACCAATGATAATTCCTCTTCTTCTTGTTCTGATGTTTTTTTCTCTTTGATCAACAA

CTTCTCCTTCACACCCCCCTGCATTTCTCTATTTTTTTTTTTTTTTTGCTTTGCTAGTAG

AAGGAAGAACAAACTAAGCAAGTGATGAGGGAGTGTAGGCCTGAATTTTTTTCTATTTTA

AACCAAAAAAAGGTGTTGGCATGGCTTTAATTAATACAATAAAAATAAATTACTCGCGGT

GGTTGCATGC

>**Gmax_MSTRG.79338.1** GeneID=Gmax_MSTRG.79338;Strand=+;Pos=Chr15:8401984-8402209,

GTTTCCGAGGTGGCCGGCGAAGGCGCGAGTAACGTTGGACATGAGGTTGTTGAAGACGTA

AACGAGGATGGCGGGAGCGGCGAGGGGGAAGAGGAGGTTGAGTTCGATCCATGTGGCGGA

GAGGATGCGCTTGGACCAGGGTAGGGTGGGGTCAGAGAGGACCTCTTCGAGACGGGAATC

AGCAGAGTGATGATGATGATGCGTTGAGTTTATCAAGGGTTGGCGT

>**Gmax_MSTRG.27311.1** GeneID=Gmax_MSTRG.27311;Strand=-;Pos=Chr06:7114702-7115046,

AGGGGACTAGCTAAAAGAAAGAAAAGTAAGCGATGTGGGACAAATAAATAACGGGGAAAT

GAAATAAATATTCCTTTTTCTCTACAGTACAATCCAAACCCAAGCAATCTCATTTGGAAG

TGTGTATAATGAGAGGGTCTGTTTCAAGAGAAGGTGTCTTGGTAATAATCTCATCATGAT

GATCCTCCGTGTCCATGACAAGAATCTCTGGAAGACGAACATTGTTACACTCCGTAGTAG

GTAATTTGGAAGGAGCAGAATTAGCATTAGTAAGCTCGTTGGCCCTTTGCACTTGGGCGT

TCCAGTCAGTGGTGCAGAGAACGAATATCATGAGAGCAGCACATG

>**Gmax_MSTRG.34861.1** GeneID=Gmax_MSTRG.34861;Strand=-;Pos=Chr07:19197000-19197256,19198043-19200043,

TTAACAACAAATACCAGACTATAAATTACTGAATAAATAATGTTAATTGCGTTAAGATTA

ATGCTAACCATTAACGTAAAAAACTATTAAAAAATTGTAATTACTACACAGTACACACCT

AACTAACTTGAATAGCTGCAATGGACAAGTCAGAGTACTTATTCTTACATGCATTGCAGC

TACCTTTAGCAATGAAAATATACAAAATTGTGCAATTTCCTAATAAATACAAATTTCCAA

CAAAAAACAGAGGGTCAAAAAGACTACCCTCTAAGCTGTAGGCTGGTCATTCAATAATTT

TCTCCCTCCCTGTTCTATTGGCCATTCAATCAATACTTGAATCAGCAAGATTTTGGCAAT

CGGTTAGCAGTAATTGGCTGCTACATTTCAATAATTTACTATAGTCCTGCAGTGATACTG

GATTCTCTTTGCAGGAATTTAGTGCCCTCATATAAGAGAGCTCTTGAGAGAATCATTTTC

TGATCATTCTGAAAATGGTTTTTTAACCTGAGGAGTGAATGTTGGAACTTCTGAGTACTG

AATGCATGTTGGTCAATCTTCTGCTCCATCTCTATACCAGATTGTATCCCAAGGCCCACT

TTTACTGCTCAACCTTAAGAAAACAAAAATATTAACTCAATATAAATCTTATGAAGAAAA

TTTTGATCATGTTATCCATAGAGTATCCTGCCCCTACCCAATAAAATACCGACATAAAAG

AGAACAAATAACATGCGACTTGATGTCATGTTACATTCATCCAACATGTCCTGTATCATT

TTGCCATTGTTCTTTGAGCTAGGATTTGAATGAGTACGACAATGGGTAATAATACACAAA

CATGAAGTTTCACAATTATCACCTTCCATATTAATTAAATACTTCACCTCCAAACTCCAG

CTAGAACACGCAAAGCCATGAAAATAAACAATCCAGTCCAGACTCCAGGAAGCCCTACTG

GAGCCGCCACCAGAAGGAAAGTTGAAGAAACTAGTCCAACTAGAACCTACCCATAACAAA

AGCCAAAAGAAAAAAAAGAGCACGAAATAATGAGTTAAGAGGGAAAAATAATATGGAATG

TTTAACTATCTTTCAACCATTGTAGGAACTTACCATCGAGTAAGCAGCATATCCAAAGTC

TGATACCCCATAATAAATCCCATCAATAACAAATGCCAAAGCATTCACTGGTTGAGATCC

AGCAACAAACTGCACATGTAAATAAATTTCAGATATTCCAATAGAGGTTACTATTTACAC

ATGCTTCCACAGAAAGTATGGAGAACTGCACTTGCATATAATGAACATTTATTAGCCCTA

CTAAAAGGTTACCATGGCTTAAACACCCATCAAGCACAAAAATTCTGATACTATCCAAAA

ACATTAGCTTTCAATGTATTCATCCACATTAGAGGAAGGACAGACAGAAAAAAACAACAT

AAACAGACATCCAAATGAAATGCATTCTTCTACTTTCTTGAACGAACTCCTTTCCTGGTA

AATGCACTATAATACTTTCAGACACATGAATTTTAATTTGGAGTTGGTACTCATGACTTA

GACACTCAGACCCACTTCAGCACAGGCTGAGTGTCTGATTCTTAAAGTCAATCCAAGTCA

ACTAGGGAGTGGCATATAAAGCTTACAAAATGACAATAAAGGTAATTTTCTGCAAACAAC

TCTGATTTCTTACCCATATACCTGACCAAGCAACATCCAGAACTTCAGAATCTGTGCTAA

ATAAACTAGAAAACGCTCCAAACCCAAAGAATAAGATTATTGACAAAGTGATTCCTGCTC

CTAAACCAATCTGTTTAACATGCAAAAGAGAGTAGGAGAAAAGAATTCTGGGTCAGTATA

AGTTCCAATCTCACTAAATGAAGGTACGGGGAATGAAGAGAGCAATTTATGGTGACAACC

TGTATCACTCTATATATAACAAGGCGTGCTTGCTCATAGTTTCCCAGGGAGTAATTACGG

GCAAGAAGTGCCTGTAAAAGACCAGCAAGCGCCAAAGCATCATTGAGCAAAGAAACCGAC

AACCAGACTTGCATGCAAATTTGATGGCCTGCCATAGGTATAGGGCCCTGCTGAGCTGCC

ACGGATGTTGACAGTGTCACAGTTATAAACACAGCCAAAGTCCTAGCAGAAACAAGACCA

CCTACAAAATGCAATAGTTAAATTAGTTATAAAGTAAAGATTAATCTGGCATTGCTCCTT

AACTCGCATAATGTATTCTGTCTAGTCATAGAAGCAGT

>**Gmax_MSTRG.55222.1** GeneID=Gmax_MSTRG.55222;Strand=-;Pos=Chr10:49055328-49055865,

AGAATAATCACCAAACCATTACTGCTGAAGGGATAGCGAAACGAAAGAACTCCCACATTC

CTTTGAACAGCTCCATAGAAATCGGTGCGCGGGTTTTTGCACATGCAGAAGAGTATCTCA

TGTATAACACAAGAAAAATCACATTTGACCATATTGAAATGCTCACAGCCAATGCTCCAC

CAACATTACTCAGACTTGTCTTGAATACCAAGGCCCAACAAAGAGGTACATGAATAATGA

GTGTGACACAAGAACTTGCAAACATGGGAAGAAGCAAACTTTGGATTTGGAAATATCGAA

CTAACGGCTGTAGAATTGCATATGCAAAAAGTGCTGGAACAAGCCAAATTGTGAACTTCC

CTGCTTCATGGGAAATTAGAGGGTCCTGGCCTATGAAAACTAGTATGGTTTCCATGTTGA

TCCAAAGGAGAGACACCGGAATAGAAACCAAAATGAGAGAGAATATAGCAGTGTATGTTT

GTATTCCAATTCTTTGATATTGTTGGCCTCCATAAGCCTGTCCACAAATTGTTTCCAG

>**Gmax_MSTRG.104177.3** GeneID=Gmax_MSTRG.104177;Strand=+;Pos=Chr20:36605682-36605828,36608596-36609241,

AAATACCTTGGATGAATGAGAAAGAAGCAGAACAATTATCGCCATTTCCTTATTTTACTG

CATTAGCTTAGTTACCTTAGTATAGCAATCCTAACAGTTCTTGCCTCAAATTTTTCTTTT

CAGGAATATTCGATGGATTTTGCACATTAGTAGGTCTCAAAGCTTAAGAAGAAAGCAGTG

AGATGGGAAAGGAAGAGACTTACAATAAGACTGAAGCCAGAGACAGCACAGAGAGAGATG

GCAATGGCTGTGCTAGAGAGAGCAAGCTTACCCAAGTGACCAACCATCATCATTGAAATA

ATCTGTAGAAAATACTGTGACAAAGTCACAGTAATCATAGGAGCTGCTAGATAACCCACT

CTTTTCATTTCCTGAGAAAAGACAGTCCATGTTATGGCTGAAGCTGAAGGATTCTCCGGC

TCTGAGTCCTTATCTAAGAGACTATTTTCCATATCCTCTCCAAAGGGTCACAGAATCACT

GCACCTTGTCGAAACTGGTAGAAATAATGGCAGGGCTTTGGTGTTTTGTATGCTAAAAGG

TTTAAATATTTACAAGTGGCAGCAGCAGTAGTTCTTCCAAGGTTTAAATATTTGTTTCAA

TAATGACTGTCTCAGAGTCTCTCATGGGCTTCCCCCTCTTATGTAGACGGAACAATATTT

CAAGTAAATTTTAAAATTTAAAATCATTCCATCTAATCTAATTTAATCTAAATTTTAATT

TTGATTTCTTATAATTTTAAATCTACAATTTTTAATTTTTTATTTTTAAATTAAGACAAT

TTAGTCAGAACAG

>**Gmax_MSTRG.104177.9** GeneID=Gmax_MSTRG.104177;Strand=+;Pos=Chr20:36605722-36605828,36606791-36608312,36608599-36609101,

GCCATTTCCTTATTTTACTGCATTAGCTTAGTTACCTTAGTATAGCAATCCTAACAGTTC

TTGCCTCAAATTTTTCTTTTCAGGAATATTCGATGGATTTTGCACATACTGTGCCAAGTG

CAGAGCCATAAAGACTTGGAAGTGGAGGCAAACCTGTTTTTCCCAGTTTGTACAACTTGT

GATGAGAGATAGCATAATTGTTTGGCAGAAGGCACCAGTCAGTATTCCAATCCAAAGGCC

TTTTCCTCTTAATTGTAGCCAGAAACCCAATATGGCAGCCATTGGAATTCCCACAACATA

ATAAGCTCAAAGGTTTACATATGCTCCTATGTGCTGCCACCCACATCCTCTAGCAATACC

TATAATGAAGGCATAACTCAGATTCCATTGCTTAAGAAGAGTAATGTGCATTGAAAAATA

GTTCAAACAAGAACTAACCTGAAAGGGTACCGTGTAACGTGTCTACTATAACAGATATAC

TTAAAAGAGGAACCATATCTGTGACATAATCCACCACATCCTGCTCATTGCTAAATACAT

AACCTAAAACCTGCCTGCTAGCAAAAATGATTGAGCTCACCAGAATGGCCTCAGAAACTG

CTAGAGTCATGGCAGCAGAAACAGATACTCGTGCTGATTGTGGACTTCCAGCTCCTAATG

CATTTGAAACTCTAGTGCTGAACAACCCGCCCAAAAGAGAAACCAATCAGAAAGAGGAAA

AAATTCATTTACTATAATATCATTCTACATGAATACGAATATGCACAAATTGATGAGCTT

AGGACTTGTGTGAATCAAAACCTTGCTGCTGAGCCAATTGCTTCTGGGATTGTGTAGATA

GTTGTGATGATGGATAAACTGGTCAGAACATAGTTAACAATAACAACAAATTAGCAACAC

TTGCATGAATGGAAATCAAAATTTGGCTGAGTAACAATTTCAACACAGATTTTAAAACAC

AAAGCCATTCATGTCTGAAGGACTAGGATAAGTTGTACATACCATATGGATAAGACTGAA

GTTTCAAGCTCTGGATTTGGTAGAAGACCAGAAAGCAAGGTTAGCAGCTCAAATGACCAC

CACTCAAGGCTGCAACGATCAAATAAGTAAGAAACATACCATGGTTGAAAATTAAATGGG

AAATAAAAACACAACACAAAAGATAAAATATAAGAGGAAGAAACTCACCAAACCATTCCA

GCTGAAGGAATAGCATAGGTGAAGAATTCACCAATCCCATGGAATAGTTCCATTGAAATT

GGGACTCGAGTCCTTTCACATTCAGTAGAGAATTTCATATATAAGCCAAGTAAAATCACA

TTCAGCCAGTATGAAGTACCAATTGAAAATGCTGCTCCCAAGTTACCAAATCCAGATTTA
AAGACCATTAACCAACTGAAAGCCACATGGAAGCAAAGAGTAATGGAGGAACTTATGACA
AGGGGACTGATCAAACTTTGCATCAAAAAGAATCGAATCAAGGCCTGAAGTGTTGCATAA
GCAAAGAGAGCAGGGATCATGCACAAAGCAAATTTTCCAGCTTCTTGTGAAATCGAAGGG
TCTTGGCCAAGAAATATGAGTATCTTCTCCAAGTAGACCCAGAAAAGAGTCAGAGGAAGA
CAAGCTAAATAGGTCTCAAAGCTTAAGAAGAAAGCAGTGAGATGGGAAAGGAAGAGACTT
ACAATAAGACTGAAGCCAGAGACAGCACAGAGAGAGATGGCAATGGCTGTGCTAGAGAGA
GCAAGCTTACCCAAGTGACCAACCATCATCATTGAAATAATCTGTAGAAAATACTGTGAC
AAAGTCACAGTAATCATAGGAGCTGCTAGATAACCCACTCTTTTCATTTCCTGAGAAAAG
ACAGTCCATGTTATGGCTGAAGCTGAAGGATTCTCCGGCTCTGAGTCCTTATCTAAGAGA
CTATTTTCCATATCCTCTCCAAAGGGTCACAGAATCACTGCACCTTGTCGAAACTGGTAG
AAATAATGGCAGGGCTTTGGTGTTTTGTATGCTAAAAGGTTTAAATATTTACAAGTGGCA
GCAGCAGTAGTTCTTCCAAGGTTTAAATATTTGTTTCAATAATGACTGTCTCAGAGTCTC
TCATGGGCTTCCCCCTCTTATGTAGACGGAAC

>**Gmax_MSTRG.96651.1** GeneID=Gmax_MSTRG.96651;Strand=-;Pos=Chr18:57124296-57124508,
AGAAACATTGGTGAGAGAGATTGCAATAGCAACACTTGAAAGAGAGAGCTGGTTAAGATG
GCCAACCATTACCAATGACACCACTTGCAAAAGATATTGCAACACACTTGCAACCACCAT
TGGAGCTGCTATAGTACCCACCTTCTTCAGCTCTTCCCTCATTGCTCTCCTCCTTTTCAT
CCATCCCCACTTTCCTGTCTCTGAACCTTCTTC

>**Gmax_MSTRG.55226.1** GeneID=Gmax_MSTRG.55226;Strand=-;Pos=Chr10:49060244-49061429,
TTGCATTTTAAGTGGGAGTACTCTAATTTTTCATTGCTATATACAAGGATGTTATTGGAA
ATTGAAGTGCTGCTCGTGAATTAATCCAATTGCTTTTACTTCACGTTACAGAACCTTAAC
CAAAATTGGATTGAGAAAAAAGAACAAATCAATATCATTACAAATGTCTTGTCATGAGAA
CCTTTTCCTCCTCTAACTCTAAAATTTGATCTTTTCTGCCACCAAAGCTAAACGATTTAC
CAATACCAACAAAAGAAGCTGACAGATTTAAGGAATCTTCTCCATTTGCTAAAACTAATC
CATCTTCTACAGCAAAATTTCTCTGAAATATCCTTTCCCATGCTTTAATTGCCTTTCTCA
GGACACTTGAGCAATGAGTACATTTCAGTCAGTAGTACATGTGTTCTCTAAAAAAACAGG
CTAGTAGTGCATGTCCAAAACTGAATCTTAATAAGATTGCATGAGTTAAGATACTACTTT
TCTTTGACTGTCAAAACAATTTCTCTAGTTTAAACATTCAATTCAAAGGGTTTGGTTTGA

TATATGAGAAAATATAACATTGTGAACATGGTGTTTTAGGACTGTGCCAAGTGCAAAGCA

TGGACTTGGAAGTGGAGGCAAACCTGTTTTTCCCAGTTTGTACAACTTGTAATGAGGGAT

ACCATAACTGTTTGGCAGAAGGCACCAGTCAGTATTCCAATCCAAAGGCCTTTTCCTCTT

AATTGTAGGCAGAAACCCAATATAGCAGCAATTGGAATTCCCACAACATAATAAGCGTCA

AGGTTTACATATGCTCCTCTGTGCTGCCACCCACATCCTCTAGCAATACCTATAATGAAC

AAGTATTACCTTACAGTATTATATTTTATGGCACAATTTCAGACTACATTGCGTAAGAGT

GATATGCATTGAAAAATAGAGAAAAGAAAGAACCAAACCTGAGAGGGTATCATGTAAGGT

GTCCAGTATAACAGATATACTTAAAAGAGGAACCATTTCTGTGAAATAATCCACCACGTC

CAGCTCATTGCTAAATGCATAACCTACAACCTGCCTGCAAGCAAATATGATTGAGCTCAC

CAGAATGGCCGCGGATGCTGCAAGAGTCATAGCAGCAGAAACAGACACTTGTGCCAATTG

TGGACTTCCACCACCTAATGCATTTGAAACTCTAGTGCTGAGTAAC

>**Gmax_MSTRG.72921.1** GeneID=Gmax_MSTRG.72921;Strand=+;Pos=Chr14:228884-229134,

GACCATCAAAAATATATGCCAGTGCATTAAATGGTTGAGAAGCACTGACAAACTAGCAAC

AATCACAGATAACAGTGTAATTCTAATGCGGATACAGACAAATGCAAACAAATACATACA

AATCAATCACCAATACCATACATACCAACGCCAGAGTTTTGACCACCTGCAAGACTTCAG

AGTCCTGAGTAAAAATAGTGGCTAAAGATCCAAACGATGCACCCAGAATTGCAGTCAAGC

AAATACCCATT

>**Gmax_MSTRG.6399.1** GeneID=Gmax_MSTRG.6399;Strand=+;Pos=Chr02:7927886-7929336,

GGAGTTGATCCAGCATTTTGACCAAATTAGAATAATATAATAAAACAGTCATGCATGGAC

ACTCCATTATTACAAACACTCCATTATTTTTCTTGTAACATTTATATTATCTGTTACTTA

TTTCTTTGCTTTCATTTATTTCTTTGGCTCTCTAGATGTTGACGTAGGTGTCTCGTAACA

TTTCTTTGCGAAACCATAGTTTGGTGACTTATTTCCTACGGAAAATCAAAATTATATATC

TATGTCTATGTGAGACACAGCTGACCAAACTAGCTTTAAGCACTCGTCATGTGATATGAT

CCATTTACGTCTGGTGGTTTAGCATCAAATACTCTTTCCCTTGCCATAATTGCCTACAAA

AATTGCACAAATCCGACACCTAATTAACACCAAACATACAATATTTCTACATTGCAAAAA

AGAAAGCATATTGGATAATTAAGGATAGTGTCAAATTTATCAATTGTGAACCTGTTTTTT

CCAGTTGGTAAGAGCAGTAATAAGGGAAAGGAGAATCGATTGGACAATGGAGCCAGTGAC

AATTCCAATCCAGAGGCCCTTTGCTCTGAAATGTGCAACAAAGCCAAGTAGGATACCCAC

AGGAATTCCTACCAGATAAAACGCTCCCAGATTGACATAGGCTCCTAGATGTTGCCACCC

ACTTCCTCTAGCAACCCCTGATTATTTTATCTATTTACTTATTTATTTTCGTTGCAGGTT

AGCAAAATAATAATGATAGTTACTGACAAAATATAACACACCAAATGCATTGGTAACACA

TTTTCTAACGTTATCTTTTTAACAAACCCGCTTTTGAAATTTAGGTGACTCCATCCTAAC

AATTTAGAAATGTTGATAACATATTTTTTAACACTTATTTTTTTAACAAATTCTTTCTTA

CTAGTTAAAATTTAAACCACTCCAAACAATTTAGGAATGTTACCCCCATCAAATAAAGAA

TGTGACTTACAACATATTTAGTGAGATGAATTTTACTCTAATGATTATGGGAGAGGCTGC

GTCTGTTAAAAGTATCAAAGAGTGGTGCATTTCTTCTAGTTTAACCAAAAAAGAGTGTAT

TTCTAGCAATTTTTTGTTCTTGTTAGCTTTATTTTACTATGCAGCATTCAAGAGTAACCT

AGTTGGTTGAAAGCACCAACGTACAATTTAATTTTCAAAAAGCAACAAGCAAAAAAAGAA

TAGGAGAGAATAATTAACCTGAAAGAACTCCTTGTAAGCTGTCAGTAAAAATAGAGAGAC

AGAGCAGAGGGATCATAACAGCCACATAATGAACAACCATACGGTCATCACTATAGGCAT

AACCCAAGATATGTCTGCAGCCGAATAGTGTTGCACTTACAATGAAACCCTCTGTGACTG

CAAGGAACATCGTTGCAGAAACAGCCACATGAACTGCTTGTGGATTCCCAGCTCCTAATT

CATTCGAAACT

>**Gmax_MSTRG.104177.10** GeneID=Gmax_MSTRG.104177;Strand=+;Pos=Chr20:36605725-36605828,36606807-36608480,36608599-36609241,

ATTTCCTTATTTTACTGCATTAGCTTAGTTACCTTAGTATAGCAATCCTAACAGTTCTTG

CCTCAAATTTTTCTTTTCAGGAATATTCGATGGATTTTGCACATAGCCATAAAGACTTGG

AAGTGGAGGCAAACCTGTTTTTCCCAGTTTGTACAACTTGTGATGAGAGATAGCATAATT

GTTTGGCAGAAGGCACCAGTCAGTATTCCAATCCAAAGGCCTTTTCCTCTTAATTGTAGC

CAGAAACCCAATATGGCAGCCATTGGAATTCCCACAACATAATAAGCTCAAAGGTTTACA

TATGCTCCTATGTGCTGCCACCCACATCCTCTAGCAATACCTATAATGAAGGCATAACTC

AGATTCCATTGCTTAAGAAGAGTAATGTGCATTGAAAAATAGTTCAAACAAGAACTAACC

TGAAAGGGTACCGTGTAACGTGTCTACTATAACAGATATACTTAAAAGAGGAACCATATC

TGTGACATAATCCACCACATCCTGCTCATTGCTAAATACATAACCTAAAACCTGCCTGCT

AGCAAAAATGATTGAGCTCACCAGAATGGCCTCAGAAACTGCTAGAGTCATGGCAGCAGA

AACAGATACTCGTGCTGATTGTGGACTTCCAGCTCCTAATGCATTTGAAACTCTAGTGCT

GAACAACCCGCCCAAAAGAGAAACCAATCAGAAAGAGGAAAAAATTCATTTACTATAATA

TCATTCTACATGAATACGAATATGCACAAATTGATGAGCTTAGGACTTGTGTGAATCAAA

ACCTTGCTGCTGAGCCAATTGCTTCTGGGATTGTGTAGATAGTTGTGATGATGGATAAAC

TGGTCAGAACATAGTTAACAATAACAACAAATTAGCAACACTTGCATGAATGGAAATCAA

AATTTGGCTGAGTAACAATTTCAACACAGATTTTAAAACACAAAGCCATTCATGTCTGAA

GGACTAGGATAAGTTGTACATACCATATGGATAAGACTGAAGTTTCAAGCTCTGGATTTG

GTAGAAGACCAGAAAGCAAGGTTAGCAGCTCAAATGACCACCACTCAAGGCTGCAACGAT

CAAATAAGTAAGAAACATACCATGGTTGAAAATTAAATGGGAAATAAAAACACAACACAA

AAGATAAAATATAAGAGGAAGAAACTCACCAAACCATTCCAGCTGAAGGAATAGCATAGG

TGAAGAATTCACCAATCCCATGGAATAGTTCCATTGAAATTGGGACTCGAGTCCTTTCAC

ATTCAGTAGAGAATTTCATATATAAGCCAAGTAAAATCACATTCAGCCAGTATGAAGTAC

CAATTGAAAATGCTGCTCCCAAGTTACCAAATCCAGATTTAAAGACCATTAACCAACTGA

AAGCCACATGGAAGCAAAGAGTAATGGAGGAACTTATGACAAGGGGACTGATCAAACTTT

GCATCAAAAAGAATCGAATCAAGGCCTGAAGTGTTGCATAAGCAAAGAGAGCAGGGATCA

TGCACAAAGCAAATTTTCCAGCTTCTTGTGAAATCGAAGGGTCTTGGCCAAGAAATATGA

GTATCTTCTCCAAGTAGACCCAGAAAAGAGTCAGAGGAAGACAAGCTAAAGTAAGAGAGA

CAATAGCAGTGTATATTTGAACACCAAATTTTCGATATTGCTGTGCTCCATATGCTTGCC

CACATTGAGTTTCCAGTGCACATGACATTCCAAACTGTTTGACATGTACAAAAAAGACAA

AGTGCAATTAATTCTTATAATTTTTTTAGAGACCATAGTAGGTCTCAAAGCTTAAGAAGA

AAGCAGTGAGATGGGAAAGGAAGAGACTTACAATAAGACTGAAGCCAGAGACAGCACAGA

GAGAGATGGCAATGGCTGTGCTAGAGAGAGCAAGCTTACCCAAGTGACCAACCATCATCA

TTGAAATAATCTGTAGAAAATACTGTGACAAAGTCACAGTAATCATAGGAGCTGCTAGAT

AACCCACTCTTTTCATTTCCTGAGAAAAGACAGTCCATGTTATGGCTGAAGCTGAAGGAT

TCTCCGGCTCTGAGTCCTTATCTAAGAGACTATTTTCCATATCCTCTCCAAAGGGTCACA

GAATCACTGCACCTTGTCGAAACTGGTAGAAATAATGGCAGGGCTTTGGTGTTTTGTATG

CTAAAAGGTTTAAATATTTACAAGTGGCAGCAGCAGTAGTTCTTCCAAGGTTTAAATATT

TGTTTCAATAATGACTGTCTCAGAGTCTCTCATGGGCTTCCCCCTCTTATGTAGACGGAA

CAATATTTCAAGTAAATTTTAAAATTTAAAATCATTCCATCTAATCTAATTTAATCTAAA

TTTTAATTTTGATTTCTTATAATTTTAAATCTACAATTTTTAATTTTTTATTTTTAAATT

AAGACAATTTAGTCAGAACAG

>**Gmax_MSTRG.91143.1** GeneID=Gmax_MSTRG.91143;Strand=+;Pos=Chr17:40283857-40284096,

GCTCAACCAAATGGTGCTCAATCATGTGAAATATCCATGTAGAAATCATAACAAGCTGGC

CTATAATCTAAAATTAAATTTCCAAGTCCTCACTCAAATACATGTCGAAAAATTTAAAGT

TCTTCATTTATTGGTGCCACTCAATAGAGTCTCTGTCTAATTCTCTACTATGATAAAAAA

AAATCATTGATCATATGAAATTGCTCCTCCCCACTTTCTCACGCGGCTTTCTGCTTCTTC

>**Gmax_MSTRG.21812.1** GeneID=Gmax_MSTRG.21812;Strand=-;Pos=Chr05:4910236-4912125,

GCGAGATCACAGAAACAGAGTCTGATTTTGATACTATGTTAGATTTCATCTTAAAATTAA

TTGGCTTTAAGTAAAATTATTCAACAGATATATAAATTACACTCTAAGGAGTTAGACAGC

GATGTGGAACTTGGTATTTCCCAACAATTACAAACACTAGTGAGTTTGAAAAGGGACTCA

ACATATTGTACATGGGAGGGAAATATAAGGTTAGTCTGACTGTGAAGGAAGAAAGAAGGA

AAATGGGAAGAGAATCATCTGCTAACAAAAAAATTAATAATTAATTTATCTATAGAAAAA

AATTTGTACATGAGAATGATCACCCGGTTTTGACACTTGATTTTATCTACGTGTTCGAGA

CATGTCTAATTCTTTCTCAGAGGTATCTTTGCACAGTTCATTTACCTTATTTCTTGAAAC

TTGTTCACGTACCTGTGCGGTACATAATAACGTTTGTGCCTTGGTATATAGCCTCTTCGA

CCCAATTTATTCTCACAATAAATGTTAACAACAAAATCAAGGAAGCAACAACACCAATCA

AGAATCCTATAATGAGTCCAGCAAGTCCTAACCGAAGCTTGAAGGCAAAAACCGCACCCA

ATGGTAAGGCCAGGAAATAGAATCCACCAATATTGGCATACATGCCAAGTCATGGTCTTG

CAGTCCCTCGCACCATTCCTCCACAAACCGTCACAGGAAAATTAAACACTTCCACTAGAG

CAATCACCAACATTGTCTTCTTCAAACCCTTTACAACCCCTTTAACATGGCTAAACAAAT

TCCCTCAAACCCTTCTAGCAGCCACCATCATGGAACCTCCAATGCAAACCGATATAACAC

TCACTGCCAAAGGCACACGTGCTGATTTATAAGCTTAACCAGCACTACGAGTGGAAACAC

AAGTGGCTAAAGAAAGCATCACCGAGTAAAGCAAATAGTCAAAGTTCAACACTAAGGCTA

GAACCCCCACTGCTTGTTTTGCATTTGCCAAGTGGCCAGTGAGCAAGACTAGAATCTCAT

AACACCACCACTCAAGGCACGTGTTGAGGCAGCATGATCCACTAAGCTTGATTAGCCTAA

TCCAATCCATCATGTTCTGATCCCACCACCCTCCTTCCTTCCACAACATGCTCCCGTTTC

TTCTCTCAAGAATCACAACATATATCGCCAGCATAACCATAACCATGAGATCGTTGATCC

AAACCGCAATTGAAACACCTCGGAGTCGCATGGTCTTGGAGAGTAGTATGTTGACGGGTA

TGTGAAAGGCTAGTGTCACAGCAGAACTAAACATGGTGGGAAGAGTCATGCACTGAGAGC

TGAGGTAGGCTTTTAAGGGACAAAGGAGTGAAGTGACCAACAAGTCAGGTGTGAGATAGG

AAACATATGTTTTGGCCACTATTGAGATCTCTTGTTGTTGGCCAAAACGGATCAAAATCT

TGTCAACCTTAAGCCACAAGAAAGACAAGGGAAGTGTTACCAAAAGCAACAAGAGGGTTG

TCATGAGAAGGGTCTTGTGAAGGAGCCGCACGTTCTTGGTTTCCATGGCGCCTCTTAGAC

CGTTCAAGACAGAGAAGCCAGTGACATTAGCAAAACTGAACCCGAGTGCGCCACCGGCTA

AGTTCAGCTCCCCAAGGTGGCCAAGAAAAGCTGTTGTTATGGCTGTCTTGGCGAACCTAG

CCAAGTTCATATGGCCACAAGTGGAAGGTCTATCCCTCGTTGAACTCTTAGCTCTTCTAG

TACCATCTTTATGAAATTGGCCTTATCGTTTGTGATTAGCTTCTCTCCTTGTTTTGATGT

TGCTGACATTTTTTGCTTAGATAACGTTTTGGTGAGATTGTTGCAGAGTTCCACTAGCGC

GTTTCTTTTATATAAGATTGGTTAAGGTTG

>**Gmax_MSTRG.10866.1** GeneID=Gmax_MSTRG.10866;Strand=+;Pos=Chr03:485824-486321,

CTTGACAACAGGCCCAAGATCTTTGAATGCTAAAAATGAGAAACCTTTCCATGTTTCATC

GCACCAACCACACGTAATAAATATTAATTGACCAATATTAGGAATCCAGAATGCCAAAAT

TGTTGAAATCATTGCACCAGGAATCCCAAACTTGAATTGTATAGTCAATAGCCAAGAAAG

AAACACATGAATGATTATTGATAAAGCTGCCAAGAATGCAATTACGACATTCTTGCTTTG

AGATTGAAGGAATGTCTGGCAATTGAATGAGACAATATAAGCAAATAAGATAGGAATTGA

CCAAAGAGAAATGGTTCCTGCCACTTGTGCTATGTTCTCATCTTGGCCTAAGAGCATCAA

TATTGGGCTTGTGAAGATGAACACAGGAAGAAGACAGAGTGCAGTTAAGAACAAAACTAT

CGATGATCTTTGAAGATACACTCCCATCATGTCATATTCTTTTGCACCGTATGCTTGTCC

ACAAAGTGTTGATAACGC

>**Gmax_MSTRG.5537.3** GeneID=Gmax_MSTRG.5537;Strand=+;Pos=Chr02:3724244-3724497,3724528-3724944,

GGAGCCAGCAATGAAGGCCTTGCTGGCGGAGAATGCGGCGGAGGGTGGCGGTGGTGGCTC

GGCGCCACCTGCGGGTCCGGGAGCGGGTCCGGCTGTGCCTGGTGCTGGTGGAGAACTAGC

GGAAGGGACTGGTGAGGTTGCAGGAGAAGAAGCAGGTGTAGGTGCAGCTGTTGGTGTTGG

CGTTGGTGTTGTTGCTGGTGGTGATGTTGCTGGTGGAGGAGTTGCTGCTGCTGGTGGTGG

TGTTGCTGGTGGTGGTGCTGATCTTGGTGGTGGTGGTGATGGGGTTGTAGTGGGTGGTTG

TGTGGGTGCAGCACCTGGGGCTTGTGCTAAGCATGAGCTAGCCAGAAGACCCAAGATCAG

AAGAAGTTGTAAAGCACCAGAACCCATCTAGCTATAGAGAAAATGAGAGACCAAAGAAAA

TGTTGTAGTGCTTGTGTGTGTAAGAGAAGAGAGAGAGAGAGAGAGTTTGAGCAGTGGAAAGA

TGATAAGGGAACAGAATATATAAAAAGAGAGGTAGAGTATGTAAGTGGGGGGCTTCAAAG

GCGTTAAAGGACAGCGCTGATTAATGCATCAATAATGCTACAAGATTCCTCCAACTCCAA

TGTCTTAATTTAATTTGCTCCAATATTTTTTTTCAGAGAGATTACTACTGTACTCCAATC

AACTAGATCAA

>**Gmax_MSTRG.85662.1** GeneID=Gmax_MSTRG.85662;Strand=-;Pos=Chr16:34081435-34081736,

AACAATAACACGTCACCTTTTTATTGTGGAGTAATAATAAGGCAGATTAATTCATGTTTG

GTACTCCATCCATTCACACCATGTAGCAGCACTGTTTTGAAAAACGATCAATGCCGGAGC

CAAAATAAAAACTTGTTTGGTTTTAGTTTATTTTCTTAGTTGTGAAAATAAAAACAAATT

TGAGAGTGGTAAATAAATATATTAAAAAATGAAGCATTGCTCCTTCTCATGTAACATTTC

CCATGTCATTCGAGTGGAGGTTGTTGATGCTCCATATTCTCATGCGATGAGCTGTTTGCT

CC

>**Gmax_MSTRG.55218.1** GeneID=Gmax_MSTRG.55218;Strand=-;Pos=Chr10:49045608-49045999,

CTGAAACCAGTAACGGTGGCAAGAGAGATGGCTAAGGCGGCGCCAGAGAGATAGAGATTG

TTATTGAGGTGACCAACCATCATGATTGACACAACCTGCAATAAATACTGGGTAGCAGTC

ACAATTACCATAGGCACTGCTATGCAAATGATCCTCTTCATTTCTTCACCCAAACCATCC

CATGTAACTTTCTGCTTCTCTCTTTGTTTTGCTAATAGATTCTCTTCCATGGTTTTCTAT

CCTACTAGTACGACGAGTCCAATCGAGAGATAGAGTGCGTGAAAGATTTGAGTTGCTAAT

ATGAAAGATTGTCTCTGCCTCTCAATCAATCATATACTATGACCACATTCTTTCTCTTTT

TAGTGAATTTATGAACCACCATGTGTTGAGGA

>**Gmax_MSTRG.20764.1** GeneID=Gmax_MSTRG.20764;Strand=-;Pos=Chr05:131279-131821,

AAACATGAGTCCGGAAGGGATAGCTAATTTCAAGAACTCTGGAATGCTAAGTAAAGCGTT

ACTAGAAAAGACAATCTTGGTCTTTTGACAAGCCGGAGAGAAGATCATATAAATTGCAAG

CCAAACCACATTCAACCAATACGAGACTCCAATGGCTAATGCAGCTCCAATGTGTCTCAG

TCCCAACTTAAAAACCAGACCCCAACAAATAGGAACATGCAAACACAGTGCGGTGATTGA

GCTGAAAACCATGGGAAAGATCATACTCTGAGTCTGGAAGTAGCGAGTGAGAGCTTGAAG

AACAGCATGGCCAAATAGAGCTGGAATAAGGTATATGCAGTACTCACGAGCTGCATGAGA

AATTTCAGGGTCTTGACTAAACAGCATGAGTATTTTATCAGTGAATATCCACACCAGAGA

TATCGGGAGACAAACCAATGTCAGAGTCACAATCGCACACCAAATGTAGTTTCCAAACTT

CCTATACTCCTCCGCACCATAGGTTTGCCCACATAAAGTTTCCAATGCACCGGACATTCC

CAA

>**Gmax_MSTRG.64516.1** GeneID=Gmax_MSTRG.64516;Strand=-;Pos=Chr12:35567010-35568748,

AAAGAATGAAAATAGAATGTTTCTATCATATTTTTTTCATTCTCTATTTTCTCCGAACAA

ACAATGGCACAAACTAAAGCAGCTTATTGTACTTGAATGGTGATTATTTCACACCTCATT

TTGGGGTATATGCTGCTTAAATTAAATAAGAAATGCTGAAAAGCTAGATATAATGGGGAA

AAAAAGTGTTAATTTTGATATATTTTGAATCTGGAGAAGGTTGTATATTTTTATATATGA

ATGGATTTGAAATTTCGATGAGAGATGCAAAGTAAGAAATAAAAGAAGTTACCAGATAGA

AAGGGAATCAAGAGCCAACTCAGGATTAGGGAGAAGCCCCGCGAGCAAAACCAGAATCTG

AAAGTACCATGTCTCCAGGCACAGCATGACCGCGGACGCAGCGGAGAGCTTGAAGAACCC

ATACAAACCGCTAAACGCCTCCCACGTGAACCCCTGCCACGTGCGCCTGCACCTCTCGCT

CTTCACTATGTACACGTACTGTCCAATCACCATTATCCACCACGACAGGCTCAGCACCAG

CGACGCCCCCAACAGCCCCAACCCAATCTCGTACACCGCCACCCAACTCATCCCCAGGTG

GACCACCAGCGTCGCCGCTGATATGTACGCGCTCGGTGCCACTATGCTCTGCGCTTGGAG

AAACTTCTGGATCGGGAAGTTCGCCGCGTATGCGAATATCTGTGGGATCAACCCGTACAC

AAAGAGTGCTGCCGCCGACGCGATTCTCGGTGATTCCCCGAGGAATATTAGCATCGGTTC

GCTGAATACGTATATCACGGTCAACACTACGCCGGCTAGTGAGAGAAGAATCGTTGACCG

CTGCATGTAGACTCCTAACATCCCGTACTTTTGAGCTCCGAATGCTTGTCCGCATAGCGT

CTCCACGGCACTCCCCATACCCAACTGCAAGTAATATATATAATTTTGTGTTTTTCAATA

ATTACGCGCAATTATCAGCAGAGATTCACTTGAAAAGAAAATGGTGGTACCATGAGGCCG

TAGGCGAACATTTGGATGCCGGTGTTGCCGAGGGAGGCGGCGGCGAGTTCGAGGTTGCCG

AGGTGGCCGGAGAAGATTTGGGTGGACATGGACATGAGGTAGTTGATGAGGTAGACGATG

ACGGCAGGGGCGGCGAGGAAGAAGAGGAGCTTCAACTCGATCCAGGTGGCGGGTCCCACA

CGGTTGAGGAAGGGTAGGGTGTCGTCGGAGAGAATTCTCTCAAGTTGCCCATGGGAGGAG

GACTCGTGCTTTGAGTTTGATCGAGTTCCCTCCGCTGTTAACAGTGGCTCTTTCTCCGCT

GAACCCATTGATCTCGATCCGAATTCACGGCAAAGAAGGCTTTCTCTCCTGTAACGTTTG

GAGCAGCAGCAATCTCGCCGCCACAAGTCCCAACTCACCTACCACGAGGAGCCGTAAGGC

ACACCATCTCTTATATATACACATATAACGAGAATATTATATTATATTATATTCAACAAC

CACTAGGAGGCGGAACTCACTGTAATTATAACTGATTTGAAACGTGTTTGGGTGGGATAA

TCGTAGAAATGCTAGCTTTTTCGTTATGAAAATTACTAGAATATATGTGTTGATTTTTTA

TCCAAGTCAATCTCATTTTTACAAGAAAAAAAAAAAACAGAATGTTAATTTTTTTTTTCAG

CCAAAAGAAAAGCTTTTAATAAATGAAGGTACAGGGTACCAATCCGTATACAAAGTCAT

>**Gmax_MSTRG.10862.1** GeneID=Gmax_MSTRG.10862;Strand=+;Pos=Chr03:466665-467217,

GAGAAGAAAGCTCACCATAACATGGCACCAGATGAAATGGAAAGCTTGGCAACCGGCCAA

AGATCTTTGAATGCTAAAAAAGAGAAACCTTTCCATGTTTCAGGGCACCAACCACATGTA

ATAAATATCAGTTGACCAATGTTCGGAATCCAGTATGCCAAAATTGTTGAAATCATTGCA

CCAGGAATCCCATACTTGAATTGCATTGTGAATAGCCAGGAGAGGGACACATGAATGATT

ATTGATAAAGCTGCCAAATATGAAATAATGACATTCTTGCTTTGAGATTGAAGGAATGTC

TGGCAGCTGTTTGAGACAATATAAGCAAATAAGACAGGAATTGACCAAATAGAAATGGTT

CTTGCCACTTGTGCTATGCTCTCATCTTGGCCTAAGAGAGTCAAAATTGGGCTTGTGAAG

ATGAACAACGGAAGAAGACAGATTGCACTTAAGAATAAAACTATCCATGATCTTTGAAGA

TACACTCCCATCATGTCATATTCTTTTGCGCCGTATGCTTGTCCACAAAGTGTTGACAAT

GCACTTGCCATTC

>**Gmax_MSTRG.540.1** GeneID=Gmax_MSTRG.540;Strand=-;Pos=Chr01:2611779-2612886,2613846-2613908,2616315-2617601,

AGTGATTATGATTTTGAAGAGTATTCAAAAAGATTATAAGATAGAGATTACCATAGCATG

ACTCCAGCAGCAGCAGAGAGTTTCAGAAACTCCCAAAGACCAGAAAACGCTTCAACAGAG

AAACCACTCCAAGTGTGAGGGCACCCACCCCAAACAACGTAGCCAAAAAGCCCCAAAGTG

AGGACCCACCAAGAGAAGTTAATAGTAGCAGCAGCACCAACAACACCAAACTGAAGCTTG

AACACGAACAACCAACTTACAAACACATGCACCACGAGCGCCACCAGAGACACCCACGCT

ATGGGCGCGGTCTTCAGCTGGCACTGCAAGAACCTCTGCAGAGGGAACTGAAACGCAAAG

GCAAAGTGCACCGGTATCATCCAGATCGACACCGCGCCGGATAGCTCCGCCAACTCCTCC

GGCTGCCCCAACAACTTCAACACCGGCGAGGCAAACAAGTACAAAGGCAAAAGAAAGATG

CAACATATGAAAAGAACGATCCATGAACGCTGCATGTAAACGCCTAACATGTAGTACTTC

TTCGCTCCAAAAGCTTGCCCGCATAGCGTTTCTAAAGCGCTTGCCATACCCAACTGATTA

AAAGATAAATGCGAGTTAGTATTAACATTTAACGATTAGAAATTTCAATTCTAATCGGAG

AACAACATTAAATTAAAATACATGTTATTGTACTGGGCAATCTTCAACCTAGCTAAATAG

TTTTCGATATTGTTCTAATTAGAGAACATGGCTAAAAACACATTATGTAACTGTGTCTAA

ATTTTGTGCTAAGTGGGATTAATAGTATTATATATTATACCAAGAGGCCGAAGTCGAAGC

CAACGACGACATTATTGGCAATGGAGATGGCAGCGAGTTCGAGGTCACCAAGGTGGCCTG

CAAAGGCTTGTGTTATGACTAACATGGAGTAGGATGCAATACGGCTGAAAATTGAAGGGC

CTACTATGTGCCAAAGCCTCTTTGACTCAAGCCAAAACCTTCGTGGGAAACTTTGGTCTT

GTTCTCTTTCATCTGTTAAAGGGTGAGCTTCTAATAGTGGAAGCTTTGCTTCTACTAAAG

GTGAATCCTCTTTGCTTGGCATCTCGGAGTCGCTAGCTAGCAATATGTGGTGCTGGTGAG

GCTAGAGCTTCTGGGTGTTCTGAAAAGTGTGAAGGAAACAAATTTAAAGAAAGGAAGAAG

AGTGATCACGAGAGAGGTTCAAAAGTACAGGCTTTTAAAGCAGATGTGGCATGGTCTGGT

TTCTTTCAAATGAACAAACAAAAAAAGGGTTGGACATATACATGGAGAAGTAAATATCAT

GAGTTGATTCAAATTTTGATACCAACTGGAGTTAAAAGGAATGCTTCGGATTCTTGGAGA

TGGAGAAACAAAAATTCTGGTATGCATTCCACGAGGTCGGCATACGAGGTGTTGACATAT

ATATAATGATGAAGTTGACCTTGTCAATCAATTCTACCCTCTGTGGTGGCGTTTGAAGAT

CTCCAATAAAGTAGCTTTCATGATTTTGAGGGATACTGTTATGACTAGAAGCATTGATCA

ACAAAACCAAGAACCAGGAACTGCAGAACCAAGCAAGGATGCAAAACCAAATAATGGCCA

AAAAGAGAAATCAACAAACCCTACTACCTTAAAGGATTTTGTTTAGCAAATTAGGAAGTA

ATGGCTGAGCTTGTAGCTGAATAGTGGCACTCTAGATGCTTTGTTGGTTGATTCCATTCC

GTTAAGAATTGTCCCCTTTGATTTTGTCATTGTTTTCCTTCAAGGCACACAATGCCAAAT

CCGTTAGAGATTTGTTAGCTGCATTTCTATATATATAGGAATGATGTATTCTAATGGATA

AGCAGAAATAAAATCTCTTTTCCTCACCTGATTCTGGAGGTTCCTAGGCCTCGAATGCTA

GGATTTATTTTCAACTCATAACAACTTTGGTGCTTTCATTAAGAGGCTCACCATGGCTGA

ATCAACTCGGTCCAAATCCAATTGGGATCGCCTTGAAGAAGTCATAGTCAAATTGGCCTC

TAACCAGATCCACGTTATTGAGAAACTTGATGACCTTATGCATCCCATCACTGTCCTCGA

AAACACTTCACCCCTAGTAGCCTCACCTTTCTCGTCCTCAGCCATACCTAAACCACCTAC

CCACTTAACCAATCCACCAAAAATGAAACTAGATGTCCCACGATTCGACGGCTTAGGCCC

TTCTGGTTGGGTTTTCAAAATTACGCAATTCTTTGAATACTATTCCACCCCTAAATCAGA

GCGTCTCACCATCGCCTCCTTTTATATAGAGGGTCCGGCCTTAGCCTGGTTTTAGTGGAT

GATGCGTAGCCATCAGCTCACCACCTGGACGAGCTTCCTGCAGGTAATCGAGGCCCGTTT

CGCGCATTCCCCGTACGAGGACCCAACCGAAATTCTCTTCAAATTAACACAAAAAGGC

>**Gmax_MSTRG.93969.1** GeneID=Gmax_MSTRG.93969;Strand=+;Pos=Chr18:23076561-23077123,23077737-23078463,

ACCAGAGCATGACAGCAGAGGCAAGAGAGAGACGAACAAAGCCCCAAAGATTGTGAAAGG

CTTTGAAGGTAAAGCCAGACCAGGCCTCCCCGCAGGCCCCACCCATTATATAAACGAGCT

GCGCAAGGTCGATGAACCACCACGACGCGTTCAGCACCACGGCAGCGCCGACGAGCCCCC

ACCGGAGCTTCAGCATGAGCAGCCAGCTGAACACGGTGTGCAGCACCAGCGCCGCCGCCG

CAATCCACGCCATCACCATGATCCTGCTCTGCGCCTGCAGGAACTTCTGCGCCGGGTAGT

TCACCGCGTACGCGAACAGCTGCGGGATCATCCACACCGCGAAATCCCCCGCCGCCGCCG

AAATCGCCTCCGTCTGCCCTATCGCCCGCAGCAGCGGCGCCGCGAAGATGTAGAGGAGCG

TCAGCAGGATCGCGGTGGCGTTCAGGATCACCCACGAGCGCTGCATGTACACGCCCAGCA

TGTGCACTTGCCCCGCTCCGTACGCTTGTCCACATAGCGTTTCCAACGCGCTTCCCATTC

CAAACTGCGCGCAAAGTCCACAATGATGCCAAGGCAGAAGCCGGCGATGACGGAGTTTTC

AATGGAGATGGCGGCGAGGGCGAGGGTGCTGACGTGGCCGGAGAAGACTTGGGTGACGGC

GCCGAGGGAGTACTGGCAGACGGAGGTGAAGATGGCGGGGCCGGCGAGGTACCAGAGCTT

CTTCGACTCGGCGAAGAACTCTCTGGCGAAGTCGCCGGCGCCGGCGATCGGAGCGATGTC

ATCGGATTTCGCCGTGAAGACGACGGCGGGGTCCGGTGTGTGGTGTCGCGGCGTGAGAAG

TGGGTGAGTATGGTCCTCATGGTCCTCCATCGTGAATTCGTGATATGAATCAGAGATACG

AAAAAATTGAAAATTCTAGAACAACTTTATAAAGAAAAAGTTCCGGCCTTCAGAGCAGGT

TTTTACTGTTACCCAATGTAATGCCACACTCCGTCATCGGTTCGGAAAAGGAGATAAAAG

TAAAAAGGATAGGGAGAAAAATCAATCATAATGTCACGGAAAGTAGATAAAAAAACATAG

TGACAAAATAAAATTATTTTAAAGTTTAGGGTTATAACTTTTTTTCCAGAGTTTAGGGTT

ATAGTTGAAACGCCATAGTTGCTATAAAATATTTTATTCGCAATTGTCCGGTTCTCATGT

AAGAAAAAGTATTGTTCATGTCATTTAAAAGATAGTTTTAGCCTTTGGAAAATTCTCATT

TGTCTTAATAAAATTTAAACGTTGAGTTTT

>**Gmax_MSTRG.104177.1** GeneID=Gmax_MSTRG.104177;Strand=+;Pos=Chr20:36602362-36602429,36607743-36608312,36608599-36609133,

GTTGTACATACCATATGGATAAGACTGAAGTTTCAAGCTCTGGATTTGGTAGAAGACCAG

AAAGCAAGGTTAGCAGCTCAAATGACCACCACTCAAGGCTGCAACGATCAAATAAGTAAG

AAACATACCATGGTTGAAAATTAAATGGGAAATAAAAACACAACACAAAAGATAAAATAT

AAGAGGAAGAAACTCACCAAACCATTCCAGCTGAAGGAATAGCATAGGTGAAGAATTCAC

CAATCCCATGGAATAGTTCCATTGAAATTGGGACTCGAGTCCTTTCACATTCAGTAGAGA

ATTTCATATATAAGCCAAGTAAAATCACATTCAGCCAGTATGAAGTACCAATTGAAAATG

CTGCTCCCAAGTTACCAAATCCAGATTTAAAGACCATTAACCAACTGAAAGCCACATGGA

AGCAAAGAGTAATGGAGGAACTTATGACAAGGGGACTGATCAAACTTTGCATCAAAAAGA

ATCGAATCAAGGCCTGAAGTGTTGCATAAGCAAAGAGAGCAGGGATCATGCACAAAGCAA

ATTTTCCAGCTTCTTGTGAAATCGAAGGGTCTTGGCCAAGAAATATGAGTATCTTCTCCA

AGTAGACCCAGAAAAGAGTCAGAGGAAGACAAGCTAAATAGGTCTCAAAGCTTAAGAAGA

AAGCAGTGAGATGGGAAAGGAAGAGACTTACAATAAGACTGAAGCCAGAGACAGCACAGA

GAGAGATGGCAATGGCTGTGCTAGAGAGAGCAAGCTTACCCAAGTGACCAACCATCATCA

TTGAAATAATCTGTAGAAAATACTGTGACAAAGTCACAGTAATCATAGGAGCTGCTAGAT

AACCCACTCTTTTCATTTCCTGAGAAAAGACAGTCCATGTTATGGCTGAAGCTGAAGGAT

TCTCCGGCTCTGAGTCCTTATCTAAGAGACTATTTTCCATATCCTCTCCAAAGGGTCACA

GAATCACTGCACCTTGTCGAAACTGGTAGAAATAATGGCAGGGCTTTGGTGTTTTGTATG

CTAAAAGGTTTAAATATTTACAAGTGGCAGCAGCAGTAGTTCTTCCAAGGTTTAAATATT

TGTTTCAATAATGACTGTCTCAGAGTCTCTCATGGGCTTCCCCCTCTTATGTAGACGGAA

CAATATTTCAAGTAAATTTTAAAATTTAAAATC

>**Gmax_MSTRG.62823.1** GeneID=Gmax_MSTRG.62823;Strand=-;Pos=Chr12:8615952-8616426,

TGGTACTGTTGATCCGAAAGATTTTTACACATGATGAATACGAAATTTATTGTTAAATAG

ATCATTTTTATAACATGTTTGTTCTCCCAACATGTGAACACAACGTGACTAACACTACGC

AAACTATTCGGGTTAGACCAACAAATTGGTAGTGAAATTATGAATTTGGCCTCATCTTAC

AATTTATTTACAACAAAAAGCTATGAGATCTAAAGCCCGATTTGTGTTATTGTATAATTG

TGGGCTAGGCTACATTGAGAAACAAAGAGCCTCAGTTATTGACAAGTGGCTCCGTTTTGT

CCTCCCACTTGTTCAACCTCTTTGCTGCTTCTTCAACCTACATTAATTCAAGAAATTATG

GTTAGAAATTATTAGCTCGTTAACTTCATACACATTACATAGTAGAATGATCAGAACAAC

AGAAGAAGGTTGTTTATAGTCTTCTTCTTTTTTACCTCTTTATTCCAATCCGTTC

>**Gmax_MSTRG.58090.2** GeneID=Gmax_MSTRG.58090;Strand=-;Pos=Chr11:8552500-8553008,8553236-8553369,

CCTGGATGACCATCCGATCAGGTGTAAAAATATTGGGAAATAACCAAGGAACCGATGTTC

CAATAATCCCTAACAACAATCCAAGTATAGCTCCAATTATCACGAGAGACCTTAGAAGCA

TTCGGGCCTGTTATTTTGACAAACTCCGATTTACTCCATATATCAATTCAGGCATAAATG

ATTGAGAGGTTTGGGAGAGAGGTTCACCCCATACGGTACACATTAAATAGGTTTGGACCA

TGACCTGAAGAGGGAATGAAGTTATCACTGGCCTTGAGTTTTTGTTTTTAAAAAAAGCTC

AGATGGCAGGCAAAGGAAGGAATAGTCACAAAACAGGAAACAAAAAAAACACTAACTTGA

TGAGCAGCCATTGTATGTGTACCCATTGATGTAGCAAAATATATAAGTAGTGCGTAAAAA

GCCACCTAAACATCAGAACTTCAGATTATATTAAAAGGTTATACCACTCTAGACAAAGCC

ACAGATCTAGTATAATACTTGAGACAACCTTTGACATCAATGTTATAAAAACAGGAGCAG

CAAGCCCAAATATCGTCAGAAGTTCCTTCCCTGAAGGAATGGAGAAGGCAAGTGCATTAT
ATCCCTTCATATTTAGATTTTGAATCATCATGTAAGAAGCAAC

>**Gmax_MSTRG.85656.1** GeneID=Gmax_MSTRG.85656;Strand=-;Pos=Chr16:34066562-34067182,
AAATAGAATAGGAACCAACAGCAATCACAGGATTGTCTAGTAGACCAGCAAGTAGCATAA
TGCATGTAATATACCATTGCTCTAAGCAATTCATTACAGATGATGCAAGGCTTAACTTAG
CAAAGGCCCACAAATCCCTAAATGCCATCCAACAAAATCCACTCCATTCTTCTTTGCACC
AACCAATGGTATACACAACCAAAGCCACAGCATATAACCACCCTACGATATTAGTCACTA
TTGCCAAGCCGGTTATACCCCAACCAAATACATTTATGAATATGTAAAGCAACCCATTTT
GTATAAGCAAATCCACAAATGCTATGCACATAATTACTTTAACCTTGCTCTGGGCCTGAA
GAAATCTCTGGATGGGAAAAGCTACAGCAAAAGAAAACATGTAGGGAATTACTTGTATAG
AATATCTTCCAGCAACTTCAGCTATTCCTTCATCTTGGCCAAGCAACTTTAAGATTGGAG
TGGCATATACATAAATAGGCAAGAGGATTATACAAGTGGCAGTAAGTATAATCCATGATC
TTTGAACATAAATACAAGTAGATTGAATTTTTCCTGCTCCAAAAGCTTGGCCACAAAGTG
TTGCCAGTGCAGATGACATAC

>**Gmax_MSTRG.55219.1** GeneID=Gmax_MSTRG.55219;Strand=-;Pos=Chr10:49046691-49047235,
CAAATCATCACTGCAGAAGGGATAGCAAAGCGAAAGAACTCCCAAATTCCTTGGAACAGC
TCCATAGAAATGGGTGCACGGGTTTTTGCACAAGCAGGAGAGTATCTCATGTATAATCCA
AGAAAAGTCACATTTAACCATATTGAAATGCTCATTGCTAATGCTCCACCAATATTATTC
ATTCCAGTCTGGAACACCAAAGCCCAACAAAGGGGTATATGGATACAAAGAGTGACACAA
GAACTTATAAGCATGGGAAGAAGCAGACTTTGCATTTGAAAATATCGAACAAACGGCTGC
ATAATTGCATGTGCAAAAAGTGCGGGAACAAGCCAGATTAAGAATTTTCCTGCTTCTTGT
GCAATTAGAGGGTCCTGGCCTATGAAAACTAGGATCTTTTCCATGCTGATCCAAATGAAA
GTTAGGGGAAGACAAACGACTGTGAGAGAGAATATAGCAGTGTATGTTTGCACTCCAACT
TTTTCATATTGCTGAGCTCCATAAGCCTGTCCACAAATAGTTTCCAGTCCACTAGCCATT
CCTGC

>**Gmax_MSTRG.47705.1** GeneID=Gmax_MSTRG.47705;Strand=+;Pos=Chr09:40373989-40374776,
CCTTCCCACCAATATGCAATACAAAAGCAAACACGATAGATGATGGAATCCCAACTATAT
AGTATGACCCCAGATTGACAAATGCACCAATTTTCTGCCAACCACATCCTCTAGCAGTGC

CTGAATTATTGTTGACAAAGTGAAAAAAGAAAGTTATACATCATCTATTTCATGCTTATG
TGATGTGCAAGAAGGAAATGTGGGTTCTAAAGAAAATTGGCCACAAAATGAACTAATTCC
GTTTAAGTCTTAACTGTCAGTATGCCTCGCATATTTATGCAATATGCATTGGAGAAATTT
AAAACTAAAGAAAATCAAATCTTACAATTGCTTCACTGAAAGCTTCACATACACTCATTT
GTACTATGCACACATGTTCCTTTCCGGGTATTGGCCCATAGTAACCAGATGTCAATTGAC
TGTATACTATTTGCATTTTTGTATGTGAGATGTATTGGAAGTTCCAATATGCCTCTCAGC
CTATACCAGTGCTTTTGAGTCATAGCTCCTGTGCAGACAAGTAAAAGTAATGGGGGAAAA
GAAAAACCTGAAAGAACACATTGGAGTCCATCCAGAAAGTTGGATGCTGCCAGAATTGGG
AACATAGTTGCTACATATTCGACCACTTCTACTTCATTGCTATATGCATAGCCCCAGATA
TTGCGTACAAGTATCATCACTGTTCCCACTATGATGCCCTCAATAATTGCTAGTACTAAG
ACAACACGCACTGCTAAACGTGCATTCCACGGACGACCAGCTCCAAGTTCATTTGAGACA
CGAATGCT

>**Gmax_MSTRG.85660.1** GeneID=Gmax_MSTRG.85660;Strand=-;Pos=Chr16:34078563-34079165,
CAATCACAGGATTGTCTAGTAGACCAGCAAGTAGTATAATGCATGTTCCATACCATTGCT
CTAAGCAACTCATAACAGATGATGCAAGGCTTAACTTAGCAAATGACCACAAATCCCTGA
ATGCCATCCATGAAAATCCAGTCCATTCTTCCTTACACCAACCAATGGTATACACAACCA
AAGCCATAGCATAAACCCACCCTGTGATATTAGTCACCATGGCTAAGCCAGTTGTACCCC
AACCAAATACATTTATGAAGATGTAAAGCAGCACATTTTGTATGACCAAAACTGCAAGTG
CTATGCACGTAATAACTTTAACCTTGATCTGGGCCTGAAGAAATGTCTGGAAGGGAAAAG
TTATAGCACAGGAAAACATGTAGGGAATTACTTGTATGGAATATCTTCCAGCAAGATCAG
CTATTTCGTGGTCTTGGCCAATAAACTTTAAGATTGGAGTGGCACATACATAAATAGGCA
AGAGGATTATACAAGTGGCAGTAAGTATAATCCATGACCTTTGAACATAAATACAAGTTG
ATTGAATTTGTCCTGCTCCAAAAGCTTGGCCACAAAGTGTTACCAATGCAGATGACATAC
CAA

>**Gmax_MSTRG.46904.1** GeneID=Gmax_MSTRG.46904;Strand=-;Pos=Chr09:33354097-33354434,
CAACAACTGAAAATAGATGGAACTCATGACCCCTTGGCTCACAGAGATAGAAGAGAGTTC
AATGTCTCCAAGATGACCAGCATATATGGAAGTTGAGGAGTTTGTGAGAACCTGGAATAG
ATGGGTCAATGCCACTGGCAATGCTATTCTCCATATCTTCACTGTCTCTGCCCACAAAAC
AAATTTAACATCCTTCAAGCTTTTCACTGGAAAATAGTCAGCTTCTGAAGTAAAATTTTG

GACCACTAATGGTGTCTCCATTTGAGGAGTTCTTCCCTTGATACTCAGTTTCTGAGGCTG
AATTTTGTTATAAACTTCCAATCATGCATCTAAGCACG

>**Gmax_MSTRG.17617.1** GeneID=Gmax_MSTRG.17617;Strand=-;Pos=Chr04:8840746-8841061,
AAAGAACTGGTTGAATGCAATTAAGAAGAACAGTAAAAGCTAACAACATTGCCAGCTCAT
TGACCATTTGGATAACGGAAGAGCTCGATGTAAAGATCAAGGCAAGATTCTTGTTGAAGG
ACACAATTACCAACCAAAATATAAATCCCACAAATAAAGTAGTTACCACAGAAACCACAG
TTGCAAATCTTGCACCTTTTGCATTGCCTGCACCAAGCTCATTTGCTACACGCACCCTGA
GTTGAAGGAAGTTAGATGATTATAAGGGAGGAAAAATTAGTTGGTTTTGTACTGAGACAC
AGTTCTTTACATCAAA

>**Gmax_MSTRG.77932.1** GeneID=Gmax_MSTRG.77932;Strand=-;Pos=Chr15:2770096-2770480,
ATCTTTTTTCAGCACTAGTGATTGTTCCATTCCATGCAATCTTTCATTTTCACGTTATTC
ATGCAATTAATTATATTTGCATGGTTACAAACAGAAGCTTGATTCTATATTTCATTTCAT
TTCACTATATATGAGACTATGAGAGTAGAGTTAGAGATTTTTCTGTACATACTTATGATG
CAGAGGTAAATTTTTCAACCAATACCCCAAGCACAATCAAGCAGGGAAAAGTGCCTATTC
TTTCCGAAGAATTTTTTAAGCGACTTAAAAACCTAGGGAAAGCTTCTCTTCACTTAAATG
TAATCAGCAAACACCACTGAAACCTAGGCCTAATATGAAGCTATAACTACGGTGTCTGCC
ATGTGCAGTCATGAGCTCCTTAGGA

>**Gmax_MSTRG.46373.1** GeneID=Gmax_MSTRG.46373;Strand=-;Pos=Chr09:18852614-18852946,
TGATAGTATGCCATTTTTTTTAATAAAATTCATTATAGACCTTATGCCATGATGGAAACT
AGTGCCAATAAGATGTATTGTCTTCATGCTTTTAACATGAGCAATGATCTAGAGGTCTAG
CTCACTTGGTTGAGCAAGATGTGTCAATCGTTATAATCCCTCTTGATACTATCGTTGATT
CCTATGGATAAAAAAACATGAGAAATGCTTACCTCCACACACCGGCAAACATGCGAAGAC
TCATGTAGATGGTTAGTGCAATCCATATCCCAACGAAACCTTTGCTCTTAGAGAGAAGAA
ACAATGAAGCAACACTTGCCAATGACACGGTGA

>**Gmax_MSTRG.547.1** GeneID=Gmax_MSTRG.547;Strand=+;Pos=Chr01:2749414-2749652,
GAGAGAACAGGCTGAACATTATTAATGACAATGCAGAAGCACAAAAATGGTGTGAGGTCC
TTAACTAGATCTTGCACTTCTGTGTCGTTTGAAAACAGGGAAGGGTACTCATTCCGCGAG

ATCATCAAAACAATAGCGAGTAAAACTCCGATCAAAACCGAAGTAATCACGGCAACCACT
AGGGAAAATAATGCTGTTCTTGGGTGGCGTGCCCCTAGTTCATTCGATATTCTCACACT

>**Gmax_MSTRG.85377.1** GeneID=Gmax_MSTRG.85377;Strand=+;Pos=Chr16:31794885-31796085,
GGTGCAACTTTATATAACAAGTAAACTTCAACTTAGAAGATAGTCACAATCTAAATTGAT
CATGAGTCAGCAATGTGAACTGTGGAGAATCAAATTTTTGGCGTTTAGCACAGGTCAAGT
TCTGACGCAAGTTATACACAGCTTGAATAATTATTATTATAAGTGCTGCATTATGTGCAA
CAGCTTTCAGGCAAAGCTGCTTTCAGTTTAATCTCTATGTCTCTCAGCCTTACATCTAAA
ATATTATCAAATGTCAGATGCCAGATTCTCACATCCCATCCACATCTCCTTGCACTTGTC
ACTCACAACACAACACAGCAAAAGCTACTTTTTCATCTCAACACCCAAAACAAAACTTGC
CCATTAGGAACACCAGAGAGAGGGAGACAAAAGTGCTAAATATATATATATGTAGCAATT
GGTATAGAAAATGAGATTTGGATTGGAATGGTTTCCCCAAGCAAATAAAATTAAATTAAA
AATAAAAAAGACAAGCCCTCCTCAAAATTATCATAAAGCAGTTAACTCACAAGCCTCCAA
CCCCATTGATCCTACCCAACAAAGAAAATCCCAATGTCATAATTGGTGTCATTTCATTTC
TCTAGTGTGTGGTATCTTTTTTCTTAGCATGTGTCATCTTTGTTGTTCCCATTTCCATTC
ACCAAGAGCCCTTTGCTTTCTTCATCTCTCTCATTTTCCTTATTCCTAAGCCCATTGCAA
CTACCCATTTCAATCCTAGTGAGCTTCTCAGCCTTCAAAGCCTCAGCTTCCCAATCTGTC
CTCACCAACACAACATAGAGGATTGACACAGCACATGCCACCTGGGCCGACAGAAGCCCA
AACCAAAGCCCACTGAACCCCACCTTGAACCAAAAGGCCAGGCCCACGGCCACTGGGGTG
CCCACAAAGTAGAATGAGCCTAGGTTTATGTGGGCCCCAATACCAGGTCGGGCCGTGCCA
CGAAGGATCCCACAGCCCGTGGTTTGTGGACAGTTCCCAAGCTCACACAGGCCCATAATC
GGCATGACTGATGCAACCAAGGCTTTTACGGGCTCGTCGTTGGTGAAAAGCCCAGCCCAC
CTTTGACCCAATATCACAGTCCATGTAACATTGATGAAGCCTATCACAAAAGCACATCCT
AAGGCAACCGTTGCTGCTAGTTTTGCCTTGTATGGTTTCCCAGCTCCAAGCTCATTCCCT
A

>**Gmax_MSTRG.27310.1** GeneID=Gmax_MSTRG.27310;Strand=-;Pos=Chr06:7113529-7114096,
GGGGGAACCCAGGAGTCCTTATAAACCCCGGAGAAGTAAATAAAGGAAGAAAGGAAGAGG
AAGAGGTTTAGATTGGTCCACACCATCGCTATTGCGACGCCAGACACACCCATTTTTAAG
TGGACCACTAATAAAAAGTTGAGAGGGACGTGGAGGAGGACGGAGACGGCGGAGCAGTAG
GTTAGGGGTAATGTGATGCTTTGTGTTCTGAGGTAGATTCTCAGAGGGTGGAGGAGGGAG

AGGAGGAAGAGGTCTGGGATAGAGAAGAGGATGAAGGTCTGTGCTGTGCTGGAGATCTCA

TGGTCCTGCCCGCACCATAAGAGGATGTTCTTCATGTTCAGCCACGTCAGGGAAATGGGG

AGGGAGCTTGAGAGGAGAAGGAGGACGGTTCTCTGGAGGGTTAGGCCCAGTGTCTTCAAT

TGTTTGGCACCGTAGGCTTGCCCGCAAATGGGTTCCATTCCCATGGCCAGGCCCGAGATC

ACGGAGTAGCCGGTGATGTTGGCGAACCCGATCGAGAGGGAGCCTCCGGCGAGCTCCATC

TCGCCTAGGTAGCCCAGGAAGACCATGG

>**Gmax_MSTRG.89036.1** GeneID=Gmax_MSTRG.89036;Strand=+;Pos=Chr17:11018480-11020414,

CATCAATAGACCAAAGGGTCGTTGTACCTATTCAATCTTGTAATCTTCAAGTCTGAAGTA

TCACTGCTTTCGTTACTACAATTACAACTAGCAATGATATTCAGGACACATCCGAGATGG

CAAACACTCAAAATGGTATTGAGAAGGGACTCAGCAAAATGAACATTGGAGTATATATAT

ATGATCGTGTTTTGACTCTAGAGTAGTGTGTCTACGACTTCTATACGTTTCTGATTCTTC

CTCACCAAGTGTTTTATACATGAATTTGGGGTGATAGAGGTTTCTTTTCACACTTGATCA

TTCTCATGGTTTTCAACAAGTTCATTTACCTCATATCTTGGAACTTGTTCCTGTACCTGT

GCGATACATACAAACGTTTGTGCCTTGGTAGCTTCTTGCACCCAATTTATTCTCACAATA

AACGTCAACAACAAAATCAAGCAAGCAACGATACCAATCAAGAGTCCTATAGTGAATCCA

GCAAGTCCTAACCGAAGCTTGAAGGCAAAAACCACACCCAACGGCAGTGTCAGGAAATAG

AACCCACCAAGACTAGCATACATGCCTAGCCGGGGTCGTCCAGTTCCTCGTACTATGCCT

CCACAAACCGTCACAGGAAAATTAAACACTTCCACCAGAGCCATCAACAACATTGCCTTC

TTCACACCCTTTACAACCCCTTTATCATGACTAAACAAATCCCCCCAAACCCCTCTTGCA

GCCACCATCGTGGAACCACCGATACAACCAGATACAACACTCAGTGCCAAAGACACACGT

GCTGATTCACAAGCTTGACCGGCACGGTTAGCACCAAGCTCATTGGACACACGAGTGGAA

ACACTAGTGGCTAGAGAAATCATCACTGCGTAATGCAAATAGTCAAAGTTCTACACTATG

GCTAGAACCCCCACTGCTTGTTTTGCATTTGCTAGGTGGCCAGTAAGGAAAAGTAGAATC

TCATAACACCACCACTCAAGGCACGTGTTGAGGCAGCATGATCCACTAAGCTTGATTAGC

CTAATCCAATCCATCATGTTCTGATCCCACCACCCTCCTTCCTTCCACAACAGGCCCTCA

CTTCTTCTCTCAAGAACCACAACATAAATCGCCAGCATAACCATAACCATGAGATCGGTT

ATCCAAACCGCAATTGCAACTCCTCGGAGTCCCATGGTTTTGGAGAGTACTATGTTGACG

GGTATGTGAAAGGCTAGTGCCACTGCAGAACTAAACATGGTGGGAAGAGTCACGCAATGA

GAGCTGAGGTAGGCTTTTAAGGGACAAAGGAGTGCCTTGATAAACAAGTCAGGTATGAGA

TTGGAAACATAGGTTTTGGCAACGGTTGAGATCTCTTGTTGTTGGCCAAAAAGAATCAAA

ATCTTGCCAAGGTTAAGCCACAAGAAAGATAAGGGAAGTGTTACCAAAAGCAACAAGAGG

GTTGTCATGAGAAGGGTCTTGTGGAGAAGCCTCGCGTTCTTGGCTCCATGTGCTTGTCCA

CAGATTGGTTCCATGGCGCCGCTTAGACCGTTCAAGACAGAGAAGCCGGTGACGTTAGCA

AAACTGAACCCGAGCGCGCCACCGGCTAAGTTCAGCTCCCCAAGGTGGCCAAGAAAAGCT

GTTGTTATGGCTAACTTGGCAAACCAAGCCAAGTTCATGGGCACAAGTGGAAGGGCTATC

CCTCGTTGGACTCTTAGCTCTTCTACCACCATCTTCACAAAATTGTTGGCATTGGAAGGG

CATTTTTGGGTGTGTGATGAAGGAAATGAGGGTTGCCGGTTGGACCAGTCGTTTGCGGTT

ATGCTCTCTCCTTTAGATGTTGCTGACATTTTTGCTGAGATGAATCAGATGATGTTTTGG

TGAGATTGTTGCCAAGTTCCACTAGCGTGTTTCTTTTCTTTAATAGTATAAGATTAGATA

AGGTTTAGACTTTGG

>**Gmax_MSTRG.55217.1** GeneID=Gmax_MSTRG.55217;Strand=-;Pos=Chr10:49042628-49043924,

TGGAAGAGTTTTCTTAAATTTAAGCAAATGCAACATACAGAGATCTTTTTTTTTTTGTGG

AATATAGGGGCCTAAGCCCCAAACAATACAACTAAAAACCACAAGGGAACACGGTTGAGG

ATATATCTGCTACAACAACATTTCATAAAAAAAAACGGAACAAAGTCAAAAATCGAACAG

CGGCCATCTAAAGATAAATCAAAGCCAGCAAGCTTGTCTGCGACCTGGTTGCCCTCTCAC

AGCGCGTGAGACCAGACAAAAACTCCTCCTTTGACCTCAAATTCATTAATGTTCTTGATC

AACATGAAACAAGGATGAGTATCGGGACACTCCTTAATAAGCTCAATGACCAGCAAGCTG

ACTCAACTATGAACTCCTTGAATCCACGACTCCAAGCAAGCTGCAAGCCAAGAAAAATTG

CCCAAAGTTCGGCTGCCAAAACAGAACAGCAACCAATGTCCGCAACGAAGGGGAATAAAA

CCAATCCAGTGTGATCACGAAGGACTCCTCCAGCTGCTGGCTTTCTCGAAGCTGTATGAA

CAGCTGGATCACAGTTCAATTTGAACTGATTTGTTGGAGGAGGGAACCACTGAATGCTAG

AGCTAGTCACAGGATTAACAATCTGAGGAACCAAACTCTATTGGCACTGCAAACTCACTG

AGATAGCATCAACCTGGAGCAAAATCTGATTTGCAATATCAATCAGCTGTAACAAGGAAT

TATTATCAAAGATTCCTTTGTTTCTATATCTCCAAATGTAGTTCAAAGCCACAGCAAAAA

TCATACACCAATTTCTGCCATTAACCATCAAGTTACTAGTGACATTCTCAAGAAGTCTAC

TCTTAAACTCCTAAAGAAAGAAGTTTGAGTGCAGAGAATGAGGAAGAATCAAGTACCATA

TGGCTCGAATAATGGGGCAGTGTCTAAAGCCATGGTCATTATTTTCTGAATCAGCACCAC

ACCTTCAGCAAGCAGCATCTAAAGTTAGGTGGCAGGAGCACTTATTCTCATTTGTCAAAA

GCGCATTAACTCCAATTTTCCAGAGTAAAACACACACCCTCTCTGGACCTCTTCAATTCC

AGATGGCAGAATACAATGGATCATTGCACAGAGCATTATTATCCAGTAAATTTCCCATCA

CTAGACCCCTTCCAAATCACGATATCAGAGCCTTTCAAGCTATTCGGGGGAGGAGTGGTT

CTCAAATATTTCCCATCGAATTTTGTTTTCTAATACATAATATGCATGGTTTATTGTAAA

GGTTTAAAAAAATTACAAATCATAAAGAAAATATAAC

>**Gmax_MSTRG.58091.1** GeneID=Gmax_MSTRG.58091;Strand=-;Pos=Chr11:8554799-8555270,

TGCAAATCAAATAAACAAATATAAATTGCGCCCAATATAATTAAGAATCTGAATTAATTT

TTCATGCTAATTATTATAGCAATCATACACATATAAGTATCAAAGTGAACTCCAACCTAT

GAGCCAACCCAGCTCGCGGACTAAACAAATTATAAAAACGATGGCTCACTTTTTAGCCGG

TTGTCCTGGTCTTAACTCGCTTAACCCATAAGCTGAGATGGTCCAACATACTACTATCCT

CTTTTTTTTTTTATTAAAACATACTTCTATCCTTGTAACTGGTGTGATGACTTCATCAAA

CTAGGCAGTCCTTAGCTTTAGCAGCTTATACTGTTCTGTATCTTCCGAGTATAAGATGCC

TTTGGGAGAAAGAAGGCGCTGGAGGGCCATTGAAAACCGAGCCTAACAATCACATAAGAA

TTACAAATCAGAATGCTTTAAGAACTAATACTCTTGACTCAGAAATGCTGGC

>**Gmax_MSTRG.65157.3** GeneID=Gmax_MSTRG.65157;Strand=+;Pos=Chr12:38511442-38511522,38511675-38511775,38511902-38512225,38549594-38549605,

AGGAGGTGTGGGGATGTAAGGTGGTTGAGATGTTGGAGATGGGGTTTTTGGAGGTGTAGG

AGTGAAAGGAGGCTGAGAAGAAGGGGAAGGGGTTTTTGGAGGGTTTGGAGTGTAGGGAGG

TTGAGAAATAGGAGAGGGAGTGTTTGGAGGGGTTGGGACATAAGGTTGGTTGCCAGGGGA

AGGTGTTTTTGGAGGAGTGGGAACATTTGGAGGACTATAAGAAGGAGAAGGTGTTTTTGG

AGGTGTAGGAACATAGGGTGGATGGTTACCCGGGGAAGGAGCTATTGGGGGGGCGTAAAT

AGGAGAAGGTGTTTTGGGAGGTGTTGGAACATTAGGAGGAGTATAAACAGGGGAAGGAGT

TTGTGGAGGAGTTGGAATAAATGGAGGATGATTAGTAGGTGAAGGTGCTTTAGGAGGAAC

ATAGGGAGGACTGTGAATAGGAGAAGGTGTTTTTGGAGGGGTTGGAACATAAGGTGGATA

AGGAATTTTTGGAGGAGTTTGAGGAGGAGTTGGAATAA

>**Gmax_MSTRG.104177.8** GeneID=Gmax_MSTRG.104177;Strand=+;Pos=Chr20:36605721-36605828,36606807-36608312,36608596-36609133,

CGCCATTTCCTTATTTTACTGCATTAGCTTAGTTACCTTAGTATAGCAATCCTAACAGTT

CTTGCCTCAAATTTTTCTTTTCAGGAATATTCGATGGATTTTGCACATAGCCATAAAGAC

TTGGAAGTGGAGGCAAACCTGTTTTTCCCAGTTTGTACAACTTGTGATGAGAGATAGCAT

AATTGTTTGGCAGAAGGCACCAGTCAGTATTCCAATCCAAAGGCCTTTTCCTCTTAATTG

TAGCCAGAAACCCAATATGGCAGCCATTGGAATTCCCACAACATAATAAGCTCAAAGGTT

TACATATGCTCCTATGTGCTGCCACCCACATCCTCTAGCAATACCTATAATGAAGGCATA

ACTCAGATTCCATTGCTTAAGAAGAGTAATGTGCATTGAAAAATAGTTCAAACAAGAACT

AACCTGAAAGGGTACCGTGTAACGTGTCTACTATAACAGATATACTTAAAAGAGGAACCA

TATCTGTGACATAATCCACCACATCCTGCTCATTGCTAAATACATAACCTAAAACCTGCC

TGCTAGCAAAAATGATTGAGCTCACCAGAATGGCCTCAGAAACTGCTAGAGTCATGGCAG

CAGAAACAGATACTCGTGCTGATTGTGGACTTCCAGCTCCTAATGCATTTGAAACTCTAG

TGCTGAACAACCCGCCCAAAAGAGAAACCAATCAGAAAGAGGAAAAAATTCATTTACTAT

AATATCATTCTACATGAATACGAATATGCACAAATTGATGAGCTTAGGACTTGTGTGAAT

CAAAACCTTGCTGCTGAGCCAATTGCTTCTGGGATTGTGTAGATAGTTGTGATGATGGAT

AAACTGGTCAGAACATAGTTAACAATAACAACAAATTAGCAACACTTGCATGAATGGAAA

TCAAAATTTGGCTGAGTAACAATTTCAACACAGATTTTAAAACACAAAGCCATTCATGTC

TGAAGGACTAGGATAAGTTGTACATACCATATGGATAAGACTGAAGTTTCAAGCTCTGGA

TTTGGTAGAAGACCAGAAAGCAAGGTTAGCAGCTCAAATGACCACCACTCAAGGCTGCAA

CGATCAAATAAGTAAGAAACATACCATGGTTGAAAATTAAATGGGAAATAAAAACACAAC

ACAAAAGATAAAATATAAGAGGAAGAAACTCACCAAACCATTCCAGCTGAAGGAATAGCA

TAGGTGAAGAATTCACCAATCCCATGGAATAGTTCCATTGAAATTGGGACTCGAGTCCTT

TCACATTCAGTAGAGAATTTCATATATAAGCCAAGTAAAATCACATTCAGCCAGTATGAA

GTACCAATTGAAAATGCTGCTCCCAAGTTACCAAATCCAGATTTAAAGACCATTAACCAA

CTGAAAGCCACATGGAAGCAAAGAGTAATGGAGGAACTTATGACAAGGGGACTGATCAAA

CTTTGCATCAAAAAGAATCGAATCAAGGCCTGAAGTGTTGCATAAGCAAAGAGAGCAGGG

ATCATGCACAAAGCAAATTTTCCAGCTTCTTGTGAAATCGAAGGGTCTTGGCCAAGAAAT

ATGAGTATCTTCTCCAAGTAGACCCAGAAAAGAGTCAGAGGAAGACAAGCTAAATAGTAG

GTCTCAAAGCTTAAGAAGAAAGCAGTGAGATGGGAAAGGAAGAGACTTACAATAAGACTG

AAGCCAGAGACAGCACAGAGAGAGATGGCAATGGCTGTGCTAGAGAGAGCAAGCTTACCC

AAGTGACCAACCATCATCATTGAAATAATCTGTAGAAAATACTGTGACAAAGTCACAGTA

ATCATAGGAGCTGCTAGATAACCCACTCTTTTCATTTCCTGAGAAAAGACAGTCCATGTT

ATGGCTGAAGCTGAAGGATTCTCCGGCTCTGAGTCCTTATCTAAGAGACTATTTTCCATA

TCCTCTCCAAAGGGTCACAGAATCACTGCACCTTGTCGAAACTGGTAGAAATAATGGCAG

GGCTTTGGTGTTTTGTATGCTAAAAGGTTTAAATATTTACAAGTGGCAGCAGCAGTAGTT

CTTCCAAGGTTTAAATATTTGTTTCAATAATGACTGTCTCAGAGTCTCTCATGGGCTTCC

CCCTCTTATGTAGACGGAACAATATTTCAAGTAAATTTTAAAATTTAAAATC

>**Gmax_MSTRG.70935.3** GeneID=Gmax_MSTRG.70935;Strand=-;Pos=Chr13:37706552-37706619,37707015-37707220,

AAGAGTAGCACCAGCAAGCTGAAGATCACCAAGGTGGCCAGCGAAAATTACAGAAACCAA

CACGATTAAGTAATAGAACAAGTTTGTAAGGAACATAGGTAGTGAAAAAAGTAATTGATG

TTTGGCCTCCTCCAAATCCAGGATTTTGCTGTTCCACCATCGGTACTGTCTTGTGTTTTC

TTTATGATGAGATACATCTAATAGAGGTGTGAAGTGTGTTACTGTGCCATCTAAACTCCT

GCTTTTCAGCATCTTCTATAATCTCTTTGCTCCT

>**Gmax_MSTRG.33766.1** GeneID=Gmax_MSTRG.33766;Strand=-;Pos=Chr07:9484568-9484827,

AAATTGTGAATCATTCATTCTCCCACTGCTAATTTAGTCTTTGGTTTGATTTTAACAGCC

CAATTATGAATCTACGAGGGCACTTTCCTTTTATATACAAAATAGTTGTACCAAAAAAAA

TGTTCACTAAAAACTGCTTTTCTAAATAATCAGAATCACTAAGCATAATTACTGTTCAAC

TGTCGAAGATTTTAATTTTGGTCACTTGGCAGTGCATTTGCATCAGTTGGCACTCCGTTG

CTTCTTATTCTTTTTGCTGC

>**Gmax_MSTRG.55224.1** GeneID=Gmax_MSTRG.55224;Strand=-;Pos=Chr10:49057222-49057518,

TCAAGGATCGGCTATTAAATGAAAAATAGTTGGCATAGCTCAAAATTACATTTTGCATTC

GAAACATTGCTAAACGTTTAATCCTATTATCATGACAAAAAATGCTACTATTATTCTTTA

ATCAGATGCACGAAGGTACAAGCTCAAATAGCTAACCACTTCCAAAAAACCAAAAACTAA

ACTCCTACTCCTCACTCACGGAAAATAGTCCCCATCTTTAGAAAATTGGCCTTTGAGGGC

TTGTCATACTCATACCAATATATTGTCTGCTGAAATTTTTTCATCAAATAACCTCTT

>**Gmax_MSTRG.33765.1** GeneID=Gmax_MSTRG.33765;Strand=-;Pos=Chr07:9481346-9481877,

CAGACCATGAGTGCTGAAGGAAAAGCAAGACTGAGAAATTGAGGAATGTTCTGCAAGGAT

TCCCTTGAGAAACCAGTCCAAGTGCTTTTGCAGGAAGAGGAGAACCTGATGTAAAGTGCT

AGTAGCACCGTGTTAAGCCAATTTGAAATGCAAAATGCAATGGCAGCTCCTTTGATGCCA

AGCCCAAATTTTAGAACCAAAAGCCAACAGAGAAGGGCATGTGCTAAGGTAGTGAATCCA

GAAGCTAGCATCATTGGAAGGACAATGTTCTGGGTTTGTAGGAACTTAGTAATGCACCGA

AGAAGAGCGTTGGCTGAAAGGCTTGGGATCAAGTATGTGGCATATAGTTGAGCTTGTGCT

GCAATTGTTTTGTCTTGATGCAGAACAACCAGAATAGGCCTCAAGTATGCCCAAATAAAA

GACATTGGTATAGTAGCAAGCATGATGATCACTATGGCTCTCTGCATGTGTATGCCAACC

ATATGATACTGCTGTGCTCCATATGATTGACCACAAAATGTGTCAAGTGCAC

>**Gmax_MSTRG.85659.1** GeneID=Gmax_MSTRG.85659;Strand=-;Pos=Chr16:34078020-34078271,

GCACTGATGACACCTTGGTAAACAGAGATAGAAGAAAGTTCAATGTCTCCAAGATGGCCA

GCATAGATGGAAGTTGAGGAGATTGTGAGAAACTGAAGCAGTGCAGACAGTGCCATAGGG

AATGCTATTCTCCATATCTTCACTGTCTCTGTCCACAAAACAAACTTCAAATCCTTCAAG

CTTTTCACCGGCAAGTAGTCAGATTCTGAAGTGAACTTCTGGATCACTAAAGGTGTCTCC

ATTTGAGGAGTT

>**Gmax_MSTRG.10861.1** GeneID=Gmax_MSTRG.10861;Strand=+;Pos=Chr03:460202-460230,465916-466105,

GTTTAGTAACAAAGAAACTGCTAACAAAGGTGACAAATCCCCCACAGCAGTAGCCACATC

TTCGTTTGAAGTAAAGAGATAAGCTACTTTTTCTCTTAAAATTAAAAAAAGAACAAATAA

GATAAACCCAATTGCAAATGATGTAAGCACTGTCACAACTATAGAGAACTTTGCAGCTTT

GGAGTTTTCCCTTCCAAGTTCATTTGCCACTCGAACACT

>**Gmax_MSTRG.10860.1** GeneID=Gmax_MSTRG.10860;Strand=+;Pos=Chr03:459418-459671,

AAAATATATCAATCCAGAGACTAACTTAAGGAACATTCAATACTTTAAGAACTACTTGTA

TAATTTATAATTTAATTAATTTAAGGACTATTTGGGAGACTACATTGATAGTTTATTCAA

ATACCTATCAAGCAATCTCTTGGCTATATAACACCGGCTAAACAAATAAGGACGATATAG

ATTAGAGTCTGAAACTAATTATCTGATGTAACTGTTTCACGGTCAAGGTCCACCTTAGAC

CACTTACTAATACG

>**Gmax_MSTRG.91145.1** GeneID=Gmax_MSTRG.91145;Strand=+;Pos=Chr17:40286874-40287099,

GTCCGAAAATGCTAGCCATGTAAATCCACTCCATGCTCCATCTGACTTAGTGATGAAAAT

GTACAACAATTGTGCAATCACAATCACCCACCATGATGTATTGAGAGTAACTGCTGCTCC

AATTAGGCCCCATCCAAGCTTAAATATAACAAGCCAACTAAAAAATGTGTGCAGCACCAA

AACAACTACTGATATCCATAACATCACCAGCACTTTCCTCTGTGCC

>**Gmax_MSTRG.58088.1** GeneID=Gmax_MSTRG.58088;Strand=-;Pos=Chr11:8549475-8550036,

ACCTAAAGCAGCAAGCTCAATTGAACTTCTCTGACCAATAACTGCAGTATCGATGAGACT

CATAAGTGGTCCACATATCCAAAGTCCAGTTGCTGGTCCTGTGAACATCACAATTTCCTT

TATCTGATCCCATATGCCTTGCTTCGCGAGTTCCTTTTTCTCACCCTGCCTCGATATTTC

TTCGTTGTCTTTCTCTTCTTCTTGTTCAAGAGCCTCAGTGATCTGGTCTTCGTCTTGGCT

TCGAGCGGTTACGAAGAAGCGATGGCGATGAAAGGTGGACGCTGAGGCTGCGCCGGATAG

GCACAGGGTGGGAGGAATGGAAGGTGCGAAGAAGCGACGAGAAAAGTGATGGTTTGAGAG

TGTTGAATTAGGGTTTTGGCGGGAAGGGGAGTGAAGAGAGCAAAGGAGAGGTAGTGATTT

CAGTTTGAAAGCCATTTTCGGTGTCTAACTCTGGTTGGGTGGAGGGAAGGAAAAAGGGAA

GCTGAATAATCCCTCCCACCAAACACAGGCTGAGTGGCTCACGATGGTAAACTCATTATC

TAGTATATAGTAGTGTTTTCTT

>**Gmax_MSTRG.85378.1** GeneID=Gmax_MSTRG.85378;Strand=+;Pos=Chr16:31798250-31799600,31799844-31800091,

AAACACAGCCTGCAAGTGCCATTGGGACAGTGTACATCATGCTAGTTGTCTGAATGAGAA

TACCAGTGGCAGCCACAGCCAGTGTTGGACGTGGCAAGTACCCAGCCAGCACAGTCACAA

TCTCGTACCACCACCACTCCAAACATATCATAAGGCAACTAGGCACAGCAAACCCCATCA

ACTGCCCCAACCCAGAGCACACCACTCCCCCACCACCACACCGCCACTTCAGCACCACCT

CCCTCTTCCTGCACACGCACACATACCCTGCCATCAGCACCACCATGTTTAGATTAGTCA

TCACTGAAGCCATGGCCACCCCTGGTACCCCAAGCCCCATCACCACCACCAAAAGATAGT

TCAATGGCACATGGAACAAAACAGCCACAAGGGAGCAATACATCATAGGTTTGGTCACTT

TTTGTGACCTCAAAAACACCCTTAAGGGTTGTAGTAAGGTGTTTGTTAAAAGGTCTGGGA

GAGAGTAGAAGCAGTAGAGTGATGCCATTCCTGTGATGGCACTGTCTTGGCCCATGAAAA

GCATGATCCTTTCGAGGTTCAGCCACAGGAGGCTTATGGGGACTATTGCCATGAGGAGGA

TGAGGACCATTCGTTGGAGAGAGAGAGAGAGAAGGTCCCAGTTTTTGCTACCAAAGGCTT

GGCTGCACACAGGTTCTAGGCCTGCTGCAAGACCCACAAGAACAGAGTACCCTGTTATGT

TGGTGAAGCCTATGGAAAGTGCACCACCTGCTAGCTCTAGGCTTCCAAGCCTACCCAAAA

AGAGAACAGAAACCACTGCTCTCACAAACACCAACATGTTCATAGCTGTGATGGGTAGAG

CCATGCCCCACAGCTCCTTCATCTCTTCCATCACCTACTTGGCAACACAACATCACATGA

CTAGAACACAAATAAGAAAGGATAAAAAAAAAACTCATTTTTAAAACAATAGTCTTTTTC

TTTTTACTGGCATCAAGGTTCTAAATTACGGTTAAGGTCACAATAATTTCGTCATGTTCT

TTGATATTGTGGGAAATTACATACACACAAATGTGGTAAGGTCTATGTGATCACCACAGG
TTGTTTTGCAGTAGCAGACACTCGAAAAACCTTATTGTTGCGAACGAAATCGCGCGAACC
GGTTTTTAAAAAGCCTTGACTTGATTTTAGCATCATGGTCGAGTATCTCTAAAAGAAACT
ACTCTTTTTCTCTTACAAAATGTGTTTCCATGGAGAAGTAAGTAGCAACATGAGTTTACC
TGAGAGGTTGTGGGGAATTTGTGGGAAAAGAAATCATGGTCCTTGTTATCTCCCATTCTT
CTTGACCAATATATGTATGTGTTGTTGTTCTTTTTTTTTCTCCTCCTTATGCTCCTTCTC
TCTAAAAACTGAAAAAAGGGGTGAAGCAAAGGGTGTGTGACAAGAAAGAAATGCTATGAG
GGAAAAGGGGGGCACTTAAGTAGAGCAACCAACATAAACAGTGTGGTATAGTATGGCTCA
AATACTTGAAAAAAGTTTCAGTCTTTCACTAATTCTAAGTATTTGTCAAAAGGTGTAAAA
ATAATTAAACATGAAACGTAAGGTCAATAATAATAATAA

>**Gmax_MSTRG.17618.1** GeneID=Gmax_MSTRG.17618;Strand=-;Pos=Chr04:8842303-8842915,
TGTCATTTAACCAATTGGATTAGTGGATTGTGTAAATTGAATTATGCGGATTGGAGTACA
TAACTCAATTCATCTCATTAGGATAAGCTAAATACTTTATCTGTGAGACCCAACCTATTA
CTCCTTTTATCTCTTCCCAAAATGTGTGTTGAAACACATTCGACCAAAGAGAGTTAACTT
CCTCTCCATTATGGCATGGTCTAGTAGGAGAGACATAAACACATTTCACAAAACCTATGT
GCGTATCTCTTCTACTGGTCTCTATAGCAATAAATGAAGACAATCCAGTTCCAAGCAAAA
AACACGACATAAAAACATGAGAAAAGGCATCCTAATCTTTTCCCCTCATCATCATCACTC
TATACATAGATTGTCCCTCTGTTCAGCGTCAACTTCACTATTATCAGTGATTGAAGACGA
CTCATCCAATTTCACATACTTCACAAGCTTATGAATCCATCATATCTTTTTCCACATTAT
CTATAAGTTCCTCTTTCAAACCCATTCCGAAACACACCAGTATAGTAGACGCGGAGTTTT
ATTTGAGCAACTGCGTCTTTTACAAGCTCCCGTTGCTTCCTGTGTTAGAAAACTAACTTT
AAGAGTATTCACC

>**Gmax_MSTRG.64518.1** GeneID=Gmax_MSTRG.64518;Strand=-;Pos=Chr12:35569920-35570159,
CCAGATAAGACGGGTTGAATGCCATTGAGGACAAGAGAAAGGGCAAGGAGTGGACAAAGA
TCTGAAACTGCAGCAGCAACCTCTTCACCCCCTGTGAAGGCATAGCTAATGACATCTCTT
AATGCAAGAACCACCAGTGCTGCAATTACTGATATTATGAAAGAAATCACTGTCACCACA
ACCACCGAAAACGATGCTGATTTTGGACTTCTTGCTCCTAGTTCGTTGCTCACCCTCACG

>**Gmax_MSTRG.85378.2** GeneID=Gmax_MSTRG.85378;Strand=+;Pos=Chr16:31798250-31799600,31799843-31800091,

AAACACAGCCTGCAAGTGCCATTGGGACAGTGTACATCATGCTAGTTGTCTGAATGAGAA

TACCAGTGGCAGCCACAGCCAGTGTTGGACGTGGCAAGTACCCAGCCAGCACAGTCACAA

TCTCGTACCACCACCACTCCAAACATATCATAAGGCAACTAGGCACAGCAAACCCCATCA

ACTGCCCCAACCCAGAGCACACCACTCCCCCACCACCACACCGCCACTTCAGCACCACCT

CCCTCTTCCTGCACACGCACACATACCCTGCCATCAGCACCACCATGTTTAGATTAGTCA

TCACTGAAGCCATGGCCACCCCTGGTACCCCAAGCCCCATCACCACCACCAAAAGATAGT

TCAATGGCACATGGAACAAAACAGCCACAAGGGAGCAATACATCATAGGTTTGGTCACTT

TTTGTGACCTCAAAAACACCCTTAAGGGTTGTAGTAAGGTGTTTGTTAAAAGGTCTGGGA

GAGAGTAGAAGCAGTAGAGTGATGCCATTCCTGTGATGGCACTGTCTTGGCCCATGAAAA

GCATGATCCTTTCGAGGTTCAGCCACAGGAGGCTTATGGGGACTATTGCCATGAGGAGGA

TGAGGACCATTCGTTGGAGAGAGAGAGAGAGAAGGTCCCAGTTTTTGCTACCAAAGGCTT

GGCTGCACACAGGTTCTAGGCCTGCTGCAAGACCCACAAGAACAGAGTACCCTGTTATGT

TGGTGAAGCCTATGGAAAGTGCACCACCTGCTAGCTCTAGGCTTCCAAGCCTACCCAAAA

AGAGAACAGAAACCACTGCTCTCACAAACACCAACATGTTCATAGCTGTGATGGGTAGAG

CCATGCCCCACAGCTCCTTCATCTCTTCCATCACCTACTTGGCAACACAACATCACATGA

CTAGAACACAAATAAGAAAGGATAAAAAAAAAACTCATTTTTAAAACAATAGTCTTTTTC

TTTTTACTGGCATCAAGGTTCTAAATTACGGTTAAGGTCACAATAATTTCGTCATGTTCT

TTGATATTGTGGGAAATTACATACACACAAATGTGGTAAGGTCTATGTGATCACCACAGG

TTGTTTTGCAGTAGCAGACACTCGAAAAACCTTATTGTTGCGAACGAAATCGCGCGAACC

GGTTTTTAAAAAGCCTTGACTTGATTTTAGCATCATGGTCGAGTATCTCTAAAAGAAACT

ACTCTTTTTCTCTTACAAAATGTGTTTCCATGGAGAAGTAAGTAGCAACATGAGTTTACC

TGAGAGGTTGTGGGGAATTTGTGGGAAAAGAAATCATGGTCCTTGTTATCTCCCATTCTT

CTTGACCAATATATGTATGTGTTGTTGTTCTCTTTTTTTTCTCCTCCTTATGCTCCTTCT

CTCTAAAAACTGAAAAAAGGGGTGAAGCAAAGGGTGTGTGACAAGAAAGAAATGCTATGA

GGGAAAAGGGGGGCACTTAAGTAGAGCAACCAACATAAACAGTGTGGTATAGTATGGCTC

AAATACTTGAAAAAAGTTTCAGTCTTTCACTAATTCTAAGTATTTGTCAAAAGGTGTAAA

AATAATTAAACATGAAACGTAAGGTCAATAATAATAATAA

>**Gmax_MSTRG.20765.1** GeneID=Gmax_MSTRG.20765;Strand=-;Pos=Chr05:132197-133379,

TGAAGGCTGAAGCATATTCATTAAACTTGTGGCATCTGATTTATCAGACGCCAGAGGCAC

AAATCCAGTACAAAAATCCCATCACATATTCACACCAACAAGTTGTGTATGTTAACATAA

TTAAATCTCTTCACCAAAAAAACAGATACAACTGGGCCACACTCACTATCCTCTATATGA

CTCTATCTTTCCACTCCACCGCAGATGACAGTCCATGCAGAAACCAAAACATCATTTATT

TTTCAGTTTTCTTAAGCTTCATAGTGAACTTTAATTGAGTTCTCAACTACTCTCTCCCTC

GCTTTAGTTGCCTGCAGCAACCACAAAATACAGTCATTTAATTGAATTTGTGGCAAATAA

TAACTAGCTAGTGTCTTAAGAATATTATGTTTTCAATGATTTTTTTAGGCTCTCCTATGA

TCCACATAATAAATATTTTTCATTTTTAGTTCTTTAACTAATACTCTAAGAACACGGGTT

AGCATTTGTGATATTTGTACAGGTTACGTAATGTAAGGTATGGAATGAACGAACCTCTTT

GTGCCAATCTGTTAGTGCTGTTACAATAGCAAGAATAATGACTTGTGTAAGAGAGCCTGA

TAGAGTTCCCATCCAAAGGCCCTTGGCTCTTAGTTGCAGATGAAAACCCAATAACAAACC

CATTGGAATTCCCACAAGATAATAGGCTCCCAGGTTCACATAGGCCCCTATTTCCTGGAA

TCCTCCCCCTCTCGCAATCCCTGATAGTGCTCCAATTAGACTATCGGCAGTAACAGACAC

ACACAAGAGTGGAGCCATCTCTGCAACGTAATCGATTACCTCCTTATCATTGCTGTACGC

GTATCCTAATACATGTCGGCAACTAATGAAGACACTGCTCACAATAACTGCCTCTGCCAC

CCCGAGAATCACAACCACTCGAACTGCACCTTTTGCTGTCTTTGGATTCCCTGCCCCCAG

TTCATTTGAAACCCGAGTGCTGCAAACATCCATCATCAGATTAAGTGTGTTGGAAGCCCC

AAAAAAAGGCAAATTTGGACCAAACCTTTTTTACCTTGCAGAGGCTCCAACAGCATAAGG

AATAAAGTAGTGCAATGTGGTTGTGTTAAGGCTGCAAGAAGCGTATGAATGATCAAAAAG

TTAATTAGAAGATCACGAGTTATTTCAGCAAGGGTATTCTTAG

>**Gmax_MSTRG.70935.2** GeneID=Gmax_MSTRG.70935;Strand=-;Pos=Chr13:37706547-37706792,37707188-37707391,

AAATTTTCCTAGCATATTGCATCATTGTTAAAGTAGCTAGCACCAATCTTATTATAAAAC

TCAACAATATTGCTTATCATACATGATAATTCATTTCACTACATTCCTAAAGAAATTCTG

AATATAATTTACCATGACAGCCAAACCAGTGACAGAAAACCAAGAGTTTGCAAGAGTAGC

ACCAGCAAGCTGAAGATCACCAAGGTGGCCAGCGAAAATTACAGAAACCAACACGATTAA

GTAATAGAACAAGTTTGTAAGGAACATAGGTAGTGAAAAAAGTAATTGATGTTTGGCCTC

CTCCAAATCCAGGATTTTGCTGTTCCACCATCGGTACTGTCTTGTGTTTTCTTTATGATG

AGATACATCTAATAGAGGTGTGAAGTGTGTTACTGTGCCATCTAAACTCCTGCTTTTCAG

CATCTTCTATAATCTCTTTGCTCCTATTTT

>**Gmax_MSTRG.85657.1** GeneID=Gmax_MSTRG.85657;Strand=-;Pos=Chr16:34067788-34068140,

CTGACATCACCTGTGAAGCACTATTGAGAACAATTGTTACACCAAGAAGGTATGCTAAAT

CAGCAGCAGCTAGTATCATATCCTCACTGTCGGTAAAGATTTTGGCAAACTCATCTTTAC

TAAAGAAAATTACGGTCATGAAAAGAATACCAAGGAGGAGAGACTGGAACATTGTCACAC

AGAAAGAGTATATGGCTGCTCTTGGATGCGACATGCCAAGCGTATTGGAGACACGAACAC

TTGGAGGGTATTGAACCAATAGTCAGTGTAAGAATGAAACAAATACATGAAAAATAGATG

TCTATGTCTTAGTTCCGATAAATATGGAAATTTTGGTTTTAGTTTATGAAAAC

>**Gmax_MSTRG.10723.1** GeneID=Gmax_MSTRG.10723;Strand=-;Pos=Chr02:48386690-48387375,

TATCTTGATGTGCATTGGAGTAGATTAGTGTAGAGAAGAGGGGCAGGAGTGGAAATTGCA

AAGAGAACCGGCTGCAGTGTGAGGAAGAGAGAGATGGGGGCAAGAAGTAAATTAAGAATG

ATGGCAGTGATGCATGAGGGGATGAAGGAAATTAGAAGGAGCGTGACCTACATGCCTCGA

CCAGGGGGATGAGGTGGTGGAGCAGGTGATGGAGGCGATGAGGCGTGTGCCGGAGGAGGT

GCTGGATTCGGGTGGTTTGAGGACTCCGGTGGGTGGGGGTGTGAGGGACCTCGATCTCTA

CGCTTCGCGAGAACACCGAGGTCGTTCCTGGCGTCGTCGAGAGCCTCGAGGTTATGTCCA

AGTAATATGCCTTCGAGCAGTTATTAGAGTACTCCCCCCTTTTTGTAATAAATAAAACTT

CATTTTCTATTGCTATTTTTTCACCTGGGTCGAATCCTCACCCTGTAATAATTCGGCTTA

ATTTTCATGTAATTATGTTTGCTCAACCAAAGAATACTTCTCTATTCCTCCTATTCGCAT

AATCTGTAATGTAATGACACTGCCTTCCACCCCCTCCGTAACATTATTAAAATACTAAAT

TGAACCATTTTCCTTTACACTGTAACATTGACTGGAAAAATGACTAATTTCATTGCGAGA

ACCTAGAAAGTGCTTCTAAAATGTCC

>**Gmax_MSTRG.10863.1** GeneID=Gmax_MSTRG.10863;Strand=+;Pos=Chr03:469768-470173,

GGCGAAGCGTATGATGACAGTGAACACAAGGGCATAAGCAGCGAGTTCCCTTGAACCAAT

ATGACCAATAAATGCTTGGCTTATAACACTGATGCCAAAGGTTGTGAACCTTGTGAATAT

GGCTGGTGCTGCCACTATCCACATCACCTTGCTCTCTTCCCACACCCTCTTCACCAATGA

TAAATTCTCTTCTTCTGATTTTTGCTCTCTGCTCAACAGCTTCTTCTCAAGATTCCCCTC

CATTTTTTGAGTCTTTGGTTGTTGTTGGAGCCTCTGAAGATGGATCAGAGACTTCACAGT

ACACAAGGGAGTTTTGGGCTGATATTATACAAAAGAAAAGGAAAAAAAGAAAAAAGAATG

AGTATCTTATCGTTGCTCTTGCATGAGAGGGACAGCAACTAGACAC

>**Gmax_MSTRG.6396.1** GeneID=Gmax_MSTRG.6396;Strand=+;Pos=Chr02:7920926-7921612,

AAGTTGTGGATTAGGTAAAAGCCCAGCAAATAATGTAAGTAGCTCAAACGACCACATTTC

AAAACTATGAACAATGAAACAATTAAGAAAATCAATTAGGAGATGACTAAACTGAAAAAA

CAATTGTGCAATGTCATAATACATTACCAAAACATCAGTCCAGAAGGAATAGCAAATTGG

CAGAACTCTGGAATGCTTAATAAAGCATTAAAAGAAAACACAATTTTGGTTTTCTCACAT

GCTGGAGAAAAATTCATATAAATTCCAAGCCCAATGACATTTAACCAATATGAAATTCCA

ATAGCATATGCTGCTCCAACATGTCCTAGGGCTAATTTGAATACCAAAGCCCAACAAATA

GGAACATGCAAACACAGAACCGCAATTGAGCTGAAAACCATGGGAAAGATCATACTCTGA

GTCTGGAAGTAGCGAATCTGACATTGAAGAACAGCAAAGCCATACAATGCAGGGATGGAG

TATATGCAGTACTCATGAGCTACATGAGAAATTTCAGGGTCTTGACCAAACAACAATAGT

ATTTTATCCACGAATATCCACAGCATAGATATGGGGAGACAAACCAAAAGCAAAGTCACA

ATTGCACAAAAAGTGTAGTTTCCAATCTCTGAAAATTCTTCTGCACCATAGGTTTGGCCA

CATAAAGTTTCCAATGCACCAGCCATT

>**Gmax_MSTRG.85661.1** GeneID=Gmax_MSTRG.85661;Strand=-;Pos=Chr16:34079744-34079972,

CCTGACATCACTTGTGAAGCGCTATTGATAACCATCGATACACCAAGAAGGTATGCTAAA

TCAGCAACAGCTCGTATCATATCCTCACTGTCGGTAAAGATCTTGGCAAACTCATCTTTA

CTCAAGAAAATGGCAATCATGAAAACAATGCCAAGGAGGAGAGACTGAAACATTGTCACG

CAGAAAGAGTATATGGCTGCTCTTGGATGCGACATGCCAAGTGTATTGG

>**Gmax_MSTRG.6401.1** GeneID=Gmax_MSTRG.6401;Strand=+;Pos=Chr02:7932706-7933126,

CCAAAACACTAAACCCAGAAACATTGGTGAGAGAGGTTGCAATAGCAACACTTGAAAGAG

AGAGCTGGTTAAGATGGCCAACCATTACTAAGGACACCACTGGCAAAAGGTATTGTAACA

CACTTGAAACCGCCATTGGAGCTGCTATAGTACCCACCTTCTTCAGCTCTTCCCTCATTT

TCATCCATCCCCACTTTCCTGTCGAACTTTCTTCCATCACTGCACCCTTTTATCATTTTT

GTACGATCAATTTTTACTCTTTTATATCACTCACTTTCAAGTGTTATATATTATTACCTT

TTCTTCTATATGTCGATCTTATCCTTCTTTGGCTCTGCCTCTACCTTTGTCCTCTTTTGT

ATCATCACAACTAAACACAACTTTGCGTTATCTTTTTCAGTCACCCACGCAACTATACCT

T

>**Gmax_MSTRG.47707.1** GeneID=Gmax_MSTRG.47707;Strand=+;Pos=Chr09:40376755-40377191,

CACCCATCCATCAGTTTAAACTTATGAAGGAAAAGAGAACTAACATACCAATAAACTGAA

ACCTGTGACAGAGGCAAAAGAAGTGGCCATGGAAGCACCAGAGAGAGACAACTCGCCAAG

ATGGCCAACAAACATGACAGATATGATACTGAGGCAAAAATTCAAAAGGGTCACTGATAT

CAAAGGCCCTGCAAGCCATAACTGCTTCTTCACTTCTTCTATAACTTCCCTTCTCTCAAT

ACCCCTCCCTGATGATGATGAGTGTGAGTGCTTAATCAATGGAGACTGAAGAGATGCACT

TTGGTTTTGGTCTCCTCTCTCCATTTCTCTTCTTTGTTGCTCTTTAAGTTCTTAACACAT

GGAAGACTCAGACAGAGAAAAATGTTGCATATTGCATGGTGTTGTTAAAAAGATTATAAA

AACATCTGAAAAACTCT

>**Gmax_MSTRG.65157.2** GeneID=Gmax_MSTRG.65157;Strand=+;Pos=Chr12:38510925-38511374,38511462-38511524,

CTACAACATAAAATTAATTATGAGACACGATCTCATCTGTCCAAAATAACAATATTAGCA

AAAAAAATGAAAATGTTCACAATTACTATATACTATAACTCAAGTTCCAAAAAACAAAAG

TTTGATTTTTCTAACCAAGAATGGTGCTTAGAGCAGCACCAGCCAGAATGGTCAGTGAAT

ACAGCCCTAACCTCGACGGTGCTACAGACGACGCGCCGTTTGGTCTGGCTGGCGTCGTTT

CGTTCGACGCCCCCGACGACGACGACGACGACGGTGAGGGAGTTGTTGCTGCCGGAGAAA

TGGTGGGAGTGGTGGCTGACGTCGACGGGGATGGTGGCGATGTTGCCGGAGCCAGGTAGG

AAGGTGGGTAAGTGGGTGCAGATGAAGGTGAAGGTGAAGGTGGGTAAGATGGAGAGAGTG

AGGTTGAGGGAGGGATGGTGGATGGATAGGGTGGTTGAGATGTTGGAGATGGGGTTTTTG

GAGGTGTAGGAGTGAAAGGAGGCTGAGAAGAAG

>**Gmax_MSTRG.65157.4** GeneID=Gmax_MSTRG.65157;Strand=+;Pos=Chr12:38511442-38511522,38511675-38511823,38511902-38512515,

AGGAGGTGTGGGGATGTAAGGTGGTTGAGATGTTGGAGATGGGGTTTTTGGAGGTGTAGG

AGTGAAAGGAGGCTGAGAAGAAGGGGAAGGGGTTTTTGGAGGGTTTGGAGTGTAGGGAGG

TTGAGAAATAGGAGAGGGAGTGTTTGGAGGGGTTGGGACATAAGGTTGGTTGCCAGGGGA

AGGTGTTTTTGGAGGAGTGGGAACATTTGGAGGACTATAAGAAGGAGACGGTGTTTTTGG

AGGAGTGGGAACATTTGGAGGACTATAAGAAGGAGAAGGTGTTTTTGGAGGTGTAGGAAC

ATAGGGTGGATGGTTACCCGGGGAAGGAGCTATTGGGGGGGCGTAAATAGGAGAAGGTGT

TTTGGGAGGTGTTGGAACATTAGGAGGAGTATAAACAGGGGAAGGAGTTTGTGGAGGAGT

TGGAATAAATGGAGGATGATTAGTAGGTGAAGGTGCTTTAGGAGGAACATAGGGAGGACT

GTGAATAGGAGAAGGTGTTTTTGGAGGGGTTGGAACATAAGGTGGATAAGGAATTTTTGG

AGGAGTTTGAGGAGGTGTTGGAACATAAGGTGCTTTTGGAGGAGTAATTGGAACATAGGG

AGTGTTTGGAGGTGTTGGAGGATAATAAGGAGGTGGCTCTCTCACAGCCAACACCACAAT

AATCAATTTCTGACCCTTTTCACAATTACCATCTTTCCCACTAATGAAGTAAAATGGACC

CGATCGATCAAACTGAAACTCCGATTCACTATCCTCAAACTTCTTGATCGGGTTCTTCTT

GTTACATTTCTCATAATCATCCTTGTTCACCACCAACACAGTATCCGAGCCCTTCTTGTA

CTTA

>**Gmax_MSTRG.104177.7** GeneID=Gmax_MSTRG.104177;Strand=+;Pos=Chr20:36605721-36605828,36608599-36609160,

CGCCATTTCCTTATTTTACTGCATTAGCTTAGTTACCTTAGTATAGCAATCCTAACAGTT

CTTGCCTCAAATTTTTCTTTTCAGGAATATTCGATGGATTTTGCACATTAGGTCTCAAAG

CTTAAGAAGAAAGCAGTGAGATGGGAAAGGAAGAGACTTACAATAAGACTGAAGCCAGAG

ACAGCACAGAGAGAGAGATGGCAATGGCTGTGCTAGAGAGAGCAAGCTTACCCAAGTGACCA

ACCATCATCATTGAAATAATCTGTAGAAAATACTGTGACAAAGTCACAGTAATCATAGGA

GCTGCTAGATAACCCACTCTTTTCATTTCCTGAGAAAAGACAGTCCATGTTATGGCTGAA

GCTGAAGGATTCTCCGGCTCTGAGTCCTTATCTAAGAGACTATTTTCCATATCCTCTCCA

AAGGGTCACAGAATCACTGCACCTTGTCGAAACTGGTAGAAATAATGGCAGGGCTTTGGT

GTTTTGTATGCTAAAAGGTTTAAATATTTACAAGTGGCAGCAGCAGTAGTTCTTCCAAGG

TTTAAATATTTGTTTCAATAATGACTGTCTCAGAGTCTCTCATGGGCTTCCCCCTCTTAT

GTAGACGGAACAATATTTCAAGTAAATTTTAAAATTTAAAATCATTCCATCTAATCTAAT

TTAATCTAAA

>**Gmax_MSTRG.17616.1** GeneID=Gmax_MSTRG.17616;Strand=-;Pos=Chr04:8838100-8839080,

GCAAGCATGACCCCAGAAGCCAAAGAAAGTTTGAAGAATTCCCAAAGGCCAACAAAAGCT

TCAACAGAAAAACCAGTCCACGAGCGAGGGCAACCACCAAAGAGTGTATACCCGAGCATT

CCCAAAACAGAGAGCCACCACGAGAAACCAATGGAAAGAGCAGTGCCAACAATCCCAATT

CTCATCCTATAAACAAAAACCCAACTCACTAACACGTGAACCGCAAGAGCCACCCCTGAA

ACCCAAGCAATGATGCCGGTCTTCAACTGGCACTGCAGGAACCGCTGCAAAGTGAACTGG

AACGGGAAGCTCAGGTGCAATGGGATCAACCAAACCGCCACCAGCCCCGCCTGCTCCGCC

ACCGCGACGGGCTGCCCTATTAGCTTCAACACCGGCGTCGCAAAAATGAACACCGGCAAC

AACAGAATGGAAGACAGGAACAGAACAACCCACGAGCGTTGCAAATAAACGCCCAATATG

CGCTGTTGTCCGGCGCCGTACGCCTGCCCGCATAGTGTCTCAAGCGCACTCGCCATACCC

AACTAAAAAAGGAATAAGTTCGTTAACAAAAATTAACTAACTAACGACTAACAACAATAA

TGCGGTGAATGAAAAGTGGGAAATAGTGAATGAAAAGTGGTAAGTGGAAAGTGTTACCAA

GAAACCGAAGGTGATGGAGATGAGGACGGTGCAGGCGATGGAAATGGCGGCGAGGTCGAG

ATCGCCGAGGTGGCCGGCTAAGGACTGGGTGACGACGGTGATGGAGAACATGGCGAGGCG

GGTGAAGATGGAGGGGGCAGCGATGTGCCATAGCTTCTTGGATTCGGAACATGATCGTTG

GAAGAGAGAGTGATTGTTGGATCCGTGTGGTTGTGGTGGCAGCTTTGACTTTATCAGAGG

ATGCTCATGCTCTCCTTCATTCAGAATATTTCCCATGTGTTTTGCTTCGCTTCGCAGGTT

GTCGATTACTGGACAACAATA

>**Gmax_MSTRG.70937.1** GeneID=Gmax_MSTRG.70937;Strand=-;Pos=Chr13:37710136-37710668,

GGGTGGGGATGTTGGCATATCTTCCATGGTATACATATTTTTTCTGCAAATATCTTTTTC

TCGTAGAAAAAATAACATTAGCACTCTTTCATGACCTACTTTGTTAACATTTGCAACAAT

TCTTTTATGAGGATACATGGTGTAATATGTTTTTAATTAGGTAATTGGAGTGCCATCAGT

ATATATAATACATAGAGAATGTCATTGCATTGTAGGTATTTCTAACAAAATCAAGGAGTT

CATGGAGGAATGAAAAAGAGGGAATGGAAGGATACTGAGGAAAATTTGACCAACCAATTT

TCATTCTCTTGCAAGCAATTGAATCTAAAAATAGTAGACTCCGTGGACTATTTTCATTGA

CCCATACTAGCAAGCAATCAGTTAAGCAGATGGTGGTTGGATTTCATCTCTGTCCAAGGA

GACATTCAGTTTAGTCCATTTGGCACGCCATGCCAAAAATGAGAGAGTCCCAGTTTGACA

AGCCAACCCACAAATCAGGCCAATCCATAAACCCTGCACATTAAAACAAATCC

>**Gmax_MSTRG.17415.1** GeneID=Gmax_MSTRG.17415;Strand=-;Pos=Chr04:7719249-7719695,

CAGGCGAGTGAGACTATCATGGAAATGCGGGCTTTGGCGGGGCGGTTGGCGCCTAACTCG

TTCCCCACTCTGGTGGAGACGGCGAGGCTGAGAGACGAAGGGAAGACGTAGACCAACGCA

GTTGTCTGAATAAGGATTCCCATGGAGGCAATGGTGGATTTTGGGTTGAGAAGGAGTCCG

CAGAGAATGATCATGAGTTCGTACCACCACCACTCGAGACAGACGGAGACGCAGTTAGGA

ACAGCTAGTGCGAGAAGGGAAGACCATCCCCTGAGGCAGTCGGTGCTAGGGGGAACCCAC

GAGTCCTTGTAGACACGAGAGAAGTAAACAAAGGAAGAAAGGAAGATGAAGAGGTTTAGG

TTGGTCCACACCATGGCTATTGCAACGCCAGACACTCCCATTTTAAAGTGGACAACTAAT

AGGAAGTTGAGAGGGACGTGGAGGAGG

>**Gmax_MSTRG.548.1** GeneID=Gmax_MSTRG.548;Strand=+;Pos=Chr01:2753205-2753825,

CACGTACGTGGATGTGTTTTGCAAGCACTCACCAGAGCATAACAGCAGAAGCAAGAGAGA

GGCGAAAGAACCCCCAAAGACTCCGAAACGCTTCCCACGAGAACCCGTTCCACGCCGGCC

AGCACCACCCTCCGAAAACATAAACCAACTGGGCCACCACCACGAACCACCACGATCCGT

TGAGCACGACGGCGGCGCCCACGAGCCCCCACTCCAGCTTCACCATGAGCAGCCAGCTCA

GCACCGGGTGCAGCACCATGGCCATCCCGGCTATCGCCGCGATCACCATCACCTTGCTCT

GCGCCTGCAGGAACTTCGCCACCGGAAAATTCAGCGCGTAAGCGAACAGCTGCGGAATCA

TCCAAATGGCGAAGGTCCCCGCAGCCTCCGAAATTTCGGTGTCTTGCCCTATCAACTTCA

GAACTTGTCCCGCGAAGATGTACAAAGGGCACAGTACACAGGCCGTGCTGAGGAGTAACA

CCCATGATCGTTGCATGTACACTCCCAGCATGTCCAGCTTCCCTGCCCCCACCGCTTGTC

CGCACAGCGTTTCCAACGCGCTTCCCATCCCTAGCTGCTCGGAATCACATTTTTTTTAT

CCAAAATAAACATCAAAATAT

>**Gmax_MSTRG.89038.1** GeneID=Gmax_MSTRG.89038;Strand=-;Pos=Chr17:11025148-11027394,

TTGGTTAGTGGTCTTAATTTCATAAATAATTACTTCGTAATATAAATTTCATCTATGCCT

TGCAAATGATGGAAGGGACAACCCATATACTTCTTGGCATTTGGTTTGAAGCAGAATCCC

CTGATTACAAGCTTTACTGTTTACTTCTTCTCTTTTCCGTTATCTTTCTCAGTAAACGAA

CGGGGCTTTGATATTGAGATTTAAGAAACACAAGAGCTGCAGTACTCCTAAATTTCTGAA

TCTCTGATGATGGGTAATTGATGCTTTGCTTTTAGATGTATGAATAGAATAGTTTTGCCT

TGATTGCATTATGATCATTTGACACAATTATAACATTAGTACGTAGACCAACGTCTTGTT

CTACCTATTGATTTAAAATGCATTAATCTTCTAATTTTCAAATCTGAAGTTCCACTGCAT

CCGTTACTACAATTACAACTAGCAATGATATGATTCAGAAAACACCCGAGATCACAAATA

CTAGTGAGATTGAGAAGGGACTTAGCATATTGTACATGTCTAATTCTTCTCACCCCAAGT

GTTGTTATATATGAATTTGGGGTGATAGAGCCATAGAGGTTTCTTTCAACGTACAAGTTC

ATTTACCTGATTTCTTGGAACTTGTTCCTGTACCTGTGCGGGAATACATACTAACATTTG

TGCCTTGGTAGCTTCTTGCACCCAATTTATTCTCACAATAAATGTTAACAACAAAATCAA

GCATGCAACAACACCAATCAAGAATCCTATAATGAGTCCAGCAAGTCCTAAACGAAGCTT

GAAGGCAAAAACCACACCCAATGGTAAGGCCAAGAAGTAGAATCCACCAATATTGGCATA

CATTCCTAGCCATGGTCTTGCAGTCCCTCGCACTATGCCTCCACAAACCGTCACAGGAAA

ATTAAACACTTCCACCAGAGCCATCAAAAACATTGTCTTCTTCACACCCTTTACAACCCC

TTTATCATGGCTAAACAAATTCCCCCAAACCCCTCTAGAAGCCACCATCATGGAACCACC
GATACAACCCGATATAACACCCACTGCCAAAGACACACGTGCTGATTTATAAGCTTGACC
AGCACTATTAGCACCAAGCTCATTGGACACACGAGTGGAAACACACGTGGCTAGAGAAAG
CATCACTGAGTAAAGCAAATAGTCAAAGTTCAACACAATGGCCAGAACCCCCAGGGCTTG
TTTTGCGTTTGCCAAGTGGCCAGTGAGCAAAACTAGAATCTCGTAACACCACCACTCAAG
GCACGTGTTGAGGCAGCATGACCCACTAAGCTTCATTAGCCTAATCCAATCCATCACATT
CTGATCCCACCACCCTCCTTCCTTCCACAACATGCCCTCATTTCTTCTCTCAAGAACCAC
AACATAAACCGCCAGCATAACCATAACCATGAGATCGGTTATCCAAACCGCAATCGAAAC
TCCTCGGAGTCCCATGGTTTTGGAGAGTACTATGTTAACGGGTATGTGAAAGGCTAGTGC
CACAGCAGAACTAAACATGGTGGGAAGAGTCATGCACTGAGAGCTGAGGTAGGTTTTTAA
GGGACAAAGGAGTGAAGTGACCAACAAGTCAGGTATGAGATAGGAAACATAGGTTTTGGC
CACGGTTGAGATCTCTTGTTGTTGGCCAAAACAAATCAAAATCTTGTCAACGTTCAACCA
CATGAAAGATATGGGGAGTGATGCCAAAAGCAACAAGAGGGTTGTCATGAGAAGGGTCTT
GTGGAGGAGCCTCACGTTCTTGGCTCCATGCGCTTGCCCACAGATTGGTTCCATGGCGCC
GCTTAGACCGTTCAAGACGGAGAAGCCGGTGATATTAGCAAAACTGAACCCGAGAGCGCC
ACCGGCTAAGTTCAGCTCCCCAAGGTGGCCAAGAAAAGCTGTTGTTATGGCTGTCTTGGC
AAACCAAGCCAAGTTCATGGCCACAAGAGGAAGGGCTATCCCTCGTTGAACTCTTAGCTC
TTCTAGTACCATCTTTATGAAATTTGCATTGAAAGGGCATTTTTTAGTGTGTGATGAAGG
AGATGAGGGCCGATGGTTAGCCTTATCGTTTGTGCTTAGGTTCTCTACTTGTTTAGATGT
TGCTGACATTTTTGCTGAGATAATGTTATGGTATGATTGTTGTGTTGTGAAGTTTCACTA
GCGTGTTTCTTTTGTTTTATATAAGATTGGTTAAGCAACCAATCTTATATAAAACAAAAG
AAACACCCTATTGGAAGTTGTTTCTCA

>**Gmax_MSTRG.10879.1** GeneID=Gmax_MSTRG.10879;Strand=+;Pos=Chr03:549584-549901,
AGAACACCATTGGCGAACCTAATGAGAACGGTGAAAACGAGAGCATAAGCGGCCAATTCC
TTAGAACCAATATGGCCAACAAAAGCTTGGCTTATAACACTGATTCCAAAGGTGGAAAAC
CTTGTGAATATGGCTGGTGCCGCCACTATCCACATAACCTTGCTCTCATTCCACACCCTC
TTGGCCAATGATAACTCTTCTTCTTCTTCTTCTTCCTCTTCCTTTCTTCTTCTCAACAGC
TTCTGCTTCAGATCCCCCTCCATTTTTATTTTTTGTTTTGGGTTTGGGATTGGTCAAAGA
GAGAAAGAGAGAATGGCG

>**Gmax_MSTRG.70933.1** GeneID=Gmax_MSTRG.70933;Strand=-;Pos=Chr13:37697679-37699947,

TTCGAGAGTCCCACTTCTCACGCGAGAAAATAATCTATCATCAATCATATGGTTCTCCAT

TGTCAATTACACGTAGTTTCCTAGTTTTTTTATAGTATTCTTTTCACGGTGGGATACATT

ACTCCCCATCTATTCTTGATGTGTTCCCGTGAGACTGTTACATTATATGCCAAGATTTAC

TCTCTGCAACTCACCAACACTCCCAAATAAAATATAAAAAAAAGTGTGAGCACTTTCTCA

AAATGAATATAAGCAAGAAACGCTTCACCAAAATCATGTTCAGTGTTCACCCTATCTGAT

AAACTTCTGCATAATAGAATGCAAACCTTTTTCTTTTTTTTTTACCATAAGTCCAATTCC

ATTCAGTTAAACAACAAGAGGCCGTTCTTTATCATTGTCCCTAGAGAGATCCAATTTAGT

CCATTTGGCACGCCTTATGAAAAGGAAGAGAGTCCCACTTTGACAAAGTAACCCACAAAT

CAGACCAATCCATAAACCCTATATCATAAAGCAAATTCAACACAATCATATTAATAAAAC

AACTGAATGCAAAAAGATTGATATCAGAACAAACATAACCAGACTATGCAATAAATATTT

TATTTACCTTGTACTGCAAATTGGTCTTAAATCCTAGGAAACATGATATTGGCAAACCAA

TAAGATAAAATGTAGCAAGGTTAATGTAAGCAGCCAAGTGCTGCCAGCCACATCCTCTGG

ACACCCCTGCAGTATTAAACACAATTTCCTCCTTGAGAATTTTTAAGTTAAAGTTATAGT

TTCAAAATTTAAAAGAAATGACAAATGAGGAATTCAAACCTGACAAGACACCTTGGATGG

CATCAAGTAATATAGAAATGGCAAGCAAGGGTGTCACTGAAGCAAACTCCTTTTTAATTG

TAGAACTGTCGCTGAAAAACTGAATCCAGATATTGTGACCAAATCCAAGTGCCAAAACAA

AACATAATCCAAGGAGGAGAGAGAGCTTCAGTGTGACACTCATCGCATGTTTAGCTCGTT

CCGGGTTGCCTGCTCCCAATTCATTGGATACCCTTGTGCTGTTAAAGTTAAATTTCAACT

TTTCAACACATGTACTAGAATGTTGATTCAGAAGATATGCAACAATGCAAGCAAAGGAGA

ATGTGTTCAATCAACTAACCTTGCAGCTGCACTGAGACCATAAGTGATCATGTAGGCAAT

GAATTCTGTGTTTATACTGCAGATTTTTTTGAAGATTAACAATAGTAAATTATATGAAAC

TTTTTTTTTTCATTTTAATTAACAATATTAAAATATATGAATCTGTTTTCGAACATATAA

AGCTATTAGAATTTAGAACCTTACCATATTGCAATCAACGAAGTGGTTATCTGTGAGTCG

GGCATTAGTCCAGCTAAGAAAACCAAAACTTCAAAAGCCCAATACTCCAAACTTGCATAA

CATAAACATCAGGTAATAAGATGATATATGAATCTGGACGAACCATGGTTTTTACCCTTG

TATTGAGGTGTTTTTCACTTTAAACAAATCGTGTCTCTGATTTTTCTTAATTTCTATTTT

GCGCTCTATAATTTATTGGTGCTCCTAGGTTTTTGCAAATTTGGGAGAATTTATTTCCAC

TGTCTATTACTCTATTATTGAGTTTGTTATTGTGTTGGCTTGTTTTTCCAATCAAGATGA

ACAATCAAAAGGGCTAGCTAAGTGAAAGGTTATTAGTAATTCATACCATACCATTGCAGC

AGAGGGCAGAGCTAGTCTCATGTTTGTAAATACGTAACGAAATGAATGCGTTGAAAATCC

TTTCCATGTCTGCTTGAACTTCTTAGCATACATTACATACAAGGCCAATAATAGCAATGA

TATCCATAGTGAAATAGAAGCAGCAACTGGTGCACCTGTAAAACTCAGACCTGACCACTG

AACCAAGCCATATGCAACACCAATATGAACCAGCATTGGAAGAGCAGAGAGTACAACCAA

TGGAATTACTACAGATTGTGTCTGGAGAAACCTCAAGATGTTTTGCAAGAAGCTATAAGC

AAACACTCCCGGGATAAGAAACTTCATATAAAGTGCAGCTGTTCTTGCAATGTCGGGAGA

TTGATGAAGCAACACTAGGATGGGTTCTGTATAGAACCAAATAATGGATATAATGATTGA

AAAAATAAGAGATATGATGCAAGAGGCTTGTAGGTAAATTCCCAGCATTTGGTATTCCTT

TGCACCAAATCCTTGTCCGCAGAGTGTTTCCAGTGCCCCACTCAAACCA

>**Gmax_MSTRG.46909.1** GeneID=Gmax_MSTRG.46909;Strand=-;Pos=Chr09:33369122-33369354,

AGGGCACTGATGACACCTTGGTAAACAGAGATAGAAGAGAGTTCAATGTCTCCAATATGG

CCAGCATAGATGGAAGTTGAGGAGATTGTGAGAAACTGAAACAGTGCAGACAGTGCCATA

GGGAATGCTATTCTCCATATCTTCACTGTTTCTGTCCACAAAACAAACTTAACATCCTTC

AAGCTTTTCACCGGCAAGTAGTCAGATTCTGAAGTGAACTTTTCGGTCACTAA

>**Gmax_MSTRG.58090.1** GeneID=Gmax_MSTRG.58090;Strand=-;Pos=Chr11:8552497-8553372,8553746-8553935,

GAAAGTTAATGTTAAACATCCTAGAAGAGTAAGTATGGGAGAAGAAATAGTAATTTTATT

ATTATATGCTAGCTTTCCCATTTGTATAGAAGATGAAACGAACATACCAGCAACGTTCCC

TCAAGGCTGACAGTAGGGGGTGTTACAGCCAGGGCTATAAAGTATGGAATCAGCACCTTA

TGCATCTGCGCCTCCTGGATGACCATCCGATCAGGTGTAAAAATATTGGGAAATAACCAA

GGAACCGATGTTCCAATAATCCCTAACAACAATCCAAGTATAGCTCCAATTATCACGAGA

GACCTTAGAAGCATTCGGGCCTGTTATGAAAACTTGAAAAAGGAGAACTGAGAAATGAGA

ATTGAATATATTTCCATAAAATAACATTGGTCAGAAAAGATAAATGGATTACCATCTTAT

AAAACTACACTTCCTAACTAAAAATAATTTATCCTCTTACATAACAAAAACGTAAACTAG

GCACAAGAGAGATCAGAAACCACAGATATGAGGATTGGACATCAGAGTATGCTTTGTAAT

GAGAAACACTAACCTTTGACAAACTCCGATTTACTCCATATATCAATTCAGGCATAAATG

ATTGAGAGGTTTGGGAGAGAGGTTCACCCCATACGGTACACATTAAATAGGTTTGGACCA

TGACCTGAAGAGGGAATGAAGTTATCACTGGCCTTGAGTTTTTGTTTTTAAAAAAAGCTC

AGATGGCAGGCAAAGGAAGGAATAGTCACAAAACAGGAAACAAAAAAAACACTAACTTGA

TGAGCAGCCATTGTATGTGTACCCATTGATGTAGCAAAATATATAAGTAGTGCGTAAAAA

GCCACCTAAACATCAGAACTTCAGATTATATTAAAAGGTTATACCACTCTAGACAAAGCC

ACAGATCTAGTATAATACTTGAGACAACCTTTGACATCAATGTTATAAAAACAGGAGCAG

CAAGCCCAAATATCGTCAGAAGTTCCTTCCCTGAAGGAATGGAGAAGGCAAGTGCATTAT

ATCCCTTCATATTTAGATTTTGAATCATCATGTAAGAAGCAACAAC

>**Gmax_MSTRG.5527.1** GeneID=Gmax_MSTRG.5527;Strand=-;Pos=Chr02:3607713-3607922,3609643-3609753,

TATTTATCCCTTTTACCTTTATTTGGGAATAAGGATCATCTTTACCAATATAGGAGAAAT

CATTATCAAAATCTTTATCAAGATAAGGGAAATACTTATTAAGTTATAAAATCAGGGTAA

TGATCCACCAACCCGCTAGTTCTTTTTATGAGGCACAAACGGGAGAATTTATCCTGGAAG

CGGAAGAGCTACTGAAAATGAGTGAAACTATCACAAGGGTTTATGTACAAAGAACGGGCA

AACCTTTATGGGTTATATCCGAAGACATGGAAATGGATGTTTTTATGTCAGCAGCAGAAG

CCCAATCTCACGGAATTGTAG

>**Gmax_MSTRG.34860.1** GeneID=Gmax_MSTRG.34860;Strand=-;Pos=Chr07:19193297-19193579,

GATTATCTAGCCTGGCAAAGTACAGAGTCGGAAGAAATTATAACATAAAGTAATGTAAAA

TCACTTATATGAGAAGATGTATGAATCAATAATTAGCCAAGTATGACATACAGCAGGTAT

ACCCATGATATTCATAAGAATGCCAGAGCCAAGCGTAAGCACCACAGTTTCAGCAATTCC

AAGAGTTGCAGCAAGTGCTAAAGAAGTTGATACTGAAGGAAGGAGCTTCTTGCTTTGGTC

CTTTCCTGAGAAATCATGACCCACATGAGATAAAAATTATTTG

>**Gmax_MSTRG.17697.1** GeneID=Gmax_MSTRG.17697;Strand=-;Pos=Chr04:9417435-9417745,

GCTATGTGAAAACTTAATTGACTGAATTAGAACTTGAAAGACTAAAATTATACATTTGAG

ATATCTAAAAGATCCATTGGAGGACAATATTTTATTGTCCTTTTAAATGATAAATACCCG

TTGTTCCCAATTTATGCAACTGGTTATGGTAGAAAGTAGAACACATTCAACAAAAGAACC

AACTTGTACACCAATCCAAAGTCCTTTTCCTCTTAGTTTTGCCAAAAAATGACAATAAGAC

TGCCATTGGAATCCCGCATAGGTAAAATGCTCCTATATTGACATAAACCCCTATGTGTTG

CCATCAACAAC

>**Gmax_MSTRG.46372.1** GeneID=Gmax_MSTRG.46372;Strand=-;Pos=Chr09:18851935-18852149,

CGGGAGGCCTATTCTGATTAAGTGCACAACAAGAACACTTTTGGAGAAGATTCCAGCTCC

AAAGTACAATCCAAATCCAACTGCAAAAGACAATCCAACTCCTAAAACAAAACTCATCTG

GAGAGTTCGTGTTGCTGCAACAAGCACCTTTTCATAGTCTTTCTCAGCAAAGGAACAAGC

TAGAATTGCCTGAAAAGGAGAAAGTAATTAGTAAT

>**Gmax_MSTRG.104177.4** GeneID=Gmax_MSTRG.104177;Strand=+;Pos=Chr20:36605682-36605828,36606807-36609160,

AAATACCTTGGATGAATGAGAAAGAAGCAGAACAATTATCGCCATTTCCTTATTTTACTG

CATTAGCTTAGTTACCTTAGTATAGCAATCCTAACAGTTCTTGCCTCAAATTTTTCTTTT

CAGGAATATTCGATGGATTTTGCACATAGCCATAAAGACTTGGAAGTGGAGGCAAACCTG

TTTTTCCCAGTTTGTACAACTTGTGATGAGAGATAGCATAATTGTTTGGCAGAAGGCACC

AGTCAGTATTCCAATCCAAAGGCCTTTTCCTCTTAATTGTAGCCAGAAACCCAATATGGC

AGCCATTGGAATTCCCACAACATAATAAGCTCAAAGGTTTACATATGCTCCTATGTGCTG

CCACCCACATCCTCTAGCAATACCTATAATGAAGGCATAACTCAGATTCCATTGCTTAAG

AAGAGTAATGTGCATTGAAAAATAGTTCAAACAAGAACTAACCTGAAAGGGTACCGTGTA

ACGTGTCTACTATAACAGATATACTTAAAAGAGGAACCATATCTGTGACATAATCCACCA

CATCCTGCTCATTGCTAAATACATAACCTAAAACCTGCCTGCTAGCAAAAATGATTGAGC

TCACCAGAATGGCCTCAGAAACTGCTAGAGTCATGGCAGCAGAAACAGATACTCGTGCTG

ATTGTGGACTTCCAGCTCCTAATGCATTTGAAACTCTAGTGCTGAACAACCCGCCCAAAA

GAGAAACCAATCAGAAAGAGGAAAAAATTCATTTACTATAATATCATTCTACATGAATAC

GAATATGCACAAATTGATGAGCTTAGGACTTGTGTGAATCAAAACCTTGCTGCTGAGCCA

ATTGCTTCTGGGATTGTGTAGATAGTTGTGATGATGGATAAACTGGTCAGAACATAGTTA

ACAATAACAACAAATTAGCAACACTTGCATGAATGGAAATCAAAATTTGGCTGAGTAACA

ATTTCAACACAGATTTTAAAACACAAAGCCATTCATGTCTGAAGGACTAGGATAAGTTGT

ACATACCATATGGATAAGACTGAAGTTTCAAGCTCTGGATTTGGTAGAAGACCAGAAAGC

AAGGTTAGCAGCTCAAATGACCACCACTCAAGGCTGCAACGATCAAATAAGTAAGAAACA

TACCATGGTTGAAAATTAAATGGGAAATAAAAACACAACACAAAAGATAAAATATAAGAG

GAAGAAACTCACCAAACCATTCCAGCTGAAGGAATAGCATAGGTGAAGAATTCACCAATC

CCATGGAATAGTTCCATTGAAATTGGGACTCGAGTCCTTTCACATTCAGTAGAGAATTTC

ATATATAAGCCAAGTAAAATCACATTCAGCCAGTATGAAGTACCAATTGAAAATGCTGCT

CCCAAGTTACCAAATCCAGATTTAAAGACCATTAACCAACTGAAAGCCACATGGAAGCAA

AGAGTAATGGAGGAACTTATGACAAGGGGACTGATCAAACTTTGCATCAAAAAGAATCGA

ATCAAGGCCTGAAGTGTTGCATAAGCAAAGAGAGCAGGGATCATGCACAAAGCAAATTTT
CCAGCTTCTTGTGAAATCGAAGGGTCTTGGCCAAGAAATATGAGTATCTTCTCCAAGTAG
ACCCAGAAAAGAGTCAGAGGAAGACAAGCTAAAGTAAGAGAGACAATAGCAGTGTATATT
TGAACACCAAATTTTCGATATTGCTGTGCTCCATATGCTTGCCCACATTGAGTTTCCAGT
GCACATGACATTCCAAACTGTTTGACATGTACAAAAAAGACAAAGTGCAATTAATTCTTA
TAATTTTTTTAGAGACCATAGGTATCTTCACATAAGAGAATCCTACTCGAACTTAAGCCA
TTTTGTGTTTTAATTCATCTTCATCTCTTTTGTTTTTAATAATTTTCCATCCTTTGATAA
TTTGGGTTCAATGCAGTAGTAGGTCTCAAAGCTTAAGAAGAAAGCAGTGAGATGGGAAAG
GAAGAGACTTACAATAAGACTGAAGCCAGAGACAGCACAGAGAGAGATGGCAATGGCTGT
GCTAGAGAGAGCAAGCTTACCCAAGTGACCAACCATCATCATTGAAATAATCTGTAGAAA
ATACTGTGACAAAGTCACAGTAATCATAGGAGCTGCTAGATAACCCACTCTTTTCATTTC
CTGAGAAAAGACAGTCCATGTTATGGCTGAAGCTGAAGGATTCTCCGGCTCTGAGTCCTT
ATCTAAGAGACTATTTTCCATATCCTCTCCAAAGGGTCACAGAATCACTGCACCTTGTCG
AAACTGGTAGAAATAATGGCAGGGCTTTGGTGTTTTGTATGCTAAAAGGTTTAAATATTT
ACAAGTGGCAGCAGCAGTAGTTCTTCCAAGGTTTAAATATTTGTTTCAATAATGACTGTC
TCAGAGTCTCTCATGGGCTTCCCCCTCTTATGTAGACGGAACAATATTTCAAGTAAATTT
TAAAATTTAAAATCATTCCATCTAATCTAATTTAATCTAAA

>**Gmax_MSTRG.85884.1** GeneID=Gmax_MSTRG.85884;Strand=+;Pos=Chr16:35991281-35993500,
ACATTCAATGCCCTGCAAGATGTAATTGAATTTTACTGGAAATAGCAGAAGTCCTTAGAG
GAGAGGCATATACCCCAGTGAATTTTTCTTAAGGACAAAACTTAGGTGCAGCAGTAACTT
ACGTGCTTTTAGTATTATTTTTTAAAATCAAAATTGAAGTTTCACATGGGTAACTTATAC
TATTTTTTTTCCTATGTAAACTTGTATTATGCAAATTACCGAGATAAAACTCTAATTTTA
ATTTGAAAATAACAAAAAAAAAAAACACTTAAGGAACTGCATCTAATGTTATCTAAAAAT
CATAAAATAAAAATGCTATCCTCTGTCTCTACCCTACTGCCCTATACAGTTAAAGACAAA
TTTACGAAACATCTTATCCTCTTCTCTTCAGTTTTTGTTTTTCTCTTTAAGGAAGTTGAT
GAATTTTAAACCAATCACTGTTCATTTTCATCTGAGTCAGCGAGCAGCAAAGAATCTTCA
TTGCTTTCATGTTTGAGTGGCTTCTCTGGATCAACCTCACGGCTCTGATCAACACCAGAA
GCTGCTCCTCCCATTCCTGTGAGTTTCTTGGCTCTTTGAGCCTCGAATTCCCAATCAGTT
CTACAAAGAACCACCAACATGGTCACTGCGCAAGACCCTTGAGCTGCAAGAAGGCCAAGC
CAGAGCCCTTGAAAGTCATAGCCAGTGAAAAAGGCCAGCCAAATGGACACAGGCATTCCC

ACAAGATAGAAGCATCCCAAGTTGATGTTAGCACCAACTTTAGGCCTTGCTGTGCCTCTC

AGCACCCCACACCCTGTTGTTTGAGGGCAATTCCCAAGCTCACAAAGACCAATTATAGGT

AACACCATAGATGTCAAAGTTATAATATCCTTGTCCTTTGTGAACATGCTAGCCCATGTG

TTCCTAACCATGAGAGCAAAAACCAATGCCAAGACACCTGACATGAAGCTGCAAGACAAC

CCCACAATGGCAGAAAGCCTTGCCTTTGAGGGCTTTTGTGCACCAAGCTTGTTGCCAACT

CTTGTTGACACACTGAAGCTCAATGAAGAAGGGAAAATGTAAAGCAAAGAAGTGGTTTGA

ATGAGAATCCCCATTGAAGCCACAGTAGCCTTGGGGTTCACCAACAAACCACACAACAAA

ATCATGATCTCATACCACCACCACTCCAAGCACACCGAAACACAGCTTGGAATGGCCAAA

TTGAGAAGAGACTTCCACTGTGTGAAGCACTCAAAGGAGAAACCACCCCATGTTTTCTTG

TGAGTGCCAGAGAAGACTATGTAAAGAATCAAGGAAGCCACAAGGTTGAAGTTTGTCAAC

ACCCCACCAAGAGCAACCCCTTTGATTCCAAGCTTGAGGTGAGCCACAAGGAAATAGTTG

ATGGGTATGTGAAGGAGAATAGAAAAACTTGCACAAAGAGTGAGAGGGAGAGTAATGGAT

TGGGTTCTGAGGTAAATTCTCAAAGGGTGTAGGAAGGATTGTGCTATGAGGTCAGGAATG

GAGTAAACAAGATATGATTGGGCTTGTGTGGCTATGGCCACATCCTGGCCACAAAGGAGG

AGAATTTGCTTCATGTAGAGCCAAAGGAGTGATATGGGGAGGGAAGTGAATAGAAGAAGG

AGAATGGTTCTTTGTAAGCAGAGGCCAAGGAGGGTGAATCTTTTGGCACCAAAGGCTTGG

CCACAAATTGGTTCCATTCCCACAGCTAGGCCTGAGAGAATTGAATAGCCAGTGATGTTT

GCAAAGCCAATAGCTAGAGAGCCACCAGCTAAAGCTAGCTCACCAAGATGGCCAAGGAAG

AGCATGGAGATCATGGAGCGGCAGTAGAGGAGGAGGCCAGTGAGGATCATTGGGATGACA

ATTTTGGATATTGAAAACACTTCTTTGATGGCTTCAGGGACATGATGGGGTTTTGGTGCT

GTTGTTGTTTTTGTTGCTGTTGTTGATGGTGGTGGTGTTTTTGTTTTTGTTGTTGTGTTG

TTATGTTGATGAGGTGGTGGTGGTGGTGTGGTGGTGTTTTGGGGTTTGGAGATCAAAGGG

TTAGTGATTGAAATTGGGCAATCAGGAGGCGAAGTGCTGCAGCATTGGCAATTGCAAGTG

ACGTCAAGAGTTGGATGACACATGGTGTGTTGTAGCTACTTTGTGTCCGTGTGGAGGGGA

AAAAAATGGTATTGTTTTGAATAGACTTGAAACAGAGAGAGAGAGAGAGAGAGAAAGATG

>**Gmax_MSTRG.4163.1** GeneID=Gmax_MSTRG.4163;Strand=-;Pos=Chr01:54702238-54704775,

TTTTTATTTCTTCTTTGTATGAATGCATGCGTTTATGCTCTGCCTCTTCTCCGGAATGTA

CAATTAAAATTAGGACAATCTAGAAAGAATCAATTTCATGTTATAAAATCTAGCTTGAAT

GCAAAAATATAAAAGAATCAACGTGACGTCACTGCAATGATGATCGATCAAACTAATGAA

TCAGAATGAGAGCATTCCTTTGTGGCGCCCGAACTGAACAAGCATTTCTGATCTTGTTCC

TCCTCCTCCTCACTAGAATCAGACGATGATGTGAGTTCTTTGGCTCTTTGAACTTGACCC

TCCCAATTGGTTCGAGCCAACACAATTAGCATGGTGAACATACAAGAGGCTTGAGCTGCC

AATAAACCAAGCCACAAGCCTTTGAAATCAAAGCCGGCAAAGAAACTCAACCTCACCGCA

ACGGGCATTCCCACTAGATAGAAGCAACCCAAATTTATATTCGCTCCCAACTTGGGCCTT

GCGGTTCCCCTCAAAACGCCGCAAACCGTTGTTTGCGGACAATTTCCAAGTTCACATAGT

CCAATGATGGGCAACACCATCGATGTCAAAGCAATGATCTCCGCGTCACTTGTGAACATC

GAAGCCCACACGTGCCTCACCGAAACTGCGAAAAACAAAGCCGAGAAACCCAAACCGTAA

CTGAAACATAACCCCACGAGGGCTGCAAGTTTTGCCTTTTTGGGATTCTCCGCACCCAGT

TCATTTCCAACACGTGTGGAAACGGCAAAGCTTAGGGAAGATGGGAAAATGTATATCAGA

GCAGTGGTTTGAATCAGAACACCCATTGATGCAACCGTTGCTTGGGGATTAATCAATAAC

CCGCATAGTAAAATCATGATTTCGTACCACCACCACTCCAGGCAAACGGAAATGCAACTT

GGAATTGCAAGATTCAAAAGCGATTTCCAACCGCTGAAGACGCCCTTCAACGAAACACCG

GGCCATGTTTTCTTGTATACGCCGGAGACCCAGATGTAGAGAATCAACGAAAAGACGAGG

TTGAAATTCGTCCAAACAGCGCCTAAGGCAATGCCTTTGATTCCAAGCTTAAGCACAGAG

ACAAGGAAGTAGTTGATGGGGACGTGAAGGAGAATAGACAGAGAGGCAGTGTATGTGAGA

GGCAGGGTTATAGATTGACTTCTTAAGTAGATTCTCAAAGGGTGTAGCAATGATTGTGCT

ACAAGATCGGGAATTGAAAATAGTATGAAAGATTGAGCTTCGGTGGCGATGTCTTCTTGT

TGGCCACACAGGACCAATATTTTCTTCATGTTGAACCACAAGAATGATATAAAAACACAA

GTTAGAAGAAGCAAGACCATTGTCCTCTGCATCGCGAGGCCCAAAAGTTTGAACCTTTTG

GCCCCGAAAGCCTGGCCGCAAATGGGCTCCATGCCCATGGCGAGGCCCGAGAGAATAGAA

TAGCCCGTGATGTTGGCGAATCCAATGGCGAGAGAGCCACCAGCTAAGGCGAGCTCACCG

ACGCGACCGAGGAAGAGCATGGAGATTACGGAGCGAGAATAGAGTAGTAACCCGGTTAGC

ACCATGGGTAATGCTATGTTGGCTATGCATTTGACTTCGTCGAGGGCGAGGGAAAAATGG

GTTTTATGGGGCTTTTGGTTATTTTGGCCTTGTTCGAAGAATGTTGTTGTTGTTGGGGGT

TTGGGGATCAAAAGGACGAACATGTCAGGCTCCTCATGAATTTTGGTTGGGGGCATGTTT

GGATGGCCCTCATTGCTTTCACACAGAGTGGAGGGAGATGATAACTGGCACATGGCTCGG

AGACAGAGATAGTTATAAGGGAGTGCGTGTTTGTGTGTAAGTCACAAAAGTTTTGGTTTG

GGGATTTGCGTTTTGCAACAAGAGTGTGTAAGGAGAGGTATTTAAAAGGGGTGGGTCATT

TGAAGATTTTTGGACGTATCTGAATTTATTTATGGAGTATCTGAGACGGCCACTAATGGA

AAGATAGGGTCGACAGAGAGTATTGGCGTTTCCGAGTAGTACCAACTCAAATGCCTCCTC

CTCACAGTTTTTTTTTTATTATTTTAAAAAAGAAGCAAATATTCAAAGCGTATTTTTATG

ACCGTCTAATATCGTTAGTGCAGGGATTAGTGGTGAAAGCTAGTGTGGGCTTTGACGTAC

GTAATATATACTACTCCTATATAGAGTGCCTCGTACTGTAGGAAGGAATTACCGGTTCAA

ACGAATAAAGGTCAAAAGCGTAGTAATTCCATTCCATTCCAAAATGAGTTAAAAATATAT

ATTTAACTAATGCACTTAATTTGTTTTGTGTAACCTCATATGATCGCTTTATATGAACTG

GCCTATAGCATGAAGAAGTGTTAGGATTTGATGTATCCGAACCAAATAGGCAGAATTGCG

TTTAGACGTCTTTCACTTTATGAGGAGAGGACCTATTATGAAAACGGCAGCTTGACTTAC

TGTTTCTAAGTTGAGAGTTTAATTTGGGTCTTTTATCTTTAAAACTTAAAACTCGTGTGA

AATAAATGCCATGTCGGC

>**Gmax_MSTRG.6397.1** GeneID=Gmax_MSTRG.6397;Strand=+;Pos=Chr02:7922530-7922920,

ATGGAACAAAAAAAGTGAGGAGATGAAAGAAGACTTTACAAGAACACAAAAGCCTGTAAC

TTCAGCAAAAGAAGTGGCAATTGCAACCCCAGAGAAGGAGACAAGTACACCAAGATGTCC

CACCATCATCAGTGACACAACTTGTAGAAGGTACTGACACATATTCGCAGCCACCATTGG

AGCTGCCATGGAGCCTACCCTTTTAAGCTCTACACAAAATGCATCATCATCCTCTAATGG

TGCCACCTCACTCTTTCTTAGAAGCGGTGTTGCTTCCTCCTTACCCATCATCTCTCTCAG

TTTTTCTATGCAAAGCAACGTTCATGAAACAGATGTTAGACAACAAGATGATCAACAACA

CGAGTTATATAGAACAAGATCACAAGAAAAA

>**Gmax_MSTRG.46910.1** GeneID=Gmax_MSTRG.46910;Strand=-;Pos=Chr09:33369728-33370433,

GAGGAAACCAAGAAATAGAAAGAGTTTTATTGAATTCAATGGAAAATGAAGAAAAAAAAT

GGAGAAAAGTGTGTAGTAGGAACCAATCTAAACAGCTGCAAATTAGTTAAAGTTGATAAT

CTTACCAAATAGAATAGGAACCAACATCAATCACAGGATTGTCTAGTAGACCAGCAAGGA

GAATAATGCATGTACTATACCATTGGTCTAAGCAACTCATAACAGATGATGCAAGGCTTA

ACTTAGCAAATGACCACAAATCCCTAAATGCCATCCATGAAAATCCAGTCCATTCTTCCT

TACACCAACCAATGGTATACACAACCAAAGCCGCAGCATAAACCCACCCTATGATATTAG

TCACCATGGCTAAGCCAGTTGTACCCCAACCAAATACATTTATGAAGATGTAAAGCAGCA

CATTTTGTATGACCAAAACAGCAAGTGCTATGCAAGTAATAACTTTAACCTTGATCTGGG

ACTGAAGAAATGTCTGAAAGGGAAAAGCTATTGCACAGGAAAACATGTAGGGAATTACTT

GTATAGAATATCTTCCGGCAAGATCAGCTATTTCTTGGTCTTGGCCAATAAAATTTAAGA

TTGGTGTGGCGTATACATAAATAGGTAAGAGGATTATACAAGTGGCAGTAAGTATAATCC

ATGATCTTTGAACATAAATACATGTTGATTGAATTTGCCCTGCTCC

>**Gmax_MSTRG.104177.6** GeneID=Gmax_MSTRG.104177;Strand=+;Pos=Chr20:36605721-36605828,36606791-36608312,36608596-36609241,

CGCCATTTCCTTATTTTACTGCATTAGCTTAGTTACCTTAGTATAGCAATCCTAACAGTT

CTTGCCTCAAATTTTTCTTTTCAGGAATATTCGATGGATTTTGCACATACTGTGCCAAGT

GCAGAGCCATAAAGACTTGGAAGTGGAGGCAAACCTGTTTTTCCCAGTTTGTACAACTTG

TGATGAGAGATAGCATAATTGTTTGGCAGAAGGCACCAGTCAGTATTCCAATCCAAAGGC

CTTTTCCTCTTAATTGTAGCCAGAAACCCAATATGGCAGCCATTGGAATTCCCACAACAT

AATAAGCTCAAAGGTTTACATATGCTCCTATGTGCTGCCACCCACATCCTCTAGCAATAC

CTATAATGAAGGCATAACTCAGATTCCATTGCTTAAGAAGAGTAATGTGCATTGAAAAAT

AGTTCAAACAAGAACTAACCTGAAAGGGTACCGTGTAACGTGTCTACTATAACAGATATA

CTTAAAAGAGGAACCATATCTGTGACATAATCCACCACATCCTGCTCATTGCTAAATACA

TAACCTAAAACCTGCCTGCTAGCAAAAATGATTGAGCTCACCAGAATGGCCTCAGAAACT

GCTAGAGTCATGGCAGCAGAAACAGATACTCGTGCTGATTGTGGACTTCCAGCTCCTAAT

GCATTTGAAACTCTAGTGCTGAACAACCCGCCCAAAAGAGAAACCAATCAGAAAGAGGAA

AAAATTCATTTACTATAATATCATTCTACATGAATACGAATATGCACAAATTGATGAGCT

TAGGACTTGTGTGAATCAAAACCTTGCTGCTGAGCCAATTGCTTCTGGGATTGTGTAGAT

AGTTGTGATGATGGATAAACTGGTCAGAACATAGTTAACAATAACAACAAATTAGCAACA

CTTGCATGAATGGAAATCAAAATTTGGCTGAGTAACAATTTCAACACAGATTTTAAAACA

CAAAGCCATTCATGTCTGAAGGACTAGGATAAGTTGTACATACCATATGGATAAGACTGA

AGTTTCAAGCTCTGGATTTGGTAGAAGACCAGAAAGCAAGGTTAGCAGCTCAAATGACCA

CCACTCAAGGCTGCAACGATCAAATAAGTAAGAAACATACCATGGTTGAAAATTAAATGG

GAAATAAAAACACAACACAAAAGATAAAATATAAGAGGAAGAAACTCACCAAACCATTCC

AGCTGAAGGAATAGCATAGGTGAAGAATTCACCAATCCCATGGAATAGTTCCATTGAAAT

TGGGACTCGAGTCCTTTCACATTCAGTAGAGAATTTCATATATAAGCCAAGTAAAATCAC

ATTCAGCCAGTATGAAGTACCAATTGAAAATGCTGCTCCCAAGTTACCAAATCCAGATTT

AAAGACCATTAACCAACTGAAAGCCACATGGAAGCAAAGAGTAATGGAGGAACTTATGAC

AAGGGGACTGATCAAACTTTGCATCAAAAAGAATCGAATCAAGGCCTGAAGTGTTGCATA

AGCAAAGAGAGCAGGGATCATGCACAAAGCAAATTTTCCAGCTTCTTGTGAAATCGAAGG

GTCTTGGCCAAGAAATATGAGTATCTTCTCCAAGTAGACCCAGAAAAGAGTCAGAGGAAG

ACAAGCTAAATAGTAGGTCTCAAAGCTTAAGAAGAAAGCAGTGAGATGGGAAAGGAAGAG

ACTTACAATAAGACTGAAGCCAGAGACAGCACAGAGAGAGATGGCAATGGCTGTGCTAGA

GAGAGCAAGCTTACCCAAGTGACCAACCATCATCATTGAAATAATCTGTAGAAAATACTG

TGACAAAGTCACAGTAATCATAGGAGCTGCTAGATAACCCACTCTTTTCATTTCCTGAGA

AAAGACAGTCCATGTTATGGCTGAAGCTGAAGGATTCTCCGGCTCTGAGTCCTTATCTAA

GAGACTATTTTCCATATCCTCTCCAAAGGGTCACAGAATCACTGCACCTTGTCGAAACTG

GTAGAAATAATGGCAGGGCTTTGGTGTTTTGTATGCTAAAAGGTTTAAATATTTACAAGT

GGCAGCAGCAGTAGTTCTTCCAAGGTTTAAATATTTGTTTCAATAATGACTGTCTCAGAG

TCTCTCATGGGCTTCCCCCTCTTATGTAGACGGAACAATATTTCAAGTAAATTTTAAAAT

TTAAAATCATTCCATCTAATCTAATTTAATCTAAATTTTAATTTTGATTTCTTATAATTT

TAAATCTACAATTTTTAATTTTTTATTTTTAAATTAAGACAATTTAGTCAGAACAG

>**Gmax_MSTRG.69516.1** GeneID=Gmax_MSTRG.69516;Strand=+;Pos=Chr13:31688211-31688444,

ATTTGTGTCATTGTTGCCGGTAGCATTATATTCCTGTGATTCATCTTTATCTTCCCTAAA

TTCTGTTGGAGCGTCAGTATTCATCACTTTGGGGGTACCTTTTTCCATGTCTTGAAGCAT

ATGATCCTCTGGCACAATTGTTTCGCCGAACTTGGCCTTACTATTACCATTTTCAGCTGC

TTTGGTGTTCAGTTTTTGAATGGTATCTTCTTCAGCCACAAAGGAAGTGGTAAT

>**Gmax_MSTRG.65157.6** GeneID=Gmax_MSTRG.65157;Strand=+;Pos=Chr12:38511645-38511775,38511902-38511961,38512178-38512512,

GGTTATTGGAACAAAAGGTGGTTGATGGGTAGGGGAAGGGGTTTTTGGAGGGTTTGGAGT

GTAGGGAGGTTGAGAAATAGGAGAGGGAGTGTTTGGAGGGGTTGGGACATAAGGTTGGTT

GCCAGGGGAAGGTGTTTTTGGAGGAGTGGGAACATTTGGAGGACTATAAGAAGGAGAAGG

TGTTTTTGGAGGGGTTGGAACATAAGGTGGATAAGGAATTTTTGGAGGAGTTTGAGGAGG

TGTTGGAACATAAGGTGCTTTTGGAGGAGTAATTGGAACATAGGGAGTGTTTGGAGGTGT

TGGAGGATAATAAGGAGGTGGCTCTCTCACAGCCAACACCACAATAATCAATTTCTGACC

CTTTTCACAATTACCATCTTTCCCACTAATGAAGTAAAATGGACCCGATCGATCAAACTG

AAACTCCGATTCACTATCCTCAAACTTCTTGATCGGGTTCTTCTTGTTACATTTCTCATA

ATCATCCTTGTTCACCACCAACACAGTATCCGAGCCCTTCTTGTAC

>**Gmax_MSTRG.55220.1** GeneID=Gmax_MSTRG.55220;Strand=-;Pos=Chr10:49047749-49049365,

TAAAAATACATTTTAAATAAAATACAAACTAAAATACTAATTATACATATATTAGAACTA

GGGGAGCTCAATGTATTTTATTTTTGGTACAAAAGGAATAAAATAAAAACAAGACAAAGA

ACCTAAAAAAATAACTACTATTCTCGGGTGAATGCAACACCCATAACATCTGCCATCAGT

ACAGTGGATATGTCTTCTGAAGTGGGATCATTCCAAGTTAGAAATTGTTTCTCTTGAGAA

GCACTCTTTTTGGCTAAGAAATCTGCCCATGCATTACCTTGATGTAATGTATGTCTAACA

TGAACAGTCCTACTTAAACAGAGAAGTGCTTGAATTCTTTTGATGATTACTGCAGAGGGG

TGGAACATATTGGTACTCCTGATAATCGATATGGCATAAGTAGAATCTGAAATCATTGAC

TCTTAAAACCACGACTCCAAGCCTCTTGAAGTCTCTTATGAAAAGTAAACACGTTGATAA

ATATTTCGTATCAAGATCATGTTGATAAAAAAAAAAAAAAACAATATTAACAACATATCA

TATTTAATATTTCAACATAATTTTAAATATTTAATAATTTAAAAATAAGTATATCAATAA

TCTTTACATCAATATTGATAAAGGGAGATAGATGAGAGGGAGGATATAGAAAAAGCGAGG

AGAGTGAGAGTGGGAAAGTTACCAAACCCAGCTCTGCGAGAAAAACTTAAGACACTACAA

ATTTAACGAAAAACAATAACAATGAAAAATAAACAAACAAAATCTCATAAAGCATTACTT

TCAAGTTTCAAACAGCTAAAAAGAGGTTGAGCTCCTACTTCTCATTCATGGAGAGTTGTT

CATGTCATTAGAAAATCGGCCTTTGATCATGGGTTTCTTATACGAGTCTATTGTCTGCTG

AAATTTCGCTATCAAATAACCTTTTTCTTGCCTTGATAGCCTGCATTGGTATTCAGTAAG

ACCACACAATTCAACAAAATGCCTAAAGTGGTAGCCAGGCAAAGAATTATATACTTGTCC

TTTTAAATTTGTTAAACAAGAATATTAAATTTTGGGGTTTTATGACAAAGGTTAGAGGAC

ATATATACCTGTTGTTCCCAATTTATGCAACTTGTTATGGTAGAAAATAGAATACATTGA

ACAAAAGCACCGACTTGTACACCAATCCAAAGTCCTTTTCCTCTCATTTTAGCCAAAAAT

GCCAGTGTGGCAGCAACAGGAATCCCACAAAGATAGAATGCCCCTAGATTCACATAAACC

CCTATGTGTTGCCATCCACAACCTCTAGCAACCCCTGAGCCGAAGTATTATAAAACAAAT

TGAGACAACGTTTCTCTTCTATATAAAATTTACCTAACAATAGAGAAGACAGAATTTTAC

CAGCGAGAACGCCTTGTATGCTGTCCAGTATAACAGATATGCAAATCAGAGGAGCCATGA

CAGTGACATAATCAACAACTTCCTTCTCATTGCTGAAAATATAACCAAAATCATGGCGAC

AGACAAAGAGGGTTCCGCTAACTATAGCTGTCTCCATGATTGCAAAAGACATTGCAGCCA

ACACAGCAACATGGGCAGCATGTGGATTCCCTGCTCCTAATTCATTTGAAATCCTTG

>**Gmax_MSTRG.55225.1** GeneID=Gmax_MSTRG.55225;Strand=-;Pos=Chr10:49058648-49059711,

TGAAGGAATAGCACAGCGGAAGAACTCCCCAATGCCATGGAATAGTTCCGTTGAAATTGG

TACCCAAGTCTTTTTACATTCAATAGAGAATTTCATGTATAATACAAGTAAAATCACGTT

CAGCCAGTATGAAGTACCAATAAAAAATGCTGCTCCTAAGTTACCAAATCCAAATTTAAA

AACCAGTAGCCAACAGAAAGCTACATGGAAGCAAAGAGTAATGGAGGAACTTATGACAAG

GGAACTCTGAAGGAGAAATAGGCTAACACAATAACAAACTCTATAACAGAGTAATAGGCA

GTGGAAATAAATCCTCCCAAACTTACAGAATCTGTGAGGATAAAGTATAGAGCACAAAAT

AGAAATTAAAGATAAGAAAGACACCATTTGTGTTTAAAGTGAAAAATCCCTCAATGCAAG

GGTAAAAATCACGGGCCGTCTAGACCAAATAAATAACTCCGATATAATCAATGAAGATAC

AAGAGAACCTCCAAACTTCAAAATATAGAACAGATAATGCGAAACACTTATCTCTCTCTA

GATATCTTTTTCCTGATCCAATCGAACAATTTGAAATATCACGCGCGTAGAATTTGCTAT

CCAAAAATCAACCTGATTTAACGGTGAACAAATCCGCAGTTATAATCTGAAAAACAAAAT

CACCATAATTTTCTCTCTCCTTCTTTCTTAACTGTTTTTCCTATCAAATAGTCCCTATCA

CTGTATCTCACCCTCTAATCTCACTTAAATAAGTGAGATATACAGGTCTTCTCATTTGGG

GTCAAACTTTGCATCAGAAAGTATCGAACCAAGGCCTGAAGTGTTGCATAATCAAAGAGA

GCAGGGGTCATGCACAAAGCAAATTTTCCAGCTTCTTGAGAAATCAAAGGGTCTTGGCCA

AGAAAAATGAGTATCTTCCCCAAGTATACCCACAAAGGACTCAAAGGAAGACAGGCTAAA

GTAAGAGAGACAATAGCAGTGTACATTTGAACACCAAATTTTCGATATTGATGTGCTCCG

TATGCTTGCCCACATTGAGTTTCCAGTGCACATGACATTGCAAA

>**Gmax_MSTRG.104177.5** GeneID=Gmax_MSTRG.104177;Strand=+;Pos=Chr20:36605682-36605828,36606807-36608312,36608599-36609160,

AAATACCTTGGATGAATGAGAAAGAAGCAGAACAATTATCGCCATTTCCTTATTTTACTG

CATTAGCTTAGTTACCTTAGTATAGCAATCCTAACAGTTCTTGCCTCAAATTTTTCTTTT

CAGGAATATTCGATGGATTTTGCACATAGCCATAAAGACTTGGAAGTGGAGGCAAACCTG

TTTTTCCCAGTTTGTACAACTTGTGATGAGAGATAGCATAATTGTTTGGCAGAAGGCACC

AGTCAGTATTCCAATCCAAAGGCCTTTTCCTCTTAATTGTAGCCAGAAACCCAATATGGC

AGCCATTGGAATTCCCACAACATAATAAGCTCAAAGGTTTACATATGCTCCTATGTGCTG

CCACCCACATCCTCTAGCAATACCTATAATGAAGGCATAACTCAGATTCCATTGCTTAAG

AAGAGTAATGTGCATTGAAAAATAGTTCAAACAAGAACTAACCTGAAAGGGTACCGTGTA

ACGTGTCTACTATAACAGATATACTTAAAAGAGGAACCATATCTGTGACATAATCCACCA

CATCCTGCTCATTGCTAAATACATAACCTAAAACCTGCCTGCTAGCAAAAATGATTGAGC

TCACCAGAATGGCCTCAGAAACTGCTAGAGTCATGGCAGCAGAAACAGATACTCGTGCTG

ATTGTGGACTTCCAGCTCCTAATGCATTTGAAACTCTAGTGCTGAACAACCCGCCCAAAA

GAGAAACCAATCAGAAAGAGGAAAAAATTCATTTACTATAATATCATTCTACATGAATAC

GAATATGCACAAATTGATGAGCTTAGGACTTGTGTGAATCAAAACCTTGCTGCTGAGCCA

ATTGCTTCTGGGATTGTGTAGATAGTTGTGATGATGGATAAACTGGTCAGAACATAGTTA

ACAATAACAACAAATTAGCAACACTTGCATGAATGGAAATCAAAATTTGGCTGAGTAACA

ATTTCAACACAGATTTTAAAACACAAAGCCATTCATGTCTGAAGGACTAGGATAAGTTGT

ACATACCATATGGATAAGACTGAAGTTTCAAGCTCTGGATTTGGTAGAAGACCAGAAAGC

AAGGTTAGCAGCTCAAATGACCACCACTCAAGGCTGCAACGATCAAATAAGTAAGAAACA

TACCATGGTTGAAAATTAAATGGGAAATAAAAACACAACACAAAAGATAAAATATAAGAG

GAAGAAACTCACCAAACCATTCCAGCTGAAGGAATAGCATAGGTGAAGAATTCACCAATC

CCATGGAATAGTTCCATTGAAATTGGGACTCGAGTCCTTTCACATTCAGTAGAGAATTTC

ATATATAAGCCAAGTAAAATCACATTCAGCCAGTATGAAGTACCAATTGAAAATGCTGCT

CCCAAGTTACCAAATCCAGATTTAAAGACCATTAACCAACTGAAAGCCACATGGAAGCAA

AGAGTAATGGAGGAACTTATGACAAGGGGACTGATCAAACTTTGCATCAAAAAGAATCGA

ATCAAGGCCTGAAGTGTTGCATAAGCAAAGAGAGCAGGGATCATGCACAAAGCAAATTTT

CCAGCTTCTTGTGAAATCGAAGGGTCTTGGCCAAGAAATATGAGTATCTTCTCCAAGTAG

ACCCAGAAAAGAGTCAGAGGAAGACAAGCTAAATAGGTCTCAAAGCTTAAGAAGAAAGCA

GTGAGATGGGAAAGGAAGAGACTTACAATAAGACTGAAGCCAGAGACAGCACAGAGAGAG

ATGGCAATGGCTGTGCTAGAGAGAGCAAGCTTACCCAAGTGACCAACCATCATCATTGAA

ATAATCTGTAGAAAATACTGTGACAAAGTCACAGTAATCATAGGAGCTGCTAGATAACCC

ACTCTTTTCATTTCCTGAGAAAAGACAGTCCATGTTATGGCTGAAGCTGAAGGATTCTCC

GGCTCTGAGTCCTTATCTAAGAGACTATTTTCCATATCCTCTCCAAAGGGTCACAGAATC

ACTGCACCTTGTCGAAACTGGTAGAAATAATGGCAGGGCTTTGGTGTTTTGTATGCTAAA

AGGTTTAAATATTTACAAGTGGCAGCAGCAGTAGTTCTTCCAAGGTTTAAATATTTGTTT

CAATAATGACTGTCTCAGAGTCTCTCATGGGCTTCCCCCTCTTATGTAGACGGAACAATA

TTTCAAGTAAATTTTAAAATTTAAAATCATTCCATCTAATCTAATTTAATCTAAA

>**Gmax_MSTRG.93967.1** GeneID=Gmax_MSTRG.93967;Strand=+;Pos=Chr18:23063092-23063383,

CTTTTTTCTTCAATACTAATATTATACTAAAACACTAGAAGAATTATTAAGAAAAACATA

ACATTACTCATTTATTTTTTAAGGAAAAAAATGAATGCAGGATCGTCCACCTGATCCATG

GGAAAGAGAAACAGCAAACAACTAAGTCATGGCTTGAAAAAAAATCAATAAAAGAAGGTA

CACCCTGCTTCTAGAATTTTCAAATTTTCATGTTTCTTGATCATTCTTCGCTATAGCATG

CGTTTTTGAATCTTCATGCCCACCCCACTGCTTTATTCTATCTTCCGCAAGG

>**Gmax_MSTRG.82352.1** GeneID=Gmax_MSTRG.82352;Strand=-;Pos=Chr15:51267918-51268113,51280055-51280065,

ACAACGAAATTACAACTGATCCTAAATGGTGAAAAGTGATACAAAACATTTTTTCTGCTC

AACAACGAAATTACAACTGATCCTAAATGGTGAAAAGTGATACAAAACATTTTTTCTGCT

CAACAACGAAATTTCTTTTGTTAGATGTCATCATCAAGACATTGAGCGGCCTCTGAGAAA

ACGCCAAGGTCCAGTTCCTGTTCCCAT

>**Gmax_MSTRG.46911.1** GeneID=Gmax_MSTRG.46911;Strand=-;Pos=Chr09:33370831-33371123,

CCTGACATCACATGTGAAGCACTATTGATAACCATTGATACGCCAAGAAGGTATGCTAAA

TCAGCAACAGCTCGAATCATATCCTTACTGTTGGTAAAGATCTTGGCAAACTCATCTTTA

CTCAAGAAAATGACAGTCATGAAAACAATGCCGAGGAGGAGAGACTGAAACATTGTCACG

CAGAAAGAGTATATGGCTGCTCTTGGATGCGACTTGCCAAGTATATAGGAGACACGAATA

CTTGGAGGGCATAGAACCAATAATCAGTGTAAGAATTGAAAAATATATAAGCA

>**Gmax_MSTRG.93968.1** GeneID=Gmax_MSTRG.93968;Strand=+;Pos=Chr18:23066532-23066883,

CCATGACCCCCTTGTCAAGCTTGTACCCTAAAATAAGACCCAGAGGAATACCGAAAAAGT

AGTAACAAGCAATATTCACGTAAGCAACCACAGCCTGCCACCCTGCGCCAACAGCAACAC

CTGAGAGAACGGGTTGCACGTTGTTGATGACAATGCAAAGGGCCAACATGGGCGTGAGCT

CTACCACAATCTTTCTCACTTCTGAATCATTTGAAAACAAGAAAGGGTACTGGTTCCGGA

AGATTATCAGCACCATCGAGAGCATGACACCGATCAGAGTTGAAGTAATCACAGCAACAA

GAAGTGAAAACTTTGCTGTTCTTGGGTGACATGCTCCTAGTTCATTTGACAC

>**Gmax_MSTRG.54536.1** GeneID=Gmax_MSTRG.54536;Strand=-;Pos=Chr10:46173137-46173501,46173594-46173706,

TGCTTCATAGCCAAGAAGGAACCCCAGTGGAAGCCCAAATAAGTAATAACAACCTATGTT

GATGTAGGCCACCAGGGCTTGCCACCCACCTCCAATAGCAACACCTGCAAAAAAAACCAC

TGGTTGAACACTGTTTAGAACCATTGTCACAGCAAGGAGGTATCCTAGTTTCGCAACAGC

CTTATGCAAAACCTCACTGTTTGTAAAAATGATGGCATAATAATCTCTAGTCGCCAAAAT

AATAGCCATGAAAAAGATTCCCAAGAAAAGCGACTGAAAGACTGTCACATAGACAGAGTA

CTTGGCAGCTCTTGGATGTCCAAGCCCAAGTTCATTGGAAACTCTGACACTGAAAATGAG

AAAAACACAAACATTACGTGGCTAAGAGAAAAGCCTGCATTGCAAGCAACCAATACTAAC

AATGTGAAATTGTCAACATATTTGCCAGAGTAGACAGACAATGGGATAAATAACAGCA

>**Gmax_MSTRG.55221.1** GeneID=Gmax_MSTRG.55221;Strand=-;Pos=Chr10:49053505-49053763,

AAGAAGACTGAAACCAGTGACCCCTGATAGAGAAATGGCAAGGGCGGCGCTAGAGAGATA

GAGTTCACCCAAATGACCAACTATCATAGTTGAGACAACTTGCAACAAATATTGTGAAGA

AACCACTGCCACCATAGGCCCTGCTATATGGCATACCCTTCTCATTTCTTCACTATAAAC

ACCCCATGTTACTCTTTCTTGCTCATGTTTCTTTACTAGACTTTCTTCCATGCTAGAGAG

AGAAAGAAGCAAGATCTTT

>**Gmax_MSTRG.10869.1** GeneID=Gmax_MSTRG.10869;Strand=+;Pos=Chr03:496734-496767,505757-506089,

AACACTGTTTAGTAGAATAGAGATTGCCAACAAAAACAAAGGTGACAAGTCCCCAACGGC

ATCGGCCACGTCTTTATTTGAGGTAAATATGTAAGCAAGTTTTCCCCTTAAAAATAAGAA

AAATAAGAAGAGAACAAATCCAATGGCCAAGGATGTAAGCACTGTCACAACTATAGAGAA

CTTTGCAGCTTTGGAGCTTCCTTTTCCAAGCTCGTTTGCCACTCGAACACTGCAAATGAG

AAACCATAAAGTTAGTATTCCATGGTAAATGAACAATAGATGAAAATGTGGTAGGTTTAG

ATGTGTGTCTGTGTGTGTTGGTGCTTGTTGAGGTTGGCCTTCTTGGCAAAACCGTCCACA

ACCCCTC

>**Gmax_MSTRG.54534.1** GeneID=Gmax_MSTRG.54534;Strand=-;Pos=Chr10:46170368-46170785,

GCCAATGACGGAGTTAATGAGGGAGATAGCAGAGAGCTGAATGTCGCCGAGGTGTCCAAC

GAACATGCTGGTCACGGAATTCACACCGAACTGACACCATATGTTGAAAACAATGGGCAT

CGCAATCTGCCACACTCTCTTCGTCTCAATCCAGAACACCTTCTTCACCTCCTTCAGCTC

CCTCACCGCCACGTAGTCGCCGTTCTCCGTCACCAGCGCCGCCCCCTCGCCTTTGGCCAG

CAGCAGGGGAGCGTCCATCTCTCAGTCACCCTACACGTCTACGCCTCTGATTCGACGTTA

AACTCTCACGGGCTTATATCCTGCAACTGCATATGCACATCCACCACTCCACCAGTGCAA

TTTTCTTTTCTTCAGTATTTATTATTATTATTATTCATACAACGAACGTAGAATGTAC

>**Gmax_MSTRG.549.1** GeneID=Gmax_MSTRG.549;Strand=+;Pos=Chr01:2754609-2755346,

TTTGCATCTGTAAGTGTTAAGAAAAGATAAAAAATATCGTGTAAAATTAAAATAAAAACT

ATAGAAGTATTCATGTGTATCATAGTATTTTAATATGTACTGTTAAAAAATAAAAATGTA

GAATTATAAAATGCTGCTAGCTAGACACGGATACTATTAGTATTCATGTGTATCAGGGTG

TGTATATGATGTAGCCTCTTTCATCAATCATGAACCGTAGTTATGTGATTATGGAGAGAA

GGATTTTACCATTATGCCGTAGGAGAAGCCGGCGATGAGGGAGTTTTCAACGGAGACGGC

GGCGAGGTCAATGGTGCCGACGTGGCCGGCAAAGATTTGAGTGAAGGCGCCGAGGGAGTA

CTTTGAGACGAAGGAGAAGATGGCGGGGCCGGCGAGGTACCAGAGCTTCTTTGACTCCAC

CATGAATTCCCTATAGAAGTCGCCGGCGCCGGTGATCGGAGCTATGTCAGGGGTGCCGGC

GCTAAAAACTGCGGTTGTTTCTGCTGGGTGAGGGTGGATTTGATGGGGTTCGTCGGCCCT

GGCCGTGAGAAGTGGCTGTGTGGAGGAGTTGCCATCTGAGTGTTTCATATTGCAGAGAGA

GAGAGAGTTGGAGTGAAATGACAGAGCAGAGTTTTTTGATAGGCTCAACAGCAAGTGTTA

CAGACCAGGTTAATTTATATATATGACCTGGCTTATCAACATTTTAATTTTATTTAATAG

AAAGAGATCATGTAAAAG

>**Gmax_MSTRG.70932.1** GeneID=Gmax_MSTRG.70932;Strand=-;Pos=Chr13:37696399-37696766,

CATAACTGCCACGCCAGTGACACTGAACCATGAATTTGCAAGAGTAGCACCAGCAAGCTG

AAGCTCACCAAGGTGACCAACAAGCATGACAGAAACCAAAATGATCAAGTGATAGAACAA

GTTGGTAAGAATCATTGGCAGTGAAAACAAGAGTTGGTGCTTGGCCTCTTCCATGTCCAA

CACTTTGTTCCACCATCCCTCAGCTTGGGGGGATCTTGTGTTTTGATCTCTTCCATTATT

ATCAGATGCTCTCAATAGGGGTACTCCTGTTCCTTCTGAACTAGAACTTGCCACCATCTT

CTCTATCTCCTCTTTTATCTGCTCCACGTTTTCCATATATATGTGTAAGGAAGCACTTTG

AATTTTTG

>**Gmax_MSTRG.62820.1** GeneID=Gmax_MSTRG.62820;Strand=-;Pos=Chr12:8609947-8610587,8611168-8611712,

AGAGAATCCAGAGCCAACTCAGGGTGAGGAAGCAACCCTGCAAGCAGAACCAAAATTTGG

AAGTACCAGGTCTCTAGGCAGAGCATCACCGCCGAAGCAGCAGACAGCTTAAAAAACTCT

GGCAAGCCCGAAAACGCTTGAAAACTGAACCCTCTCCAAGTGTGCTTACACTTTTCACTC

TTGACAATGTACACAAACTGTGCAATCACAATGATCCACCACGAAACACTCAACACCAGC

GACGCACCCAACAGACCAAGCCCCACCTCGTACACAACAAAATAACTCAACACAAGATGG

ACCAACAACGTTGCTGTTGAAATGTATGCGCTTGGAGCCACTATGCTCTGGGCTTGGAGG

AATTTTTGAATGGGGAAGTTTACAGCGTAAGCGAAGATTTGAGGGATTAGGCCGTAGACG

AAAAGTGCTGCTGCGGATGCGATTCTTGGGGATTCTCCTAGGAAGATTAGAATGGGTTCG

GAGAAGATGTATATGATGGTTAGAATAATGCCTGCTAGCGTCAGAAGCACCGTTGATCTT

TGCAGCAATGAGATGGAGAGAAACAGGGTGAGTAGTACTGTACCATGAGGCCATAAGCGA

AGACTTGGATGCCGGTGTTTCCAAGAGAAGCAGCAGCAAGTTCAAGGTTACCGAGGTGGC

CTGAAAAGATTTGTGTGGACATGGACATGACATAGTTGATAAGGTAGACGATAACAGCAG

GAGCAGCAAGGTGAAACAAGAGCTTCAACTCCACCCATGTTGCCGGTCCAAGACGCTTCG

CGAAAGGGACGCTAGTGTTTGAGAGTATTCGCTCAAGTTCACCATCTGATCCATGCTTTG

AGCTGAATGATTGAGTACAAGAAGGTGGCTCGGGTGAAGGTTCGTTTGAGACCAGCAAAG

GTTCATCAATGTCCTTGTGAACTGATGTAGAATCCATTTGGGAACTAACTTGTTTATGTT

TGGAAAATGTTGTGGACTAAGCCTTTTTTCCCTCTGCAGCAGAGGAGGTTTTGAGGCTCA

ACTACAAAAGGATTGGATGGGATTATATATTGTCCACTCTTAAATACGTTTTTAATTCTT

TCATTTCATTTGTTTTTGTTTCTTGTCTTGGTTATAATTTTTTTTTAAATAGTCATTCTC

ATTAATATTATTTAGTGATATGTAACCTGTATCAAACCATGTCTAG

>**Gmax_MSTRG.46905.1** GeneID=Gmax_MSTRG.46905;Strand=-;Pos=Chr09:33355690-33356316,

CAAATAGAATAGGAGCCAACAGCAATCACAGGATTGTCTAGAAGACCAGCAAGTAGCATA

ATGCATGTCATATACCATTGCTCTAAGCAACCCATTACAGATGATTGAAGACTTAACTTA

GCAAAGGCCAACAAATCCCTAAATGCCATCCAAGAAAATCCACTCCATTCTTCTTTGCAC

CAACTAATGGTATAGACAACCAAAGCCCCAGCATACAACCACCCTATAATATTACTCACC

ATGGCCAAGCCGGTTATACCCCAACCAAATATATTTATGAAGATGTAAAGCAGTCCATTT

TGTATGAGCAAAACAACAAATGCTATGCACATAATAACTTTAACCTTGCTCTGGGCCTGA

AGAAATCTAAGGGTGGGAAAAACTATAGCAAATGAAAACATGTGGGGAATTACTTGAATA

GAATATCTGCCAGCAAGATTAGCTATTCCTTCATCTTGGCCAAGCAACTTTAAGATTGGA

GTGGCATATATATAAATAGGCAAGAGGATTATACAAGTGGCAGTAAGTATAATCCATGAC

CTTTGAACATAAATGCAAGTAGATTGAATTTGTCCTGCTCCAAAAGCTTGGCCACAAAGT

GTGGCCAGTGCAGATGACATACCAAAC

>**Gmax_MSTRG.46371.1** GeneID=Gmax_MSTRG.46371;Strand=-;Pos=Chr09:18849747-18850399,

GGTTTTAGACCCATTGCAGCTAATAGAGGTTTTGCAGCAAATATAAGGGTTGTTGCTTGA

AGGAGACCAAGGATTGTGCCAAAAAGTAATGCTGTGGATGCTGAAGCAACGTGTCGCTTC

TTCCTTCCATCTTTTGGCACACTCTTGTTACTGCTACTACTACTAGTATCAGAGGTAGAC

TTGCATATACTTGTATTGCAATCTGATAAAATGTGAGAACACAAAATTTCAATGCATTAA

TTTCCCTCCATTCATATATCTAAGTTTATCATAATGAATGTCCTACTCATTGGTTTGTGC

TTATGCATTTGAACCAAATTAATTTACATCAACATGGACATTTATACACAGCAGTAGAAA

AGGAAAAACTAGAAAGGGATAGAAAGTACCATTCATTTCCACATTCTCAGTCTCATTGTT

CTCTTTGGATGCAACTTTCTCTATGTCTTGAAGGGAATGATCATCGAGCATTACTTGTTC

TTTGGACTTAGCCTTATCACTTAACTTCTTTTCTGTATTGATTTTTTCAATGGTATTTTC

CTCAGCTACGAAGGAAGTTGTGATGCTAACCAAAGGGAAGATGGTAATCCTTGAAGCTTG

GTTGAACAAAGCAATGGACACTCCTGCAGCAGCAAGCTCCACGGGCCCTAAAC

>**Gmax_MSTRG.47701.1** GeneID=Gmax_MSTRG.47701;Strand=+;Pos=Chr09:40368419-40368631,

AGCTTTAATTTTAACCATCTACTTGAATTATATGTTAAGATTCGTTCTTTTGAAACTCTC

ATATGTGTAATGTGTGCGTGTGTGTTGAGAAAGAGAGAGATGACATTTTGTATGGTGATT

TAAATTAATGCCTCATGTCTTTTAATTCTGTAAAGATGACATCAATTCATTTCTGTGATG

GTAAATGGATCTCTCTGTGGCAACTCCTGAGGG

>**Gmax_MSTRG.65157.7** GeneID=Gmax_MSTRG.65157;Strand=+;Pos=Chr12:38511645-38511775,38511902-38511961,38512088-38512282,38566213-38566222,

GGTTATTGGAACAAAAGGTGGTTGATGGGTAGGGGAAGGGGTTTTTGGAGGGTTTGGAGT

GTAGGGAGGTTGAGAAATAGGAGAGGGAGTGTTTGGAGGGGTTGGGACATAAGGTTGGTT

GCCAGGGGAAGGTGTTTTTGGAGGAGTGGGAACATTTGGAGGACTATAAGAAGGAGAAGG

TGTTTTTGGAGGAGTTGGAATAAATGGAGGATGATTAGTAGGTGAAGGTGCTTTAGGAGG

AACATAGGGAGGACTGTGAATAGGAGAAGGTGTTTTTGGAGGGGTTGGAACATAAGGTGG

ATAAGGAATTTTTGGAGGAGTTTGAGGAGGTGTTGGAACATAAGGTGCTTTTGGAGGAGT

AATTGGAACATAGGGAGTGTTTGGAGCAGGTGTTTT

>**Gmax_MSTRG.8277.1** GeneID=Gmax_MSTRG.8277;Strand=-;Pos=Chr02:31120425-31120669,

GTAAAAGAATCACTTTTTTCAAATAAAAAACTATCATTTTAATAGAGCAACCCTTACCCA

CAAAGGGAGCAATCACTTGCAGTAGTACTAGTATTCCATGATGGGAGGATTCTCTTCAAT

GTAGCTTGTGTTGTGCACATGAGAATCAGAATACCAAAACTCTCAGTCGTTAGTAATAGG

CTGGTACTACTAAAGCCCAACATTGTTTACCTTAAGGAAGCTCCAAGGCCCTGATCCAGT

ACCAA

>**Gmax_MSTRG.10870.1** GeneID=Gmax_MSTRG.10870;Strand=+;Pos=Chr03:497768-497800,506951-507465,

GACAACAGGCCAAAGATCTTTGAATGCCAAAAACAAAAATGAGAAACCTTTCCATGTATC

GGAGCACCAACCACATGTTATAAATATTAGTTGACCAATATTAGGAATCCAAAATGCCAA

ATTTGTTGAAGTCATTGCACCAGGAATCTCGAGCTTGAATTGAATTGTCAATAGCCAAGA

GAGGAACAAATGAATAACTATTGAGAATGCTGCCAAGAAAGAAATGATGGTATTTTTGCT

TTGAGATTGCAGGAAATTCTGGCAAGTGAAAGAGGCAATAAAAGCAAATATCATAGGAAT

TGACCAAAGAGAAATATTTCCTGCCACTTCTGCTATATTCTCATCTTGGCCTAAGAGCAT

CAAAATCGGCCTCGTGAAGATGAACACCGGAAGAAGAAAGAGTGAAGTTATGGACATAAC

TATCCATGATCTTTGAAGATGCACTCCCATCATGACATATTCTTTTGCACCGTATGCTTG

TCCGCAAAGTGTTGACAACGCACTCGCCATTCCTAACTAGAGTTGTTACAACAGACACAA

ATAACTTG

>**Gmax_MSTRG.55223.1** GeneID=Gmax_MSTRG.55223;Strand=-;Pos=Chr10:49056210-49056895,

CCCAATTTATGCAACCTGTTATGATAGAAAGTAGAATACATTGAACAAAAGCACCACTTT

GTATGCCAATCCATAGTCCTTTTCCTCCCAATCGCACCAAAAATGCTAATAAGGCAGCCA

TTGGAATCCCACAAAGGTAGAAGGCCCCTAGATTGACATAAACCCCTAAATGTTGCCATC

CACAACCTCTAGCAATCCCTGAACCAAATAATTAAACAAATTGAGAGAAAGCTTTTCTTC

TATATAAAATTCACTTAAGGATGGCCATTGAGAGAAAGAATACCTGTTAGAACACCTTGT

ATGCTGTCTAGTATAACAGATATACATACCAGAGGAGCCATGGCAGTGACATAATCAACA

ACTTCCTTCTCATTGCTGAAAATATAACCAAAAACATTGCGGCAGGCGAAGAGTGTTGCA

CTTACTATACTTGTCTCAATTACTGCAAGAGACATTGCTGCTAACACGGCAACACGTGCA

GCATGTGAATTCCCAGCTCCTAATTCATTTGAAACCCTTGTGCTATAATCAGAGAGGAGA

AAAGATAATTAGTTTAGATGCATGCTCAAGGCAGCTGGGTACTAGTGTAACATTTAAACT

ATGAAGAATGAGTGTGTCTATGATGCAACCTTGCTGCTGCACCAATTCCAAAGGGTATCG

TATAGAGAGTTGCAATGGTGTTGAGG

>**Gmax_MSTRG.46912.1** GeneID=Gmax_MSTRG.46912;Strand=-;Pos=Chr09:33372465-33372784,

GGCAAAGATGAACGTTTATTGATACGTGACTCCATAAACTCTACATTTTTTTTTTTTGGT

GGGTCCATTAACTCTACATAGTGACTGTGTACAAGTCGTCTTCTACAGTTCTACTAGATC

AATCAACCAAAGTAAAGGGACATATGTAAACCATAAATGGATTTAACTGCACACTCTTTT

ACCATATTCTTTTAAACATACTCTATGTTATTATCTTGAAGTTTAGAAGAAAATATAAAA
TTATGTAAGATTCATTTCTTATTAAATGAATTTTTTACATAATTTTATTAATTTCAGTAA
AATGAAACGTTGCTATTTCT

>**Gmax_MSTRG.6169.1** GeneID=Gmax_MSTRG.6169;Strand=+;Pos=Chr02:6597684-6598881,
GTCAAACAAGTAGAACCAACTCTTAATAACAGGTAAACTTCAATTTAGATCAAAGTCACG
ATCTAAATTGATCATGAGTCAGCAGTGTGGACTGTGGAGAATCAAATTGTTGGCGTTTAG
CACAGGTCGAGTTCTGACGCGAGTTATACACGGCTTGAATAATTATCAAGTGCTGCATTG
TGTGCAACAGCTTTCAGGCAAAGCTGCTTTCAGTTTAATCTCTGTCTCTTACGTCTCAGC
CTTACATCTAAAATATTATCAAATGTCAGATGCCAGATTCTCACATCCAGTCACATCTCC
ATGCACTTGTCACTCACAACACAGCAAAAGCTACTTTTTCATCACAATACCCAAAACAAA
ACTTGCCCATTAGGAACACAAGAGAAGGTGAGAGAAAAGTGCTAAAATATGTAAACAATT
GGTATCGAAAATGAGATTTGGATTGGAATGGTTTCCCAAGCAAATTAAATTAAATTTTAA
AAAAATAAGATAAGCCCTCCTCAAAATTATCATAAAGCAACCACTCACAAGCCTCCAACC
CCATTGATCCTACCCACCAAACAAAATCCCAATGTCATAAATCATAATTGGTGTCATTTC
ATTTCTCTAGTGTGGTGTCTTTTTTCTTAGCAAATGTCATCTTTGTTGTTCCCATTTCCA
TTCACCAACAACCCTTTTCTTTCTTCATCTCTCTCATTTTCCTTATTCCTAAGCCCATTG
CAACTACCCATTTCAATCCTTGTGAGCTTCTCAGCCTTCAAAGCCTCAGCTTCCCAATCT
GTCCTCACCAACACAACATAGAGGATTGACACAGCACATGCAACCTGGGCCGACAGAAGC
CCAAACCAAAGCCCACTGAACCCAACCTTGAACCAAAATGCCAGGCCCACTGCCACCGGG
GTGCCCACAAAGTAGAAAGAGCCCAGGTTTATGTGAGCCCCAATACCAGGCCGGGCCATG
CCACGAAGGATCCCACAGCCCGTGGTTTGTGGGCAGTTTCCAAGCTCACACAAGCCCATA
ATTGGCATAACTGATGCAACCAACGCTTTGACGGGCTCATCATTGGTGAAAAGCCCAGCC
CATCTTTGACCCAGAATCACAGTCCATGTCACATTGATGAAGCCTATCACAAAAGCACAT
CCTAAGGCAACCACTGCTGCTAGCTTTGCCTTGTATGGTTTTCCAGCTCCAAGCTCAT

>**Gmax_MSTRG.21809.1** GeneID=Gmax_MSTRG.21809;Strand=+;Pos=Chr05:4903580-4905472,
CGAACTAAATAGTTTTTCCTTGAGTGCATTCTGATCATTCAATACAAATATTAGACCAAC
GGGTAGTTCTACTTCTACCTATTGATTTAAAATGCATAATCTTGTAATTTTCAAATCCGA
GATCGCAAACACTCAGAATGGTATTGAGAAAGGACTCAGCAAAATGAACATTGGAGTATG
ATCACCGGTTTTGACTCTTGAGTGTGTCTACGACTTCTATACGTTTCTGATTCTTCCTCA

CCAAGTGTTATATACATGAATATGGGGTGATAGAGGTTTCTTTGCACACTTGATCACCTT

CAGGGTTTTCAGTAAGTTCATTTACCTCATTTCTTGAAACTTGTTCATGTACCTGTGCGT

TACATACAAACGTTTGTGCCTTGGTAGCTTCTTCCACCCAATTTATTCTCACAATAAATG

TTAACAACAGAACCAAGCAAGCAGCAATACCAATCAGGAGTCCTATAAGGAGTCCACCAA

GTCCAAACCGAAGCTTGAAGGCAAAAACCACACCCAATGGTAGTGTCAGGAAATAGAACC

CACCAAGACTAGCATACATGCTTAGCCAGGGTCGTCCAGTTCCTCGTACTATGCCTCCAC

AAACCGTCACAGGAAAATTAAACACTTCGACCAGAGCCATCAGCAACATTGCCTTCTCCA

CACCCTTTACAACCCCTTTTTGATGACTAAACAAATTCCCCCAAACCCCTCTAGCAGCCA

CCATGATGGAACCTCCAATGCAACCCGATATAACACTCACTGCCAAAGACACACGTGCTG

ATTTATAAGCTTGACCAGCACGGTTAGCACCAAGTTCATTGGACACACGAGTGGAAACAC

TAGTGGCTAGAGAAAGCATCACTGCATAAAGCAAATAGTCAAAGTTTAACACTATGGCCA

GAACCCCCACTGCTTGTTTTGCATTTGCCAAGTGGCCAGTGAGGAAAAGTAGAATCTCAT

AACACCACCACTCAAGGCACGTGTTGAGGCAGCATGATCCACTAAGCTTGATTAGCCTAC

TCCAATCCATCATGTTCTGATCCCACCACCCTCCTTCCTTCCACAACATGCTCCCATTTC

TTCTCTCAAGAACCACAACATAAATTGCAAGCATAACCATAACAATGAGGTCGGTTACCC

AAACCGCGATTGAAACTCCTCGGAGTCCCATGGTTTTGGAGAGTAGTATGTTAACGGGTA

TGTGAAAGGCTAGTGCCACAGCAGAACTAAACATGGTGGGAAGAGTCATGCACTGAGAGC

TGAGGTAGGCTTTTAAGGGACAAAGGAGTGCCTTGATAAACAAGTCAGGTATGAGATAGG

AAACATAGGTTTTGGCCACTATTGAGATCTCTTGTTGTTGGCCAAAAAGAATCAAAATCT

TGTCAACGTTAAGCCACAAGAAAGATAAGGGAAGTGTTACCAAAAGCAACAAGAGGGTTG

TCATGAGAAGGGTCTTGTGAAGGAGCCTCACGTTCTTGGCTCCATGTGCTTGTCCACAAA

TGGGTTCCATGGCGCCGCTTAGACCGTTCAAGACAGCGAAGCCGGAGACATTAGCAAAAC

TGAACCCGAGTGCGCCACCGGCTAAGTTCAGCTCCCCAAGGTGGCCGAGAAAAGCTGTTG

TTATGGCTAACTTGGCGAACCAAGCCAAGTTCATGGCCACAAGTGGAAGGGCTATCCCTC

GTTGGACTCTTAGCTCTTCTACCACCATCTTCACAAAATTGGCATTCAAAGGGCATTTTT

TAGTGTGCGATGAAGGAGTTGAGGGGTGTCGGTTGGCCCTGTCGTTTGTGCTTAAGTTCT

CTCCTTCTTTAGATGTTGCTGACATTTTTGCTGAGATGAATCAGATAATGTTTTGGTGAG

ATTGTTGCCAAGTTTCACTAGCGTACCTGTTTC

>**Gmax_MSTRG.157.1** GeneID=Gmax_MSTRG.157;Strand=+;Pos=Chr01:707531-709259,

CGTCAATCAACAATCATCATAATTTTTTTTTTTATTTCATCAAGAGTAGTTGTTGTTGTC

CAAAGTGGCAATCAACGGTTGTTTGTGGCCGTCGGATCCTTCATCAAGGGCCGTAAGTAA

CTGGGCCCGACAGGCCTGGTACTCCCAATCGGTGGTCCCAATCATATACAACATGAGGCC

CGCACAACAAACCTGGGCCGACAATAGGCCCAGCCAGAGCCCACAGAACCCAACCTCGAG

CCAGAACGCAAGCCCAACTGCCACGGGCATCCCCACCAGATAAAACGCTCCCAGGTTCAC

ATTCGCCGCCACGTTCGGCCTCGCCGTCCCCCTCACCACCCCGCACCCCACCGTCTGCGG

GCAGTTTCCCAGCTCGCACAGCCCCAGAATCGGTAGCGCCGCCCCCGTCAGCCGCAGAAT

CCCCTCGTCTCCGGTGAACATCCTCCCCCACCTCCTCCGCATCGCCGTCGCAAAAACCAC

CGCGGAGAACCCCATTACGGCGGCAAAGAACACCGCCACCACCGCCGACATCCTGGCGCG

TCGTCCCCTGTTGGCGCCAAGCTCGTTTCCCACGCGCGTAGACACCGCAAGCCCCAGCGA

GGAGGGAAAAACGTAGATCAAGGACGTAGTCTGGATTAAAATCCCCATAGCCGCCACGCT

GGCAGTGGGGTCCACCAACAACCCACACAACAAAATCATGATCTCATACCACCACCACTC

CAAACAAACGGAGACACAGCTCGGCGCCGCGAGCCGAATTAGCGGCTCCCAGCAGGTTAA

ACACTCCCGGCTCGGCGCGGTCCACGTGGCCAGGTGGACCCCACTTATCCACAAATAGAG

AACCAGCAAACATAGAATGGAAAAGCTGGAGGCGGCGGCGGCGGCGGCGACGCCACCCAG

GCCGCGCTCGACGAGAGCCAGGTTAAAGGCCACGTGGAGGAGGGTGCCGGCGAGGGACGC

TAAGGTGACAGGGTGGGTGACGTTCTGGGCGCGAAGGTAGACTCTGATTGGGTGGAGGAA

AGAGTTGATAAGGAGGTCGGGGAGGAGGAAGAGCAAGTAGGTTTGTGCCATTTGGGTGAT

GTGTGTGTGCTGGTTGAGTAAGATGAAGATNNNNNNNNNNNNNNNNNNNNNNNNNNNNNN

NNNNNNNNNNNNNNNNNNNNNNNNNNNNNNNNNNNNNNNNNNNNNNNNNNNNNNNNNNNN

NNNNNNNNNNAGACCATGGAACGCGCGTAGAAAATTAGACCAGTGAGTGCTGTCGGGAAT

GCCACCTCCCACAGGGATTTCGCTTCTCCTATCACATCCCACATTATTGTCCTAGTCGAA

CCTCGTCTTGTTAATAACCCACCTAATTCTGTCTGCTTTTCCGTATCATGCATCTGCTCT

GATTTTGATTCGCACATGTTCTTGTTATATCAGTACGTCTTCGTTGATTAGGAAATCAAG

TTAATCTAGGAGGTAAACACCCAGAAATTTTTATCACGCAAAGTAACCTTACACCATAGC

TTAAATCCAAAACCAAGGTGGGAACTCAAAGTAGGGTGTGATGGATTTACTATATGTGTG

TTTATGAGAATGATACACAAACACACACTAGCTAGTGTGTTGTGATGAATGAAATTAAGA

GGAGGGGGTGGGGACTCTGTTTTTGTTTGATGGGTGGAAAGCGAACAAGGGAGGAAATAG

GTGGAGGGAAGGAGCGGAGTGATAAATTAAGAAACTAGGTAATTATTAC

>**Gmax_MSTRG.70936.1** GeneID=Gmax_MSTRG.70936;Strand=-;Pos=Chr13:37708922-37709311,

CCTGATAAGACACCTTGGACAGAATCTAGTAATATGGAAATGGAAAGAAAGGGTGTCAGT

GAAGCCAACTCCTCTTTAATTTTAGAACTATCACTGAACATCTGAATCCATATATTGTGA

CCAAATGCAAGTGCCAAAACAAAACATAAACCAAGGAGGAGAGAGAGCTTGAGACTGACA

CCCATTGCATGCTTAGCTCGATCCAGGTGGCCTGATCCCAATTCATTGGAGACCCTTGTA

CTAATATCATACTCAATTTTTTTAGCTATTCAAAAATTGTATAATGTGTGCCGATTCAGA

AGATACACAACAATGCAAACAAAAGGAGAACATTCTTAACTTACTAACCTTGCAGCTGCA

CTGAGACCATAAGTGATCAGATAGGCAATT

>**Gmax_MSTRG.99026.1** GeneID=Gmax_MSTRG.99026;Strand=-;Pos=Chr19:37882699-37883154,

GCTAAAACAGAGAAACCTTTCCATGTTTCAGGGCACCAACCACATGTAATAAATATTAGT

TGACCAATATTCGGAATCCAGTATGCCAAAATTGTTGAAATCATTGCCCCAGGAATCCCA

TACTTGAATTGCATTGTGAATAGCCAAGAGAGGGACACATGAATGATTATTGATAAAGCT

GCCAAAAATGAAATAATGACATTCTTGCTTTGAGATTGAAGGAATGTCTGGCAGTTGTTT

GAGACAATATAAGCAAATAAGATAGGAATTGACCAAAGAGAAATGGTTCCTGCCACTTGT

CCTATGCTCTCATCTTGGCCTAAGATAGTCAAAATTGGGCTTGTGAAGATCAACAGGGGA

AGAAGACAGATTGCAGTTAAGAATAAAACTATCCATGATCTTTGAAGATACACTCCCATC

ATGTCATATTCTTTTGCACCGTATGCTTGTCCACAA

>**Gmax_MSTRG.5524.1** GeneID=Gmax_MSTRG.5524;Strand=-;Pos=Chr02:3553029-3553331,3625710-3626398,

GTAAGTTCTTTCTTAACATTAAAAATTACCCACTTATTTGAAAACTCCAATTTTATAGAC

TTCAACATGAGTAAAGTGCCATCAAACATGGGCAGGTGGTATTCTTCAAAGAAAATTCTT

CAAAAGGTTACAGTTGAAGGTATAAAATGAATAATCTATACAGTCACTGGCAGTATGACA

TTACATCTGACGCTTATCAGCATATTGACTATGAATACCTCCAAACTACCTAGTTGCATG

CTTTTGCATCTGCCTTAGAACCTGTAGGGTGAAATCTTTCTGAAGCCGCCATGCATTTTA

AACCAAAACTTCTGCAGCTTCCTTTTCTACTGAAAACTTCACCAACTTTCTTGTATAATG

CTATAAATACATTGGTCAGCAACAAGAAAAAAAAGTTGCTGCCTCACTGTACTGCCTTTA

AAAAGCCAGAACAAAAATAGATTATCTGATGTGTAAACACAAATCATGCCATGGCATCAG

CCTGTTTTCTGGTTAACCGTGCATACAAACCATCTTTTAAGAGAAGTTCCCTGTGACTTC

CCATCTGCTTGAACAGAAGTTAATGAAAATTATGTTTCCGATTAGGGCAAAGAAAGGATA

AGCATAGCTTCTCATTCAAACACATAAAATCCAAACAGTTTCAGTAATGCTTGGGATGTT

AAAGAAGCCTTATTCTACTTGTAAGTTCAAGATGATATAGAAGAAAGTGTAAGGTTTAAG

AATAATCTCCAATGAAGGACAAATAGTAGAAACGGAGGTTATGTTTTTTTCTCTCCGAG

GGATGGATGGATGAAGAACTTCCAAAAGCGCAAGAGTATAATGCAGCTTTTACAATGATT

TTCTTACAACCTTGAATAACCTCTATCGTCTTCGTATTGCTCCATGAATTCATCATTGAA

CTTTTTGAAACTATCCATAATATCAGGCATGTCTTCCTCAGCTGGTAAAATAGTTGTCCT

CAAATGGAACACCCTGTAAGCATAAGTTTGAC

>**Gmax_MSTRG.104177.2** GeneID=Gmax_MSTRG.104177;Strand=+;Pos=Chr20:36602365-36602429,36607743-36609133,

GTACATACCATATGGATAAGACTGAAGTTTCAAGCTCTGGATTTGGTAGAAGACCAGAAA

GCAAGGTTAGCAGCTCAAATGACCACCACTCAAGGCTGCAACGATCAAATAAGTAAGAAA

CATACCATGGTTGAAAATTAAATGGGAAATAAAAACACAACACAAAAGATAAAATATAAG

AGGAAGAAACTCACCAAACCATTCCAGCTGAAGGAATAGCATAGGTGAAGAATTCACCAA

TCCCATGGAATAGTTCCATTGAAATTGGGACTCGAGTCCTTTCACATTCAGTAGAGAATT

TCATATATAAGCCAAGTAAAATCACATTCAGCCAGTATGAAGTACCAATTGAAAATGCTG

CTCCCAAGTTACCAAATCCAGATTTAAAGACCATTAACCAACTGAAAGCCACATGGAAGC

AAAGAGTAATGGAGGAACTTATGACAAGGGGACTGATCAAACTTTGCATCAAAAAGAATC

GAATCAAGGCCTGAAGTGTTGCATAAGCAAAGAGAGCAGGGATCATGCACAAAGCAAATT

TTCCAGCTTCTTGTGAAATCGAAGGGTCTTGGCCAAGAAATATGAGTATCTTCTCCAAGT

AGACCCAGAAAAGAGTCAGAGGAAGACAAGCTAAAGTAAGAGAGACAATAGCAGTGTATA

TTTGAACACCAAATTTTCGATATTGCTGTGCTCCATATGCTTGCCCACATTGAGTTTCCA

GTGCACATGACATTCCAAACTGTTTGACATGTACAAAAAAGACAAAGTGCAATTAATTCT

TATAATTTTTTTAGAGACCATAGGTATCTTCACATAAGAGAATCCTACTCGAACTTAAGC

CATTTTGTGTTTTAATTCATCTTCATCTCTTTTGTTTTTAATAATTTTCCATCCTTTGAT

AATTTGGGTTCAATGCAGTAGTAGGTCTCAAAGCTTAAGAAGAAAGCAGTGAGATGGGAA

AGGAAGAGACTTACAATAAGACTGAAGCCAGAGACAGCACAGAGAGAGATGGCAATGGCT

GTGCTAGAGAGAGCAAGCTTACCCAAGTGACCAACCATCATCATTGAAATAATCTGTAGA

AAATACTGTGACAAAGTCACAGTAATCATAGGAGCTGCTAGATAACCCACTCTTTTCATT

TCCTGAGAAAAGACAGTCCATGTTATGGCTGAAGCTGAAGGATTCTCCGGCTCTGAGTCC

TTATCTAAGAGACTATTTTCCATATCCTCTCCAAAGGGTCACAGAATCACTGCACCTTGT

CGAAACTGGTAGAAATAATGGCAGGGCTTTGGTGTTTTGTATGCTAAAAGGTTTAAATAT

TTACAAGTGGCAGCAGCAGTAGTTCTTCCAAGGTTTAAATATTTGTTTCAATAATGACTG

TCTCAGAGTCTCTCATGGGCTTCCCCCTCTTATGTAGACGGAACAATATTTCAAGTAAAT

TTTAAAATTTAAAATC

>**Gmax_MSTRG.99025.1** GeneID=Gmax_MSTRG.99025;Strand=-;Pos=Chr19:37876995-37877310,37879224-37879843,

ATAGAGTAAATAATAGGTCGTAAATGCTCCTAACTATAAATAGCGCTAGGTTTTTCAGAT

TGATTCCTCAGCCTCCTCTGTCTCTCATTTTCGTTTTTCCCCTTCTCCTCTCAAAACCCT

TTCTTTTTCCCGCCGCCCACAAAACCTGTCTCAGAAAAATGATGATCTCGGACTCATTCA

CCGTTGGATTGTCGTGAAATTTGAGCACCACGTTCGCAACCCAATTTCGAGAATTCTCAC

CGTTGGGAATTGAAAAATCATGTCTGGGCTTAGAGGAAAACCCTTCGCATTGTAGCTTTT

TATTTTTCTGCAGAAACCCAAAACTGTCTCGGTAAAACTACGATCCCGGTTTCGTTAACC

GTTGGATTTTCATGAAATTTGGATATGTTGCTCGAAATTAAATTTCTCACACTCTCACCG

TTGGGATTTGCGAGATAATATTCTTGGTGGGAGAAAAAGGAATCGCATGAAGACAGTACA

AGCGGAGGCTTCAATCTCTTCTCCGTCTCTCTGACGTTCGAGAATTCTATCGGAGCAGTC

GGAGGAAAAACTTGAGGAATCTCAGGAAACCGCTATAGATGTCTCTATCGTTGTCGGAAG

ACACGTGAGTCCGCTTAGAGACGACTAGGTGAATTGATTTAAGGAACCTTTTGCTGAAGG

ACGTCGAGACACAACGCTCTGATAGGATGTGGCATTGGGGTATAGGGTTTTATATTAATT

GTATGAAGTCTTAAACGGTCTTGCTTAAACCGAGATAACTTTATTAGAAATTTGGACAAG

TTTGAATATGATGTAGAAGAAAGTGAATGTGAGCCTTTTATCCTTTTGAAAAACTTGTAT

TTACAAATGTAAAAAAAAAAATACTATTAATTAATATTTGGATTTTTATTCCTTTATCAG

TATATATGTGAGAGATAGATGGTGTCACAGTAAGAG

>**Gmax_MSTRG.34859.1** GeneID=Gmax_MSTRG.34859;Strand=-;Pos=Chr07:19191538-19191837,

CTGAAATGACGAAGCAGCGAAGAAGAGGAATTATATGGTGGTTTAGGGGAAGAGGGTTGG

AGGGACGGAGTTTCAACAGAATCATTATTGGCATTGTTGTTTTGCGAAGAAGCTTTGGGT

GGAACTCGAACTCGAGCACGAAAACGAGCGAAGCAATGAGGCTTTGTGAGAGGTTTCAGC

GTTGGGGATTTCAGAGAGTGTGTGCAGAGAAAAGAGGTAGTAGAGAGAGAAGACGCCATT

CTCAATTTCGAGCTTCAGAATCAGATATGAATCTTGAGAGTGGAATTTATGGAAATGCCA

>**Gmax_MSTRG.5537.2** GeneID=Gmax_MSTRG.5537;Strand=+;Pos=Chr02:3723902-3723911,3724148-3724578,3724648-3724859,

AATGATCAAGGGCGGAAAAGCCTCCACGTGGACGAATCATCAAAGACAATGCTTCTCTCG

AAAGTTTTTAAGCCAAGACCATGGCTACGAAGATTCCAGAGAGTGCGGAGCCAGCAATGA

AGGCCTTGCTGGCGGAGAATGCGGCGGAGGGTGGCGGTGGTGGCTCGGCGCCACCTGCGG

GTCCGGGAGCGGGTCCGGCTGTGCCTGGTGCTGGTGGAGAACTAGCGGAAGGGACTGGTG

AGGTTGCAGGAGAAGAAGCAGGTGTAGGTGCAGCTGTTGGTGTTGGCGTTGGTGTTGTTG

CTGGTGGTGATGTTGCTGGTGGAGGAGTTGCTGCTGCTGGTGGTGGTGTTGCTGGTGGTG

GTGTTGCTGGCGGGGTGGGTGCTGGTGCAGGTGCTGATCTTGGTGGTGGTGGTGATGGGG

TTGTAGTGGGTGGTTGTGTGGCACCAGAACCCATCTAGCTATAGAGAAAATGAGAGACCA

AAGAAAATGTTGTAGTGCTTGTGTGTGTAAGAGAAGAGAGAGAGAGAGAGTTTGAGCAGT

GGAAAGATGATAAGGGAACAGAATATATAAAAAGAGAGGTAGAGTATGTAAGTGGGGGGC

TTCAAAGGCGTTAAAGGACAGCGCTGATTAATGCATCAATAATGCTACAAGAT

>**Gmax_MSTRG.20763.1** GeneID=Gmax_MSTRG.20763;Strand=-;Pos=Chr05:130422-130681,

GGTAGCAATAGCAACTCCAGAGAAGGAGACCAGTATCCCAAGATGTCCCACCATCATCAA

AGACACAACTTGTAGAAGGTACTGCGACACCGTGACAGCCACCATTGGAGCCGCCATGGA

GCTTACCCTCTTGAACTCTTGGCAAAATGTACTCTCCACCCCATTATTATTCTCTTGCTC

GCCACTCTTTCTCAGCACTAGCAGTGGTGCTGCCAACTCTCTGGAGCTTTCCATATTGTC

AACACTGGTCAATGTTCATG

>**Gmax_MSTRG.6400.1** GeneID=Gmax_MSTRG.6400;Strand=+;Pos=Chr02:7929647-7930398,

CTCGCAGGCCCACCACTTAAGACTGTATGACAGATAAGTGACCATGTTGGCCAAATCATT

ATGATCGTGTTGCAAAATCTTCTCAAAATACTCAAGACTTTTTTTTTTTGTATTGGCAGA

AATAAAAAATAGTATAAGGAGATTTATTTTAATTAATAAAATATTCTTAAAAAAAAGTGT

ATGGATAGCAAAGCGAGAGAATAGTTACCAAACCATGACTGCTGCTGGGACAGCAAAGCG

AAAGAACTCTCCAACACCAACCAAAGCATTCTTGGAGAAAGAGATGCGTGTTTTCTCACA

AGCAGAGGAATACCTCACAAAGGACAAAAGCATTATCACATTAAACCAAACGCAAAGGCT

GAAGGAGATTGCGGCACCAACATGTCCCAACCCCAGTTTAAAAACAAGGGTCCAACATGT

TACTACGTGGAAGCACAAAGCTATAGCGGAGGTTAGAATCATGGGAGAAATCAAACTCTG

GGTCTGGAAAAAATCGTGTTAGAGGTTTGAGAATTGCGGAACCAAATAGAGCAGGTATTAG

CCAAATGGCATATTTACGAACTTCAAGGGATATTGTTGGGTCTTGGCCTAATAGAGTTAA

TATTTTGTCATTGAAAATCCATAGAATTGTGATCGGGAAACAAACCAAAGAAAGGGATAT

TATGGCAGTGTAAGTATATAGTCCAAATTTTTCATATTGCCCTGCCCCGAAAGCTTGGCC

ACATAAAGTTTCCAATCCACCAGCCATCCCTG

>**Gmax_MSTRG.10877.1** GeneID=Gmax_MSTRG.10877;Strand=+;Pos=Chr03:546888-547123,

GAGAGTACAGGTTGAACACTGTTCAGTAATATAGAGACCGACAACAAAGGTGACAAATCC

CCAACAGCAAAGGCCACCTCTTTATTTGAGGTAAATATATATGCAAGTCTTTCTCTAAAA

AATAAGAAGAATATGAACAGAAGGAATCCGATGGCCAATGATGTGAGCACTGACACAATA

ATAGAGAACTTTGCAGCTTTGGCACTTCCTCTTCCAAGTTCATTTGCCACTCGAAC
