# Supplementary material for: The Identification of MATE Antisense Transcripts in Soybean Using Strand-Specific RNA-Seq Datasets
Source: Genes (Basel). 2022 Jan 26;13(2):228. doi: 10.3390/genes13020228 (PMC8871956; doi:10.3390/genes13020228)
Supplement: Supplementary file 1 [file genes-13-00228-s001.zip › Supplementary Table S1.pdf]

Table S1. List of strand-specific RNA-seq datasets of *Glycine soja* [1] used for transcriptome assembly and *MATE* antisense transcript identification.

BioProject/SRA Accession	Soybean germplasm	Tissue type	Condition
SRP158454 [2]	W05	Embryo, cotyledon and hypocotyl, root, apical bud, stem, flower, 7-day pod, 14-day pod, 14-day seed, 40-day pod, 40-day seed	Normal
		Nodule, root	Roots were treated with *Sinorhizobium fredii* CCBAU 25509 or *Sinorhizobium fredii* CCBAU 45436 to form nodules
PRJNA281671 [3]	C1, C3, C4, C5, C6, C7, C8, C9, Y1, Y2, Y3, Y4, Y5, Y6, Y7, Y8, Y9, Y10	Seed	Normal
PRJNA369483 [4]	ys1, ys16, ys17, ys23, ys26, ys29, ys34	Seed	Normal
PRJNA336336 [5]	PI407179, PI424007, PI424123, PI507656	Leaf	Ozone

References

1. Lin, X.; Lin, W.; Ku, Y.-S.; Wong, F.; Li, M.-W.; Lam, H.-M.; Ngai, S.-M.; Chan, T.-F. Analysis of soybean long non-coding RNAs reveals a subset of small peptide-coding transcripts. *Plant Physiol.* **2020**, *182*, 1359–1374.

2. Xie, M.; Chung, C.Y.-L.; Li, M.-W.; Wong, F.-L.; Wang, X.; Liu, A.; Wang, Z.; Leung, A.K.-Y.; Wong, T.-H.; Tong, S.-W.; et al. A reference-grade wild soybean genome. *Nat. Commun.* **2019**, *10*.

3. Lu, X.; Li, Q.-T.; Xiong, Q.; Li, W.; Bi, Y.-D.; Lai, Y.-C.; Liu, X.-L.; Man, W.-Q.; Zhang, W.-K.; Ma, B.; et al. The transcriptomic signature of developing soybean seeds reveals the genetic basis of seed trait adaptation during domestication. *Plant J.* **2016**, *86*, 530–544.

4. Yu, C.; Qu, Z.; Zhang, Y.; Zhang, X.; Lan, T.; Adelson, D.L.; Wang, D.; Zhu, Y. Seed weight differences between wild and domesticated soybeans are associated with specific changes in gene expression. *Plant Cell Rep.* **2017**, *36*, 1417–1426.

5. Waldeck, N.; Burkey, K.; Carter, T.; Dickey, D.; Song, Q.; Taliercio, E. RNA-Seq study reveals genetic responses of diverse wild soybean accessions to increased ozone levels. *BMC Genomics* **2017**, *18*, 498.
