# Supplementary material for: The Identification of MATE Antisense Transcripts in Soybean Using Strand-Specific RNA-Seq Datasets
Source: Genes (Basel). 2022 Jan 26;13(2):228. doi: 10.3390/genes13020228 (PMC8871956; doi:10.3390/genes13020228)
Supplement: Supplementary file 1 [file genes-13-00228-s001.zip › Supplementary Table S2.pdf]

**Table S2.** List of primers used in this study.

| **Primer[#]** | **Primer sequence (5'-3')** |
|---|---|
| MSTRG.9425 RTq-PCR forward (*Glyma.03G0018400* RT-qPCR reverse) | CCGTGACAAGAGTTGGTTGTT |
| MSTRG.9425 RTq-PCR reverse (*Glyma.03G0018400* RT-qPCR forward) | CAGCTTCATGGTACTGTGCCT |
| MSTRG.47510 RTq-PCR forward (*Glyma.11G026300* RT-qPCR reverse) | GCTTAACAGGTCCAACTTTGAGT |
| MSTRG.47510 RTq-PCR reverse (*Glyma.11G026300* RT-qPCR forward) | AGATCCAACGCAGAGACCAA |
| MSTRG.45962 RTq-PCR forward (*Glyma.10G287600* RT-qPCR reverse) | TTGTCCTCGTCTGCCTGTGA |
| MSTRG.45962 RTq-PCR reverse (*Glyma.10G287600* RT-qPCR forward) | GGACGGGTACTTGTCTGATGT |
| MSTRG.5534 RTq-PCR forward (*Glyma.02G097800* RT-qPCR reverse) | TTGTTCCTGGTCTCTGGTGT |
| MSTRG.5534 RTq-PCR reverse (*Glyma.02G097800* RT-qPCR forward) | GGTTCACCTGAATGGCTTGTG |
| MSTRG.84397 RTq-PCR forward (*Glyma.19G120900* RT-qPCR reverse) | AAGCTACTTTTTCTCTTGCTGC |
| MSTRG.84397 RTq-PCR forward (*Glyma.19G120900* RT-qPCR forward) | ACATGAAAAACGCCGAGGTTC |

[#]The forward primer for antisense transcript was used as the reverse primer for the corresponding sense transcript and *vice versa*.
